# Community context and sub-neighborhood scale detail to explain dengue, chikungunya and Zika patterns in Cali, Colombia

**DOI:** 10.1371/journal.pone.0181208

**Published:** 2017-08-02

**Authors:** Amy R. Krystosik, Andrew Curtis, Paola Buritica, Jayakrishnan Ajayakumar, Robert Squires, Diana Dávalos, Robinson Pacheco, Madhav P. Bhatta, Mark A. James

**Affiliations:** 1 Department of Biostatistics, Environmental Health Sciences, and Epidemiology, College of Public Health, Kent State University, Kent, OH, United States of America; 2 Health & Hazards Lab, Department of Geography, Kent State University, Kent, OH, United States of America; 3 Grupo de Investigación en Epidemiología y Servicios (GRIEPIS), Universidad Libre, Cali, Colombia; 4 Department of Public Health and Community Medicine, Universidad ICESI, Cali, Valle del Cauca, Colombia; 5 Center for Clinical Research, Fundación Valle del Lili (FVL), Cali, Valle del Cauca, Colombia; Cary Institute of Ecosystem Studies, UNITED STATES

## Abstract

**Background:**

Cali, Colombia has experienced chikungunya and Zika outbreaks and hypoendemic dengue. Studies have explained Cali’s dengue patterns but lack the sub-neighborhood-scale detail investigated here.

**Methods:**

Spatial-video geonarratives (SVG) with Ministry of Health officials and Community Health Workers were collected in hotspots, providing perspective on perceptions of why dengue, chikungunya and Zika hotspots exist, impediments to control, and social outcomes. Using spatial video and Google Street View, sub-neighborhood features possibly contributing to incidence were mapped to create risk surfaces, later compared with dengue, chikungunya and Zika case data.

**Results:**

SVG captured insights in 24 neighborhoods. Trash and water risks in Calipso were mapped using SVG results. Perceived risk factors included proximity to standing water, canals, poverty, invasions, localized violence and military migration. These risks overlapped case density maps and identified areas that are suitable for transmission but are possibly underreporting to the surveillance system.

**Conclusion:**

Resulting risk maps with local context could be leveraged to increase vector-control efficiency- targeting key areas of environmental risk.

## Introduction

### Global health relevance

Zika [1], dengue [2–5] and chikungunya [6, 7] are arboviral diseases transmitted by the bite of an infected *Aedes spp*. mosquito. Zika can also be transmitted through sexual contact [8–10]. Individually, each represents an important global public health threat and are often co-endemic.

Dengue, chikungunya, and Zika research in Colombia has generated considerable geographic investigations, mostly focused on spatial and temporal patterns of either mosquito intensity or human case data [11–21]. However, there is still a need for greater understanding at a granular sub-neighborhood scale that incorporates specific features such as houses, streets, standing water and other environmental risks [22, 23].

### Study location

The metropolitan city of Santiago de Cali, Colombia (Cali) is 122 Km from the Pacific Coast of Colombia in the department of Valle de Cauca with a population of 2,181,317 in 2010 [24]. The major ethnic populations are Afro-Colombian (26%), Caucasian and mixed (73.3%), and indigenous groups (0.5%) [25]. Of relevance to the dengue situation in the city are the high levels of poverty and violence; 26.1% of the population in Cali living in poverty [24], and the primary cause of death is homicide (including traffic accidents) [24]. The tropical climate, with a median temperature of 24.6°C and annual precipitation of 1,588 mm [24], is highly suitable for mosquito breeding. These climatic conditions favor vector production during any season. Additionally, the study was carried out during an El Niño year which caused a longer dry period than expected. Drought conditions may stimulate human water storage that creates suitable breeding sites for container-breeding vectors such as *Aedes* [26, 27]. Additionally, warmer conditions may contribute to transmission by reducing the intrinsic incubation period [28].

### Suitability of study location

The City of Cali provides an excellent study location to show the importance of a granular perspective on dengue, chikungunya, and Zika. It is recognized as having a mosquito-borne disease problem, exacerbated by historical and ongoing violence which has disrupted health protection. The city has a wide variety of neighborhood types, and a rich history of dengue-related research, which has considered the various aspects of disease presence, and the associated risk factors in an attempt to better inform intervention strategies [22, 29–32]. For example, Delmelle and colleagues [33] used geographically weighted regression to tease out neighborhood scale variations within the city. Their work not only included a rich array of input variables (cemeteries, 49 plant nurseries, 23 water pumping stations, 499 tire shops, 4 rivers, and green spaces), but also incorporated local expert insight in terms of what they initially needed to consider in analysis. Even given their results, which validated many other similar findings with regards to the vulnerabilities posed by human density and socioeconomic status, they acknowledged that richer local and contextualized data are required. The authors further concluded that the quality of surveillance data in the poorest neighborhoods suffered for a variety of reasons.

This last point is of particular concern if neighborhoods vulnerable to arboviral infection coincide with perceived and actual security risks [24, 34] which may limit vector control work^1^. Human case data (at least in Cali) is likely to be underreported in poor areas due to a lack of clinical testing as a result of impediments to access or a lack of confidence in the health care system^2^. Of case data that are available, the most marginalized areas (unplanned urbanization and invasions) are least likely to be successfully geocoded to an actual or proximate address [33]. Even when hotspot analyses are performed, issues of how to contextualize the results remain. For example, a neighborhood may present as a hotspot–but why? More specifically, why does the east side of the canal appear to be more susceptible to disease than the west? This hints at a more complex causation than simple proximity. Variation also occurs along the course of the feature as the flow of water may be disrupted by engineering work, increased trash (especially around informal housing) or edge vegetation, all of which can increase the risk of mosquito breeding^3^.

Potentially as important as having more granular data is the addition of local insight to contextualize risk. For example, in their study on mosquito control in a medium-sized U.S. city, Curtis et al. used SVG to capture institutional knowledge from local vector control experts: spatially specific insights from those who had worked for a number of years in the area and who had amassed valuable information on topics such as which locations cause mosquito outbreaks; which species were involved; what control measures had been successfully used; and what were the proximate human diseases [35].

### Underreporting and lack of access to care

Cases of arboviral infection are routinely under-reported in this region due to complex social, economic, and political reasons. The use of health services, and subsequent case reporting, is influenced by access and external environment. Access defined as the fit between the patient and the health care system is determined by 5 factors: availability, accessibility, accommodations, affordability, and acceptability [36]. Increased access and decreased cost is expected to lead to increased formal care/treatment-seeking behavior, and thus increased positive outcome. In the developing context, health outcomes differ widely by treatment choice [37].

In this study site, some proximate barriers to health care access and case reporting include to health care access, especially in the areas that are most at risk of arboviral infection (lack of trust in health care system; feeling that the clinics will only give pain medicine and hydration recommendation instead of cure; lack of paid time off or sick leave to attend clinic; lack of resources to confirm diagnosis with laboratory testing); or limited public health resources for data management and reporting. These may hold true in other regions with endemic transmission of one of these asymptomatic arboviruses. For example, one study in Brazil estimated 12–17:1 dengue cases per reported case in the community [38], with comparable results observed in Nicaragua, Thailand, Cambodia, Brazil, Colombia, and Mexico [39].

We suspect systematic underreporting by region according to access to health services related to SES as also reported by Sarti et al, where in Colombia, incidence rates for confirmed dengue were 5.8 times higher in the independent study compared to local state and 3.5 times higher compared to local levels [39]. For example, chikungunya cases in this sample represented an estimated 5% of total cases (MoH, personal communication).

### Need for a granular scale approach

As discussed by Curtis et al and Smiley et al, [40–42], there is a need for fine scale data on disease incidence, risk, and environmental variables to facilitate understanding of local disease ecology, epidemiology, and points of intervention which may vary over space and time. Smiley et al [42] mapped the water-fetching paths from Dar es Salaam, Tanzania to show how environmental and physical health risks vary over space. In Haiti, environmental layers (standing water, trash and animals) were mapped at a granular scale to help explain urban cholera patterns and then support epidemiological field testing [40, 41].

Surveillance data and the correlated environmental and socioeconomic data are frequently not available at fine scales that would support intervention. For example—knowing that a city, or part of a city has high dengue rates is useful for an overall impression—but does not help in understanding where the local drivers of disease are located (for example, a single canal), and where to intervene.

Interpreting these environmental risks at the neighborhood level, context is added to more traditional geographic results [33, 43–45]. For example, one might ask questions about how local human behavior increases or mitigates disease risk [45, 46], even when environments appear similar; or the local perception of disease risk within hotspots identified by traditional surveillance. Using this contextual approach, an already vibrant research agenda focused on dengue, chikungunya, and Zika in Cali, Colombia is enriched.

Most spatial dengue work involves the identification and explanation of geographic patterns of disease-carrying mosquitoes, or human surveillance data [29, 45, 47–49]. Causation and correlation with these patterns involves the incorporation of various spatial layers of which environmental (including moisture and vegetation), built environment (infrastructure), climatic (micro and macro), entomological, and human (density, social, behavioral, political and disease surveillance) are the most common [43, 46]. Adding complexity to these studies is an issue of geographic scale and the realization that the pattern-cause/correlation nexus will vary geographically; between countries, regions of countries, cities, and even within cities [49]. Though there are still accepted universal risk factors for *Aedes* spp. arboviral transmission, such as human density, various measures of economic hardship [29, 33], and proximity to urban environmental “risk” features such as canals and ditches, these will vary in importance across the same urban space due to nuances in the “attributes” of each, such as how the micro environments of the same canal will change risks along different stretches. Adding spatial and temporal dynamism to these findings are intervention methods such as mosquito control and disease education strategies [50–52]. Spatial research at a more granular *scale of intervention*, especially in combination with on-the-ground perceptions and insights, can help explain these findings and what traditional data have missed [46]. This granular scale is the focus here, showing how street level environmental data extracted from spatial video (SV) and Google Street View (GSV), and mapped expert insight captured by spatial video geonarratives (SVG) can be used at any location to better understand disease risk. We will use examples taken from these expert narratives throughout the paper to illustrate the various points being raised (**S3 Text. SVG Interview Footnotes**).

### Spatial video and spatial video geonarratives

Spatial video (SV) has been used to map various types of challenging and data-poor environments [53, 54]. In Haiti, environmental layers (standing water, trash and animals) were mapped at a granular scale to help explain urban cholera patterns and then support epidemiological field testing [40, 41]. Basic data layer creation involves watching spatially encoded video and then digitizing risk factors into either Google Earth or a geographic information system (GIS). While this does not replace more traditional epidemiological approaches, it can complement such methodologies with local spatial detail, especially in spatial-data-poor environments. The SVG method is SV with an audio recorder added to capture “expert” insight during data collection. Software written at Kent State University is then used to produce mapped layers from these transcribed commentaries. If there are enough SVGs (the depth scenario), key words can be queried out as a point layer and used as input for more traditional spatial analysis [55]. Alternatively, a single expert is used to cover multiple spaces (the breadth scenario) and to provide institutional insight where none exists [35]. For an investigation into dengue, these narratives can be used to gain further granular insight into a neighborhood or area known to be a hotspot, with the identification of specific features or even behavioral / political actions that increase vulnerability.

**Fig 1:**
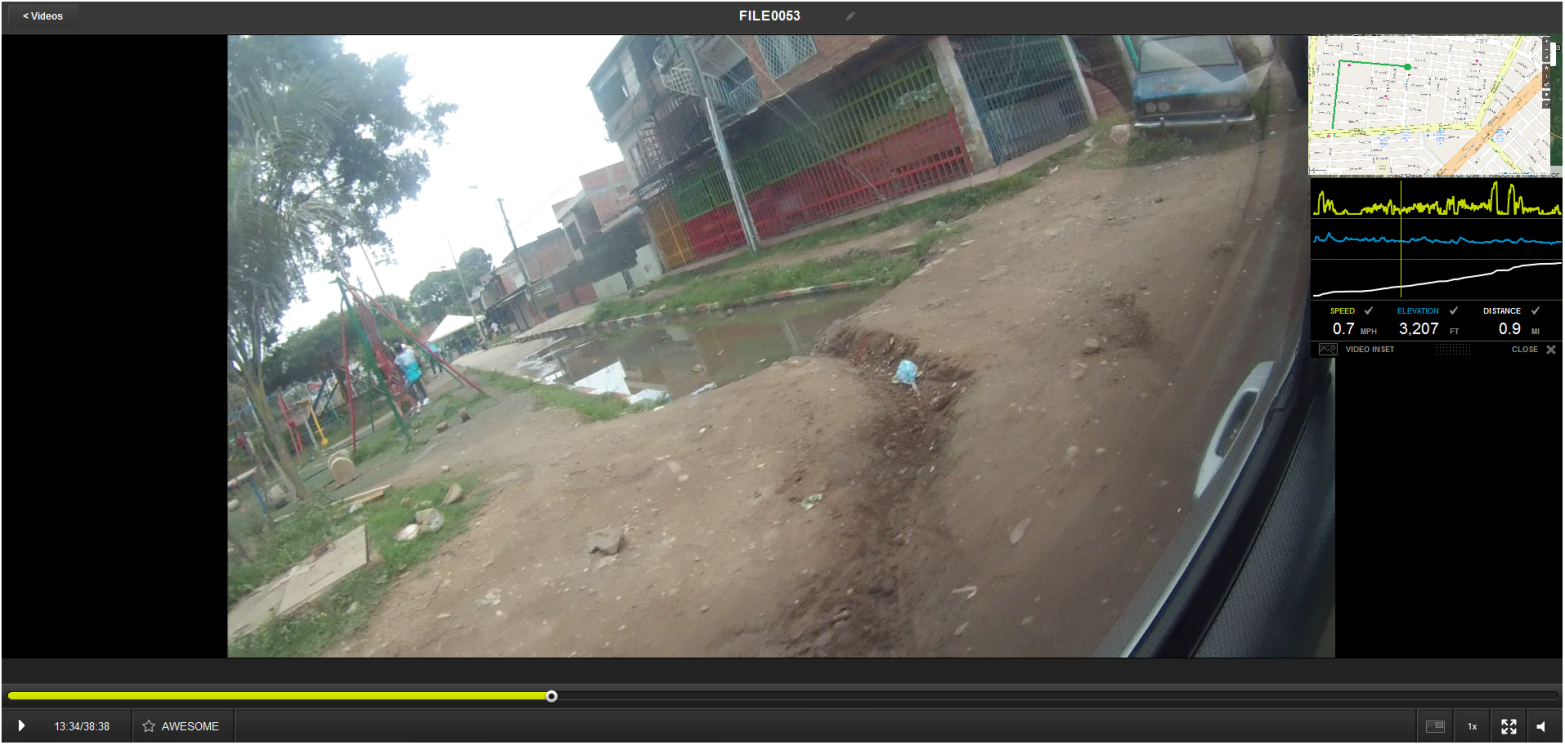
A spatial video risk example. Note presence of both a potential breeding site and vulnerable human activity.

There are three types of insight gained from either the depth or breadth SVG: *Spatially specific*, where an exact location is identified, such as a collection of abandoned tires. These data are usually described as the vehicle passes the location, and while there is an uncertainty space with regards the geographic extent of the comment, the final point on the map is still near the actual risk. The ability to return to the video for visual validation not only helps in contextualizing the comment, but can also reduce the uncertainty of the location to an exact place. For example, Fig 1 displays a small drainage canal which is described as problematic for mosquito breeding while also being proximate to a child play space.

*Spatially proximate*, is a general risk space identified from a location's sights sounds and smells even though no precise place is identified. Note, while previous research has stressed the visual side stimulating comment, it is all the senses working together. This is especially true with smells being an indicator of trash and stagnant organic filled water. An example could be the description of how rainfall would often flood this part of the neighborhood which, in combination with unfinished road surfaces, would produce small pools for breeding. *Spatially inspired* or *fuzzy spaces*, contain information that emerges during the SVG ride about the topic under investigation without referencing spatially specific locations. Some examples of s*patially inspired* or *fuzzy spaces* from this study include insights into the challenges facing vector control^4^; the experience of living with dengue, or chikungunya or Zika^5^; and limitations in the local disease surveillance system^6^. However, even with these there can still be a geographic stimulus–for example being in an economically disadvantaged neighborhood could result in comments on vector control or disease surveillance, *for that type of neighborhood*.

### Dengue

Dengue fever, responsible for 390 million infections per year [56], is caused by one of four dengue virus (dengue) serotypes of the family *Flaviviridae*. *Aedes aegypti* mosquitoes are known to be the primary vector of the virus, with *Aedes albopictus* transmitting another ~100 million cases per year [57].

Control efforts had been relatively successful during the middle of the last century but a variety of deficiencies in control beginning in the 1970s, culminating in the failure of the PAHO mosquito eradication program (particularly in areas experiencing conflict and violence) allowed the reemergence of the disease [58–61]. One such urban example of this reemergence and hypoendemicity is the metropolitan city of Santiago de Cali, Colombia (Cali), which experienced an epidemic of dengue in 2013 with 13,433 cases of dengue reported and a cumulative incidence of 56.7/10,000 habitants (95% CI = 55.7–57.6) and over 18,000 cases reported to the National System of Vigilance in Public Health [SIVIGILA] [62], in the department of Valle de Cauca between week 1 and week 48 of 2015 [56, 63].

In the year 2010, there was a major dengue outbreak in Colombia, with more than 150,000 cases and 289 deaths reported in the country. More than one million cases were reported in all of Latin America [64]. In Cali, Colombia, a week in July, 2016 recorded a total of 126 cases [65].

One review of epidemiology of dengue in Colombia concluded that “dengue disease in Colombia was characterized by a stable ‘baseline’ annual number of dengue fever cases, with major outbreaks in 2001–2003 and 2010. The geographical distribution of dengue disease cases showed a steady increase, with most of the country affected by the 2010 outbreak” [66]. Case fatality rates were high in the 1990s, declined between 2000–2009, and increased greatly in 2011. The geographical range increased from 2000–2010. Most dengue occurred in those <15 years of age, with a majority of cases in children <1 in 2009 [67]. In the department of Valle del Cauca, the number of cases increased from 2010 to 2015 [68], possibly due to improvements in diagnosis and clinical management [67].

Dengue is not the only mosquito-borne problem in the city. For example, an outbreak of chikungunya occurred between September 2014 and September 2015, resulting in endemic transmission. Chikungunya transmission occurred simultaneously with dengue by the same vector species, putting the same host populations at risk.

### Chikungunya

An estimated 1.3 billion persons live in areas where chikungunya can be transmitted [69]. Chikungunya was responsible for 1,386–1,081,962 years of life lost in 2005 [70]. There is increasing global concern over expanding distribution [71–76]. Since introduction in 2013, CDC estimates more than 1.7 million suspected cases in 45 countries in the Americas [74]. However, there is confusion between probable and confirmed cases [77] as the disease is often co-endemic and confused with dengue fever [73, 78, 79] and Zika [80].

The 2014–2015 chikungunya outbreak affected 1.5 million people in the Americas with cases of atypical mortality reported in Colombia [81]. The outbreak in Colombia occurred between September 2014 and September 25, 2015 [82], with a total of 439,000 cases reported in 712 municipalities [82–84].

Cali was one of the most affected regions in the country with 44,877 accumulated cases reported up to October 17, 2015 [85] due to the Asian genotype [86]; 63.6% of cases were reported among females and 11.3% among 25 -29-year-olds [87].

### Zika

Zika is a reemerging vector-borne viral disease declared to be a public health emergency of international concern in 2016 by WHO [88]. Zika was reclassified as a continued threat November 18, 2016 [89]. Zika virus is a flavivirus known to circulate in Africa, the Americas, Asia, and the Pacific with recent outbreaks in the Americas [90].

Zika was imported to Colombia from Brazil with the first new cases reported in October, 2015 [91], resulting in 20,297 suspected and 1,050 laboratory-confirmed cases reported by the National Institute of Health between epidemiological week 40 of 2015 and week 3 of 2016 [92]. Colombia declared a Zika outbreak in October 2015. Through April 2016, 3,139 suspected cases of Zika were reported to the Secretary of Health in Cali [62]. Surveillance started August 2015. 65,726 cases reported in Colombia by April 2, 2016, (4% confirmed by RT-PCR) with females reporting 2x as much as males. 11,944 pregnant women reported with Zika, 14% confirmed by RT-PCR. 66.10% of reported Zika cases were females with 63.57% from the contributory scheme and 14.09% among 25 to 29-year-olds, 2.66% among infants one-year-old or less, and 3.24% among adults 65 or older. 0.68% were reported among indigenous groups and 1.83% among Afro-Colombians [87].

Geographic distribution of Zika incidence was reported using SIVIGILA data at the municipality level in Valle del Cauca and at the communa level in Cali (23), reporting that more than a third of all Zika cases reported in Valle del Cauca were concentrated in Cali, the capital city. Incidence rates reported were between 250 and 499 cases/100,000. However, fine scale incidence at the neighborhood level was not reported.

## Methods

This study was approved by the appropriate local (Universidad ICESI #061) and University institutional review boards (Kent State University #15–529). Human case data are presented at the aggregate neighborhood level and individuals are not identifiable.

### Preliminary risk mapping

**Fig 2:**
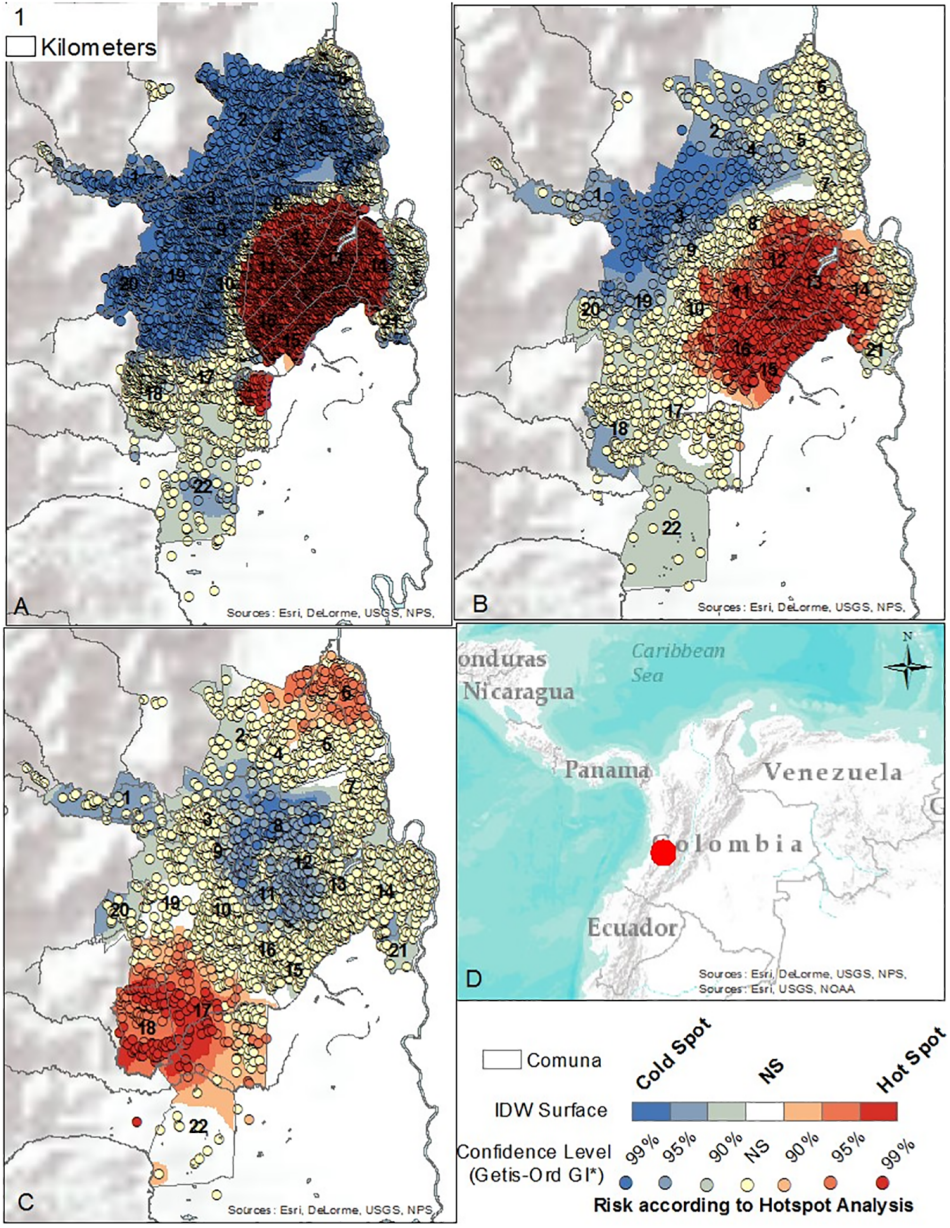
Hotspot maps. A. Dengue Hotspots, Bandwidth = 2500 meters. October 2014—April 2016. B. Zika Hotspots, Bandwidth = 3400 meters. November 2015—April 2016 C. Chikungunya Hotspots, Bandwidth = 2500 meters. January 2015—April 2016. D. Cali, Colombia.

**Fig 3:**
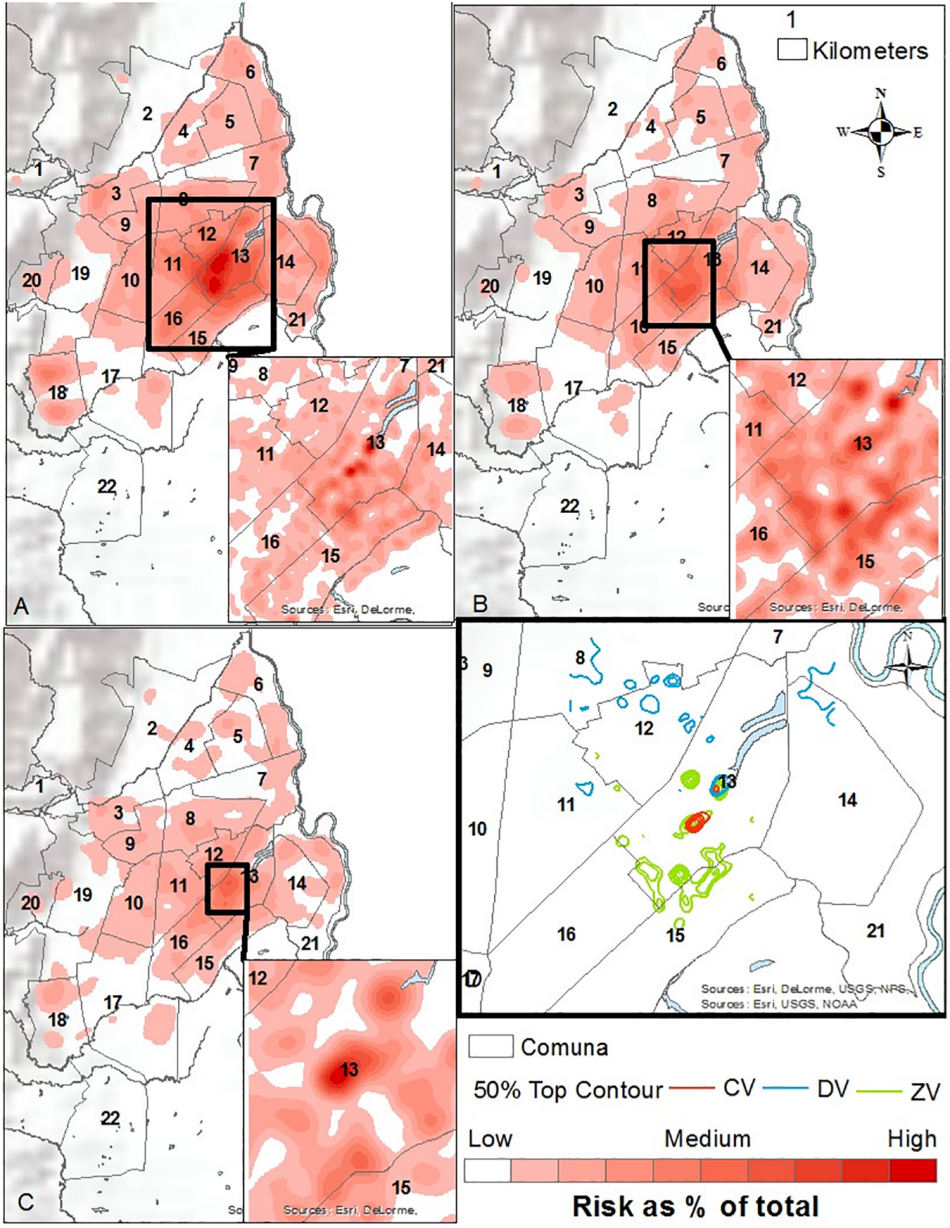
Kernel density analysis. A. Dengue Kernel Density Analysis, Bandwidth = 600/200 meters. October 2014—April 2016. B. Zika Kernel Density Analysis, Bandwidth = 600/200 meters. November 2015—April 2016. C. Chikungunya Kernel Density Analysis, Bandwidth = 600/200 meters. January 2015—April 2016. D. Top 50% Contours of Risk by Disease.

To determine where to collect sub-neighborhood scale data, and where to conduct contextual interviews, a preliminary mapping (**S1 Data. Folder A in S1 Data. Shape files and Files 1, 2, and 3: Case data aggregated to neighborhood level with neighborhood characteristics and Folder B. Geodatabase of shapefiles.**) of dengue, chikungunya, and Zika human case data reported between October 2014 and April 2016 to the national database, SIVIGILA, was performed. Of note, these diseases are historically under-reported to SIVIGILA in Cali. Vector data were not available. After georeferencing using the Secretary of Health Software, a Getis-Ord General G statistic was performed in ArcGIS 10.3 at 2500, 2500, and 3400 meter bandwidths, respectively (chosen to optimize spatial autocorrelation analysis); and Kernel Density at 600 and 200 meter bandwidths for full extent and zoomed analyses, respectively (Figs 2 and 3). The resulting hotspot maps are not interpreted here beyond showing that chikungunya did not overlap Zika and dengue as expected, whereas in the non-parametric kernel density analysis, the highest risk areas overlapped for Zika, dengue, and chikungunya.

These maps were used to plan a subsequent series of 107 semi-structured interviews in 26 neighborhoods during a drought (October 14 –December 1, 2015 and January 25 –February 9, 2016) and rainy season (April 9–16, 2016) with vector specialists, community health nurses, program leaders and local community leaders and members. Participants recommended other leaders for interviews, leading to a snowballed sample. Some interviews were conducted using focus groups at homes, local community centers, and during group walks to areas of interest. In each case, the investigator started the interview with an explanation of the goals and aims of the research, obtained written informed consent, and asked the participant to describe the environmental or social risks for dengue, chikungunya, and Zika in the neighborhood. The research subject then guided the interview in terms of content and geography, only prompted by the investigator with follow-up questions if further clarification was needed. A list of questions was available if needed for the investigator to prompt the interviewee (**S2 Text. SVG interviewee prompts**) on specific environmental risk factors in the neighborhood.

### Spatial video geonarrative interviews

**Fig 4:**
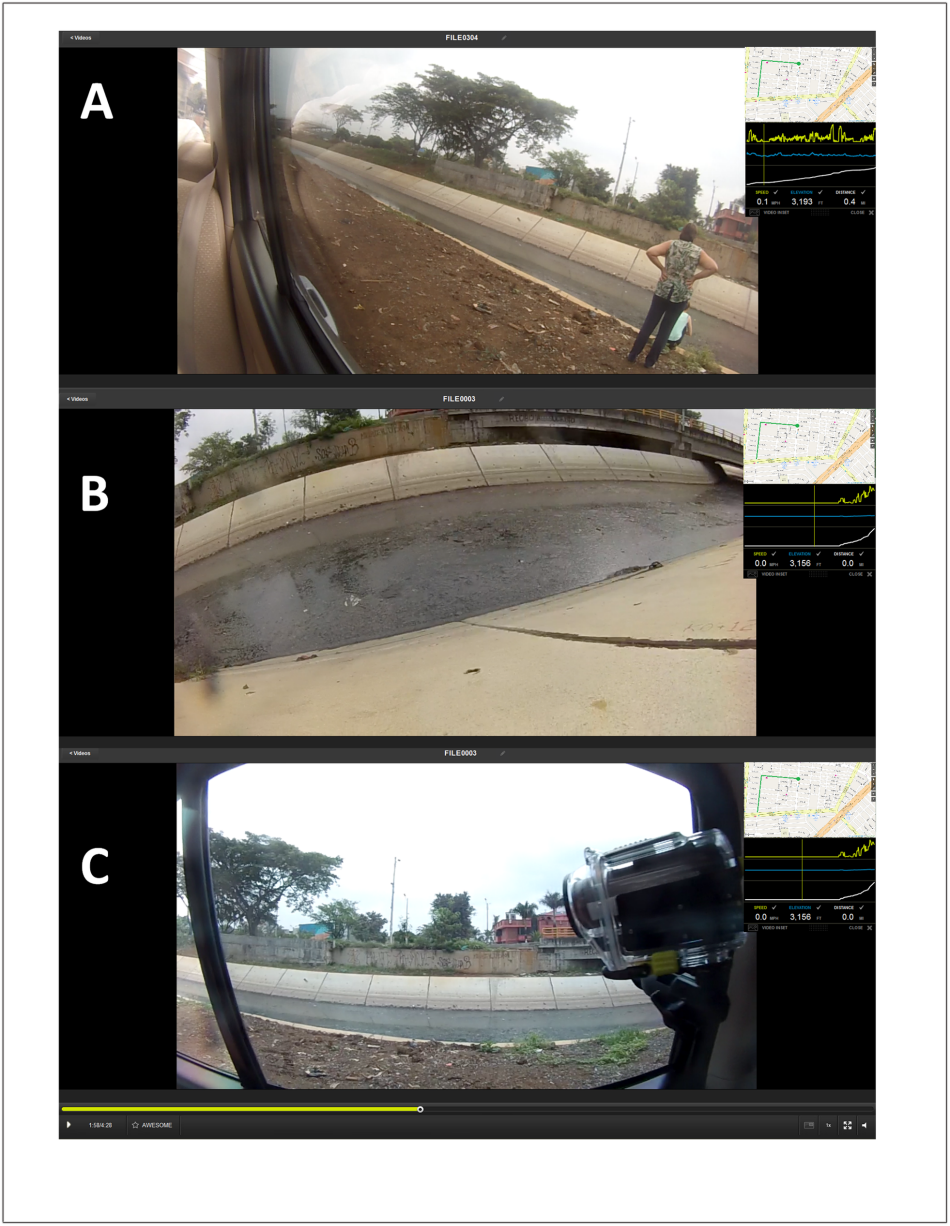
SVG interview camera setup. A and B: Two Contour 2 cameras mounted on the inside left and right window of a vehicle- angled down to capture the conditions by the side of a road); C: hand-recording of mosquito larvae by the two individuals seen in Fig B.

For the SVG interviews, two Contour 2 cameras, designed for extreme sports usage, were mounted on the inside left and right window of a vehicle using a suction mount. The cameras were angled down to capture the conditions by the side of a road and to limit the uneasiness of passersby who might not want to be recorded. Fig 4A displays the location of the camera on the window, while Fig 4B shows the image captured by this camera at this location. During the drive, stops would occasionally be made if the environment was not suitable for vehicles, or if added detail was needed regarding a key location (Fig 4C). On these stops, one camera was removed and hand-carried to record local conditions. Fig 4C shows the hand-recording of mosquito larvae by the two individuals seen in Fig 4A. The expert’s commentary was primarily captured by the recorder, but his/her voice was also on the video. This was important for syncing the eventual transcription time stamp with those of the video and GPS.

Each ride would usually capture more than one neighborhood. The length of ride varied by several factors, including size of neighborhood, accessibility within the neighborhood, and perceived safety issues.

### Data analysis

#### Transcription, translation, and interpolation

After data collection, the video was downloaded from each camera in Colombia. Upon returning to the United States, the audio of each SVG was translated from Spanish and the earliest part of the transcription that could be matched to the video was used as a starting point for the word interpolation. For this to happen the same transcribed word had to be clearly heard on the video, and the GPS had to have been acquired for that video segment. The software Contour Storyteller (examples are seen in Figs 1 and 2) allowed the simultaneous viewing of GPS and video. The transcription (as a text file), GPS (as a CSV file), and the six digit GMT time were entered into Wordmapper, a specially written computer program created in the GIS Health and Hazards Lab at Kent State University. Output from Wordmapper includes a GIS shapefile of words, where each word is interpolated over the time range between two conversation time stamps, and a shapefile of "comments" where each conversation segment is attached to the coordinate of the beginning word. The reason for these two outputs is that these allow for a single word to be queried in the GIS, while also spatially important conversations can be extracted around an object being discussed.

#### Kernel density estimate (KDE)

After each neighborhood had been mapped by spatially encoded words and geo-located comments, a series of exploratory analyses were used. The word file was queried for similar risk factors and these were mapped using a kernel density estimate (KDE) to aid in the visual interpretation. KDE is a commonly applied technique in epidemiology, with several examples using mosquito-related data, including previous research in Cali, Colombia [32, 33, 93]. KDE has also been used to map out SV and SVG data [40, 55]. Each KDE was classified into ten equal breaks, and these were used to determine the contouring bands.

#### Coding spatial insights

Each transcription was also read for general insights, and then spatially specific locations, especially those important to understanding local mosquito activity (such as a known “problem” park, or a stagnant water trench, or a discarded pile of tires). These spatial insights were identified in the GIS using the comments shapefile, and each object was buffered to 50 m. Key larger features identified in the commentary, such as a canal, were digitized by their shape and again buffered. In this way, a risk map for each neighborhood was created.

#### Digitizing risks

Concurrently to the SVG analysis, a few key neighborhoods of particular interest were digitized for risks seen in the video, using the same approach previously employed in Haiti [40]. SV was viewed on one screen while risk locations were digitized into Google Earth. Digitized points included water, trash (and tire) locations. The intensity of points captured the spatial extent of the risk, while the weight of each approximated the depth or volume. These Google earth layers were saved as KMZs and imported into Arc GIS 10.4 where the same KDE approach as previously described was used (50m kernel, classification of ten equal breaks).

### Google Street View

As Google Street View (GSV) has more frequently been used in research [35, 94, 95], a second mapping of the neighborhoods occurred using this as the data source. As it has been noted that one limitation of GSV is a fluctuation in time frames [96], all image time stamps were recorded.

## Results

**Fig 5:**
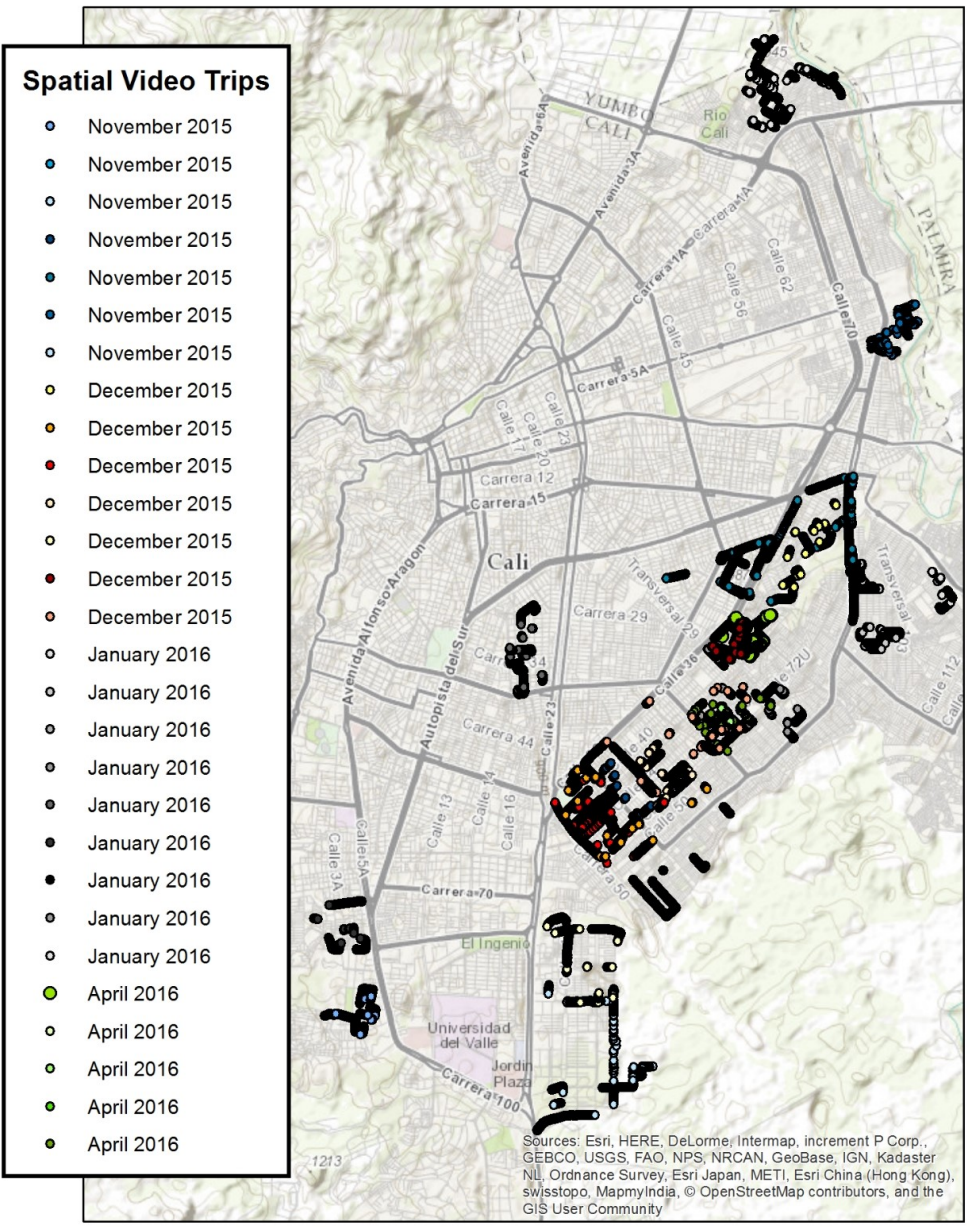
SVG routes. For each route, note the interview date given in the legend.

SVG rides were used to capture insights from “expert” passengers who had detailed information about dengue, chikungunya and Zika in 24 neighborhoods of Cali, Colombia. Note, GPS problems occurred in some neighborhoods, while in others the amount of commentary was too short for useful risk mapping. Only the “successful’ SVG are reported here. Interviewees included mosquito control experts, public health officials, and local leaders. For some rides, more than one expert was in the vehicle. Each ride began with the reading of an IRB consent script and obtaining informed consent. The path of the SVG was determined by the researcher selecting the neighborhood (using the hotspot and coldspot maps of Figs 2 and 3 as a guide), with the expert identifying which features were important to visit inside the area of study. In all cases the expert had a solid working knowledge of the neighborhood being studied. Fig 5 shows the overall spatial distribution of the rides. While most neighborhoods were covered only once, there were a few occasions where the neighborhood was repeatedly examined (Calipso) up to three times.

**Fig 6:**
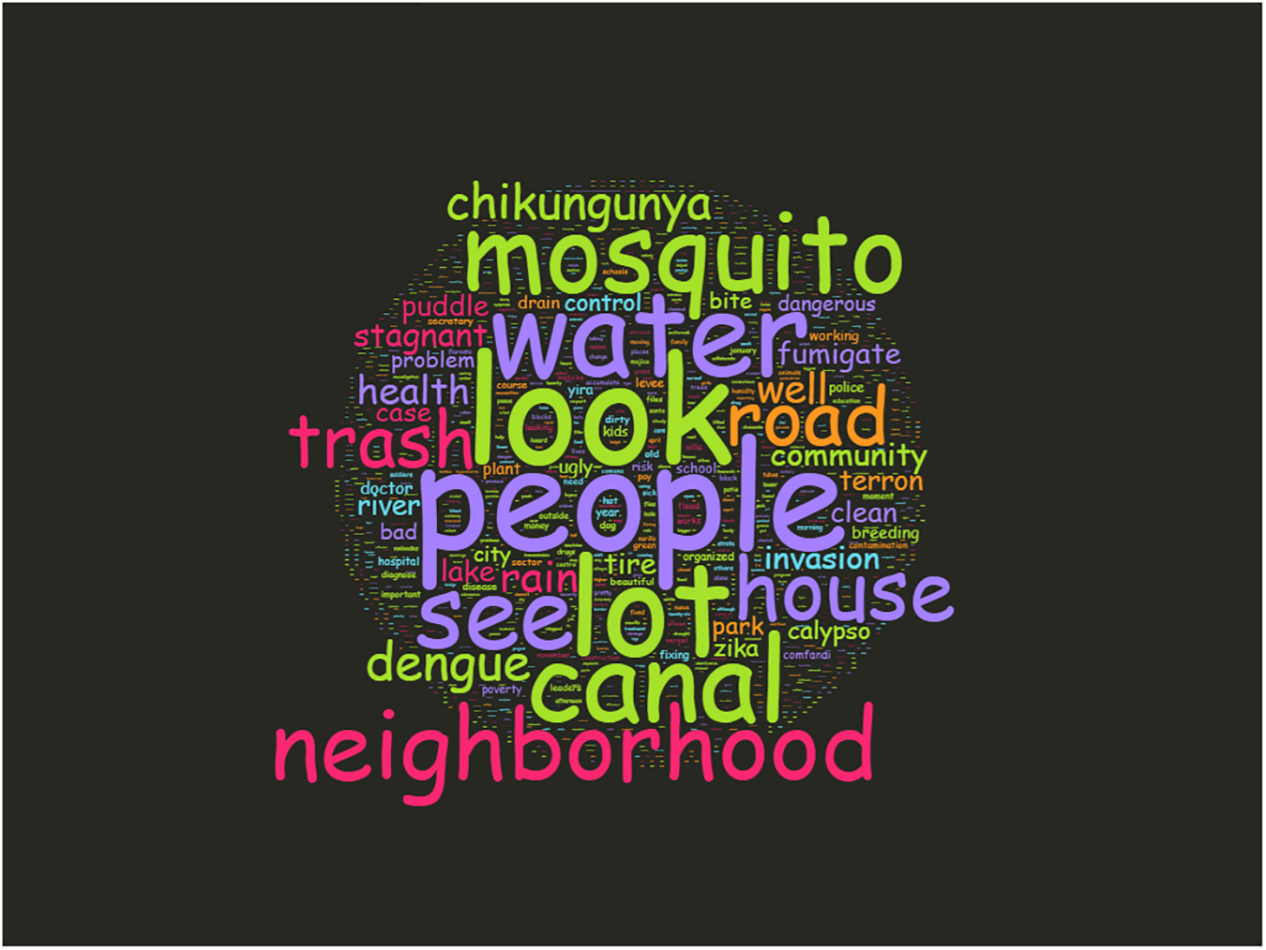
Word cloud of geospatial interview transcripts.

Fig 6 shows a simple word cloud of all SVG. From this, certain themes emerged, such as canal, trash, stagnant, and invasion. Also of interest is the emphasis on “people”; a common impression was the role people played in either exacerbating, or mitigating risk in their communities. The novel aspect of the SVG is that this is a spatialized word cloud–each of these words can also be mapped across the city.

Table 1 provides an alternative summary of the rides, by neighborhood, date, and the expert being interviewed. The length of each ride is also presented in terms of the total video length, and the total of spatially coded words. The number of spatially important mentions is also tabulated; these being identified locations that potentially play some role in the mosquito situation for each neighborhood. Finally, as an example of how the spatial word file can be investigated, a count is provided of “invasion” which is a local expression for an informal human settlement. This word is chosen because these invasions were frequently mentioned as playing an important role in the dengue situation, in terms of having poor infrastructure, more trash, less mosquito control (for both political and security reasons), and insufficient access to medical facilities, and as a result questionable surveillance data^7^ [39]. The SVG rides ranged from approximately 20–90 minutes, with the number of associated spatial words being loosely connected with trip length. One ride (Antonio Nariño) is split as the interview was broken into two segments.

**Table 1:** Spatial video and spatial video geonarrative field collections and resulting mapped length, words, and spatial mentions. VC = vector control specialist. MOH = Ministry of Health official. CHW = Community Health Worker.

Interviewee	Date	Length Mapped	Total Words	# Spatial mentions	# Invasions
VC and MOH	11/22/2015	71	2030	28	5
VC	11/26/2015	86	1291	19	0
VC	11/26/2015	84	2725	34	7
VC	11/26/2015	32	769	13	4
CHW	11/27/2015	24	962	11	0
CHW	11/27/2015	22	1436	9	0
VC	12/4/2015	37	657	4	4
VC	12/4/2015	65	1849	18	2
VC	12/4/2015	63	926	22	0
VC	12/4/2015	27	582	7	2
VC	12/5/2015	36	742	9	0
VC and MOH	1/15/2016	95	1873	23	5
VC	1/27/2016	25	534	3	0
VC	1/27/2016	21	729	7	5
VC	1/29/2016	22	609	11	0
VC	1/29/2016	28	1003	8	0
VC	1/29/2016	56	846	15	3
VC and CHW	1/29/2016	20			
VC and CHW	1/29/2016	80	3563	17	0
VC and CHW	1/30/2016	18	263	6	0
VC and CHW	1/30/2016	23	549	9	0
VC and CHW	4/10/2016	73	1375	15	8
VC and CHW	4/10/2016	44	859	16	1
VC and CHW	4/10/2016	42	933	30	0
VC	4/15/2016	28	1485	13	0

To demonstrate the utility of SV and SVG one neighborhood is highlighted, Calipso, which is medium social strata (3), just over 1.5 square km, and has a total population of 5,455 of which 30% are Afro-Colombian. While this neighborhood has many of the social and physical vulnerabilities that should indicate a high dengue incidence, it received 4.3 mm of rain during the study period and contains a canal; this is not reflected in surveillance outcomes [33].

The SVG for Calipso was transcribed and mapped using Wordmapper. From the resulting point shapefile, a KDE was performed on all words having some connection to water (canals, puddles, words describing flow, etc.), the output of which was classified into ten equal bands (to approximate percentage risk) and contoured. The resulting risk map shows a general risk surface based on water mentions where “risk” increases in 10% bands, and specific locations where the subject identified an importance to mosquito presence.

**Fig 7:**
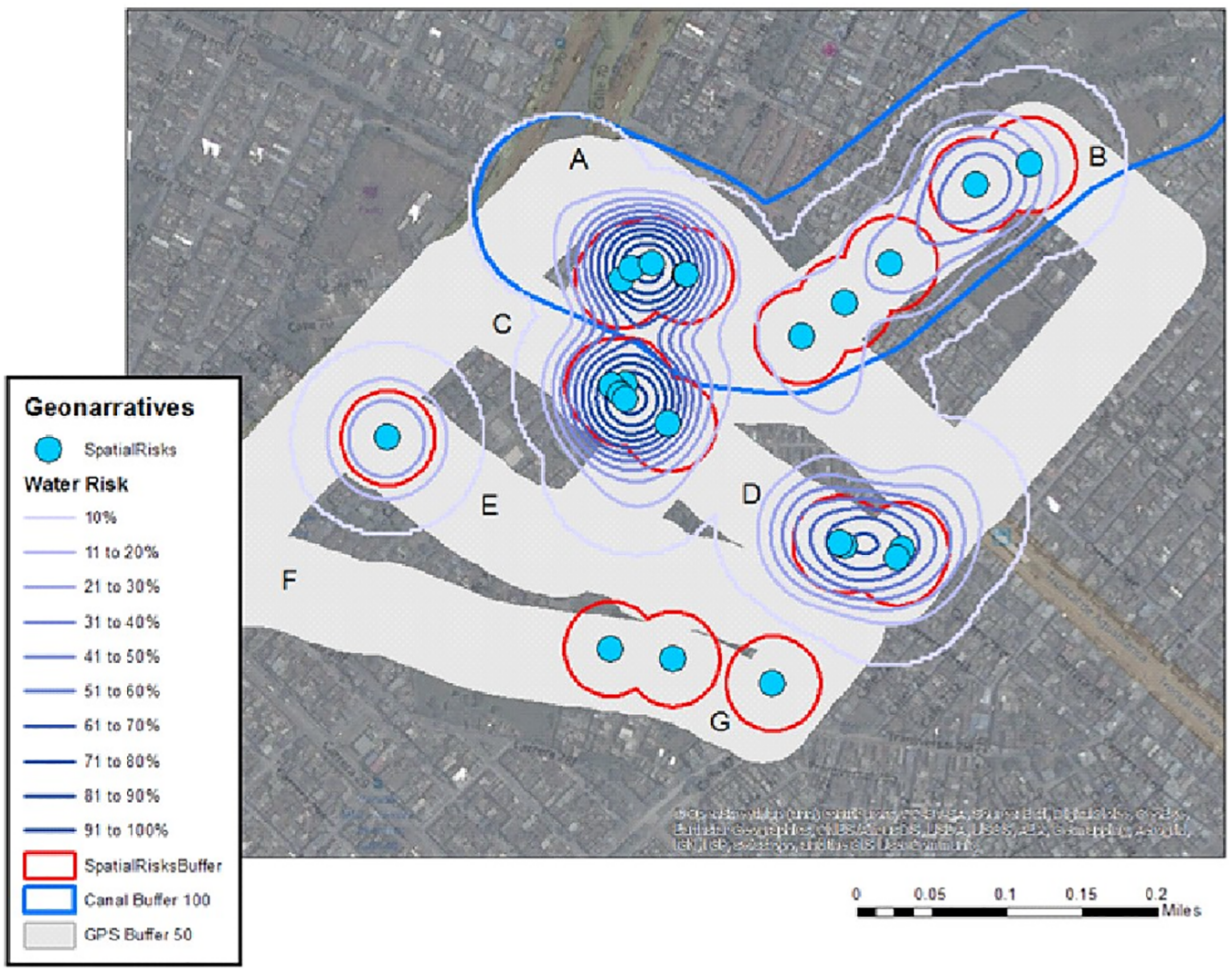
Mapping of the Calipso neighborhood. KDE of spatial risk classified into ten equal categories and contoured.

Fig 7 displays these risk contours along with the SVG path which has been buffered to 50 m, and can be used as a proxy for the denominator geography investigated in the neighborhood; anything of (visual) note occurring along this path would hopefully have been recorded in the comments. Spatial risk comments identified from the SVG are also displayed, again with a 50 m buffer (in red). Seven labels (A to G) have been added so that comparisons can be made between the graphics. One key geographic feature, a drainage canal, is also identified and buffered to 100 m. In the narrative, the potential impact this canal has on the rest of the neighborhood was described as far larger than 100m. We conservatively keep the risk distance to be in proportion to the other identified risks. There is some overlap between the canal buffer and the spatial risk locations because specific features in and around the canal are identified, such as engineering work, bridges, and stagnant water sections, all of which posed an elevated risk along the feature. The buffered canal, key spatial comments and the contoured “water” keywords overlap around areas A to C. A further common area between the key comments and contours is found at D. The remaining key mentions (E and G) are obviously not water-specific.

**Fig 8:**
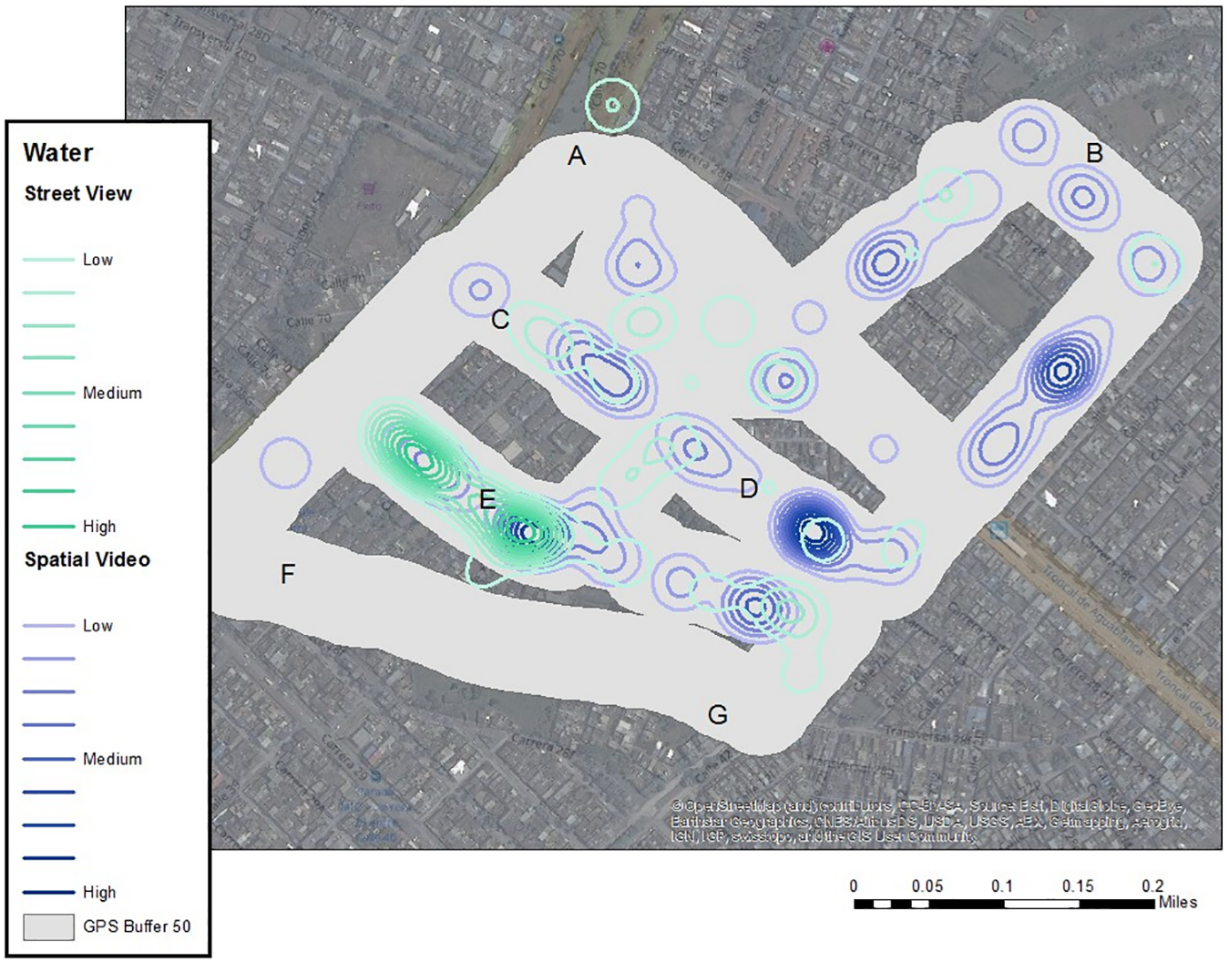
Google street view versus spatial video. KDE of water classified into ten equal categories and contoured.

The SV was also used as a data source for the digitizing of risks, including standing water, trash accumulations, dogs, and other variables with potential connection to dengue, such as vegetation and evidence of poverty. Each risk location (for example, standing water) was digitized as points to show the spatial extent, while a weight was added to approximate the visual depth. While obviously open to user coding error, previous use of this method has proven successful [97], especially if the same coder is used which eliminates the problem of inter-rater reliability. The same approach was also used for Google Street View (GSV) coverage of the area. This resulted in 93 digitized water locations for SV and 99 for GSV, and 110 trash or tire locations for SV and 80 for GSV (Fig 8). Of concern with the GSV coverage was the number of image date stamps (7/2013, 10/2013, 1/2014, 9/2014, 1/2015, 12/2015, 4/2016).

**Fig 9:**
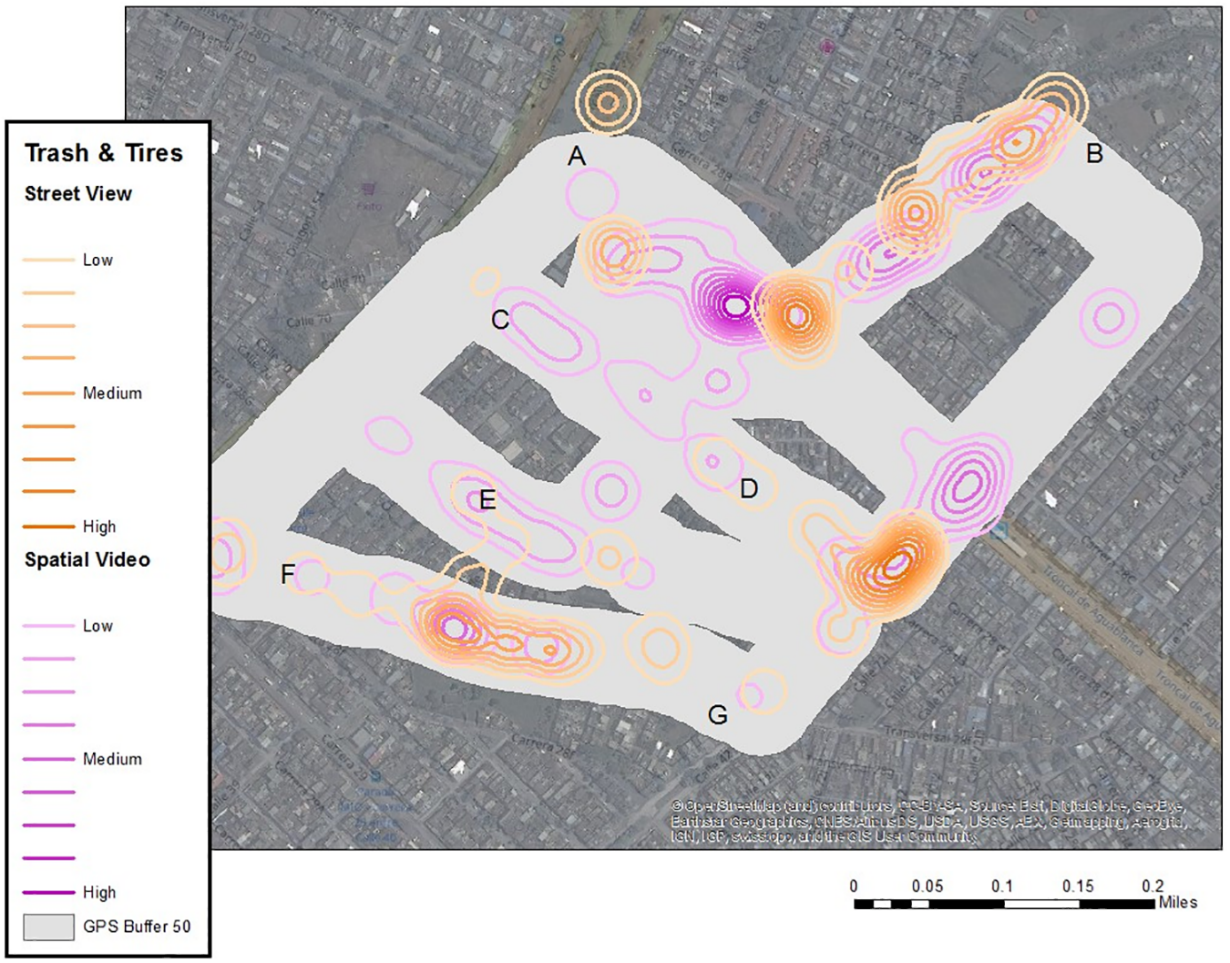
Kernel density analysis of trash. KDE of trash and tires classified into ten equal categories and contoured.

Each digitized point was used as input into a KDE with a 50 m kernel, weighted by the depth or volume of the risk. Each KDE was classified into ten equal categories and contoured (Fig 8 through Fig 9; the buffered SV path is still included for comparison purposes). In Fig 8, the best overlap for both the GSV and SV sources was area E. While there is also some similarity with areas C and D, SV dominates in intensity. This pattern is also true for the general section of the map between B and D. While the area between A and B is proximate to the canal, there is not a lot of visible standing water outside of the channel. When comparing Fig 8 to Fig 9, areas A through D exhibit similarity between SVG and the two digitized sources of data, while interestingly the most intense visible section (E) for both GSV and SV is not reflected in the comments of the SVG.

There are similarities between the GSV and SV sources in terms of visible tires and trash, with the path between A and B being similar. This is also true for the path between F and G, and the area to the southwest of D. Area C to D and especially E which had high levels of visible standing water have lower levels of visible trash. If this map is compared with Fig 9, there is consistency between spatial mentions involving trash along the path leading to B (from A), between F and G, and the corner between D and G.

## Discussion

There is a rich tradition of spatial research on mosquito-vectored diseases, and especially dengue. Cali, Colombia is a large urban area with hypoendemic dengue transmission with recent outbreaks of Zika and chikungunya. Several studies have focused on the dengue, Zika and chikungunya situation in Colombia [11–21], ranging from disease patterns to the identification of fine scale environmental and social risks [29, 31–33]. Even in this well-studied urban environment, there is still a lack of sub-neighborhood scale investigations enriched with local context, which arguably is what is needed to design effective control interventions. We have addressed this deficiency using expert insight captured with SVG for various neighborhoods of the city. In addition, we have mapped, compared and contrasted risk features digitized from two similar data sources: SV and GSV.

SVG revealed both spatially specific and general observations regarding the status of disease in the city. While key locations such as unfinished roads with puddles, proximity to the canal, and the presence of trash are expected, it is the detail around each which makes SVG such a valuable tool. For example, risk in proximity to the canal does not decrease in a steady decay function, but rather the local conditions in and around have dramatic impacts. Proximity to informal settlements increases the amount of trash, while building works can also cause the water to stagnate. Smaller open drainage channels, which are a risk in themselves, sometimes border childhood playgrounds. Added to these risks are the actions of people in the various communities, some of whom are perceived to be unconcerned (or not educated) to the risks posed by adding to surface water and discarding trash which can provide breeding sites. The security of each neighborhood also plays a role, in limiting fumigation, trash collection (and other services), and access to health care services.

SVG can also be used to develop systematic risk mapping for a neighborhood by identifying key locations of risk, or by analyzing all words describing a similar risk. For one neighborhood with suspiciously low disease surveillance, SVG maps were compared to risks digitized from either GSV or SV as a data source. While there were some variations between the three sources, there were also areas of commonality, suggesting perceived and visual robustness in risk presence. For example, when combining all sources, the most likely areas of vulnerability include the path from A to B (water and trash); the path from C to D (water and trash); water risks around E; and trash between F and G.

**Fig 10:**
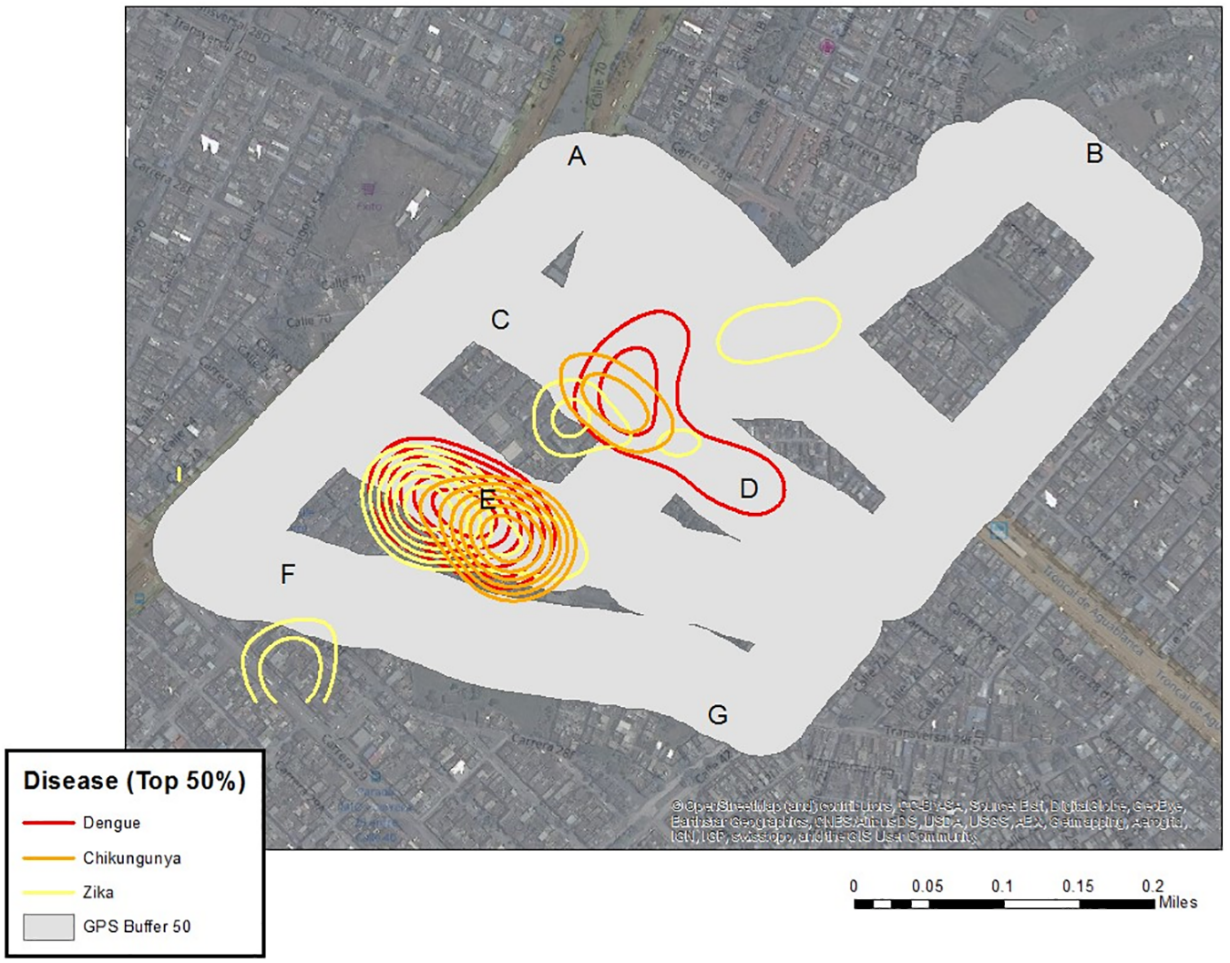
Risk maps. Top 50% Contours of Risk by Disease. Surveillance data for dengue, chikungunya, and Zika are overlaid on the path buffer. Dengue October 2014—April 2016. Zika November 2015—April 2016. Chikungunya January 2015—April 2016.

The next step is to overlay surveillance data for comparison of dengue, chikungunya, and Zika (Fig 10). A similar mapping procedure as for the previous risks was applied (a KDE with a kernel of 100 m, classified into ten equal categories and then contoured). Two immediate patterns emerged from Fig 10, the high intensity around E and between C and D. It is also interesting to note that all three diseases exhibited markedly similar patterns, which is not surprising considering the same vector is involved. If this map based on disease cases had been targeted for use, the two respective areas would certainly suggest priorities for intervention. However, there would be little insight as to what might be causing these “hotspots”, and therefore how to design an intervention or education strategy.

If we again consider Fig 8 through Fig 9, point “E” generated the highest and most consistent (between GSV and SV) water-related risk, with some visible trash (especially using SV). The path between C and D emerges as both spatial mentions of risk, and water-related comments in Fig 8, and digitized water and trash risks for both GSV and SV sources, though SV dominates. Therefore, using these risk sources, it is suggested that potential causes for the disease hotspots are neighborhood conditions resulting in visible standing water and trash accumulation.

**Fig 11:**
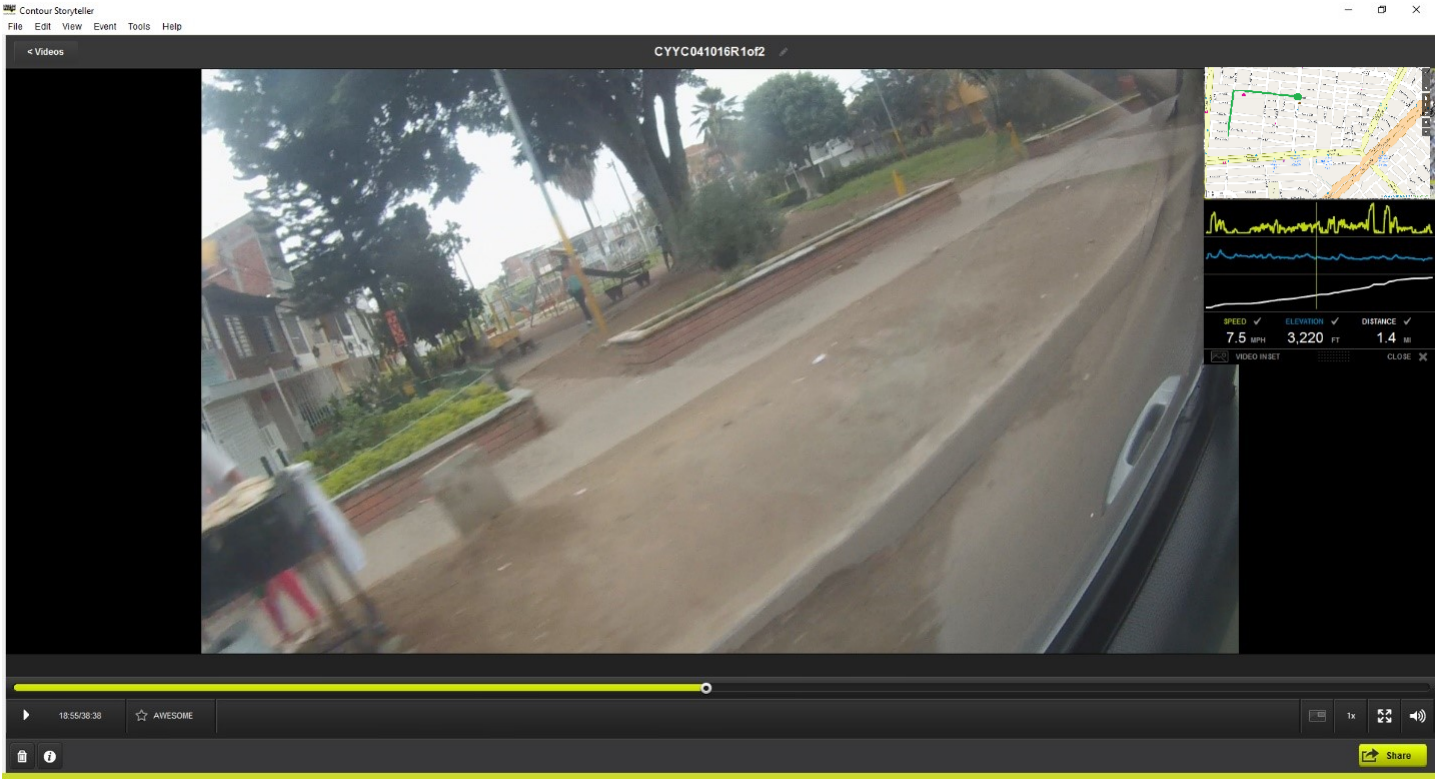
Point E. Small pocket parks with playsets overlaps point “E”.

An additional benefit of the SV and SVG approach is that the source material (transcribed comments and video) can be referred to for information, i.e., the area around E is a commercial strip with several stores. The road also has small pocket parks with playsets (Fig 11, the location of this park is where “E” is on the previous Figs). Visible side roads (which were not driven) are densely packed with houses. Although there are sections of broken road with standing water puddles, most of the roads are well maintained. The quality of the buildings and road surface deteriorates closer to the canal. As few of the dense residential streets were covered on the SVG drive, the next step would be to return to these areas and cover them on foot or bicycle to gain an even more granular perspective of the neighborhood.

The maps also warrant further investigation. First, why were some of the highest risk areas identified through the three sources (A to B) not present in the disease risk maps even though this is a dense urban area? While possible explanations exist regarding the lack of surveillance data for people living under the most impoverished economic conditions, SVG reveals that “poverty” often increases closer to the canal edge. This could be due to the fact that the risks identified around the canal impact a far wider area easily extending to C, D and E because of the flight range of the mosquito, though one would still expect more cases closer to the source.

In another data inconsistency, given the importance of E in terms of visible water risks, and disease occurrence, why was this section not commented on during the SVG? Again, the benefit of the SV and SVG approach is that the source data can be referred to and reexamined for this exact segment of data collection. When one returns to the spatial comments, the sole mention closest to E does in fact state, “Look at the stagnant water in the (channel).” As more SVG are collected for the neighborhood, more confidence can be placed on the locations described as reflecting the community perspective and not just a single ride.

Another research question raised in this paper was, is Google Street View a suitable data source, especially if no SV is available? While there is consistency in output, especially for the disease hotspot of E, the number of time periods included in GSV is an obvious concern. In additional work not reported here, we have compared how environments have changed in Cali between the GSV and SV dates, and for some neighborhoods there is considerable variation. Therefore, GSV is a reasonable source of insight if the urban area has not undergone considerable change and if one acknowledges the variations in time frame. However, for many disease-threatened areas, GSV is not available, and this, along with being able to dictate when video data are collected, still suggests SV is the better source.

## Conclusions

This study has revealed the types of risks present at a geographic scale not commonly analyzed with regards to dengue or other mosquito-borne diseases. The combined SV and SVG approach not only can be used to identify potential risks, but also, when overlaid with actual disease cases, can be used as an ongoing data resource to determine what was happening inside each hotspot, what it looks like, and how it is described. This detail is invaluable when targeting limited intervention resources. Given the insights revealed from the SV and SVG, improvements in drainage and road surface quality might have an immediate impact on disease reduction. However, as only the main (commercial) roads were driven, a return visit might include all residential streets to assess the conditions closer to where people live. People in the general vicinity should also be educated as to the real disease risk and encouraged to use window screens, mosquito repellant or at least wear protective clothing while outside.

The approach presented here should also be considered as only a first step. It provides a baseline that can be supplemented or modified as seasons change or after any intervention. By collecting further SVG, more depth can also be added to the neighborhood, and at the same time incorporate different perspectives. As such, this approach not only bridges the gap between researchers and practitioners, but it can also incorporate community participation in the process. Community insight is not only valuable in terms of explaining why risk occurs, but also can provide an understanding of local perceptions, beliefs and practices that influence health promotion and education. Why do so few houses use screens? Why is there no local desire to control trash even around the home? Why do men work without shirts even though mosquitoes are present? These are the types of questions that can be investigated through SVG, leading to more culturally appropriate intervention strategies.

## Supporting information

S1 Data Files 1, 2, and 3: Case data aggregated to neighborhood level with neighborhood characteristics. All cases were mapped to the neighborhood level. **Folder A. Shape files.** Shapefiles were used to define the neighborhoods. **Folder B. Geodatabase of shapefiles.** Neighborhood characteristic. 10.6084/m9.figshare.5172508.
(ZIP)

S1 Text Spatial video geonnarative transcripts (English, De-identified). All SVG interviews were transcribed in Spanish and translated from Spanish to English by Fluent and Native speakers. 10.6084/m9.figshare.5172502.
(DOCX)

S2 Text SVG interviewee prompts. SVG interviews were semi-structured and led by the local expert or leader. These questions were used to prompt leaders to point risks related to arboviral transmission in the neighborhood. 10.6084/m9.figshare.5197315.
(DOCX)

S3 Text SVG interview footnotes. All SVG interviews were transcribed and translated. We used examples taken from these expert narratives throughout the paper to illustrate the various points being raised. CHW = community health worker, VC = vector control specialist. 10.6084/m9.figshare.5172970.
(DOCX)
