# Supplementary material for: Community context and sub-neighborhood scale detail to explain dengue, chikungunya and Zika patterns in Cali, Colombia
Source: PLoS One. 2017 Aug 2;12(8):e0181208. doi: 10.1371/journal.pone.0181208 (PMC5540594; doi:10.1371/journal.pone.0181208)
Supplement: S1 Text — All SVG interviews were transcribed in Spanish and translated from Spanish to English by Fluent and Native speakers. 10.6084/m9.figshare.5172502. (DOCX) [file pone.0181208.s002.docx]

ZOOM0002.Wav

Neighborhood: Calipso and Yira Castro

Date: April 10-16 2016

[00:00:07] A: We are in Calipso and Yira Castro.

[00:01:00]

[00:02:00]

[00:02:56] D: This is Calypso’s canal.

[00:02:59] D: They were fixing it.

[00:03:01] D: They put some sheets on it and they want to know if the water is running or is

stagnant.

[00:03:07] D: Look how the canal is. It is completely stagnant.

[00:03:13] D: You know why? Because of the construction more ahead.

[00:03:17] D: And look at the larvae how you can see them there.

[00:03:21] A: All that movement is larva?

[00:03:23] L: Yes.

[00:03:33] D: Yes, look at how they are moving in the water. Is because they are fixing

something more ahead and the machine is inside in the middle

[00:03:43] A: But for a lot of months?

[00:03:45] D: Yes.

[00:03:45] A: Is it like that for a lot of time?

[00:03:47] D: Yes because the other time we came those borders in the wall weren&#39;t there.

They were making them, of the canal.

[00:03:55] D: It was in concrete and it had those green costal up there.

[00:04:00] D: And they made that block there. And they are making those blocks but more

ahead then the machines

[00:04:07] D: are across the canal. And since they are crossing it and have materials in the back

that

[00:04:12] D: Does not let the water run, it becomes stagnant.

[00:04:17] D: But look at the amount of larva that you see there.

[00:04:30] A: That is a very important hot spot.

[00:05:09] D: That is why this is the worse hot spots in the map.

[00:05:44] D: Look on the other side they are fixing something so the water has become

stagnant.

[00:05:49] D: And that bellow that bridge has to be a breeding site. Big. And from here to there

must be too.

[00:05:56] D: And here bellow that bridge filled with streets, all of them there have the risks of

getting one of these diseases.

[00:06:03] D: But yes, all of those are larvae.

[00:06:11] D: All of that is larva movement.

[00:06:23] A: But they are not biting me here. And we are in a time where they should be

biting, no?

[00:06:31] D: The hour, for me, of the mosquito is in the morning and the night.

[00:06:35] A: Yes.

[00:06:35] D: At six of the afternoon.

[00:06:38] A: But also, those mosquitoes go to the houses. They do not stay here, they search

in houses too.

[00:06:45] L: Where there is more density of people, no?

[00:06:49] A: Yes. They like houses more.

[00:06:54] D: This is Calypso’s most dangerous hot spot in my opinion.

[00:07:12] L: At least there is one mosquito that flew by.

[00:08:00]

[00:09:00]

[00:09:59] D: Look this is a new street; it was not here when we passed last time.

[00:10:02] A: No?

[00:10:03] D: All that filth you see is from the strainers of rainwater. Look, that are (()).

[00:10:12] D: That wasn&#39;t here. And you could see it back there too.

[00:10:18] D: They are brand new.

[00:10:22] D: Then since water became stagnant they made them again.

[00:10:30] D: This street the other day, you see how the asphalt is really black, this street was

not here. It was all uncovered.

[00:10:38] D: You see the strainers that are new, they have not came to clean the filth that

was left.

[00:11:38] D: In this side of Calipso is where they are fixing the canal, towards there.

[00:11:44] D: The mosquito reaches this entire kilometer, to move from there to here. Some

should come from there too.

[00:11:52] D: Because further there the water is more stagnant.

[00:13:07]

[00:13:16] D: Look at the stagnant water there, look.

[00:13:20] A: In the park with all the kids.

[00:13:23] L: In the parks is where you see more tires and waters.

[00:13:30] A: There is movement in that water from larvae.

[00:14:00]

[00:14:20] D: A look at the water how is stagnant here in the street.

[00:14:22] A:  A lot.

[00:14:24] D: That is not from now or that they washed. That is rainwater.

[00:14:35] D: Since it is raining season in this ugly streets water gets stuck there more.

[00:15:00]

[00:16:00]

[00:16:20] D: There is a lot of stagnant water, look.

[00:16:24] D: Stagnant water everywhere.

[00:16:42] A: There is stagnant water in that passage way too.

[00:16:47] D: Those small canals that they use as a drain, there&#39;s stagnant water in the sides.

[00:17:00]

[00:17:15] D: There&#39;s a lot of stagnant water, look, on the sides of the street.

[00:18:00]

[00:19:00]

[00:19:30] D: Look at the stagnant water in the (()).

[00:19:34] A: A lot of water.

[00:20:00]

[00:21:00]

[00:22:00]

[00:23:00]

[00:23:23] D: A lot of mountains like these...helps.

[00:23:26] A: A lot of mountains. A lot.

[00:23:31] A: It likes being there. Waiting.

[00:23:34] D: There is a lot of tires lying there.

[00:24:00]

[00:24:30] D: A lot with a park of tires.

[00:24:50] D: This is the big lot I told you about.

[00:24:53] A: Ah yes with the ((energy)) correct? I remember.

[00:25:03] A: The mosquito park.

[00:26:00]

[00:27:00]

[00:28:00]

[00:28:35] D: Big streets like this are stagnant.

[00:28:40] A: At least it is not rising and going out.

[00:30:00]

[00:31:00]

[00:32:00]

[00:32:49] D: Look at the canal. I told you they were fixing it and it is stagnant, look.

[00:33:00]

[00:33:15] D: Look how it is there. And here it is bigger.

[00:33:20] A: And filled with filth.

[00:33:24] D: And look at the machine there. That is why the one here is fixing things and

making the water stand still.

[00:34:00]

[00:34:03] D: Look at the stagnant water in the streets too.

[00:34:39] D: This is the machine that works in the canal. A (()).

[00:35:00]

[00:35:46] A: It smells really bad.

[00:35:49] L There are smells here.

[00:35:52] D: Since they are constructing the rainwater canal, look they are barely over there.

See how you can see the concrete blocks.

[00:35:59] D:  In the others, the machine needs to get in and get the filth out and scrapes the

side to leave the mold where

[00:36:06] D: where the construction is going to be. Then by doing this, the water gets more

stagnant.

[00:37:00]

[00:38:00]

[00:38:07] D: Look how the water is there.

[00:38:09] L: Uh, look at that.

[00:39:00]

[00:40:00]

[00:41:00]

[00:42:00]

Neighborhood: calipso y yira castro

Interviewer: Amy

Community health worker: Luz Angela Peralta Valencia

Community leaders:

Secretary of health: maribel

Date: April 14 2016

[00:00:03] S1: They taught us that they take alcohol—a flash of alcohol.

[00:00:10] S1: And they buy Thiamine. Thiamine is an— Is an medicine that works for the

bones.

[00:00:17] Amy: Yes.

[00:00:18] S1: Is for decreasing inflammation and strengthens bones. They send that to—All of

the adults.

[00:00:25] S1: Yes, if that isn’t—that is like calcium in the elderly. If not it is that is very

cheap, they are small bags of ten pills, they are about a thousand, eight hundred pesos.

[00:00:41] S1: Then, what do people do over there.

[00:00:43] S1: They take the ten pills and they crush them. They crush them and put them in

alcohol.

[00:00:51] S1: Then they diluted it there. Then at night, they say that they apply it at night.

[00:00:57] S1: And with that, they say the mosquito doesn’t get close.

[00:01:05] Amy:  Ah yes. Thiamine is the pill I was going to take.

[00:01:09] S1: Yes. Then, they take that and apply it, and now we have copied them. And we

do that too here.

[00:01:19] Amy:  And it works.

[00:01:20] S1: And it works.

[00:01:21] S1: That shoos them—shoos the mosquitoes.

[00:01:26] S2: Why do they dilute it with alcohol?

[00:01:29] S1: Well they say, I think, because the alcohol is a disinfectant.

[00:01:40] S1: Since it is a disinfectant, the alcohol, and then they suddenly say that with that

they clean so that the thiamine conserves with the alcohol.

[00:01:53] S1: Then people do it and it works.

[00:01:56] Amy:  Interesting, but they don’t drink it.

[00:02:00] S1: And they take it too, but not the pill alone. Ah, we ((Maintain)) with thiamine

and …

[00:02:05] S2: What’s the connection that you guys have as community leaders eh—in relation

with the management of Dengue, Zika and Chikungunya with the health center?

[00:02:15] S2: You think that the health center is efficient or that it still needs…

[00:02:19] S1: Look, us here, ((imagine)) that we as community leaders do not get close to the

health center, the one from there.

[00:02:30] S1:  Because they don’t let you-- - Nothing against them. But, they always tell us

every time that we ask for collaboration, because what happens. Even thought that this is a

extract three—extract three.

[00:02:49] S1: We, sometimes, there’s people like humble, that I’m saying, too humble—too

humble, but too humble.

[00:03:00] S1: Sometimes—and they suddenly have, they have servants, but suddenly many

times there’s people that do not even have for a consultation. To pay for a consultation.

[00:03:16] S1:  Which is what we say, and that Comfandi has helped us without interest.

[00:03:23] Amy:  Yes.

[00:03:24] S1:  Because everything needs to be said. Without interest, Comfandi has helped

community leaders a lot.

[00:03:31] S1:  More than anything in this sector. Calipso is divided in five sectors, but three

community assemblies. The one from one, from two, from three, from four and from five. The

one from three works with the—with those three sectors.

[00:03:51] S1:  That is very good that the interview is with _______, because ______ leads the

sector close to Rubis. Right?

[00:04:00] S1:  And that part over there, there’s an alley, where that alley is uncovered and

they throw a lot of trash, which is that part behind the apartments over there.

[00:04:10] S1:  Then, over there you see a lot of problems, then what happens to the health

center. We went there, to ask for a collaboration, but the only thing they tell us is: “ Ah, no.

You guys have to go…”

[00:04:27] S2:  To the Hospital.

[00:04:28] S1:  Hospital. You need to go.

[00:04:29] S2:  To the S.

[00:04:29] S1: You have to go there and there—They will give you guys what you need.

[00:04:38] S1:  We here don’t do that, if not only consultations and care for the SISBEN people.

And we told them, why is it that you guys are inside this neighborhood of us, and you need to

collaborate with us because this is public health.

[00:04:55] S2:  What were you asking for?

[00:04:57] S1: That they serve people. There are a lot of people. Before, there was a

community restaurant and the—that restaurant was for everyone, for Calipso, for Robles,

Comunero, Yira, people came her from all of the surrounding to there in the communal

headquarters.

[00:05:19] S1:  They collected that and took it away, Then the elderly that do not have the

capacity and more than anything the money, could have the Sisben and do not understand. But

they do not have enough for a consultation, as small as it is, o transportation then they died

because do not have anyone that can collaborate with them.

[00:05:43] S1:  When we noticed, then we with _______, _______ in person, is to better say it

good.

[00:05:53] S1: Then, what happened? What happens? He in his car, we transport people when

we noticed, but there’s other occasions, that there’s no—we do not notice. Then what

happens?

[00:06:10] S1:  Because there’s no one there capable of tending to them. Instead, sometimes

Comfandi, we go to the people. They tend to them and look what happens (( )). They are from

here, from the neighborhood and they know them in Comfandi.

[00:06:28] S1:  They have a committee here in the sector, but are indifferent to us, the

leaders. They are different people. And we have talked to them so they can tell them.

[00:06:44] S1:  That like them, like public health, like a health center has, is that they have

the obligation of tending to people even if they do not have Sisben, even if they do, well,

another , ((epeseto)) but they are urgencies.

[00:07:01] S1:  Where people don’t have money, so they can comply. Then, that is what we

don’t want. Nobody wants that health center it here.

[00:07:12] S1:  Look, ask what people go there. The people around here don’t go there.

[00:07:27] S1:  And then they—No one goes to that health center.

[00:07:32] S2:  And with what relation to education that the center provides groups the

subjects of Zika, Chikungunya and Dengue.

[00:07:39] S1:  Comfandi has given it better. They could have them, but since people do not go

there because is indifferent to them.

[00:07:45] S2:  They don’t do campaigns.

[00:07:46] S1:  No. We haven’t seen it, we haven’t noticed. Here, the only one is Comfandi

and is one of the different P S.

[00:07:58] Amy:  And this year—are people here cleaning the siphons, for example the

secretary?

[00:08:06] S1: Yes. We don’t have to tell them. They come and fumigate too. What we told

them, we went these days and we told them, that because they only pass on the streets, and

we told asked them why they couldn’t come in.

[00:08:26] S1:  We have a entrance and that a car fits through and why we have that—we did it

like that because the car can come until the corner and fumigates here and fumigates there,

and on the other corner fumigates there and fumigates—it goes into the sectors.

[00:08:47] S2:  It is to say, the car that fumigates and does not goes it the main streets but

they do not go into the neighborhoods.

[00:08:48] S1: They don’t come in, that, the mains, they do not come in the sector.

[00:08:56] S1: And we have the streets, but for example here, the fire truck can come inside in

an accident, the ambulances.

[00:09:08] S1:  Then, why can’t the car? Then, that’s what we told them. Because is

impressive, imagine what they in fumigation is worth, because when they fumigate things calm

down.

[00:09:22] S1: Imagine, and fumigating and coming in and fumigating. There are a lot of green

areas, as you see in quantity.  And the mosquitoes settle in the green zone. Then, if they come

in, and fumigate the trees and the green zone, then it would be very good. Then we told the

other one but she says that we are going to send and report to public health.

[00:09:46] S1: I tell you, but it is to fast. Because in this moments we are going though

problems. And you already told us.

[00:09:56] S1:  The canal is over there, on top of it there’s mosquitoes, its impressive.

[00:10:00] Amy: Yes, it looks like it is raining, ((with the larvae there.))

[00:10:04] S1:  Yes, it is impressive. And you—And it starts at six of the afternoon, during the

day, if you go lay for a while it will bite you.

[00:10:17] S1: Over here they use the fan a lot. They use the fan. Moreover, they have fans

over there.

[00:10:24] S1:  Because it is impressive, the fan. It ventilates and if you saw the quantity that

is left dead in the floor.

[00:10:37] S1: The mosquitoes. And if you would imagine—one fumigates because we use that

one, the spirals, that are the cheapest. They are about a thousand pesos and bring about five

of them.

[00:10:52] Amy:  Yes.

[00:10:54] S1:  Then, people here use one, but people over there divide it, and that that we

took some and gifted them. Then they put then there but they are bad for the kids.

[00:11:06] S1:  Then is one versus another one. There are a lot of things one does, that can be

done, but people sometimes don’t do them.

[00:11:16] S1:  And it is not because they do not have but because they do not want. Because,

see, everything we have talked about and people keep coming up with problems.

[00:11:30] S2:  Why is it that they don’t—What would be needed for the community to act upon

it? You that have managed the community, what do you think happens to those people,

independently that they don’t want to, if not why they don’t want to? Why don’t they assume

the responsibility of their health.

[00:11:58] S1:  No, because for them there’s better things than that. Surviving.

[00:12:02] S2:  Yes

[00:12:05] S1:  Do you think I will stop giving food or that I will stop eating to buy medicine.

With a thousand pesos that a acetaminophen cost, I eat breakfast or lunch. I go to the (( )) I

got and I buy viscera that cost five hundred dollars and I make some broth. That’s what you tell

you.

[00:12:27] S1: I am going to buy some acetaminophen that I know won’t work. That’s what

they tell you, that they do not work.

[00:12:38] S1:  I tell them that they do not work in the moment, but if you keep taking them

they work. Then they, you told me—then this are what happen.

[00:12:46] S1: They are people that think very differently to one. I have training because I am

a professional nurse, an accountant assistant and a professor, I am a scholastic.

[00:13:00] And—But, there’s others and here, in Calipso you find a lot of people like you also

find, like I tell you about ________. She lives in three and three is a good neighborhood. But

she lives like—She lives very humble.

[00:13:22] S1: Very humble. She—Her husband works in a ((taxi)), but a lot of times he doesn’t

have a job. And she is a seamstress and she goes and searches but she likes it too—As she

suffers and sees the necessities, she goes around and everything.

[00:13:44] S1:  And she—She has a position, she got named like a representative like the one

from, she is like from Cauco. Then, she has a lot of contacts.

[00:13:59] S1:   She is a key for you guys because if we go there, she lives with them.

[00:14:07] S1:  In that elections, if you would see, how the people are with her. She gathers up

people impressively, humble people. We support her a lot. She tells us where, and she really

knows all of the neighborhoods. She tells us which neighborhood and what there’s lack of.

[00:14:34] S1:  Then I tell her, okay and we go. Then, we organize everything and go. But she is

a leader, to a community level.

[00:14:47] S1:  Moreover she is also from the neighborhood.

[00:14:55] S1: Then, that is a good one—And then we have fought against that a lot. Against

that, better said we have even made politics.

[00:15:11] S1:  So they can listen to us in public health and they make a standstill to the health

center. The put a lot of money in that and go see and look for the small amount of people that

they keep there. Did you see?

[00:15:32] Amy:   Yes, is that I…

[00:15:33] S1:  And people are up since five in the morning and you go by and there are three

and four people. And if you would see how pretty that they—All the pretty things they did.

[00:15:45] And it doesn’t work for anything. Because there—It happened to me the other day.

[00:16:20] S2:  And do you know of the cases that they have not tended to Dengue or Zika

patients in the health center.

[00:16:26] S1:  Oh no, of Dengue and Zika no. I tell you—people do not go there. One tells

them. And that machine inside of the, look—this is what it is.

[00:16:36] Amy: Yes, that. Patchouli also works, is an aromatics and it works very well and

doesn’t harms the kids.

[00:16:54] S2: Is it a herb?

[00:16:57] Amy:  It is an herb, and they make aromatics with that.

[00:16:59] S1:  That’s very good.

[00:17:02] S2:  And where do you find it?

[00:17:04] Amy:   In any market with natural things

[00:17:07] S1:  Ah yes.

[00:17:08] Amy:  Or with incense.

[00:17:11] S1:  Incense, ah.

[00:17:15] Amy:  Ask if the have patchouli.

[00:17:18] S1: No, people do not go. And then the people who copy them take over and there

are people who go there. But the people who need it, do not go because one goes and they tell

you what identification they have. There was a case with a neighbor.

[00:17:49] S1:  She went the other day and on these days she go the Chikungunya. And she

went there. They asked her for identification and she told them she did not have it but she was

feeling bad and might have Chikungunya please help everything and me. And they told her no.

[00:18:15] S2: It is to say that this system does not…

[00:18:16] S1:  No, they are not helping. Not helping.

[00:18:21] Amy:  And where do they send her?

[00:18:21] S1:  No go Carlos Holmes, they will tend to her in emergencies.

[00:18:26] Amy:  The emergency room is there?

[00:18:26] S1: One is sick, and one can go walking. When one is good, but is not one goes to

Carlos Holmes.

[00:18:45] S1:  She came and protested and said that how was she supposed to go down to the

health center. And because I saw her—She asked me whether it was Zika or Chikungunya,

because we see the—I mean the difference.

[00:19:02] S1:  The she said, &quot; What do you think with this fever”. Because I was there with

her, and I told her that we give her the mango and she did it—I mean it calmed down and I gave

her acetaminophen and she started taking it and it stopped her fever and some symptoms.

[00:19:30] S1:  But as same as her she went and they told her no, if you do not have

identification the consultation is like eight, nine, twelve thousand.

[00:19:43] S1:  Then she asked him what happened, I don’t have money, not even for the bus. I

don’t even have money for if I have a Sisben. They just told her they could not tend to her.

[00:19:55] S1:  They were not even capable of telling to go home and take acetaminophen

every four hours. They did not even tell her that.

[00:20:04] S1:  Then she—They do not work for nothing,_____. That is because you do not

speak. Look, when we are in election times, we tell that to the political figures that they fix

our problems with that or to take it out of here. How those that help us? And put a better

school there that we do not have.

[00:20:24] S1:  The closest public school that closest is—of S E P A S. That one, how was it?

[00:20:32] Amy:  Of S E P A S from Cali.

[00:20:31] S1: That is the new one. That is the closest one, but the crime rate is impressive.

Then how will one risk our kids, for example my son, he has fourteen years, a kid I have raised

well and to take him there and for him to mix in with those—who do not have the same

education.

[00:20:57] S1: Then I need to have him her in a private school, that is close by and very good

and has coverage. They wanted to take it away but with a political figure that we did politics

for, he helped.

[00:21:13] S1:  It is a good school.

[00:21:14] S2:  It is to say that for you guys to—get organize you have to go to politics.

[00:21:23] S1:  Yes.

[00:21:23] S2:  Because if not, there’s no other way.

[00:21:26] S1:  Another option for nothing.

[00:21:28] S2:  What organization your community has that fight…

[00:21:31] S1: For them to fight for—no. Us, you see, we fought like committees for projects,

like fixing courts, green areas, you see? But we have never stopped with the health center.

Better said, no one wants that health center.

[00:21:50] S2: For example, do you have something to do for the canal that you say or does

that not exist?

[00:22:00] S1:  We don’t have anywhere to go. This is why this is for me and I will tell everyone

what I did with you. This copy helps me and in these days we do meetings and I will tell them.

Because this is really important, because I know that this way you take it there and they will

pay attention to us.

[00:22:20] S2:  The study she has is for a university in United States, yes? But, what she will

give you is the study. It is true that they do.

[00:22:35] Amy:  Yes, we will send the results to the health secretary and to you through

_____. She is in charge of taking the results to you, the leaders we are working with.

[00:22:50] S1:  Ah perfect.

[00:22:52] S2:  And with hat report, you guys have to ask ___ for it so you can do your meetings

and from there you can do collective creations, because there’s no collective construction.

[00:23:04] S1:  Look, we—Do you know doctor ____? Who is the council president. We work for

him. And he is—that is one of the one we ask for. As leaders of the community we can’t do

politics. But towards people.

[00:23:28] S1:  For what? So they can put a public school nearby. Then, people here say, you

can ask them yourself, that they want that health center removed and that they put a public

school because that health center doesn’t work for us.

[00:23:49] S1:  Comfandi serves more use, which is a private institution. Is a D S ((E P S)).

There—we got information they gave us the recipe. They did not give us the medication, but I

got it. And the acetaminophen and we went to all the people. And that works a lot. Too much.

[00:24:15] S1:  It works a lot because is rigorous through out the neighborhood and everyone

buys and does and you know people when that happens to them, they take that similarly to the

Zika, and that works for it too.

[00:24:28] S1: Because—They told us it was a low defense in the articulations in the muscles

and everything. Then the mango has a substance that raises the defenses and the pepper too.

[00:24:57] S1: Is the one in charge of mending articulation pain.

[00:25:04] Amy:  The inflammation, the pepper is good for that, lime too.

[00:25:011] S1:  And well, that is what we need, thank God, if you would see how Comfandi

fills up. And it maintains that way. You go to that health center and the doctors are relaxed.

[00:25:37] S1: You see them drinking coffee and everything, I say that is lazy.

[00:25:44] S2:  In relation to Chikungunya and Dengue, the case you know is Liliana’s?

[00:25:50] S1:  Yes.

[00:25:51] S2:  Is there more cases where they haven’t serve, or that people that people who

have are close to this sector have died?

[00:26:03] S1: No, they tell us. In example, oh how they (()), that is—that center does not

work. It always—They took someone and they did not want to serve them.

[00:26:00] S2:  Is the attention friendly?

[00:26:23] S1:  Look, I haven’t been there a lot, but people go, I did not go—It has been a while

since I went because I once went, being in the community board because there is a companion

that is also a leader too and is part of the association there, which is in this neighborhood.

There’s from every sector, two people and in our sector those two people are from that board.

[00:26:57] S1:  We have told them, what is all that you do there, you—if that does not work.

You know, I went there once and I remember I had Dengue. I was burning with fever and with a

bad headache with shivers but I did not have any outbreaks or anything. 4

[00:27:20] S1:  My husband lost his job and did not have EPS. Then I went there and they told

me I needed an identification and I told them my husband’s situation—I had Comfandi, but had

since my husband lost his job I don’t. Sign up first they said, and I told them that sure, my

sickness will wait while my husband comes and gets settled, or while I do the SISBEN.

[00:27:48] S1:  And I, being a community leader—You need to understand while we get the

identification. That identification—But they said, you could be the president but if you do not

have the identification. But that is my identification—but no, I will still charge the

consultation. I will tend to you but I will charge you the consultation as a particular.

[00:28:10] S1:  And then how are we here. That’s not how it is. That’s why it is called health

center because you have to—Hi, Lily, come and tell us the experience you had with the health

center when--

[00:28:43] S1:  I told them you had Chikungunya fifteen days ago and you went to the health

center and they said…

[00:28:51] L: They told me the could not tend to me because I had to go to (( )) to church there

is. I told them they always tend to me there. They told me that—I have the SISBEN three and I

had to go to—I drink the pills (( )) and the Loratadine and Acetaminophen . Because that’s what

they tell us, drink liquids, rest and acetaminophen each four hours.

[00:29:25] S2:  How much did the disease last?

[00:29:28] L: Two days.

[00:29:30] S2:  No more?

[00:29:30] S1:  Yes, because she attacked it..

[00:29:32] L: No, I did not break out and she gave me a Loratadine and I took it,

((Matarratón)), what our grandfathers used. (( )) and I cooked it.

[00:29:41] S1:  And the mango?

[00:29:45] S2:  And body aches?

[00:29:46] L: Yes, my articulations hurt and the acetaminophen yes-

[00:29:50] S1:  You got inflammation.

[00:29:52] L: But it only lasted two days and the other day (( )) because it was-

[00:29:56] S2:  And what did you do to know it was Chikungunya and not dengue?

[00:29:59] Unintelligible.

[00:30:03] S2:  The diagnostics?

[00:30:06] S1:  We classified like that because the outbreak comes like I tell you.

[00:30:10] L: Yes, I had it. It started with itching and then an outbreak and that—I had a small

fever. And as I had articulation pain because that day I started like that and the other day I

went to get up to get my kid ready to school. When I went to stand up, I couldn’t because of

the pain in my knee and I fell back into the bed.

[00:30:34] L: Then I told my son to help me stand up and he did but I walked like a robot,

because I could not take care of myself. ((I got )) I couldn’t with the pain. I had to go

around like a robot, stiff.

[00:30:47] L: In the other park there’s a matarratón tree. Well, there with a (( )) and I washed

them and put them in pot and bathed two days with it.

[00:31:01] S1:  This is what we do. She went to the health center because we met. And there

she told them, the world goes by and they tell them because she goes out like that. One goes

to that center and they do not serve you like Comfandi. Not in Comfandi, we go and right

there—they even know you. In change, we are indifferent to them. Then—and knowing that it is

something--

[00:31:50] S2: Do you know people who have suffered Dengue, Chikungunya o Zika in the

sector?

[00:32:00] S1:  In the sector...

[00:32:02] L: Well, _____ mom. What was her name? She has it in this moment. Where she

lived.

[00:32:17] L: __________ also had it. A lot of people have gotten it here.

[00:32:21] S1:  Yes ____ also kind of had it.

[00:32:23] L: Who?

[00:32:23] S1: _________.

[00:32:24] L: A __________ ?

[00:32:24] S1:  The kids.

[00:32:26] L: Ah, the kid. __________ .

[00:32:29] S2:  Do you guys use the tornillo to or?

[00:32:33] L: No, with this heat no one uses them.

[00:32:36] S2:  That is one of the causes to not use that, then.

[00:32:42] L: I know that thing.

[00:32:46] S1:  The spiral.

[00:32:46] L: The thing that connects.

[00:32:49] S1:  Ah yes, she has that...

[00:32:50] Amy:  I used that thing today.

[00:32:52] L: That is the one I know, I turn it on at seven and I put it for when I get (( )).

[00:32:57] Amy:  Yes.

[00:32:58] S1:  Yeah but that—depends on the conditions of—cheap, that is what we use as

_____ said. Us, that’s what we are the for to the Aguas Blancas district . People over there

don’t have how. ((I did not do it, _____ did it))  Do you think I will buy those things while I can

feed my kids with that?

[00:33:20] L: Yes, because daily that costs about three hundred pesos. Right?

[00:33:27] S1:  Well, that is.

[00:33:28]  L: I put it there because y sister brought it when she came from (( )). She brought

me a box. That’s why, when it falls, but when I have the (( )).

[00:33:39] S2:  From your family, has anyone gotten Dengue, Chikungunya o Zika?

[00:33:46] L: No. My daughter in law but she lives over there. She had that disease and she is a

(( )) but she came to Comfandi.

[00:34:03] S2:  Do you guys know how Zika o Chikungunya is transmitted?

[00:34:07] L: Through—When there’s humidity and the water is stagnant.

[00:34:13] S1:  They even get in your mouth, those mosquitoes.

[00:34:17] L: When there’s water in the tires and everything, we have to avoid it? But it is

here; in the living room you can see them, the mosquitoes looks like a crown.

[00:34:30] S2:  There are tires here?

[00:34:34] S1:  No, there used to.

[00:34:38] L: You know what happens, the canal is over there.

[00:34:41] S1:  It is accumulated in the-

[00:34:43] L: Yes, they made a bridge of (( )) and everything (( )) of the river. But that is why

the water does not run something is in the way. Then-

[00:34:55] S1:  Because they are working on it.

[00:34:57] L: Then imagine the people that live behind that canal? The people who live here in

Yira. With houses here and the canals there. For-

[00:35:04] S1:  That is the investigation that ____ will do there. Right close by which is Yira.

[00:35:11] L: Because here is the house and here is the canal. And the water (( )).

[00:35:15] S1:  Yeah, they took pictures.

[00:35:17] Amy: In the canal, people where sitting there and the mosquitoes were biting the

children.

[00:35:23] L: Yes?

[00:35:25] S1:  Then, I told her, but they pay and a lot of us pay the grotto because—And

the—And plants and everything like that, also—plants that one sometimes— In example, the

plants that uncle has her, and I even put them over there because of humidity.

[00:35:46] S2:  What plants are the ones you guys think can help the mosquito transmission?

[00:35:50] S1:  Ah Miami, the aquatic one, Miami.

[00:35:55] S2:  Miami is aquatic.

[00:35:56] S1:  Miami is aquatic. It is an aquatic plant, and is an coiler. Then, she’s from water

and he is from earth. A lot of people had it because it decorates the grotto or aquarium.

[00:36:11] S2:  Or that greenish stick that they use in offices. Another one that is long that you

had (( ))

[00:36:19] S1:  Yes, yes.

[00:36:20] S2:  That they use in the offices, that bamboo. It lives in water.

[00:36:28] S1:  Everyone around here, because it is impressive but a lot of people here,

correct. We sit there in the corner, in a place that sells coffee.

[00:36:43] S1:  They sell coffee, pan de ((hongo)), buñuelo and everything. And we sat there,

and people passed and everyone was with the Chikungunya, and Chikungunya and we laughed.

Oh girl, leave the Chikungunya alone. But a lot of people, correct.

[00:36:58] S2:  In those plants right there, those it accumulates too?

[00:37:01] S1:   Uh yes.

[00:37:02] S2:  And how is that called?

[00:37:04] S1:  That one right there is called…

[00:37:07] S2:  Is that limoncillo? That is...

[00:37:11] S1:  No, no. Those plants, that is—What is the name?

[00:37:15] L: It is called...

[00:37:17] S1: That is used to make siege.

[00:37:20] L: It is a Singla.

[00:37:22] S1:  Yes, Singla. Public health gave us that and we planted it. There’s the

bracero—that one is borrachero. It says it is for—No that is Imam, it is a flower that looks like a

bell. Yes.

[00:37:42] S2:  Ah okay.

[00:37:44] S1:  That is the—What is the name, ______? It’s similar to—from the bamboo family.

[00:38:00] S1:  And the pals are there but we notice a lot in this one. In this one and that one a

lot of mosquitoes accumulate.

[00:38:11] S2:  And with the relation to those rainwaters and those too.

[00:38:19] S1:  That is when—What happens there is that the lid fell off because that is a canal.

But similarly that runs and that’s it.

[00:38:56] S1:  And what they say over there, oh no they come and fumigate and it is only in

the streets and they don’t come in and it would good if they would come in and fumigate the

plants and green zones and avoid that.

[00:39:27] S2:  The important thing is—The importance is their knowledge or formulations or

what you guys (( )) with the situation about Chikungunya, Dengue or Zika. And it for the study

that they are doing in Kent University is very important.

[00:40:19] Amy:  Well we are doing this study with Kent State university in the health network

of (( )), foundation (( aeledu)) and the health secretary. And it is called Chikungunya and

Dengue in Cali. Here you put the place and the address.

[00:40:59]

[00:41:17]

[00:41:28] S2:  What other leaders we can find here that would also be involved with health?

Nurses or involved with that?

[00:41:42] S1:  Well—she is not a nurse.

[00:42:36]

[00:43:42]

[00:44:01] Amy: And here I will put the place, which is Calypso. And the date, which is April

fourteen. And here I will put my name and the name of _____ as a witness. I already explained

the study.

[00:44:24] Amy:  And here is the permission of using your voice recording, then we put your

name here again and identity card _____________ and date....

[00:45:15] Amy:  And yes or no if we can use your voice in the investigation, in presentations

and publications.

[00:45:23] L: Yes.

[00:45:24] Amy:  Yes, ah good. Thanks.

[00:46:02]

[00:46:17]

[00:46:49]

[00:48:02] Amy:  Yes, we were recording videos here on the draught and now rainwater. Then

we want to see how it changed with the rainwater and the Zika policy, because it’s obvious

there’s more social interest by the secretary and they are putting more people to clean. In

example, the siphons and canals, water and everything because that moves people more, no?

[00:48:33] L: But, eh. You tell public health to send for the siphons, correct? But they do not

come by around for a while.

[00:48:41] Amy:  No?

[00:48:42] L: No. It is enough, they came by and put powder in the siphons for mosquitoes but

it has been a while. That they do not come by (( )).

[00:48:55] S2:  In the year, how many more or less?

[00:48:57] L: No, this year—No, this coming year I did not see them come and neither this year.

[00:49:04] S2: You guys ask for the health center or how do you do for making right of petitions

so they come to sanitize the environment.

[00:49:13] L: Well, we thought of talking with the committee so they call and that is why the

DAGMA comes, no? They sent them for—They come from the DAGMA and put plates so they

cannot cut them.

[00:49:43] S2: Yes, they make a tree inventory and the types—But let’s say that you guys see an

augmentation of mosquitoes, what do you guys do?

[00:49:56] L: Well here...

[00:49:58] S2:  Which organizations you go and tell them that you have a problem?

[00:50:06] L: They called the other time, I don’t remember—The car that fumigates but only

thorough main streets. It passes and we feel the smell of the poison and everything. But they

haven’t passed this year. They haven’t fumigated this year.

[00:50:29] S2:  Do you guys have seen that with the rain there’s an increase in mosquitoes?

[00:50:33] L: Yes. A lot.

[00:50:38] S2:  Why does that happen? Why does it increase?

[00:50:42] L: The humidity, I think. Correct? Or because the grass maintains that humidity and

the plants, three and everything, I think that increases the humidity. The canal increases it

too.

[00:50:59] Amy:  That canal is a very critical spot.

[00:51:01] L: I think that the principal spot for the mosquito is in that canal.

[00:51:06] Amy:  And the second one is the garden with that humidity.

[00:51:10] L: Yes.

[00:51:11] Amy: We have to clean the canal, yes? And the gardens we have to cut them or?

[00:51:16] L: Like cutting. Of course here, we here, the neighborhoods and everything, we cut

the Singla there. This is week is when the board, yes the one from the board, the president was

clearing the way. Don’t you see—Yes there’s a part, I know over here, one or another that he

cleared up a bit.

[00:51:48] But that isn’t everything because he is the one that has to speak to the DAGMA, you

need to send a letter to clean everything that is the park, no?

[00:51:59] S2:  Oh, so you know the mosquitoes, correct?

[00:52:01] L: Well I have seen it in TV, black with long legs and the stomach red with blood.

[00:52:12] Amy:  Yes, lady. That’s how it is.

[00:52:15] L: And the symptoms like headache, fever and articulation pain are Chikungunya no?

[00:52:23] Amy:  Yes.

[00:52:23] L: That.

[00:52:25] S2:  What is the difference with the Zika?

[00:52:29] L: With Zika well, they say is very similar to Chikungunya. It starts the outbreak, red

spots like erosions in the skin. Later, the articulation pain and headaches and abdominal pain

start. The colic starts and that ruins the stomach.

[00:52:59] L: But I did not get it like that, I did not know if it is Chikungunya or Zika because I

go erosions all over my body, I got hives and a small headache and articulation pain

articulations. But I did not have abdominal pain or damage. I did not get that.

[00:53:19] L: I have also seen other people who have told me they go stomach pain, headache

and a fever, and I say that is very different, no?

[00:53:29] Amy:  Yes.

[00:53:30] S2:  What other important spots do you know produce Zika.

[00:53:39] L: Other important spots of…

[00:53:40] S2:  If it was from the canal or the plants, do you think they are important spots

where the mosquitoes or larvae are produced.

[00:53:56] S1: Not in the siphons because we have put a net in them.

[00:53:59] L: Yes, but I tell you that they have not sent the powder for the houses that they

used to put in the siphons, no?

[00:54:06] S1:  In the houses, no the sewers, I told them, in the sewers but inside the house

they used to send them and they were really good.

[00:54:15] S2:  And since when do they not do that?

[00:54:18] S1:  A couple years ago.

[00:54:20] L: Yes I told her, this year they have not come to fumigate.

[00:54:24] Amy:  We saw a man doing that in his neighborhood this morning. I asked him why is

he here in the reed beds, here not there where the Dengue is. What is here doing here? Doesn’t

make sense.

[00:54:40] S1:  They used to come and went in the houses and in all the siphons but neither this

nor the last year.

[00:54:49] L: In this week, I saw one in a Poblado Uno in the siphons outside.

[00:54:53] S1:  Not in the siphons, here too but the siphons on the outside. But not in here, and

I told them that day I saw them. Listen, come here, you guys are in the best place but have you

gone in the houses?, No, we are only from the sewers. There are other people that are from

the houses.

[00:55:16] L: Yes, but it has been years that they don’t send them to the houses, no because

before they came with identification and asked permission. And then they looked at the

siphons, took the tanks of the toilets and they out a powder in the sink, everything.

[00:55:30] Amy:  Is there larvae in the tank?

[00:55:36] L: Since that water—Not in the tanks, because that stays clean.

[00:55:38] S1:  No, we got in agreements to have them empty. That’s why they sometimes stop

our water supply. With no moments notice and everything is left without anything. We do not

leave the water stagnant in the tanks. Not at all.

[00:55:56] Amy:  Okay.

[00:55:56] S1:  We have made (( ))

[00:55:59] Amy:  Yes, they have that with very good practices.

[00:56:03] S1:  Yes. Maintain it.

[00:56:04] L: At least in my house there are no plants. There are no plants because that—When

they gift flowers we have them there but we change the water and throw it out. But there are

no vases there.

[00:56:19] S1:  People have raised awareness here.

[00:56:21] S2:  Yes.

[00:56:22] S1:  But the problem is the one over here what happens is what she explained, we

are in high risk. Is where we find most cases, and then I tell here, it is because of that because

there wasn’t any before. That’s why the canal is stagnant. We didn’t see that many mosquitos

before.

[00:56:46] L: Look at this canal before, it rain and water ran. But what makes them do that is,

you see and you guys see that water green and stagnant. That canal is horrible.

[00:56:58] S2:  Did you know the lagoon before, too?

[00:57:00] S1:  The one from ((fondanio))?

[00:57:02] L: The one from Podaje?

[00:57:02] S1:  No, when—when we came here to live here before they were apartments, they

had reduced. And then, after Yira got populated, the canal got reduced.

[00:57:24] L: The only one I know is the Charco Azul Lagoon.

[00:57:27] S1:  That one is from where they come from.

[00:57:29] L: Is the one that comes from—that is all good.

[00:57:32] S1:  That one was very pretty.

[00:57:34] L: Even there...

[00:57:36] S2: But that is black now.

[00:57:37] S1:  Yes.

[00:57:37] L: Yes, now is dirty water.

[00:57:40] S1:  It a focus of contamination. Impressive.

[00:57:40] L: When I studied. I passed Charco Azul, when I was well little. One of my

schoolmates drowned there, in Charco Azul.

[00:57:50] S1: One could bathe there.

00:58:20] L: No but now that is—In the shore is pure danger, invasion. People go out there to

kidnap the people who go by the avenue. They have even found dead people there..

[00:58:58] S1:  They were working on it they were working on it.

[00:59:00] Amy: It is halfway done, right?

[00:59:03] S1:  It is halfway done. They told them—What happens is that people do not

collaborate that keeps being that way. We proposed with the leader over there, doctor _____

and from Charco Azul, so they put security. Because you see, they put security and when they

relocate, invasions where there again. Because of not paying security so they don’t do that…

[00:59:31] L: What happens is the relocate and gives them a house. They rent it and (( )) so

they can come back with another person and get another one.

[00:59:43] Amy:   That’s what it is.

[00:59:43] S1:  And they rent them.

[00:59:46] Amy:  Is not the first time I heard that.

[00:59:46] L: Look, over there in Bajaluz in the hospital—What’s the name of the pretty

hospital? Norte Casino, well there is an invasion, which is dangerous.

[01:01:31] L: But there is dangerous, there are multiple raids, gangs that one cannot passed

from one side to the other.

[01:01:49] L: There in Poto Grande, they also relocated part of the people here of the

lagoon—from here of Podaje. And they relocated there. But people had to leave due to the cost

of vaccinations.

[01:02:07] L: Imagine that there is a woman there that sweeps the streets, how is it possible

that she sweeps the streets everyday and can still give someone else a vaccine for ten thousand

pesos. What she wins gives the living for her kids. And she sweeps and everything. And if they

don’t threaten her with her kid. There are people who have push down doors and everything.

[01:02:31] Do you imagine how the Chikungunya there is? People get sick to that side.

[01:02:34] S2:  And who charges that?

[01:02:36] L: Wells those guys--

[01:02:37] S1:  The same, the delinquents. The same ones.

[01:02:40] S2:  There are gangs?

[01:02:42] S1:  Gangs, a lot.

[01:02:43] Amy:  They are charging for the protections of themselves.

[01:02:47] S2:  Do the gangs have names?

[01:02:48] L: Yes.

[01:02:49] S1:  Yes. They have--

[01:02:48] S2:  Narcotrafficking or…

[01:02:50] L: No. But imagine if it were narcotrafficking, they would charge a million. But ten

or five hundred pesos no, they are vicious people, gang members.

[01:03:01] S1:  Gang members, common.

[01:03:04] S2:  ((micro traffic))

[01:03:05] S1:  Yes, it can be like that, because that comes from others—If that comes from

above and bring them so they sell them and everything and that’s how they seek life. Yes we

always talk about it here.

[01:03:19] S2:  It is to say that they bring drugs from somewhere else to sell them.

[01:03:23] L: So they can resell it too.

[01:03:24] S1:  The first doses for the kids are free (()).

[01:03:29] Amy:  And always free. Sad, isn’t it—It is like that in universities too.

[01:03:58]

[01:04:08] S2:  And do you guys see that the mosquito production here, for example, has to do

with the social problem, right?

[01:04:17] S1:  Yes, because they don’t send us—We went and they didn’t…

[01:04:23] L: Isn’t that apart of the DAGMA, no?

[01:04:25] S1:  Yes, to public health.

[01:04:28] Amy:  Yes, for example you guys are very conscious about the water, trash and

garden problem.

[01:04:36] S1:  Yes.

[01:04:36] L: But in a few curbs they are changing and that is also part of the (( writing))can go

for up to five kilometers. And is also affecting that—That sickness where they can register it.

Then, it is very connected with the social subject, no?

[01:04:54] S1:  Yes, we are very careful, because in example it has been a while, when

Chikungunya was in full force a lot of people in the area got it But we got it, we got prevention

and then that was the (( )).

[01:05:18] L: Who remembers first?

[01:05:21] S2:  When was the first time you found out about Chikungunya?

[01:05:25] L: No, because for us it was first Dengue, no? Yes, because after it was Dengue and

then Chikungunya. That was last year, right?

[01:05:32] S1:  That was last year.

[01:05:34] S2:   For you which are the mosquitoes perceive the community is that is more grave

that can kill more or do more damage?

[01:05:43] S1: It is Chikungunya. Because we—Zika has happened, what we have noticed with

the classification we have given it, it is not that bad. Better yet like a Dengue, in change

Chikungunya has killed more people and elderly that suffered from sugar and blood pressure.

[01:06:06] S2: Did something occur about Chikungunya of Alto Mayor that caused death?

[01:06:12] S1:  Yes, they’re in Yira. The lady from Yira can confirm that.

[01:06:21] L: Which lady from Yira?

[01:06:23] S1:  A lady from Yira that they told us had died because she was very elderly. She

was from there and came to our prayer groups at church and I do have her name present.

[01:06:37] S2:  And in relation to Dengue and everything, do you think it disappeared or what

happened?

[01:06:43] S1:  No, suddenly there’s none, but now we control it better.

[01:06:50] L: But for me, I don’t know, I thought Dengue, Chikungunya and Zika were the same?

[01:06:57] S1:  No.

[01:06:59] L: I think of it as almost the same.

[01:07:00] Amy:  Yes, lady, you are right, because it is the same mosquito.

[01:07:06] L: Didn’t they change that Hemorrhagic Dengue came out off Chikungunya and

Chikungunya came from Zika, no?

[01:07:14] S1: Because suddenly—I think that you know each body and organism is

different. The suddenly, each person gets it differently, so the classification should be the

same but then—The same mosquito but different classifications.

[01:07:40] S1:  Because how we tell you, for example she got Chikungunya. Chikungunya gave

her itching and she plates got there. The itching and—But it was not the Zika that I like a lady,

____, she got an outbreak and had many tiny red dots.

[01:08:08] S1: I went to visit her and she told me—I told her well if I was there in Comfandi it

was not Chikungunya, it was Zika. She told me her junctures did not her. it was a headache.

[01:08:22] S2:  Ah, but the diagnostics?

[01:08:24] S1:  Yes the diagnostic, Comfandi. The doctor.

[01:08:26] S2:  The doctor.

[01:08:28] S1:  The lady ah?

[01:08:31] L: But for me it wasn’t, ______. I went with her to Comfandi. And she went there

with body aches and swollen hands and everything. Then I told the doctor and she said that her

body hurt.

[01:08:47] L: And he told her he also had gotten it, what could he do. Acetaminophen and

nothing else I can do. One goes and then she got mad. And he told them that one comes here

so they can do something with the pain and the only thing they tell them is that is Chikungunya

and to take acetaminophen and rest. That’s all they told them.

[01:09:07] L: Rest, acetaminophen every eight hours and liquids.

[01:09:09] S2:  And the doctor said it was Chikungunya.

[01:09:13] L: There, it was Chikungunya. He told her. Good thing that that was last year,

before she got pregnant. Because now she has a kid that has (( ))—Twenty-two he has each to

months. I told her, be careful because knowing no to get pregnant because of Zika because

there are a lot of kid in TV and the news. There are cases of kids that were born affected by

Chikungunya no?

[01:09:46] Amy: Zika.

[01:09:45] L: That they get Zika in their blood and they have...

[01:09:48] Amy:  That’s why is important that they are importing it so that the secretary

notices that we have a problem with Zika, lets do something. Because that also generates a

interest in getting a vaccine or a better treatment, something likes that. I think that the

biggest problem with these diseases is that no one wants to invest money in getting a vaccine

or a better treatment if we don’t report it as one—We have to report it so they give more

interest to it.

[01:10:26] L: Of course, they put more interest. At least in Comfandi they had time when

Chikungunya was here, correct? They were giving mango water and sweet cloves and what else?

[01:10:41] S1: Pepper and cinnamon.

[01:10:42] L: No sweet pepper.

[01:10:45] S1:  Sweet pepper, yes.

[01:10:47] L: And biche mango and cinnamon.

[01:10:48] S1: Biche Mango, cinnamon y lime. And it gave results.

[01:10:50] L: And they were giving for articulations and everything.

[01:10:52] S1:  They went to ( )) and they gave it to everyone. Then to us, I went and soaked in

the formula they gave us. And we gave the formula to everyone here. The ones that had ____

we gave them, we put it on them with what we have we bough them the (( )) and that things so

they would use them. And when the other leaders we gave them and when they did not have

we brought it to then, which helped them a lot.

[01:11:18] S2:  In this moment, do you consider the epidemic or the diseases that are

prominent are due more from Dengue, Chikungunya or Zika, in this moments of rain.

[01:11:29] S1:  Of Chikungunya, the majority there--

[01:11:33] L: You hear more from Chikungunya than Zika.

[01:11:37] S1:  Because it is very strong, the Chikungunya.

[01:11:41] Amy: Ah, I have a question. Are woman really avoiding getting pregnant in this

moment with the Zika or no?

[01:11:47] S1:  Ah , yes yes yes. People are. The girls, no.

[01:11:48] Amy:  Yes?

[01:11:49] S2:  How do you identify that the girls are preventing it, like teenagers for example.

[01:11:56] S1:  Well here—We here.

[01:11:59] L: Yes, because here there are girls that their parents—Well you know nowadays

girls have boyfriends and relationships at an early age. So they put them on contraceptives.

[01:12:09] S1:  They put contraceptives, they give them at an early age.

[01:12:13] L: They put the contraceptives and they put them on them .

[01:12:17] S1:  Since they are twelve they put them on contraceptives. twelve, thirteen,

fourteen, fifteen…

[01:12:27] L: Yes, because today a lot of girls at thirteen and fourteen already have

relationships.

[01:12:32] S1:  When we—When I worked in Norte Casio, we would do that. We went and open,

_______ , _______’s mate with another ((choquiana)), a lady too. And we were going to get in

contact with girls from nine and ten years that were pregnant and everything. And then we

would take to Pro family and they were going to but them contraceptives.

[01:13:15] Amy:  That stick that they have there under the skin.

[01:13:18] S1:  Yes, they put it here, because they put it. And they are like sticks, like this

long.

[01:13:27] Amy:  Yes, and it last like four years.

[01:13:28] S1:  And that last, no. Last four years.

[01:13:30] L: Four years. Yes, you have to be very careful, to check on then because they run a

lot…

[01:13:35] S2:  Have you see Zika cases in pregnant woman here?

[01:13:39] S1:  No, thank God no.

[01:13:42] S2:  What have you heard about microcephaly in relation to Zika?

[01:13:46] L: Ah that Zika does it, no? That is born with children—it affect them and...

[01:13:52] S2:  But there haven’t been cases here.

[01:13:54] S1:  No. No cases here at all.

[01:13:56] L: In the news we have heard that in Barraquillas, Santa Marta and those parts, kids

have been born with microcephaly. That makes the head like big.

[01:14:09] S1:  Yes, but look--

[01:14:13] S2:  Microcephaly.

[01:14:13] L: Ah micro makes it small. And macrocephaly is big no?

[01:14:21] S1:  Loo, not here, thank God. And neither there, what we have noticed, we

communicate with the leaders a lot because--

[01:14:31] S2:  Is there a lot of Zika there?

[01:14:33] S1:  There’s it happens a lot—Not Zika, Chikungunya.

[01:14:40]

[01:15:39]

[01:16:46]

[01:18:30]

[01:19:15]

[01:19:52]

[01:20:06]

[01:20:14] S1:  Then we have classified very clearly about the mosquito, which is the biggest

and has white legs.

[01:21:06] S1:  Another detail we missed in the racquet.

[01:21:16] L: That traps the,.

[01:21:18] Amy:  They sell them in the streets.

[01:21:20] S2:  Electric.

[01:21:20] S1:  Yes electrical that sounds. Is Rechargeable. And it smells, bad.

[01:21:26] L: You can hear it in the house. I say oh, they are using the racquet.

[01:21:30] Amy:  I used it (( )) in the house when I saw a mosquitoes. They kill it with that ((

))

[01:21:36] S1:  They kids have fun with that. But we kill a lot of them with that. When you see

the crown that they form here, it is a small amount that last.

[01:21:47] L: I used to buy the eucalyptus and I put it there. I put it all around the house for ((

)) of Eucalyptus.

[01:21:55] S1:  They died with eucalyptus. The house here is impregnated.

[01:22:00] S2:  And the mosquitoes do not come in.

[01:21:59] S1:  The mosquito does not come in anymore.

[01:22:04] S2:  In which part of the house do you think mosquitoes are more?

[01:22:09] S1:  The closets, the wardrobe, and the bathrooms.

[01:22:11] L: For the—the seats since they are dark, where they darkness is there are the

mosquitoes.

[01:22:22] S2:  In the darkness. Underneath or over the seats?

[01:22:22] L: No, behind—of the house since it is a rotunda in that side.

[01:22:27] S2:  Behind the seats.

[01:22:28] L: My mom has a rocking chair there in the corner of the window and in the

darkness, when you turn on the light in that darkness.

[01:22:41] S1:  Yes because they seek the darkness, no? While they had light then—I used to

have a lamp and I put it away. Because the lights makes them go away. But these days, not

even the light affects them.

[01:23:02] S2:  When did it start increasing, more or less? When the rain started?

[01:23:02] S1:  Yes, when the rain started.

[01:23:05] S2: It started again.

[01:23:06] S1:  Because that canal was dry because of the arrangements that they are making.

They covered here to work on the part over there. But apparently they left it dry here and

went to work on it over there.

[01:23:22] S1:  It results that since the rain came in a moments notice, it got full. But then

they should have came and drained it or open it so--

[01:23:36] S2:  But what labor has the community board done for it to happen? So they take

that out, they take that out…

[01:23:43] S1:  But we tell the president to make a letter and everything. But what happens is

since we are in a moment of community board change they are neglecting that part.

[01:23:59] S1:  We went to Cali Trece and we asked to collaborate to see if they drain the

canal and they made it work.

[01:24:10] L: But they said that they were going to let the water run first.

[01:24:11] S1: But people there—I told you right now. They did not let them because if they let

the water run the extract of the apartment increases. Because the other apartments are

extract two and we are extract three then it will increase for them. The extract would

increase.

[01:24:28] L: But what happens is—since it has not rained hard and because that overflows.

[01:24:35] S1:  Because that is stagnant.

[01:24:38] L: And there are parts of Calipso that don’t flood, no? Because the house that—the

ones that flood are from the corner towards there, correct?

[01:24:49] S2:  From this corner.

[01:24:49] L: From this corner no, the other corner to there it floods. There’s part of Calipso.

[01:24:56] S2:  With the canal.

[01:24:57] L: And when it rains hard and the canal overflows.

[01:25:04] S1:  But them since the canal used to run then they put a lot of trash in it but they

kept cleaning it.

[01:25:13] Amy:  Yes? Who?

[01:25:15] S1:  We—That came from the industries and they maintained it because we kept

sending rights of petitions. Then they kept it clean.

[01:25:24] S1:   But since we started that job for the (( )) then going to take it out but they did

not let them. Then, more later what they were going to do was for cleaning the—They started

here towards there to clean the lagoon de Charco Azul.

[01:25:46] S1:  But they did not count on—They left that clean but did not count on the rain

and it got full. But they have not done anything to come and fix it.

[01:25:57] L: Yes, because look, the last year or this year, they found the body of a woman

there that had disappeared.

[01:26:06] S1:  Over there in Exito. Next to Exito, they found a girl there.

[01:26:09] L: They found the woman’s body there. How that is there, it lends to--

[01:26:17] S1:  It lends for people to go—and they throw a lot of trash there.

[01:26:22] L: They also killed a soldier there too, no? He came to visit his mom and went out.

Came to say goodbye, went out and they killed him.

[01:26:33] S1:  Then it lends to more things, no? Well it is good for the disease but there are

quantities. The ones from Yira, from the said over there of Yira and more than anything there

in Comunero there’s an invasion. Cinta larga.

[01:26:54] L: No but there is one, Paz, Florida, and there are many that I do not even know.

[01:27:01] S1:  Yes—the diseases there are a lot. Do you imagine if they used to have diarrhea

and all those things in the skin they are worse because about the mosquito. Then there is the

stagnant water and we are waiting for the things of the board.

[01:27:31] L: Well let’s see what happens if the product--

[01:27:35] Amy: more or less, when did the rain start?

[01:27:38] S1:   The rain, like twenty days ago o a month now.

[01:27:43] L: It started a little bit in March and now in April.

[01:27:48] S1:  Yes, late March and the beginning of April.

[01:27:50] L: I saw a news story that said the rains were until April.

[01:27:57] S1: June.

[01:27:57] S2:   From April to June, you guys expect more mosquitoes.

[01:28:03] S1:  Yes, we are waiting—Evolving a lot to se if the come and open the canal so that

comes out.

[01:28:13] L: They need to get machines and get all of the dirt because is like there is—In the

bridge because one passes over here and on the side of ((Ilícito)) and there is trash stuck there.

I t does not let the water run and the water is green.

[01:28:33] Amy: And it smells bad.

[01:28:35] L: Did you guys go there?

[01:28:36] Amy: Yes.

[01:28:37] S1:  They filmed there.

[01:28:38] Amy: In drought and in rain season. But in drought I went in January and it has

stagnant water.

[01:28:45] L: Yes because of…

[01:28:46] Amy:  But lower.

[01:28:49] S1:  Yes, because they were working. But it was cleaner because they kept it that

and we were always vigilant.

[01:28:59] S1:  But now it got out of our hands because they did not count on the news that the

Niño phenomenon was going to be until June and look, everything went bad, because it started

in March. Then, they did not count on that. So they were working there.

[01:29:22] S1:  And now, we then told them, we understand so let’s fix it. Fix it for us. There’s

no time to say that happened. No, it happened and we are living in something very delicate.

We told the ones in Cali Trece.

[01:29:38] S1:  They were going to send them public health and the DAGMA. And then over

here, we send the municipal industries. The one we always pressure so they can clean the

canal.

[01:29:51] S1:  Then, we are here in the wait.

[01:29:54] Amy:  Yes because the machine is quiet.

[01:29:58] S1: Yes that is like no one is still. But it is because what they are working is more

over there.  And they did not come back—And they come and go to eat there in Abuela. Then,

that’s were we get them.

[01:30:14] S1:   No, we told them that and we were there. Well, no but we can collaborate and

they told us to send a rights of petition to Cali so that Cali

[01:30:25] S2: And they sent it?

[01:30:25] S1:  Yes, we sent it.

[01:30:27] S2:  You are waiting…

[01:30:27] S1:  We are waiting.

[01:30:29] S2:  Since when did you send it?

[01:30:32] S1:  No, it was barely last week.

[01:30:32] S2:  Oh Okay. They are waiting for an answer.

[01:30:33] S1:  Yes because we—Yes because we were in that story of boards and then we have

made seminars, meetings and we had no time.

[01:30:44] S1:  And then, we asked for a collaboration from the doctor of Cali Trece so she

could help us, with the DAGMA and public health.

[01:30:56] S1:  And then they told us—They put Cali, then we already did the rights of petition

last week and we are waiting.

[01:31:06] S2:  There are fifteen days for--

[01:31:10] Amy:  It works with more cards here, more right of petitions more responses, or not?

[01:31:14] S1:  Yes, Yes. We, Thank God. But it is everyone’s pressure.

[01:31:22] Amy:  Yes.

[01:31:20] S1:  We sent the right of petition and that way everyone came. People collaborate

and that is how we start collecting signatures, we did it under here, we had a delicate

problem. Because we had the—Where the water came out of Yira, of Comunero, everything

ends in the apartments in a intake that was there. And that got full because the box was to

small to get everything that went down. And then it flooded and it blocked. And we would get

flooded until here in that part. And all of those houses flooded.

[01:32:08] The home appliances and everything. And they did not respond. And we got to work

with _____.

[01:32:14] L: But at the start, the water got here.

[01:32:19] S1:  Si, until here.

[01:32:18] Amy:  In that house? Until here?

[01:32:21] S1: Yes, it came until here—the water came out of the bathrooms, through the

siphons. And it flooded, but impressively.

[01:32:30] L: And if you saw the rats that would come out of there. They looked like cats.

[01:32:31] S1: The problem is impressive, right? And then we got in charge. We—We got tired of

making rights of petition. Some lawyers collaborate with us and then they were with political

figures and then lawyers—The political figures helped with the lawyers and we made a—right of

petition no?

[01:32:58] S2:  A ((tutena)).

[01:33:01] S1:  That is for the community. Well is a demand.

[01:33:08] L: Not conformed or something like that?

[01:33:11] S1:  Is a demand in a community level and that’s what we did, with gathered

everyone’s signature. And we started, and the demand went out in a certain date and we were

there in the judgment, there in Cali.

[01:33:26] S1:  And we pressured them until they made our case and came and everything and

they fined Cali. Because that—Because since then since they made this houses, they knew

about that problem. That we should have our own sewers but we share it with others.

[01:33:52] S1:  Well, we have won the demand.

[01:33:54] S2:  That fight.

[01:33:55] S1:  Yes. And they came and fixed everything. Now it is flooding and that is the

problem there. Because it comes dirty and is stagnant with a lot of filth and everything and

that is why it blocks—But that’s it thanks to God.

[01:34:15] Amy:  I think they have helped us a lot.

[01:34:49]

[01:35:11]

[01:35:58]

Calipso y Yira Castro April 14 2016.WAV

Neighborhood: Calipso y Yira Castro

Date: April 14 2016

[00:13:16] S2: throw in--

[00:13:17] A: Is a dump.

[00:13:18] S1: In transformed into a dump. But there were some areas that there was only-- you remember, that there were some plants and they only flourished there.

[00:13:27] S2: And they flourished. And you could find a lot of frogs all of those weird things. Pretty things.

[00:13:37] S1: There were animals that you could not find here but there in the lagoon they lived.

[00:13:44] S3: The population that is close to the canal is placed else where or

[00:13:49] S1: Not all, but there is some.

[00:13:51] S2: Yes, in the beginning that is why it was conserved. Because people where proprietaries.

[00:13:59] S2: But from a moment to another one, came the invasion. Then they did that on all the shore. Starting that also started her. Yira was also a settlement and they organized them why.

[00:14:15] S2: Why they took care of that. Then that's why the allowed and they gave them the--

[00:14:32] S1: Before, they had to refill it, because it reached up here. They refilled it to locate them. Because part of this neighborhood is on top of the lagoon.

[00:14:48] S2: This was part of the lagoon. These neighborhoods, us the Yira, where part of the lagoon.

[00:14:59] S2: The cut from it. Now the only thing left is what we call the canal. Because they reduced it and put cement on it's sides and it turned into a canal.

[00:15:16] S2: But why? So they could invade. People invaded and left only that. Then what happens today? It is big there, but what happened? They came and the people that bought left the capacity of the lagoon.

[00:15:36] S2: But the invaders came and invaded that surroundings and started to get it dirty and contaminate it. And then all of the plants, everything it turned into something harmful for us.

[00:15:56] S2: What before, in contrary, was very pretty. You can find the photos there very pretty.

[00:16:14] S2: We are trying to recover it. All of the people in it's surrounding are being relocated slowly.

[00:16:24] S2: They have cleaned it. Then why is the objective here? Here they wanted to cover it. That part, but people did not want to.

[00:16:35] S3: Cover it how?

[00:16:41] S2: is to put cement on top of it.

[00:16:43] S3: Like a layer?

[00:16:43] S2: Yes, that. Not closing it but just covering it. So avoid what we are talking about.

[00:16:50] S2: Then, the people from the apartments did not allow it. Because of that, their extract has not gone up. They are extract two. Which you know is the extractification.

[00:17:06] S2: They are very pretty apartments, but since, they do not call it lagoon, if not the canal--Then, if they cover that, then they would raise their extract. Then they did not let them do it.

[00:17:23] S2: Then what, they are recovering it over there.

[00:17:28] S3: Towards their side?

[00:17:29] S2: From here on, what’s the name?

[00:17:32] S1: Yira? When you're going to go--This is very close to Yira Robles and them she talks about that side, to the side of Yira.

[00:17:41] S2: Yes, if that keeps up like that and you go to Comunero and look that the zone that is in disadvantage is the one close to the lagoon.

[00:17:52] S2: Then, what happened? They left this here to start working over there. But they did not think it was going to rain so much. SO that has gotten full and that water has become stagnant there.

[00:18:14] S2: Then, what happens. That affected us in that sense. Then what happens here?  Since we have a lot of green areas, then that is where they accumulate. We went and looked and we moved the-- and a big amount came out.

[00:18:33] S2: You can not do, for example, yesterday was a day that, better said we got there at seven and we met at the park with don _____ and some leaders and there we were talking and putting care into what the youth was...

[00:18:53] S2: And right there, when we looked, it was impossible to stay there. Someone talked and the mosquitoes go into our mouths.

[00:19:01] S3: And at what times are the mosquitoes going out?

[00:19:04] S2: They have a-- Better said, at six of the afternoon they are--people have opted for, and we too, to burn eucalyptus, and that makes them go away.

[00:19:24] S2: Here *﻿*﻿I have, and a lot of people here too, grottoes which has a pump and it with water and everything.

[00:19:36] S2: We had them. I put hypochlorite on mine so it conserves better. But look if is so strong that mosquito that it filled with them. Look how it is now.

[00:19:50] S3: Hypochlorite?

[00:19:50] S2: Hypochlorite. Look at it and it still grew there, the larva.

[00:19:58] S2: And then we all reached an agreement because we talked to all of them.

[00:20:00] S1: What happens with Calipso is that it is a very organized neighborhood. From the district, is the one that does not look like a district?  Because it has apartment there and there that are like if they were from another extract.

[00:20:14] S1: Because they have income. People here trust that they established Calipso. Then, they made sure that is was not just any neighborhood. A different neighborhood from all from all of the one's that surround them.

[00:20:36] S1: Then, when we go around, this things they do not have them inside, if not outside. That is something that characterizes the neighborhood.

[00:20:48] S2: I have one outside, which I am barely using. In the garden.

[00:20:51] S1: In the garden. Well you see a lot of that, small water fountains, that is what identifies them.

[00:21:02] S2: Yeah, here they call the entry door of Calipso, is the entry door of Aguas Blancas district. That's what we always say, that the district is not like everyone says. I am always proud of saying I am from here.

[00:21:27] S2: Why? Because they delinquency and all that occurs here, it is not because people are bad, we are not like that.

[00:21:35] S2: If not it's unfortunate that there are people that come, invaders. And they are the ones that suddenly--but the owners, the ones that were born here, the founders and all are good people.

[00:21:51] S3: Where do the invaders come from?

[00:21:53] S2: They are from Cauca. They are displaced. They come from Cauca, from over there in Pasto. They come from all of those parts over there. And there is too much.

[00:22:06] S2: Do you remember the yellow colony? That's why it is called like that, because they come from Narinjo. And they are-- I used to work in the Duarte Cancino Hospital. And it was impressive.

[00:22:37] S2: We had an invasion close to Duarte Cancino. As a nurse, since we did social work and everything, and it is really hard that they let people in. Whoever it's let in is better-said won.

[00:22:53] S3: The colony.

[00:22:53] S2: In the colony. There, when the nurses needed to go to the house hospitals where they lived and to the (( )) there. They got here and she was saved from being killed.

[00:23:09] S2: Because, when they were in the hospital, she treated them badly. Then, she won that problem. While the others, we lived with them because they are people just like us.

[00:23:20] S2: And you need to know how to treat them and get to them.

[00:24:30] S3: And it is also a area of mosquitoes?

[00:24:33] S2:It is impressive. And is the amount of people. The leaders here got acetaminophen and we did-- they taught me, I was in Comfandi when the Chikungunya was rowdy. And there in Comfandi they taught me how to make a recipe that is excellent and natural.

[00:25:06] S1: Which?

[00:25:07] S2: You take three daily. Three green mangos, not biche. And then you peel it and cut it. The you put it in a litter of what and put pepper balls in it, cinnamon and lime.

[00:25:37] S2: And you boil it. And that water you take it all day with Acetaminophen. And then that took away the articulation pain. The we would make a lot of it and we went through the parts where the was a lot of people and we would take them some.

[00:26:03] S3: There are a lot of people Chikungunya?

[00:26:05] S2: To many. It is impressive. And here, is also impressive. And when that happened they were working there but it was not raining, the humidity is what brings them here.

[00:26:18] S3: Remember that we talked about how there was a very impressive drought here. That it never rained. But with all of that, there was still the situation with Chikungunya.

[00:26:32] A:  I was recording the canal in that moment too, when we were in drought. And with the rain...

[00:26:42] S2: It increased, the Zika. I think it increased, because you do not see Chikungunya that much anymore. Look, there has been a big amount and we have done that a lot of times over there. And for what, it worked a lot because people here also did it. But people here did not...

[00:27:08] S2: The people that are more capable, not that we are better than them. But them people would buy their things and we would also give it to them. Then here, and look that for example the people from Alta Mayores, a lot of them died because of that.

[00:27:32] S2: Because it affected people with diabetes and Hypertension and they did not stand that, with those pains and everything, and a lot of people died because of that.

[00:27:46] S3: With everything that there was a lot of sick people, where they register in the health center? Or is it because people said that they had Chikungunya, or dengue.

[00:27:54] S2: No because they, in example, there are a lot of people that are careless. We have the Sisben and that is excellent. But there are people that do not interest them. They [00:28:03] S2: Oh, why am I going to go there. No, with these pains and fever to go make lines and I don't know what else. No, I’ll stay here in my house. And so they can send me to buy Acetaminophen and I do not have anything to buy it with.

[00:28:16] S2: Then that is why we go to them. Because the leaders told us, we have this problem.

[00:28:22] S1: To me knowing how you guys knew how to know if it was Dengue that it was not Chikungunya or Zika appears to be important.

[00:28:29] S2: because no. Then, we did not know about Zika. It was just Chikungunya. And the symptoms were--And for example, since I am a nurse and they have a lot of faith in me. I went and got soaked in the health center.

[00:28:51] S2: Then, they would give me the information. I would come and put the information in my house, the posters they would give me. Then, I would share with my partners, the leaders of other neighborhoods.

[00:29:08] S2: I would tell them: on this date and this time we are coming to do a brigade. Then, suddenly they did not know, they said: “OH, you only take a acetaminophen and that's it" And no. That is not like that, you had to take it every four hours.

[00:29:28] S2: With a lot of judgment and everything. They told us the induction and suddenly they gave us the authorization so we could do that.

[00:29:42] S3: What I see, is that a lot of cases were presented symptomatology but no diagnosed.

[00:29:49] S2: But not diagnosed because people did not like it.

[00:29:51] S3: Simply because people would say they had articulation pain, I have fever, is dengue or they matriculated they as they said.

[00:30:01] S1: How do you differentiate?

[00:30:02] S2: Now a days, the outbreak. The outbreak. Because in Chikungunya, the outbreak was more thick as Chikungunya.

[00:30:17] S2: That was a key that we had. Now a days we have.

[00:30:19] S1: The outbreak of Chikungunya is more...

[00:30:23] S2: More thick, it was more...

[00:30:25] S1: And Zika?

[00:30:25] S2: Is more-- it left space. Yes, that. Then, where there was it would accumulate, the Chikungunya it was sparser and it itches, while Zika it was continuous and you would scratch it and spread it.

[00:30:54] S1: And dengue?

[00:30:54] S2: And with Dengue, a lot of people would have outbreaks and it was red, it did not have pores.

[00:31:08] S3: It was skin colored.

[00:31:10] S2: And some people, because some people did not have it. And that difference between those three, we saw it like that and that is why we identify and classify it.

[00:31:26] S2: For example here, we have three nurses that are-- we do community work. Now a days, I do not practice. I dedicate my time to that, with hers. We are three and we do social hours.

[00:31:47] S2: We recollect the drug that a lot of people, more than anything the ones from here because the ones from Calipso we have a lot of people that bring drugs to us.

[00:31:57] S3: Medicine.

[00:31:58] S2: Yes medicine.

[00:32:02] S2: The medicine but we do not give the medicine out to any one. We go and do brigades. We take the medicine...

[00:32:19] S2: Medicine that we have already there. We look at the expiration date that is good. And we go do the brigades.

[00:32:29] S2: We take the medicine, and if the woman calls a lot for the blood pressure, (()), the medicine that they have sent them and people do not go to reclaim them, because people are like that.

[00:32:47] S1: To the side were you kept going, did you guys identify that there was a lot of cases. Have were the streets? Did they have puddles, pools of still water and streets without pavement?

[00:33:06] S2: Yes. And the houses since they are invasions, and since they do not have-- they do things very traditionally. For example they put a tub through the whole half, artisanal, half of the house, since they do not have pavement, then that is buried.

[00:33:33] S2: Then they put that through the half of the house and the water passes through there. And the smell, the rats and they live with all of that.

[00:33:44] S2: That's how those people live. Those kids. We are terrified. A lot of people, we gift them ballasts so they could put it in those things.

[00:34:00] S2: We gave them tubes, so they put a connection there and hide that. And that brings a lot of humidity. And not even the ((tornillos)), we would go with the other nurses and the leaders to ask for them to collaborate with the ((tornillos)) that they did not need anymore in their houses.

[00:34:25] S2: We gave them ((tornillos)) but they did not even use them. We would go and they did not even had them on, they had store them. Sometimes, things happen to people because of not caring, not because they do not have.

[00:34:39] S3: And what excuse do they have to not use the ((tornillo)).

[00:34:44] S2: No, they tell lies. They say they put it, when they go to sleep they put it.

[00:34:51] S1: And what do you think happens?

[00:34:54] S2: No, people are lazy. Like, they are indifferent to all of those things.

[00:35:01] S1: There’s no care in their heath.

[00:35:02] S2: Yes, very indifferent to all of that. They think that it is a lie. That people do not die of that that it is something that will pass.

[00:35:15] S3: When you talk about people that took Acetaminophen, did you find any cases, for example, that they auto medicated with that or any other medicine.

[00:35:24] S2: Yes, they would go to the drugstore, they would say they go there but that is not--Oh the drugstore is selling a medicine that it cures it. And I told them never.

[00:35:39] S2: Do not say that because that never--that is not controlled yet. You control it with Acetaminophen, no more.

[00:35:49] S2: They would say that no one could stand the pain in the articulations, and I would tell them that that is the problem. The mosquito bites and that is what affect the articulations.

[00:36:01] S1: Was there ever a moment when people said that it was not because of the mosquito, but because something else?

[00:36:05] S2: Ah, yes. They said, since there was a drought, they said it was the a lie. The ones above would say that is a mosquito, but is a lie, but to make it rain, they went up to the clouds and put a chemical and that is what has us like that.

[00:36:41] S2: And that chemical got to the rivers and where we consume the water. And that is what has us like that, but what mosquito we have always had mosquitoes.

[00:36:51] S2: You look; the mosquitoes are stronger now than before. Look at the difference between it now and before.

[00:37:11] S2: Then that is what they would say. But now a days... they have created a conscious about it. They say that there are a lot of mosquitoes.

[00:37:31] S2: And we would ask them, what do you do for all of the mosquitoes, how can you sleep?  Ah no, we put eucalyptus.

[00:37:40] S1: A plant?

[00:37:40] S2: Eucalyptus is a plant and you know what they say. That is from the eggs. The carton where you put the eggs. Is really effective but is bad for the lunges.

[00:38:01] A: They are burning that in the houses?

[00:38:03] S2: Those chemicals, people are burning them in the houses.

[00:38:05] S3: Is there a lot of respiratory diseases because of that?

[00:38:08] S2: Because of that.

[00:38:09] S3: The use of Eucalyptus and the carton?

[00:38:11] S2: Not Eucalyptus, the carton. They say they were the ones who taught us because we did not know. We sometimes are in the park and we do not have Eucalyptus so we use that carton.

[00:38:24] S2: And if you saw how they go away. I do not know what chemical that has but that is bad because you start coughing when you turn it on. They use it because they find that everywhere.

[00:38:43] S3: Where do they find the Eucalyptus.

[00:38:44] S2: There are trees, for example they’re in that sector there is one.

[00:38:45] S1: They are trees.

[00:38:46] S2: They are trees.

[00:38:48] S1: It is beautiful.

[00:38:48] A: And smells good.

[00:38:48] S2: And smells delicious. They have a lot of them, but they get more since Eucalyptus is used in medicine, so it does not harm.

[00:39:05] S2: It excess is bad because of the smoke but just a little bit you can even use it in the bathroom. For example, I put Eucalyptus a lot in our bathrooms because that scares the mosquito away.

[00:39:18] S2: But since they find that in the trash, those cartons, then they use them.

[00:39:25] S1: In the recycle bin.

[00:39:25] S2: Then they do it, but they are getting worse, because they are getting better of something but worsening with another one.

[00:39:35] S2: Which is the lunges.

[00:39:59] S3: How are you managing, something you told was very characteristic of the neighborhoods, which are these...

[00:40:06] S2: The grottos.

[00:40:07] S3: Yes, and if it was understood that it was a form of...

[00:40:12] S2: Oh, it was impressive.

[00:40:14] S3: And how is it being managed?

[00:40:15] S2: No, everyone came onto agreement and we do not turn them on. We do not turn them on.

[00:40:20] S3: You simply do it.

[00:40:21] S2: Simply does it. The people that do have them have them off. And the people who have not made them, they won't do it. Because we tested with the hypochlorite. I had it on, it was very pretty, and it has lights and everything.

[00:40:45] S2: And since that does not work unless it is with water then. I tested it and I had it like that and I put the hypochlorite. And I let it there.

[00:40:57] S3: How much hypochlorite did you put?

[00:40:58] S2: I put about a cup on it.

[00:41:00] S3: The normal one they use at home?

[00:41:04] S2: In houses? No, the one for the floor. Limpid, no, because the hypochlorite is stronger and more disinfectant.

[00:41:14] S3: (()) or what?

[00:41:17] S2: The limpid which ever you buy is different to the hypochlorite because that is the one we use at the clinics to disinfect and everything and stronger.

[00:41:30] S3: The percentage is higher.

[00:41:31] S2: Higher yes.

[00:41:33] S3: Than the normal, which is five percent.

[00:41:36] S2: Yes, then we tested, I tested together with my other partners we put it.

[00:41:43] S3: It is to say that not even the hypochlorite that was used in the clinics is strong enough. You saw that it did not give results.

[00:41:47] S2:  That it did not give any results. Because we thought because it that works, than we turn them on and nothing happens.

[00:41:56] S2: But we can't risk it. And we even tried it for three days and it got full with live larvae. Then, we said, we have to get rid of that.

[00:42:13] S3: People recognize the mosquitoes that produce Dengue, Chikungunya and Zika?

[00:42:21] S2: They, we all say that they are the biggest. Because from mosquito to mosquito, there's small ones and there are big black ones.

[00:42:35] A: They have white legs.

[00:42:37] S2: That, they have white legs.

[00:42:39] S3: That one?

[00:42:39] S2: For us, that is the one.

[00:42:42] S3: Black, with white lines.

[00:42:52]

[00:43:53]

[00:44:55]

[00:45:55]

[00:46:57]

[00:47:57]

[00:48:56]

[00:50:00]

ZOOM0002.Wav

Neighborhood: Calipso and Yira Castro

Date: April 10-16 2016

[00:00:07] A: We are in Calipso and Yira Castro.

[00:01:00]

[00:02:00]

[00:02:56] D: This is Calypso’s canal.

[00:02:59] D: They were fixing it.

[00:03:01] D: They put some sheets on it and they want to know if the water is running or is

stagnant.

[00:03:07] D: Look how the canal is. It is completely stagnant.

[00:03:13] D: You know why? Because of the construction more ahead.

[00:03:17] D: And look at the larvae how you can see them there.

[00:03:21] A: All that movement is larva?

[00:03:23] L: Yes.

[00:03:33] D: Yes, look at how they are moving in the water. Is because they are fixing

something more ahead and the machine is inside in the middle

[00:03:43] A: But for a lot of months?

[00:03:45] D: Yes.

[00:03:45] A: Is it like that for a lot of time?

[00:03:47] D: Yes because the other time we came those borders in the wall weren&#39;t there.

They were making them, of the canal.

[00:03:55] D: It was in concrete and it had those green costal up there.

[00:04:00] D: And they made that block there. And they are making those blocks but more

ahead then the machines

[00:04:07] D: are across the canal. And since they are crossing it and have materials in the back

that

[00:04:12] D: Does not let the water run, it becomes stagnant.

[00:04:17] D: But look at the amount of larva that you see there.

[00:04:30] A: That is a very important hot spot.

[00:05:09] D: That is why this is the worse hot spots in the map.

[00:05:44] D: Look on the other side they are fixing something so the water has become

stagnant.

[00:05:49] D: And that bellow that bridge has to be a breeding site. Big. And from here to there

must be too.

[00:05:56] D: And here bellow that bridge filled with streets, all of them there have the risks of

getting one of these diseases.

[00:06:03] D: But yes, all of those are larvae.

[00:06:11] D: All of that is larva movement.

[00:06:23] A: But they are not biting me here. And we are in a time where they should be

biting, no?

[00:06:31] D: The hour, for me, of the mosquito is in the morning and the night.

[00:06:35] A: Yes.

[00:06:35] D: At six of the afternoon.

[00:06:38] A: But also, those mosquitoes go to the houses. They do not stay here, they search

in houses too.

[00:06:45] L: Where there is more density of people, no?

[00:06:49] A: Yes. They like houses more.

[00:06:54] D: This is Calypso’s most dangerous hot spot in my opinion.

[00:07:12] L: At least there is one mosquito that flew by.

[00:08:00]

[00:09:00]

[00:09:59] D: Look this is a new street; it was not here when we passed last time.

[00:10:02] A: No?

[00:10:03] D: All that filth you see is from the strainers of rainwater. Look, that are (()).

[00:10:12] D: That wasn&#39;t here. And you could see it back there too.

[00:10:18] D: They are brand new.

[00:10:22] D: Then since water became stagnant they made them again.

[00:10:30] D: This street the other day, you see how the asphalt is really black, this street was

not here. It was all uncovered.

[00:10:38] D: You see the strainers that are new, they have not came to clean the filth that

was left.

[00:11:38] D: In this side of Calipso is where they are fixing the canal, towards there.

[00:11:44] D: The mosquito reaches this entire kilometer, to move from there to here. Some

should come from there too.

[00:11:52] D: Because further there the water is more stagnant.

[00:13:07]

[00:13:16] D: Look at the stagnant water there, look.

[00:13:20] A: In the park with all the kids.

[00:13:23] L: In the parks is where you see more tires and waters.

[00:13:30] A: There is movement in that water from larvae.

[00:14:00]

[00:14:20] D: A look at the water how is stagnant here in the street.

[00:14:22] A:  A lot.

[00:14:24] D: That is not from now or that they washed. That is rainwater.

[00:14:35] D: Since it is raining season in this ugly streets water gets stuck there more.

[00:15:00]

[00:16:00]

[00:16:20] D: There is a lot of stagnant water, look.

[00:16:24] D: Stagnant water everywhere.

[00:16:42] A: There is stagnant water in that passage way too.

[00:16:47] D: Those small canals that they use as a drain, there&#39;s stagnant water in the sides.

[00:17:00]

[00:17:15] D: There&#39;s a lot of stagnant water, look, on the sides of the street.

[00:18:00]

[00:19:00]

[00:19:30] D: Look at the stagnant water in the (()).

[00:19:34] A: A lot of water.

[00:20:00]

[00:21:00]

[00:22:00]

[00:23:00]

[00:23:23] D: A lot of mountains like these...helps.

[00:23:26] A: A lot of mountains. A lot.

[00:23:31] A: It likes being there. Waiting.

[00:23:34] D: There is a lot of tires lying there.

[00:24:00]

[00:24:30] D: A lot with a park of tires.

[00:24:50] D: This is the big lot I told you about.

[00:24:53] A: Ah yes with the ((energy)) correct? I remember.

[00:25:03] A: The mosquito park.

[00:26:00]

[00:27:00]

[00:28:00]

[00:28:35] D: Big streets like this are stagnant.

[00:28:40] A: At least it is not rising and going out.

[00:30:00]

[00:31:00]

[00:32:00]

[00:32:49] D: Look at the canal. I told you they were fixing it and it is stagnant, look.

[00:33:00]

[00:33:15] D: Look how it is there. And here it is bigger.

[00:33:20] A: And filled with filth.

[00:33:24] D: And look at the machine there. That is why the one here is fixing things and

making the water stand still.

[00:34:00]

[00:34:03] D: Look at the stagnant water in the streets too.

[00:34:39] D: This is the machine that works in the canal. A (()).

[00:35:00]

[00:35:46] A: It smells really bad.

[00:35:49] L There are smells here.

[00:35:52] D: Since they are constructing the rainwater canal, look they are barely over there.

See how you can see the concrete blocks.

[00:35:59] D:  In the others, the machine needs to get in and get the filth out and scrapes the

side to leave the mold where

[00:36:06] D: where the construction is going to be. Then by doing this, the water gets more

stagnant.

[00:37:00]

[00:38:00]

[00:38:07] D: Look how the water is there.

[00:38:09] L: Uh, look at that.

[00:39:00]

[00:40:00]

[00:41:00]

[00:42:00]

Audio File: C:\Users\Amy\Google Drive\Kent\james\dissertation\chkv and dengue\data\video data\Floralia_nov28\ZOOM0004.WAV

Associated video files:

1. C:\Users\Amy\Google Drive\Kent\james\dissertation\chkv and dengue\data\video data\Floralia_nov28\camera2\FILE0267.MOV

a. Transcript: [00:02:19] Paola: This is a dead end but here is a park and we have to film the park because they are foci [of vectors]

b. Video: [00:04:59]

2. FILE0015.MOV

a. Transcript: [00:02:19] Paola: This is a dead end but here is a park and we have to film the park because they are foci [of vectors]

b. Video: [00:04:50]

3. FILE0225.MOV

a. Transcript: [00:02:19] Paola: This is a dead end but here is a park and we have to film the park because they are foci [of vectors]

b. Video: [00:04:48]

Neighborhood: Floralia

Date: November 28, 2015

Interviewer: Amy Krystosik

Ministry of Health: Paola

Driver, vector control specialist: David

[00:01:55] David: We will go down the streets that are vehicular. They are all pedestrian streets

[00:02:19] Paola: This is a dead end but here is a park and we have to film the park because they are foci [of vectors]

[00:02:33] David: if you want, get down and film it

Paola: let's go

[00:02:45]

[00:02:55] Amy: Then we are in the principal park or what?

Paola: No, this is the park where....

[00:03:16]

[00:04:05]

[00:04:15]

Amy: Look, there are also lots of tires

Paola: Yes, the yes them to plant plants

[00:04:42]

[00:05:01]

Amy: Well, I see lots of breeding sites here. Tires,

Paola: Look, the mosquitoes come out []

[00:05:21]

[00:05:42]

Paola: This has maintenance; it doesn't have anything. Its clean.

[00:05:50]

[00:06:06]

[00:06:33]

Paola: Its dry. In these days with the sun

[00:06:40]

[00:06:49]

Amy: flat roofs too.

Paola: The flats roofs collect water too

[00:07:02]

[00:07:16]

[00:07:29]

[00:07:47]

[00:07:57]

[00:08:08]

[00:08:26]

[00:08:42]

[00:08:49]

[00:08:59]

[00:09:12]

[00:09:19]

[00:09:27]

[00:09:34]

[00:09:56]

[00:10:18]

[00:11:33]

[00:12:27]

Amy: The streets here are better

Paola: yes

Amy: Not as many holes

[00:12:34]

David: []

[00:12:46]

[00:13:03]

Paola: We are here with the football field

Amy: They maintain this football field well

[00:13:15]

David: And here is a greenery with plants and pots

Paola: Where they sell plants

Amy: Mosquitoes too, a lot of mosquitoes

Paola: Well it depends, how they take care of it

[00:13:51]

[00:14:05]

[00:15:09]

[00:15:14]

David: This is the health post of Floralia, right

Paola: Yes, this is the health post

[00:15:21]

David: here is the [] recreational and health post

David: Here comes the sick people.

[00:15:28]

Paola: They also have doctors, nurses, []

Amy: Also a health post can be a risk. They said there were some hospitals where ~75% of the doctors and nurses had chikungunya too because there were mosquitoes inside.

[00:15:58]

Paola: Yes, that can be. That's not strange. Look, I know that dengue can be brought by the prison. And it is inside the institution of health that’s inside the prison. And from there I came sick, three days later I was sick with dengue. I brought it from there in the prison because . . .

[00:16:21]

David: In the prison, they kill them there. One leaves with any kind of illness

[00:16:22]

David: Sickness not just health but psychology too.

[00:16:35]

Amy: Plants, plants, tires

David: Me too, remember Paola, one time I entered there in the prison and when I left it gave me a sickness, mortal.

[00:16:51]

David: I came home with a sickness, it sent me to bed

Paola: The prison, no

[00:17:24]

David: Here is another park and the police station. From here to there is closed for security.

[00:17:41]

[00:17:47]

Paola: But this park,

David: Look, no, it looks

Paola: It doesn't look like the other. What about this one to film it?

[00:18:10]

David: the entrance is over there. Look here are the police and over there the drug addicts. Smoking.

Paola: This way are the boys, they fall there and it kills them.

David: Lets go around

Paola: Yes, let’s go around and see if we can enter and film it

[00:18:29]

[00:18:36]

Amy: The kids who are playing are healthy, but those who are sitting there?

Paola: Those are smoking.

[00:18:47]

Paola: I have seen the soccer games they do. Marijuana smokers and police. The two groups.

[00:19:12]

David: here there is no entrance, it is pedestrian only.

[00:19:32]

[00:24:27]

[00:24:51]

[00:24:49]

Paola: We're going to film this park

[00:25:11]

[00:27:32]

[00:30:50]

[00:34:39]

[00:34:42]

Paola: OK, we filmed the park

Amy: [discussing invasions in another area with diarrhea cases without water/energy]

Paola: The invasions from here can be invasions but they have water and energy. They are close

David: Remember that I showed you the post with the cables attached

Amy: They have energy

David: and water

Amy: But toilet they have, with tubing and everything?

[00:36:06]

David: Yes

Paola: Yes

[00:36:35]

[00:37:08]

[00:37:59]

Paola: They are from the basic sanitation, those guys. In the green jackets, they are the ones who do sanitation, who look, who add larvicides in the drains. They are looking what are the risks where are the wells. They revise the pharmacy. But they revise the pharmacies for the drugs, which drugs they have, not for dengue. They have all the various components of the work.

Amy: then it would be interesting to go around with one of them so they could explain.

[00:38:48]

David: No, they are charge of the drain more than anything. This is the truck from the mayor.

[00:39:23]

David: They are going to fix the grass on the field, they are putting on it [] and everything to plant it again.

Amy: The take good care of it.

[00:40:36]

[00:43:12]

[00:43:42]

David: Over there we went to find patients of Paola's in the informal settlement up there. A sick man.

[00:44:06]

[00:46:34]

[00:48:36]

Amy: And this street up there

Paola: That’s the levee of Floralia

David: All mountain. If you want, we can go up there. We can go up there and come down.

[00:48:48]

Amy: Yes

David: Right Paola, for there we can walk in the car

Paola: Yes, up there is not so bad. Safer

Amy: and there they sell the recyclables, right?

Paola: Right.

[00:49:08]

Paola: We had con up there with [], remember?

[00:50:03]

[00:51:09]

Amy: We can visit your patients.

Paola: He lives here

David: Do the follow up

[00:51:23]

Paola: No, but with him we already closed the case. They were going to leave him abandoned but I said no. Why would you leave him alone if he took 11 months of treatment?

Amy: Then he is cured?

Paola: They took the exam and he is resistant.

Amy: Nothing else to do.

Paola: It took him. They are people who . . . I talk with them

[00:51:52]

Paola: Do you see the kinds of houses there are

David: They are only rooms

[00:52:04]

Paola: That’s how it is on the levee

[00:52:10]

David: There is a wagon parking lot

[00:52:17]

David: [] that they can't live and the people live there

Paola: The houses, have

David: There are houses good and others no

[00:52:39]

Paola: Look at this house well assembled

David: That must be the church because it has seats and everything.

Paola: This invasion is more beautiful than the one in Lopez.

David: That on has more drug addiction. If you go there, they will rob you. Terrible.

[00:53:01]

Paola: And displaced African people from the pacific. On the other hand, you don't see that here. Here its more organized, beautiful and calm

[00:53:12]

Amy: In Alfonzo Lopez you see more

Paola: Where we were in Alfonzo Lopez in the levee, you see more, this is more dangerous

David: It is too dangerous for you to go there in car. There they come out armed and they follow you and rob you.

[00:53:35]

Amy: Every time there is a river there are informal settlements?

Paola: Look at the river there. We can go in there. This is the most beautiful street in all the levee.

[00:54:01]

Paola: There is the river

[00:54:29]

Paola: This street is organized, the houses

Amy: Who puts the streets in concrete?

[00:54:48]

David: I imagine that the people did that.

Paola: This is done by the people because these informal settlements are not recognized. If you want, we can get down and film the river.

[00:58:02]

Paola: They urban not recognized.

David: Further up is a lady who is on international TV that she has the Cali Zoo. You haven't heard about her?

[00:58:10]

Paola: A zoo but she is maintaining it with her own resources

David: She has her animals there.

Amy: Why?

Paola: Because she wants. They are rescued animals. She has monkeys, a lion, all are rescued.

Amy: Rescued from where?

Paola: From the circus. The lion it kisses and hugs her

Amy: It’s here in Floralia?

Paola: Yes

David: In this levee on the corner

[01:01:09]

Paola: Look at how the houses are, all in fragile wood.

Amy: But here it is a mix because there are normal houses too

[01:01:10]

Amy: but they aren't poor either. They have their cars, their business

Paola: Yes, this is more organized than the one in Lopez. Lopez is ugly.

David: Lopez is only invasion and pure... there are no businesses

Paola: Yes, but over there you don't see that. And it looks ugly. Here it looks fixed up.

David: In Lopez you can't get out of the car like we did here. You get down and they take everything.

[01:02:09]

[01:04:30]

Amy: Here on the right is something like a hole that fills with water, no?

Paola: I don't know what it is

Amy: Well, I saw that it’s like a hole and when there is rain I am sure it fills with water.

David: You want to look back there.

Amy: no, I already saw that it’s a whole with grass and no more.

[01:06:56]

Amy: all this side without you I can't go because I am not sure what is the best option

Paola: For all these neighborhoods, most of all in the west, over there exist many imaginary lines

David: Over there the problem is the imaginary lines

Paola: Do you know what are imaginary lines?

Amy: Yes, they told us that in Siloe and Terron Colorado, it was difficult but we did it

[01:08:33]

[01:11:36]

Paola: Lets go in and review the park

David: This park is beautiful.

[01:12:02]

David: Look

[01:12:10]

David: Do you want to film?

Amy: It looks normal, just grass no more. No.

[01:12:49]

[01:16:46]

Amy: I was going to ask you if we could use this levee here as an example of what we would find in Alfonzo Lopez in the part that we missed. That it’s the same.

Paola: Yes, in Lopez, it’s the same.

[01:18:00]

Paola: Yes, this levee in Alfonzo Lopez, the difference is that it is more populated, less organized, more dangerous. Right? And a bit dirtier.

David: But the people are the same.

Paola: Due to the higher population, it can be that their residual waters are not adequately managed

[01:18:30]

Paola: That levee, if you want to go you have to go with Jorge

David: That levee, I don't know. It’s dangerous

[01:20:54]

David: The levee of the Cali River because the other side was the Cauca River. This is the Cali River

[01:21:00]

[01:22:54]

Paola: Here is an entrance for the Cali River

[01:23:17]

Paola: We are going to see the Cali River

[01:24:51]

Paola: It’s not ugly

Amy: Yes, very normal

Paola: Look, here we can't see the river

[01:25:47]

David: Ask the lady there if we can go see it.

[01:27:58]

David: Those were sewer tops there. But that water was stagnant water there

Paola: They are sewer tops there. What they do there. That's why they need those tanks because when they make the [] they have to get it wet so for that they put it in these tanks of water.

[01:28:23]

Amy: To make bricks

Paola: Yes, to make the blocks, look at the blocks, the round ones.

[01:29:04]

Paola: Look, it has inside the blocks. They have to get them wet.

[01:29:31]

Paola: Only levee. But look the houses are organized. Here they don't pay for services.

Amy: taxes, nothing

Paola: Nothing. Services they pay because if they have a plaque then the services come to them. But that which is agricultural they pay.

[01:32:02]

David: Look at the river there.

Paola: There we can enter.

[01:32:12]

Paola: The people have their cars. This looks like it’s an office. It has carpet and everything. A factory of furniture.

[01:32:31]

Paola: Here they don't pay taxes. The services they pay cheap because this is social strata one.

[01:32:36]

Paola: Let's go.

[01:32:43]

[01:32:49]

[01:32:56]

[01:33:01]

[01:33:06]

[01:33:12]

[01:33:27]

[01:33:35]

[01:33:37]

[01:33:47]

[01:34:03]

[01:34:11]

[01:35:17]

Paola: Done

David: You saw it over there

Paola: Yes, over there you see it

David: And there you see how it meets the Cauca River?

Paola: No

[01:35:26]

ZOOM0004.MP3 &amp; ZOOM0005.MP3

Neighborhood: Comuna Trece at [00:00:00]

Driver/vector control specialist:

Interviewer: Maribel

Interviewee: Inés

Date: -- --

[00:00:00] I: That there were a lot of mosquitoes. It tells me that here in Comuna Trece there&#39;s

a lot of arborization.

[00:00:07] M: Okay. ((With you, the trees are the ones that produce))

[00:00:18] I: ((The trees and)) and that is black on top. Through here.

[00:00:43] M: Then, this...

[00:00:57] I: At least you touch this at six, seven of the night, and there is a big amount of

mosquitoes.

[00:01:11] I: Before, this was a ((stamped street)).

[00:01:33] I: Before, there was a lot of use of parks. It was with tires from the cars. They made

a lot of ornaments so they kids could play, above all in the schools.

[00:01:47] I: There were a lot of tires. Slowly they started taking that out because they kids

would play and come out with bites from the mosquito.

[00:02:04] I: Here in Comuna Trece, yes it is true, here in Comuna Trece. And another thing

that is important is that in time they do not come to cut the grass. In example, in this part,

that is grass that has not been cut in like three or four months.

[00:02:30] M: And when it rains, those it forms pools of water?

[00:02:31] I: And when it rains, but that is the mosquito emigrates more. It forms pools of

water. Look how that is.

[00:02:39] I: Then, look at all this space that is sector two. Calipso sector two. Calipso is only

one, but is divided in three phases. Calipso one, two, three and five. They are divided by

phases.

[00:02:55] M: And four does not exist?

[00:02:56] I: Not in the moment. I do not know, there&#39;s no four for now. If not five

[00:03:05] I: There is one, two, three and five. This is the park of sector Calipso Three. Its own

community is-- we are above all...

[00:03:29] I: This is the sector three, which is Calipso three. I live in F two. This is the street

seventy-two F alone and he street seventy-two F one.

[00:03:46] I: If we go around this, we will come out in F K. They do not come on time ((... the

mosquitoes))

[00:04:02] I: This space here it&#39;s own community is the one that cleans, organizes and above all

is the new mayor of the Comuna Trece.

[00:04:19] I: He makes them clean. In these days they order to cut this because that (())

[00:04:28] M: In this ones, when it rains, does it form pools of water?

[00:04:30] I: Yes. When it rains, it forms pools of water. And sometimes the current here stays,

at least, where it is, it stays...

[00:04:43] I: There where it is it has water. That is what I tell you. It starts...

[00:04:54] I: This is street seventy-two F one. This is a tree that has not been trimmed in

fourteen and fifteen years. But them, the guy over here trims it more thinly and there is more

maturity.

[00:05:17] I: The woman, like I am telling you, a lot of times they did not come to cut the grass

on time, so she is doing it.

[00:05:26] I: She orders to cut it. And she herself cleans it. Sometimes, she is permanently cuts

it because they sometimes do not come to cut the grass.

[00:05:36] I: Then, this is sector three Calipso. Now we go through here and this is the street

seventy-two F two.

[00:05:55] I: Here is where all the water falls when they clean, it rains, and here is where all

the water falls. And that is what I told you.

[00:06:06] M: All of the blocks have this ditches? That when it rains.

[00:06:08] I: That when it rains the water doesn&#39;t fall. It starts to go out.

[00:06:15] M: Has this ever flooded?

[00:06:18] I: Yes. I have suffered a lot with this block because it has a level how you see it,

((how it is expressed, it divides)) where the water goes in all of the houses.

[00:06:31] I: It went in because thank god, the streets like the twenty-eight E it was paved and

it&#39;s all ready.

[00:07:00]

[00:07:15]

[00:07:19] I: From here towards there, the water goes in. We had to come, like they said,

in canoes.

[00:07:28] I: When you had to take the bus you had to bring-- look, this is what I mean. We

cleaned it and sometimes a man comes, sometimes they pay him to clean, he takes all the

trash out and cleans it, but what about the water.

[00:07:47] I: The water is black.

[00:07:58] I: Here this is new. All of this we made it when the streets were made. This is new.

[00:08:13] I: This is Yira Castro.

[00:08:33] M: But that was also the lagoons area.

[00:08:36] I: Yes, this was all the lagoons area. This used to be, when recently purchased and

populated, this was all lagoon.

[00:08:57] I: And also that is an absence of the community. To not throw trash out. They have

thrown a bottle and the peel of a lemon, or an orange, which produces mosquitoes.

[00:09:23] I: Here they are constructing this street, which was paved.

[00:09:32] M: This was uncovered.

[00:09:34] I: Yes it was uncovered. And now they are fixing it.

[00:09:41] I: This neighborhood was progressed because before this was just bridges and you

passed from side to side.

[00:09:55] I: This street is very good because they are finishing the work. It was not like this

before.

[00:10:05] I: We are in the canal now.

[00:10:30] I: This is the park. Do you see that the park is new but they have water?

[00:10:50] I: Do you see how they parks are?

[00:10:55] I: The grass grows a lot.

[00:11:16]

[00:11:35]

[00:11:40] I: That one has enough water, no?

[00:12:00]

[00:12:30]

[00:13:00]

[00:13:33]

[00:13:44] M: That is Yira Castro.

[00:13:46] I: All of that is Yira Castro.

[00:13:51] It was continually a water damn but before it was mostly dirt with some plants in

the (()).

[00:14:18] All of this is part of ((Estropa)). Is it being made with the construction.

[00:14:27] M: But this was a lagoon before?

[00:14:29] I: Yes, this was a lagoon. Above all, when canal gets full the one&#39;s who suffer the

most are the ones here.

[00:14:41] I: Look, they are already there.

[00:14:59] I: This canal is ducted this part. We are visiting-- the community wants so cover it

but it’s going to stay like that.

[00:15:22] Ineligible.

[00:15:51] I: This was here a (()) of ours. (())

[00:16:24] M: A lot of mosquitoes.

[00:16:28] I: Before, when the fumigation came, you could see them in the floor, but not

anymore.

[00:17:30]

[00:17:41] I: All of this part was houses and small ranches. The water went out there.

[00:17:49] M: The water then-- that got filled with water.

[00:17:52] I: They suffered a lot.

[00:18:07] I: This has improved a lot. This streets in Yira have improved a lot.

[00:18:21] I: And Yira Castro also suffers a lot because of those mosquitos that come out a lot.

[00:18:34] I: All of these streets are new, newly made.

[00:19:00]

[00:19:36] I: This street was also like that. It did not have pavement, but because of a popular

action from the presidents of the communities from the-- And this streets is in a project of

fixing it. All this part from the seventy to seventy-five is uncovered.

[00:20:10] I: When it rains there are a lot of deposits. In the summer you do not see it as much.

[00:20:30]

[00:20:32] I: This street is in the project to put pavement too.

[00:21:00]

[00:21:31] I: Almost all of the alleys have this (()) here.

[00:21:48] I: This is Calipso sector two.

[00:22:00]

[00:22:14] M: This (()) why do they make it. Why to they put them here.

[00:22:18] I: They put them there to cats do not their necessities here.

[00:22:30] M: How much time do does waters remain there?

[00:22:31] I: Or sometimes the dogs that stop to do their necessities. That is there because of

the cats. The bottles are to stop the dogs from raising their legs and pee there.

[00:22:44] M: But what about the stagnant water, when do they change it?

[00:22:48] I: How it is there, it can be seen that they have not changed it at least in eight days

when the water-- when the water is clear and looks clean you can see they changed it recently

but there where it is it has to have at least eight days because the water is green. It is not

fresh it has around eight days.

[00:23:16] I: This is a-- all of this part is Calipso. That is the school to go to the health point.

[00:23:46] I: Yes that is a lack of capacitating people. Of making visits to the neighborhood.

Making meetings. That is what we are, in this community board meeting, we getting out what

health is and we are going to work for that part, the environment.

[00:24:13] I: Not throwing out trash, not letting it out.

[00:24:17] M: When you give education, people... (()).

[00:24:26] I: Ah, but before we, the main of us, when ((EMA)) came to cut grass, we did went

our to clean and sweep. But when the community board does not work, that doesn&#39;t...

[00:24:52] I: The idea is to make campaigns, no.  Those campaigns are very good.

[00:25:00] I: Fifteen days ago, we went out to work on pets. (())...The civilians of Comuna

Trece came and the environment police was here too.

[00:25:30] M: The paved everything, no? And they have finished with the...

[00:25:36] I: Yes, for example this passage, before when we received the houses, it had a green

area. But the green are became that some have one ore three dogs and they took them out so

they could do their necessities and they did not gather the excrements.

[00:25:59] I: Then, each started to pave the way and now there are almost no green areas.

Because they gave this to us with the passages with green areas.

[00:26:22] I: Here we end, the Calipso communal headquarters.

[00:27:00]

[00:27:30]

[00:28:00]

ZOOM0005.MP3

[00:00:00] I: And now we are in that part behind Sorbento, the other one there.

[00:00:13] I: ((They should be fighting, I don’;t know if it the health thing but the doctor had))

ZOOM0003.Wav

Neighborhood: ----

Interviewer: Maribel

Interviewee: Ines

Date: ----

[00:00:03] M:  Good afternoon, Ines. My name is Maribel Murillo Tenorio. I am participating in this exercise of investigation with the University of Kent, the Ladera health network and the Valle de Lily Foundation.

[00:00:19] M: The idea is that we want to know what are your knowledge, representation or impression on Zika, Dengue and Chikungunya problem in the Calipso sector and what adjoins with Yira Castro.

[00:00:36] M: To participate in this investigation, I have to fill this format where you authorize the University of Kent, the Ladera health network and the Valle de Lily Foundation so they can use the information you are supply us in publications, project and in some presentations.

[00:01:02] M: You authorize?

[00:01:03] I: Yes, of course. With pleasure.

[00:01:05] M: Already filled out, here you should fill this out, please. Here and here, where you are saying that you accept to participate in the study.

[00:01:30]

[00:01:47] M: Ines, after accepting to participate in the study and filled out all the formats, accepting in a voluntary manner to be in the investigation by filling in your information, then I would like us to start by you introducing yourself.

[00:02:04] M: Tell us whom you are, what do you do, what you dedicate your time to, and since when do you live in this sector.

[00:02:09] I: Well, to all that are hearing this, my name is Maria Ines Sanchez, I live here in Calipso sector 3, I Amy:  apart of the Comuna Trece. In the moment, I’m a delegate of the community action board. I have participated for twelve years in the E.S.E. de Oriente Network in Calipso of the Comuna Trece.

[00:02:37] I: I am a leader in the network that gives support to elders and I am a part of the foundation of children who are incapacitated.

[00:02:52] I: Also, of woman in families without action and in this moment we are reforming the board of community action, and I am currently the delegate for the year two thousand seventeen, twenty. 2016-20.

[00:03:14] I: They are informing me of the work from the foundation, here my partner from the Valle de Lily Foundation tells me about what the Chikungunya and Dengue are. Because there are a lot of patients in the hospitals, in the CTS's, is because of the mosquito bite.

[00:03:44] I: We have made campaigns in the community Comuna Trece through the secretary of public health; the fumigation and they have collaborated a lot. It always continues to be ((polygraph )) of that animal.

[00:04:05] I: In this moments I have a neighbor in Robles and Yira Castro, here in Calipso in the streets seventy F-one. There's a sprouting of the bite.

[00:04:17] M: Why are there problems of-- in the sector? What is there that allows so many people to have mosquito bites.

[00:04:25] I: Well, there is a problem with what is the mosquito. But we are looking that in the sewer or at times at night, like in example at five-thirty there's is a large quantity. And they come out--they are from the residuals, there are large amounts that come out.

[00:04:52] I: And you see a tree and are full of mosquitoes. In the tires, the plants, the leaves, the bottles for example we have made a trip last year, we did it three months ago. We also did it to visit the people to tell them not to leave laundry rooms with stagnant water.

[00:05:15] I: To not leave them in bottles, or to put them if they have vases or they have tubs, in example gallons, that more than anything because people use those paints tins a lot.

[00:05:29] I: Put the tube upside down so it does stay--another thing to cover the water because here in Comuna, we run out of water when they are working the businesses cut our water two or three days.

[00:05:43] M: But what work are they doing that they have to cut the water?

[00:05:45] I: More than anything, fixing the sewers. And they do, the water is cut because they have to do constructions and annex everything that has to do with the canals.

[00:06:02] I: So they do cuts. In this moments there in street seventy-two with twenty-eight, they are paving. Yesterday we have water cut but that is why people store water and leaves them there.

[00:06:17] M: They store that water and sometimes they leave it stagnant for several days. The larva appears..

[00:06:26] I: That's how the mosquito propagates more.

[00:06:31]  M: What other--Later, you had talked about trees, is there a canal or a lake nearby?

[00:06:38] I: Yes,we here in Yira Castro, by the Troncal, we have a canal that we have suffered from because in the canal you get close at four or five of the afternoon and see thousands and thousands of mosquitoes.

[00:06:55] I: We are now with the secretary of health, who we sent this message to on April ninth so they could fumigate above the canal. Everything that is the canal, you can see thousands and thousands of mosquitoes.

[00:07:11] I: Then, we want them to the favor and they fumigate. We had talked to the official for him to go at five in the morning and in the afternoon at six, because that's when the mosquitoes come out.

[00:07:31] I: And in the part at the bottom, towards the invasion, they have commented to us that there are a large number of kids that have outbreaks. (( A lot of them have bites, and what they are doing is putting their "tornillos".

[00:07:48] I: Last  time, we had commented to see if they gave us some tornillos to the, above all, pregnant women, and they gave out more than a thousand tornillos for the mosquitoes for the pregnant women.

[00:08:06] M: Do you know, in accordance to your experience, you thought that was a solution for the problem? Giving out tornillos against the vector?

[00:08:18] I: It gives a little of protection but really no because  during the whole day the mosquitos appear but above all in the night.

[00:08:32] M:  So you told us that the mosquito appear during five thirty in the afternoon in those areas where they are constructing, fixing the sewers because residual waters gets accumulated and towards the side where the canal where they are fixing it.

[00:08:50] M: What solutions do you think they are for the canal and for when they are constructing or fixing the way...

[00:08:58] I: Well I as committee-- I am apart of the committee for plan making here in Comuna Trece. We came to agreement so they could cover the canal.

[00:09:10] I: And they are doing that. We are going to see what we can achieve now with the new major with the process that they do in the plan making community to see if they are really going to cover it or all of the canal.

[00:09:28] I: For the Simon Bolivar avenue from the seventy to the Pondaje and Comunero two and towards the Nueva Ilusion, all of that canal over there.

[00:09:39] I: Well we have seen that is good-- above all when it rain the canal overflows and for us is a solutions to cover it.

[00:09:54] M: When it rains, the canal overflows?

[00:09:56] I: It overflows.

[00:09:57] M: And in the drought, what is the problem?

[00:10:00] I: In the drought, the problem is still there if the canal remains with water. A lot of water. It stays with a lot of water and you know that where there is still water , the mosquitoes are there. There the larvae are born and they reproduce.

[00:10:16] M: The solution is to cover it?

[00:10:18] I: To cover the canal.

[00:10:19] M: What other solutions do you think that should appear for the subject of the mosquitoes here.

[00:10:25] I: Fumigating.

[00:10:26] M: Fumigating

[00:10:28] I: Fumigating.

[00:10:29] M: You talk about how sometimes in houses there’s still waters. You talk about tires.

[00:10:36] I: Stagnant. Tires, bottles...

[00:10:39] M: Whom do does tires belong to?

[00:10:41] I: Well in example, in the parks, for example in the part below Comunero, there are tires that they leave there or are installed as games.

[00:10:54] I: And you see that and you see the bellow the tire and even if the children are playing to jump, those tires, you touch them and the mosquitoes come out. The mosquitoes come out.

[00:11:08] I: And for me  also another way that you can control it in the residuals is to clean them constantly. Which that-- they throw a lot of trash and grease...

[00:11:27] M: In that sewer.

[00:11:31] I: In that sewer. In those residuals that recollect when it rains and that becomes stagnant.

[00:11:38] M: And why do the parks have tires?

[00:11:40] I: Generally, in the parks in the bottom they put tires. In this park here in Calipso they put some tires, but really when it rains that becomes stagnant-- the water that is there still and the it is better to not have those tires.

[00:12:07] I: In the parks, the children for example still--in sector two there is still some tires. They are a small quantity because people have been taking them out.

[00:12:19] I: When you go and see the water is stagnant there and it lasts days there.

[00:12:27] M: So that was the resource that was found so that the kids could play?

[00:12:29] I: Yes.

[00:12:29] M: But you have noticed that they need to be removed because that is where the mosquitoes are.

[00:12:33] I: Yes.

[00:12:35] M: Tell me how long have you been her? How did you get to this neighborhood.

[00:12:39] I: To this neighborhood, I got here when housing started and I was looking a house.

[00:12:48] M: Where did you came from?

[00:12:48] I: I come from a apartment in Cauca. I am apart of the ((Cabildo Nasa)), in search of a job and for help for my family, my parents.

[00:13:02] I: And I worked here in Cali for like eleven years here in the fourteenth.

[00:13:06] M: But you got here from what age?

[00:13:08] I: I got here when I was eighteen. I have my identification, I had just gotten it. Some aunts brought me here. I have nine aunts here in Cali. And they brought me and I started washing clothes and waited until I had a job so I presented myself in the fourteenth and I stayed there working.

[00:13:31] I: I worked around eleven years with them and I retired as independent because I am a Dressmaker. I am a professor or confection and cutting. And I teach free in the foundations and in the communal centers and the people who ask me for a certain cut I teach them completely free.

[00:13:52] I: I am participate in a lot of free things and I do it willingly because  I like working with the community and above everything with kids and the elderly.

[00:14:02] M: You say you were looking for a place to live.

[00:14:06] I: Yes I was looking for somewhere to live and I liked it. I visited this housing and I liked it because they are big. And since the business offered me an opportunity to buy something for my future and that's how it went.

[00:14:24] M: Look for a place, you got to this neighborhood.

[00:14:27] I: Yes, this neighborhood.

[00:14:28] M: And they had a promotion, what happened?

[00:14:31] I: In reality, they had an offer, it was a good offer, and with my savings I had possibilities because I have been a woman that since small age I had savings.

[00:14:45] I: And then, seeing the opportunity that I could actually pay the installments. Then  I got into the habit that I could actually buy my house and I am currently living there.

[00:14:57] M: Then that house--what they sold you was the land or the house.

[00:15:00] I: No, with the house

[00:15:01] M: Who was the seller, the town or who?

[00:15:03] I: No, this was through "Mi vivienda" and I got in to buy it with fifteen years to pay for it.

[00:15:13] M: The house as it is.

[00:15:13] I: The house as it is.

[00:15:15] M: And you that know the history of this neighborhood and how it formed...

[00:15:17] I: Well, this neighborhood was very populated with a lot of houses. But with time it kept populating more and more.

[00:15:30] I: This neighborhood is a pretty, beautiful one and more calm. The community has prepared a lot.

[00:15:39] I: We have grown up here. For example my son has twenty-five years and he is a very well formed boy, he is a professor in--

[00:15:46] M: So you have lived here since when?

[00:15:48] I: Here in Calipso, it has been thirty years.

[00:15:54] M: Thirty years here in Calipso.

[00:15:55] I: I got married in the year (((nineteen eighty five)) and in that same year  bought the house.

[00:16:01] M: Oh that was with a house, a car and a scholarship?

[00:16:03] I: House, a car and a scholarship, yes.

[00:16:06] M: And a husband on board.

[00:16:07] I: And a husband on board.

[00:16:09] M : Does your husband still live?

[00:16:10] I: Yes, my husband still lives. My husband's name is ___ and he is fifty-four years old. I am older than him. I have fifty-nine years and my son is twenty-five. We are three. And I also lived with my father, but he passed away five years ago.

[00:16:26] I:I brought him over here. And we were living with him until God took him away. And Calipso is a great neighborhood to live in. For everything, it is a very tranquil neighborhood.

[00:16:42] I: Well, like any neighborhood there is a lot of insecurities but it is very good to live here. I would recommend.

[00:16:50] M: One question, you have heard of cases of people affected by the virus. What have you heard about how they cure they virus? What have they tried?

[00:17:08] I: Well, of the virus I know a lot because I have gone and visit many patients and I have gone to visit them and see them when I was with the Oriente Network. We were visiting a lot. Well, they always give them acetaminophen and a lot of liquids.

[00:17:32] M: And what is the use for taking acetaminophen and a lot of liquids.

[00:17:35] I: Well, the acetaminophen is to calm the discomfort, they always give them that for the discomfort.

[00:17:43] I: And a lot of liquids are because the body needs it-- that liquid. They always tell them to take a lot of juice, in example, juices that are not only water but fruit juices for that gives them proteins and vitamins because since that ((dilates)) them a lot, it produces a lot of fever, outbreaks, they have a lot of rash.

[00:18:17] I: A lot of discomfort and it gives them fevers.

[00:18:18] M: That is the use for acetaminophen.

[00:18:20] I: That is the use of the acetaminophen.

[00:18:22] M: Do you know another remedy that they use to control or slow down..

[00:18:29] I: Profane is more than anything for the pain, because when the mosquitoes bites. For example, that happened to me the other day and they took me to the emergency room and they took that remedy to the clinic.

[00:18:46] I: My feet swelled and my junctures hurt, which start with a discomfort. And when you go to stand up you can't because the junctures hurt. Your ankles hurt, the knees, arms...

[00:19:00] M: Which one did you get? Dengue, Zika or Chikungunya.

[00:19:09] I: I think it was Dengue because they gave me the treatment, because I have hypertension and diabetes. They gave me that, the acetaminophen and they told me to drink a lot of liquids. That's what they told me to drink.

[00:19:22] M: Did they take blood tests?

[00:19:23] I: Yes, they took a blood test. The Chikungunya bit my son, but that's what they always tell us, that it was the Chikungunya.

[00:19:31] M: And what did the doctor explained to you when they took you? It should go out or did they take a test? They did the test and what did they tell you? Is Dengue? Did he tell you directly

[00:19:40] I: He told me that is was Dengue. Then, I told him that if it was Dengue what was the process I had to do, or is it really harmful or what.

[00:19:50] I: He said no that is mild. That is a pain, discomfort that you get but it goes away in four or five days but it lasted eighteen days. Eighteen days.

[00:20:04] I: With-- not that much discomfort if not the junctures, which is like they would have given me---or would have gotten up a mountain or just walking a lot. All the junctures, specially the toes.

[00:20:16] M: And did you follow the (( )) remedy or did you use homemade remedies.\

[00:20:20] I: No, I only used-- Since the doctor that they could not give me a lot of medicaments because of my problem with diabetes and since I had hypertension I had to take the medication that they recommended.

[00:20:35] I: And the Chikungunya. The bite of the little animal.

[00:20:37] M: Have you heard of people who suddenly have been sick because of the virus and have not gone to the doctor?

[00:20:45] I: Yes, there's some people that only go two or three times or only one time and they know what they have to take but they almost do not go.

[00:20:55] M: And what do they say they take?

[00:20:57] I: They take a lot of things. They take lemonade, herbs.

[00:21:03] M: What herbs?

[00:21:04] I: For example, the lemongrass, the aguapanela with lemongrass. They always take herbs. They say that they take a certain thing and that happened.

[00:21:12] M: Then there's people that have been sick, have not consulted the doctor and have taken homemade remedies and the sickness has passed.

[00:21:20] I: It has passed.

[00:21:23] M: Why are there people that knowing they have that do not come to the doctor?

[00:21:27] I: Because a lot of times they do not take care of themselves or don't like it or someone else tells them "take this" and then they prescribe that.

[00:21:36] I: But that is very bad because one must always consult a doctor and know that that is a guarantee as a patient when your going to visit the doctor because he has the knowledge of what you really have and that's why the test are for.

[00:21:54] I: The frame of the Dengue virus is very difficult because they took a large amount of blood out of me, and they even asked why are taking so much blood and is because they make a lot of different tests , a lot of processes and types of tests.

[00:22:07] I: First of all to know what is it really.

[00:22:12] M: And if your family...

[00:22:14] I: From my family, the three of us had it. My husband, some of the people from my block have been like that.

[00:22:25] M: Is there a sewer that might be stagnant?

[00:22:29] I: We only have one five meters from here, the canal. Half a block from here we have the canal.

[00:22:33] M: The canal.

[00:22:36] I: The canal in (( Troncal)).

[00:22:37] M: The those mosquitoes that were breed in the canal are the ones (( I left five letters)).

[00:22:42] I: But I am sure of something. Because were are even making meetings in these days because of so many mosquitoes, because I say that the mosquitoes come from the surroundings.

[00:22:59] I: Like from the sides, from those parts that they come to the city.

[00:23:07] M: From what neighborhoods, tell me.

[00:23:08] I: Like in example from-- we are living in Comuna Trece. I say that you go to Comuna twenty-one , in Pisamos. You cannot open your mouth. You have to cover yourself because that is the biggest mosquito ever.

[00:23:29] M: But did it happen there?

[00:23:30] I: It did not happen?

[00:23:34] M: The large parts of the cases are in these sectors.

[00:23:36] I: Well, I blame the canals a lot. Because that canal, I would like for us to pass in the truck because it is full of dirty water. Go look at it and take a picture and you will see the amount of mosquitoes there.

[00:23:55] I: They are thousands. They are small and they are big. And you see some mosquitoes and their legs are white. Those white legs.

[00:24:02] M: So those are the mosquitoes, then the canal is the cause (( the gives more)).

[00:24:11] I: Yes, and because of leaving a lot of stagnant waters.

[00:24:15] M: What can we do to prevent this other than fumigating, covering the canal, talking to people...What other actions do we need to take in order to control this better? What other actions?

[00:24:30] I: Well I say that if there was a implementation to add, I don’t know, that liquid when they fumigate-- There's so many things, so many chemicals. To see if they reinforcing it a little bit. Because when they fumigate you can't feel it. You can't.

[00:24:57] M: The smell

[00:24:58] I: You can't feel the smell.

[00:25:00] M: It used to before but not any more?

[00:25:00] I: It used to. I say that it used too, we even covered ourselves and the birdies or the animals that one had in their houses, like pets because the smell was strong.

[00:25:13] M: And there were not that many people with Dengue before?

[00:25:17] I: No, there wasn't before. Now there's a lot. Because you feel the fumigation, because someone from the secretary came and told me, "You were the person who send the right of petition to do it". And I said yes.

[00:25:34] I: He then told me to go up ((and put a mouth cover )) and I took it off and it was not strong.

[00:25:42] M: And why do you think that it does not smell so strong?

[00:25:46] I: They decreased it or it may be another type of medication that was applied. I don't know. And before you could put a sheet in the terrace or a curtain and you put it on the floor and you could see the mosquitoes falling.

[00:26:06] I: But now you do that and it doesn't fall.

[00:26:12] M: So it stopped being potent? And it stopped being strong.

[00:26:14] I: Yes.

[00:26:16] M: And what you think is that it has decreased?

[00:26:18] I: They have decreased the chemicals and we, you see, in the floor, even in your arms you could see them falling. Or in the patio.

[00:26:31] I: If the patio was white...for example my brother in law patio is white and he--you see the mosquitoes. You throw a sheet over it or white curtain and everything fell on it. But not any more.

[00:26:46] I: Now you fumigate and look at your terrace or patio and nothing. You do not see a dead mosquito. Before yes. It had a strong smell. Before, you achieved that your grandfathers-- Oh, who told us that they were going to fumigate this day that time we are going to fumigate.

[00:27:08] I: But not now.

[00:27:08] M: And what do you think about the fumigation being early in the day?

[00:27:13] I: I like it a lot, and anyone could tell you that. Is because at five thirty into he afternoon they start to migrate and at five in the morning they are migrating to their holes that they have.

[00:27:27] I: For-- they always get in the trees, in the branches or in the sewers.

[00:27:35] M: So they need to fumigate at that time.

[00:27:38] I: For me, I solicited that they did it at five in the morning and at six of the afternoon. That at six in the afternoon they start coming here. At seven in the night that they start to go out at six of the afternoon is a good thing.

[00:27:55] M: They are going out of work at six.

[00:27:57] I: They go out to devour blood. To suck blood.

[00:28:01] M: Well, something else that you want to comment on?

[00:28:05] I: No, that you are welcomed every time that you want to come and do problem. And if there is a fumigation in Comuna Trece, it would be a honor to have you here.

[00:28:16] M: Then preventing and fumigating, education in the houses, correct? And cover the canal.

[00:28:25] I: Yes, covering the canal.

[00:28:26] M: For you guys, the cause of the mosquitos is in relation to the canal over there. That used to be a lagoon.

[00:28:32] I: Yes a lagoon. And that comes from above there. And now they are making some arrangements and we are going to see if that--what is happening with those arrangements.

[00:28:45] M: Ines, if it used to be a lagoon, why did it become a canal.

[00:28:50] I: Well because, they put cement in the sides so it divides the water because when it used to rain the water expanded everywhere. And now it has changed a lot because they fixed it. Furthermore, they are still fixing it right now.

[00:29:20] M: Do you want to show me the place where the mosquitoes come out?

[00:29:24] I: Where the mosquitoes are? Yes, with pleasure.

File: C:\Users\Amy\Google Drive\Kent\james\dissertation\chkv and dengue\data\video

data\january 27\audio\ZOOM0001.WAV

Associated videos: C:\Users\Amy\Google Drive\Kent\james\dissertation\chkv and

dengue\data\video data\january 27

Neighborhood: Napoles

Driver/vector control specialist: David

Interviewer: Amy

Date: January 27, 2016

[00:00:00]

[00:01:09]

[00:01:21]

[00:01:32]

[00:01:49]

[00:01:53]

[00:02:17]

[00:02:24]

[00:02:36]

[00:02:38]

[00:03:54]

Amy:  then we are ready. We are here in Napoles.

David: Yes, Napoles.

[00:04:07]

[00:04:15]

Amy:  and today is January 27 and we are again in drought.

David: yes, this is the neighborhood Napoles.

[00:04:37]

David: in the south of Cali.

[00:04:44]

Amy:  but still in the west, no? all in the west.

David: mhm.

[00:04:57]

David: Napoles has a canal of rainwater that passes by the batallion.

[00:05:03]

David: and that would create maybe the focal point for the vectores.

Amy:  I was thinking too that the battallion could be a risk point due to all of the soliders that are

traveling to all parts.

David: yes, of course, becuase they travel to all parts of colombia and they come back and they

bring it.

Amy:  yes, that.

[00:05:29]

Amy:  I think we saw that in Melendez too. Melendez has dengue but it doesn&#39;t have a reason to

have dengue except for the battalion.

[00:05:39]

David: of course, they go to even the pacific parts sometimes. They can bring even malaria.

[00:05:46]

Amy:  yes.

[00:05:49]

David: the yellow fever they have there too, right? In the coast.

Amy:  but you don&#39;t have that in colombia?

David: in the pacific, yes.

[00:06:32]

[00:06:46]

[00:08:23]

[00:08:51]

Amy:  The quartel de Napole. What is it?

David: What is it? That&#39;s the Battalion.

[00:09:15]

[00:09:28]

[00:09:43]

[00:09:54]

[00:11:03]

David: This hill you see in front. There is the dyke that they put to do surveillance outside.

[00:11:38]

Amy:  then they said it won&#39;t rain until August, right?

David: oh yes, no.

[00:12:11]

David: Look at the canal that is on this side.

Amy:  its dry, no?

[00:12:24]

Amy:  I never saw any of the flooding in Cali. It has always been in drought since I have been

here.

David: you don&#39;t know the canals?

Amy:  No, I never saw them. One night it rained hard but only some streets had water, no more.

In the rest of the time, dry. Maybe I brought the drought.

[00:12:53]

[00:13:29]

[00:13:23]

[00:14:20]

Amy:  There is nobody in the park.

[00:14:27]

[00:14:51]

[00:14:55]

Amy:  Its a very organized neighborhood.

David: hm?

Amy:  Its an organized neighborhood. Its clean.

[00:15:08]

[00:15:34]

[00:16:12]

David: Napoles is a neighborhood of social strata 4.

Amy:  yes?

David: yes, in between 3 and 4.

[00:16:21]

Amy:  its organized and clean.

[00:16:27]

David: the only thing it has is the canal on that side.

Amy:  the canal and the battalion.

[00:16:32]

[00:16:58]

[00:17:08]

[00:18:13]

Amy:  but yes there are some holes in the street that can be like puddles.

[00:18:25]

[00:18:51]

[00:19:21]

[00:19:54]

David: here in this district 16 there are only soldiers who have a bachelor degree. Those that

don&#39;t have bachelor degrees, the send them to the bush.

Amy:  Bachelor is what, highschool?

David: yes

Amy:  and bush is what country?

David: yes, where the guerilla fighting is.

Amy:  that&#39;s not fair. THey should give them highschool instead of sending them to fight the

guerilla.

[00:20:35]

David: this is the psychiatric hospital.

Amy:  that you see from the 5th street, right?

David: yes.

[00:20:45]

David: I came to fumigate here. In all the rooms.

Amy:  in the hospital rooms?

David: yes.

Amy:  and the patients, where were they?

David: there, there.

[00:21:01]

David: I had to fumigate over all of them.

Amy:  for what, ants?

[00:21:06]

David: ants, roaches. This is a preventive control.

[00:21:11]

[00:21:54]

[00:22:53]

[00:23:59]

[00:24:23]

[00:25:04]

[00:25:24]

David: ok, amy I think here we have finished.

Amy:  ok, then lets go to Morchial de comfandi.

[00:25:48]

[00:26:26]

[00:27:24]

[00:28:32]

File: C:\Users\Amy\Google Drive\Kent\james\dissertation\chkv and dengue\data\video data\january 27\audio\ZOOM0002.WAV

Associated videos: C:\Users\Amy\Google Drive\Kent\james\dissertation\chkv and dengue\data\video data\january 27

Neighborhood: Morichal de Comfandi

Driver/vector control specialist: David

Interviewer: Amy

Date: January 27, 2016

[00:00:00]

David: the neighborhood of Morichal de Comfandi- the houses were built about 10-12 years ago. They did it for social interest from a pension account called Comfandi. So its called Morichal de comfandi.

Amy:  ok, then its a new neighborhood.

David: yes. Its a neighborhood of all laborers, workers of companies that through this savings program saved the initial payment and they pay the rest of 10-15 years.

Amy:  very good

[00:00:41]

David: and they have their own house.

Amy:  its very dry here.

[00:00:55]

David: its on the other side of Ciudad Cordoba that was all soccer fields, do you remember?

Amy:  fields

David: soccer fields, yes. This is on the other side of Ciudad Cordoba.

Amy:  and we are going to see the canal here too.

David: over there is a dyke but I don't know what is on the other side of it.

[00:01:26]

Amy:  how is the danger in this neighborhood, high, medium, low?

David: the danger here is medium becuase the side over there by the trash dump has always been an invasion.

[00:01:43]

David: the people who make there shacks over there- all people of low class who are robbers, drug users, drug addicts.

[00:01:57]

Amy:  and that invasion is next to this neighborhood or is part of this neighborhood?

David: no, no

Amy:  behind the neighborhood.

David: No, at least, this kids with this look here are from the other side.

[00:02:10]

Amy:  for the clothes?

David: no, its stigmatizing one but uh the african population is always from over there. from the dyke zones, the invasion.

[00:02:24]

Amy: and we haven't seen this invasion here?

David: the invasion

Amy:  the one here

David: no

Amy:  and it's not safe to enter?

David: no.

[00:02:39]

David: no, becuase they are more than anything recyclers.

Amy:  ok, ok

[00:02:47]

Amy:  Yes, that's a danger for the neighborhood to have dengue here too.

[00:02:51]

David: yes, as they have always had the trash dump there. All the people from the streets go to see what they can take from there.

[00:03:01]

Amy:  and the trash company is from the city or from a company of Cali.

David: no, it was from the city. It was called [la barra].

[00:03:09]

Amy:  and they let everyone enter there to take what they wanted because that is a service for the city.

David: mhm.

[00:03:16]

David: but as they closed it and sent it, they sent it to be done with all the norms and all the rules that it needs to function. But here it didn't work that way. Here it was, pshoo, throw it to the pile and no more.

[00:03:30]

David: and the people from the street started to go there rummage and take out the things to recycle and everything.

[00:03:37]

Amy:  to sell it in the

David: yes, the cardboard, the scraps, everything. on the other hand, over there, where it is working, they have machines and carousels.

[00:03:46]

Amy:  This other company that is working, where is it?

David: in viges, they took it out of the city. but what happened is that this already has a very big contamination.

[00:04:00]

Amy:  and the city left it, this one.

David: For that reason, for the high contamination and they know that it wasn't giving [] and so it was going to contaminate more. From there they started to leave from the lot designed for what it was. They took it out.

[00:04:22]

Amy:  difficult to control.

[00:04:30]

Amy:  the houses here look like they are new or they have 12 years but that they don't have much maintenance.

[00:04:46]

David: becuase it is of people of class middle and low.

[00:04:50]

David: not enough money to improve it.

[00:05:04]

David: It always has to be like that in time.

[00:05:13]

[00:05:39]

[00:05:47]

[00:05:56]

[00:06:07]

Amy:  what happened here? This is an abandoned lot?

David: yes.

[00:06:13]

[00:06:59]

[00:09:02]

[00:09:39]

[00:11:00]

[00:11:20]

[00:11:35]

[00:12:13]

[00:12:37]

Amy:  the streets are good here.

[00:12:49]

David: and on the side where we finish the morichal, there is another neighborhood called vallado, that we are going to see, this vallado has many ugly parts, many robbers, and some parts that have witches. There are some that enter into houses, to mess with married men.

[00:13:21]

Amy:  the witches enter the houses of the people from here? How? Without a key?

David: hm?

[00:13:32]

[00:14:36]

[00:15:27]

[00:16:22]

[00:16:41]

[00:18:45]

[00:19:11]

Amy:  here, what is it?

David: it is a circus

Amy:  and the horses are part of the circus?

David: yes, but it looks for pirated to me, very hidden. That is prohibited.

[00:19:44]

[00:20:15]

[00:21:52]

File: C:\Users\Amy\Google Drive\Kent\james\dissertation\chkv and dengue\data\video data\january 27\audio\ZOOM0003.WAV

Associated videos: C:\Users\Amy\Google Drive\Kent\james\dissertation\chkv and dengue\data\video data\january 27

Neighborhood: El Vergel

Driver/vector control specialist: David

Interviewer: Amy

Date: January 27, 2016

[00:00:00]

[00:00:10]

[00:00:56]

David: its ugly, right?

Amy:  Yes, there is a lot of trash and the street is not in very good condition.

[00:01:08]

[00:01:14]

[00:01:23]

Amy:  there is a big hole there, can we pass?

David: no.

[00:01:35]

Amy:  the laguna

[00:01:51]

David: el retiro is on that side, of which we have fear for the danger.

[00:02:07]

[00:02:12]

David: they are small but they are dangerous.

Amy:  el retiro, you are telling me is dangerous?

David: yes

Amy:  and el Vergel, how is it?

David: El Vergel is next to El Retiro but El Retiro is more dangerous than El Vergel.

[00:02:44]

Amy:  ok.

[00:02:49]

[00:03:14]

David: look at this house

[00:03:17]

David: and these dead ends here are dangerous.

Amy:  around 6, 7, 8 at night.

David: no, you can't come here at night. They will stop you and come out with arms and rob you and everything.

Amy:  just for the money?

David: for whatever one has. If you have a cell phone, whatever you have the rob. boys there.

[00:03:51]

Amy:  but it's not for territories?

David: he is hiding something there, an arm or something.

David: yes, here it also micro-trafficking for drugs.

[00:04:07]

David: ah, here is where we weren't going to enter, it is bad.

[00:04:11]

David: el retiro is over there, very very very dangerous

[00:04:25]

Amy:  the fire that is over there, that is the trash they are burning?

David: yes.

[00:04:39]

Amy:  and the boys are recyclers or what?

David: no, they are all drug addicts here in this sector.

[00:05:01]

[00:05:11]

Amy:  the trash that they leave out here is for the trash truck to pick up?

David: yes

[00:05:32]

[00:05:53]

David: a small neighborhood but the problem is- at night its tranquil, but at night. the boys look at night to go out.

[00:06:05]

David: to rob, to consume, all at night.

[00:06:15]

[00:06:24]

[00:06:37]

[00:07:15]

[00:07:29]

[00:07:44]

[00:08:26]

[00:09:21]

[00:10:08]

[00:10:46]

[00:12:03]

[00:12:40]

[00:13:49]

David: look at this shack.

Amy:  there are people that live there?

David: yes, there is clothes hanging there.

[00:14:09]

[00:14:38]

[00:15:04]

[00:15:34]

[00:16:17]

Amy:  and this neighborhood has- doesn't have the canal inside but it has one around and it has a dyke.

[00:16:35]

[00:17:33]

[00:18:37]

[00:19:40]

[00:20:22]

[00:20:55]

[00:21:01]

David: santa helena isn't on your list?

Amy:  no

David: And Santa Helena gets lots of dengue.

Amy:  maybe its because they aren't reporting. Its like Charco azul. They told me that in charco azul there is lots of dengue but they prefer to go to the witch doctor rather than report it to the secretary.

[00:21:26]

David: yes, there are lots of people who go to the pharmacy for the pain and no more. they are not diagnosed.

Amy:  yes, there are many people who are not going to be diagnosed because there is no treatment or anything so why are they going...

David: yes, because they feel bad so they say this I can cure from the pharmacy

[00:21:58]

Amy:  yes, this I understand. I don't like the doctor either. its expensive, it takes a lot of time.

David: yes, because the doctor don't send them for the exam or anything. they just say, take the acetaminophen and ask for an appointment on this day. and they come back on that day and if they are the same then they let a lot of time pass and they don't diagnose it. they don't say "you have this. with this exam it came back with this thing." They put them to wait and to wait to endure the sickness.

[00:22:27]

Amy:  yes, because neither can they do anything for the dengue nor the chikungunya. they can't do anything

[00:22:32]

David: take the diagnosis and no more. take the acetaminophen.

[00:22:39]

Amy:  at least with malaria they go to the doctor for the drugs that they have but the rest no.

Amy:  then I turn off here, we finished El Vergel?

[00:22:52]

David: yes.

File: C:\Users\Amy\Google Drive\Kent\james\dissertation\chkv and dengue\data\video data\january 28\audio\ZOOM0001.WAV

Associated videos: C:\Users\Amy\Google Drive\Kent\james\dissertation\chkv and dengue\data\video data\january 28

Neighborhood: Cristobal Colon

Driver/vector control specialist: David

Interviewer: Amy

Date: January 29, 2016

[00:00:00]

Amy:  ok, we are in Cristobal Colon, January 29 with David. Let's see what there is. Do you know why there is so much dengue here?

[00:00:17]

David: really, they have also a canal of rain water but there in the corner.

Amy:  in the corner, ok.

[00:00:31]

Amy: Interesting that they are doing a stronger control now when there is no rain. It is a risk or no?

[00:00:49]

David: its probable that it serves more becuase there are less foci so attacking those should be better to diminish the activity.

[00:01:02]

Amy:  and you have here aedes aggypti not albopictus, no

David: what?

Amy:  there are two types of mosquito that transmit the dengue, aedes albo

David: yes, the aedes aegypti

Amy:  and the other is albopictus

[00:01:17]

Amy:  you have or just the agypti

David: agypti they call anopheles here

Amy:  agypti is the one with white legs and the other

David: the anopheles is similar to him.

[00:01:43]

Amy:  the anopheles, no. that is for malaria.

David: but there is another that is for dengue too, no?

Amy:  yes. ablopictus

[00:01:47]

David: albopictus its called.

Amy:  albopictus, yes. This one has on its back a shape

David: some marks like a diomond

Amy:  of this form, white.

David: i think the one is the aedes agypti.

Amy:  ok.

David: that is the canal that I told you. it was of rain water but i think in this time period the rainwater one shoudl be dry.

Amy:  yes, its dry.

Amy:  No, I ask.

David: no look

Amy:  yes, there is something moving. Yes, its moving.

Amy:  its becuase the albopictus they like the dirty water and the agypti they like the clean water.

[00:02:30]

Amy:  then yes []

[00:02:37]

David: I think this is the most critical part of Colon becuase in the rest of the neighborhood there is no canal, there is nothing. and the neighborhood is very well permeated.

Amy:  permeated?

David: yes, it doesn't have hardly any holes.

[00:02:55]

[00:03:16]

David: the 38 is where the canal is.

[00:03:21]

Amy:  its small. very small.

David: yes, its small. its not big.

[00:03:41]

[00:04:08]

[00:04:47]

[00:05:04]

David: from here to there is the neighborhood Santa Elena. Cristobal Colon goes till here.

[00:05:13]

Amy:  and Santa Helena doesn't have a canal?

David: Santa Helena has a canal. a canal of rainwater. What happens is that as santa helena is so commercial, if there are cases there, everybody goes to their house and in their house they look for remedy and they don't report it maybe, no?

[00:05:31]

Amy:  yes, if there are cases, there are no cases there.

[00:05:37]

[00:06:07]

[00:06:51]

[00:07:14]

[00:07:24]

[00:07:33]

[00:08:01]

[00:08:37]

[00:09:18]

[00:10:55]

[00:12:02]

[00:12:21]

[00:12:43]

David: cristobal colon is a neighborhood that is social strata 3 or in these parts like the comercial centers, they are social strata 4.

[00:12:53]

David: they charge a little bit more but because they live in a comercial area.

Amy:  then in one neighborhood, there can be many levels.

David: yes

[00:13:03]

Amy:  i didn't know that becuase in the database that I have, each neighborhood has one level

David: one level. yes. but its that they can charge more in the comercial areas. For example, in the center, there are parts where they charge up to strata 6. and with that comes the more expesnive services. there are some services where they collect the trash up to two times a day

Amy:  two times!

David: mhm, they pass in the morning to take all trash left out at night or the pass at night to take all the trash taken out in the day.

[00:13:42]

Amy:  and imagine, one block over and they don't collect anything in the week.

David: yes.

Amy:  then, what does it serve.

[00:13:54]

David: yes, but as there is so much trash in the center, the charge them for that. they charge them for the commercial zone, that there they sell.

[00:14:08]

[00:14:15]

[00:14:49]

[00:15:02]

David: this is the health center of Cristobal Colon. Here is where they report all of the cases, i think. they come the patients with the dengue and chikungunya and now the zika.

Amy:  yes, now the zika too.

[00:15:22]

[00:15:52]

[00:16:20]

Amy:  its a beatufiul neighborhood. it looks very organized, with families, with houses, not too new but beautiful.

[00:16:52]

[00:17:01]

David: this is the highschool of Cristobal Colon. the parish and on the other side the hghschool. This is a Franciscan high school

[00:17:13]

Amy:  private.

David: mhm. although these high schools these days- enter the students who don't have resources. they sponsor their studies.

[00:17:30]

Amy:  that's good.

[00:17:41]

[00:17:53]

David: becuase before there was discrimination. those who didn't have money couldn't study there.

Amy:  and the public schools, how are they?

David: well the public schools have gotten much better the eduction.

[00:18:06]

David: what happens now is that they require that all the highschools have a certain level so that they sponsor them so they pay them.

[00:18:19]

Amy:  the goverment.

David: yes, if they don't have an academic level, the close them. the state sends to do tests every year. Some tests of knowledge, called the tests of know.

[00:18:33]

[00:18:29]

[00:19:16]

[00:20:08]

[00:20:37]

[00:20:53]

[00:21:25]

[00:21:39]

[00:22:24]

[00:23:03]

[00:23:16]

[00:23:30]

David: the house is abandoned.

[00:23:37]

[00:24:12]

[00:24:29]

David: yes, i think that cristobal colon the only thing it has like a canal is this.

[00:24:43]

[00:24:54]

[00:25:16]

[00:25:30]

[00:25:59]

[00:26:19]

David: santa helena has a canal over there, look.

Amy:  at the end.

[00:26:33]

David: [] black on the curve. behind that is the canal.

Amy:  ok

[00:26:39]

Amy:  in the storm drains, the mosquitoes can breed there too, or no?

David: well I think so. if they are blocked for a long time. yes.

[00:26:59]

David: [ ] the water. they always look for the water.

[00:27:15]

Amy:  i was reading that if they do control of the biggest breeding sites

David: the breeding sites

Amy:  yes, the breeding sites, if they do control of the biggest ones then the mosquitoes disperse and look all over where to put its eggs. then they say it doesn't work to just focus on breeding sites but you have to do all the area.

[00:28:00]

David: if you attack one area, the miggrate. it is a migratory plague.

[00:28:02]

David: they migrate to another area and the do another area. ok, i think here we finished colon.

[00:28:10]

File: C:\Users\Amy\Google Drive\Kent\james\dissertation\chkv and dengue\data\video data\january 28\audio\ZOOM0002.WAV

Associated videos: C:\Users\Amy\Google Drive\Kent\james\dissertation\chkv and dengue\data\video data\january 28

Neighborhood: Santa Elena

Driver/vector control specialist: David

Interviewer: Amy

Date: January 29, 2016

[00:00:00]

Amy:  here we are in Santa Elena that is the neighbor of Cristobal Colon and here again is the canal.

[00:00:11]

Amy:  and all the people who work here don't live here, says David. That they have to go to their house at night. Then this can also be a foci but they are not reporting here.

[00:00:21]

David: this is Santa helena from the highway to up there with the canal

[00:00:38]

Amy:  with the canal and all the people there.

[00:00:40]

David: and the people around it.

[00:00:42]

Amy:  in the gallery. and of course all the people come here to do their shopping and they go to their houses.

David: they go out and they are gone.

[00:00:50]

David: equally those who work here don't live here. they come to work.

[00:00:59]

Amy:  yes. of course, it has a lot of sense.

[00:01:07]

[00:01:29]

[00:01:37]

File: C:\Users\Amy\Google Drive\Kent\james\dissertation\chkv and dengue\data\video data\january 28\audio\ZOOM0003.WAV

Associated videos: C:\Users\Amy\Google Drive\Kent\james\dissertation\chkv and dengue\data\video data\january 28

Neighborhood: El Poblado 1

Driver/vector control specialist: David

Interviewer: Amy

Date: January 29, 2016

[00:00:00]

[00:00:43]

David: for me it has served me a lot to work in that company of fumigation becuase there I knew alot of

Amy:  yes. of the neighborhoods, of the risks

David: neighborhoods, nomenclature.

[00:00:54]

[00:01:21]

[00:01:42]

[00:02:26]

[00:02:48]

[00:03:05]

[00:04:10]

[00:04:48]

[00:05:26]

[00:06:09]

[00:06:25]

[00:06:53]

[00:07:20]

[00:07:37]

[00:08:44]

[00:09:36]

[00:10:11]

[00:10:48]

[00:11:14]

[00:11:59]

[00:12:31]

[00:12:59]

[00:13:42]

[00:13:55]

[00:15:12]

[00:15:57]

[00:17:20]

[00:17:27]

Amy:  this fire is always lit?

David: all day. that guy looks like a drug seller there.

[00:17:35]

David: as you saw those guys over there, the recyclers, they come here to smoke. They buy here and they start to smoke right away here. they are all recyclers.

[00:17:54]

[00:18:27]

[00:19:32]

[00:20:28]

[00:21:29]

David: that thing you see there is called Narino Colony. It was an invasion many years ago that was there. On this side is the hospital [] de cansino and on that side is all invasion. Over there is horrible. ALl the day and night is dangerous.

[00:21:53]

Amy:  and the invasion was of people from Narino?

David: initially it was all people from Narino but now any bad guy wants to go there.

[00:22:13]

[00:23:40]

[00:24:33]

[00:24:48]

[00:25:12]

[00:28:34]

[00:29:45]

[00:30:39]

Amy:  this side is much calmer

David: yes.

[00:30:47]

[00:30:59]

[00:31:27]

[00:33:19]

David: From here to there is Mojica. Mojica 2. From here it changes. I told you that Mojica is dangerous. Mojica we have to do too. From here to the canal.

Amy:  its small.

[00:33:31]

[00:33:35]

David: its dangerous. [] with the police.

[00:33:39]

Amy:  yes, better.

David: where the boys are at highschool, the black ones, with a bad outfit, having bad meetings.

Amy:  yes?

[00:33:52]

David: yes, those that are on bicycle, see. They are here to rob.

[00:33:57]

Amy:  then its that they don't have work here or?

David: they don't want.

[00:34:04]

Amy:  they don't want?

David: they could have work and everything. Look at these guys here, a group of drug users. Look.

[00:34:14]

David: and those little kids.

Amy:  oh, they have a knife.

[00:34:19]

David: knife, smoking drugs.

Amy:  its that they don't study?

[00:34:24]

David: they don't do anything.

[00:34:30]

David: from here to there is Mojica.

[00:34:42]

[00:35:56]

[00:36:57]

David: there are the police.

[00:37:05]

[00:37:07]

David: I think this here is the canal of rainwater, no.

Amy:  yes, in the principle.

[00:37:17]

[00:37:35]

[00:38:21]

[00:38:28]

David: here we arrive to the hospital.

[00:38:38]

David: and here is the hospital look. they fixed it up because it was a house.

Amy:  its big

David: they remodeled it beautifully.

David: and here is emergencies. those that come with an injury or with knife. they bring them all here.

[00:39:11]

Amy:  when we went to emergencies in HUV there were people with handcuffs.

[00:39:33]

David: we are missing these blocks until we get there to the canal.

[00:39:52]

Amy:  yes, we saw in emergencies of HUV that there were many people with handcuffs still becuase although they had injuries, they had to go to jail after.

David: yes, many times there are thieves and they are with the police.

[00:40:16]

David: the police take care of them there until they are cured and they take them to the jail.

[00:40:20]

David: the stories one hears here.

[00:40:35]

[00:40:46]

David: for that side over there is ugly. For that side were we passed by Mojica and the Narino colony, that is ugly ugly. We were going to fumigate in the truli car to the hospital, in the other one i told you was over there, and one day they came out with a dead guy there. And "you have to give us money, you have to give us money." Becuase they killed, the police he killed. that I don't know what. and we "no, relax." I had about 5 thousand pesos and psu.

Amy:  of course, bye.

David: 5 thousand pesos so they buried him.

[00:41:30]

Amy:

David: collaborate so they buried him. and my friend too gave 5 thousand. "no, go for it, man. we don't see anything."

[00:41:41]

Amy:  no, we don't see anything. its fine. but you had to pay to bury . . .

David: to be able to pass there becuase they were with the dead person.

[00:41:51]

Amy:  ah.

[00:41:53]

David:  [] were we were on the other side behind this building, the [] seperates the canal.

Amy:  mhm.

[00:42:05]

David: and all of this they fixed up and its beautiful but this at night is so dangerous.

[00:42:15]

Amy:  yes, this park?

David: yes, this poblado 2 is dangerous.

[00:42:20]

Amy:  in the day it looks very normal.

David: it looks relaxed.

Amy:  the people are outside talking

David: but at night i think all the world locks themselves inside.

[00:42:30]

Amy:  huh, and the crazy people come out.

David: the crazy people come out to screw in the street.

[00:42:38]

Amy:  hm

David: to see who they can rob

[00:42:41]

Amy:  no

[00:42:48]

[00:44:29]

[00:45:01]

[00:45:32]

[00:46:11]

[00:46:19]

David: imagine these alleys at night with people.

Amy:  mhm, full of people, you couldn't pass.

[00:46:29]

David: nobody could pass and at night, [] better said.

[00:46:35]

[00:48:36]

[00:49:55]

[00:50:38]

[00:51:00]

David: this street doesn't seem to dangerous. I think at night it's not too dangerous. There are others that should be more dangerous.

Amy:  mhm.

[00:51:10]

[00:52:05]

[00:52:57]

[00:53:57]

[00:55:31]

[00:55:47]

David: all of these here have the same style in the parks.

Amy:  mhm, the parks are beautiful.

[00:55:55]

[00:56:13]

David: this is to claim medicine from the insurance.

[00:56:22]

David: the subsidized pharmacy. the people who don't pay insurance, the attend them anyway. if they have to come to get medicine here in these.

Amy:  ah, ok. that's why the line is so long.

David: yes.

[00:56:42]

[00:56:51]

File: C:\Users\Amy\Google Drive\Kent\james\dissertation\chkv and dengue\data\video data\january 28\audio\ZOOM0004.WAV

Associated videos: C:\Users\Amy\Google Drive\Kent\james\dissertation\chkv and dengue\data\video data\january 28

Neighborhood: El Diamante

Driver/vector control specialist: David

Interviewer: Amy

Date: January 29, 2016

[00:00:00]

[00:00:17]

David: El Diamante neighborhood is a medium social strata.

[00:00:36]

David: The neighborhood is old and its not too small. Its medium. The size.

[00:00:43]

Amy:  It also has a lot of comercial area.

[00:01:00]

[00:01:21]

[00:01:45]

[00:01:58]

[00:02:15]

[00:06:06]

[00:07:46]

David: the neighborhood Diamante doesn't have a canal or residual water canals or anything.

Amy:  it's only for its neighbors, maybe?

David: yes.

[00:08:03]

Amy:  those that work in the other parts come here at night or what?

David: uhuh. well this part high maybe overflows when it rains and create puddles.

[00:08:13]

Amy:  mhm .

David: all of the 42 is unpaved, did you see?

[00:08:20]

Amy:  unpaved, the canal?

David: no, the street is not paved.

[00:08:25]

Amy:  ah ok, the streets are not in good condition.

David: yes, all of the 42 is this one.

[00:08:30]

David: this is the police station of the Diamante but look they have there sticks in the way for the private cars.

Amy:  ah, ok.

[00:08:55]

[00:09:16]

[00:09:36]

[00:10:18]

[00:10:49]

[00:11:34]

[00:13:37]

David: a corner of drug users here.

Amy:  how do you know?

David: they are all []

[00:13:48]

[00:14:59]

David: this here is a recreative center. THe center of family wellness of Diamante.

Amy:  mhm.

[00:15:12]

[00:15:46]

[00:16:20]

David: smoking marijuana there. boys.

Amy:  yes. they are very young.

[00:16:31]

David: selling. look at the one on the bike selling.

[00:16:34]

[00:16:54]

[00:17:04]

[00:17:16]

[00:17:36]

[00:18:45]

[00:19:08]

[00:19:56]

[00:20:11]

[00:21:12]

[00:21:54]

[00:22:45]

[00:23:08]

[00:23:27]

[00:23:56]

[00:24:40]

[00:24:54]

[00:25:42]

[00:26:27]

[00:26:55]

[00:27:15]

[00:27:40]

[00:28:09]

[00:28:34]

[00:28:34]

[00:28:56]

[00:29:18]

[00:29:40]

[00:29:55]

[00:30:44]

[00:31:22]

[00:31:45]

[00:31:59]

[00:33:36]

File: C:\Users\Amy\Google Drive\Kent\james\dissertation\chkv and dengue\data\video

data\January 29\audio\ZOOM0002.WAV

Neighborhood: Marroquin 3 and Bonilla Aregon at [00:32:56]

Driver/vector control specialist: David

Interviewer: Amy

Community health worker: Luz Angela Peralta Valencia

Date: January 29, 2016

[00:34:41]

[00:34:58]

[00:35:04] Luz Angela: But, it is the lack of control, that is--

[00:35:09] David: They should decide.

[00:35:09] Luz Angela: They have to decide already.

[00:35:11] David: How are they going to claim another one?

[00:35:13] Luz Angela: Well, if people are like so that—Poverty is exactly that, poverty of

conscious, poverty of everything..

[00:35:21] Luz Angela: That is poverty, is just not about the money, but if not believe that

money--

[00:35:30] Luz Angela: They keep living in the same conditions just to have a house that they do

no inhabit.

[00:35:36] David: We stayed in M. is that M too, yes?

[00:35:39] Luz Angela: M two.

[00:35:41] Amy: Mhm

[00:35:42] Luz Angela: Seventy-three B M two Carrera twenty-six. M two.

[00:35:49] David: And it starts from the M. That is, that is M one and then M alone follows.

[00:35:59]

[00:36:18]

[00:36:27]

[00:36:34]

[00:36:52]

Henriquez 2

[00:36:59]

[00:37:11]

[00:37:18] David: And they are cleaning the—those siphons because it is sure that it is going to

rain. They are preventing--

[00:37:27] Luz Angela: Flooding.

[00:37:31] Amy: That is good.

[00:37:32] Amy: They are taking care of that.

[00:37:35] Luz Angela: Uh, they must remove all kinds of things.

[00:37:43]

[00:38:04]

[00:38:34]

[00:39:14]

[00:39:37]

[00:39:53]

[00:40:18]

[00:40:49]

[00:40:59] Amy: Are we in twenty-six M one?

[00:41:02] David: Mhm.

[00:41:03] Amy: Above this one.

[00:41:06] David: But we are in eighty-seven.

[00:41:09] Amy: We are in eighty-seven? Okay.

[00:41:13] Amy: We are going up.

[00:41:16] David: Yes.

[00:41:17] Amy: Okay

[00:41:32]

[00:42:03] David: The air conditioning doesn’t harm me, but it harms other people. It harms my

wife.

[00:42:10] Luz Angela: Yes, I have a cough like—I have to go to ____ so I can take a

((bacilloscopy)) test.

[00:42:22]

[00:42:27] Luz Angela:  But, is more like an allergic reaction, yes.

Henriquez 3

[00:42:30] David: Allergic. She has gotten it bad. On these days, the same thing has happened to

her. Fifteen more days and she has to go get a ((bacilloscopy)).

[00:42:37] Luz Angela: No, and even more with her that she is with the program.

[00:42:41] Amy: Yes.

[00:42:41] Luz Angela: Everyone must be very attentive because, the ones who—A doctor who

works with tuberculosis was telling me, that the number of nurses and doctors who had be

contaminated with tuberculosis was higher.

[00:43:04] Luz Angela: Because one thought that tuberculosis was only a problem with ((    ))

and poverty. But also, in hospitals one can.

[00:43:16] David: No, of course, if the immune system is weak.

[00:43:19] Luz Angela: Yes, because from anything, not necessary malnutrition.

[00:43:25] David: It is like the resistance of the organism.

[00:43:30] David: At least, it is difficult for me to get sick, ((better said)).  That’s how____ is, it

is very difficult for her to get sick. That’s why when she got that Chikungunya, she ((      ))

[00:43:43] Luz Angela: It broke her.

[00:43:44] David: Because she was the type of person you never saw lying down, or like she

could not even get up. No, that is weird.

[00:43:52] Luz Angela: Uh, no, that Chikungunya—When the pain started, I tried getting up and

could not. That is to say, I turned around and I sat down, but I could not get out of bed.

[00:44:08] David: That’s how she was. And she could not do anything. ((She said that here in her

hands, they couldn’t reach to move. ))

[00:44:19] Luz Angela: I was, well with the incapacity for the—for symptomology I was

incapacitated for twelve days.

[00:44:29] Luz Angela:  And it continued for almost six months.

[00:44:33] David: And now that Zika one can supposedly last even a year in the body.

[00:44:41] Luz Angela: In the organism, yes.

[00:44:43] David: In the entire organism.

[00:44:46] David: And, it always has the tendency to neurologic harm.

[00:44:51] Luz Angela: That’s why is it being associated with Guillain-Barre.

[00:45:01]

[00:45:02] David: We are going to twenty what?

Henriquez 4

[00:45:04] Amy: Twenty-six M until-- Carrera twenty-six M until Carrera twenty eight D.

[00:45:11] David: Twenty-eight now. We are going to twenty-six B. They are increasing.

[00:45:21]

[00:45:28]

[00:45:34]

[00:45:40]

[00:45:47]

[00:45:53]

[00:45:57]

[00:46:03]

[00:46:11]

[00:46:17]

[00:46:24]

[00:46:30]

[00:46:36]

[00:46:42]

[00:46:48]

[00:46:52]

[00:46:57]

[00:47:10]

[00:47:17]

[00:47:22]

[00:47:28]

[00:47:37]

[00:47:44]

[00:47:50]

[00:47:56]

[00:48:02]

[00:48:08]

[00:48:18]

[00:48:24]

Henriquez 5

[00:48:31]

[00:48:38]

[00:48:45]

[00:48:53]

[00:48:58]

[00:49:04]

[00:49:11]

[00:49:20]

[00:49:27]

[00:49:34]

[00:49:41]

[00:49:49]

[00:49:56]

[00:50:02]

[00:50:11]

[00:50:18]

[00:50:26]

[00:50:32]

[00:50:38]

[00:50:45]

[00:50:49]

[00:50:56]

[00:50:59]

[00:51:07]

[00:51:13]

[00:51:20]

[00:51:26]

[00:51:33]

[00:51:41]

[00:51:49]

[00:51:57]

Henriquez 6

[00:52:05]

[00:52:13]

[00:52:16] David: Yes look, they are cleaning with the ((     )).

[00:52:22] David: Look at those drains too. They are the ones Public Health are controlling

the—The ((toilets)) too.

[00:52:28] Amy: Okay

[00:52:31] David: And since they named ((It was commune)). In what communes where they

going to attack more?

[00:52:37] Amy: All of the ones we are crossing. The same ones. They said that Calypsos was

the most dangerous one.

[00:52:46] David: Ah Calypso, do you remember when we stopped there and we were looking at

the canal that they were remodeling and it was got blocked.

[00:52:51] Amy: Mhm. The lagoon.

[00:52:59] David: I believe that—I feel like if the water is moving, they do not have a chance of

reproducing.

[00:53:06] Amy: No, that’s the problem. If it’s moving, it is good.

[00:53:13] Amy: Even thought they can also reproduce in the border of the river.

[00:53:20] Luz Angela: In the shore, yes.

[00:53:22] David: Of course, but if there is something that like helps the water stop there.

[00:53:26] David: But that one, since they are remodeling it, is stopped completely.

[00:53:33] David: That is a little bit--

[00:53:36] Amy: Terrible.

[00:53:39] David: Yes, primarly there.

[00:53:50]

[00:54:00]

[00:54:08]

[00:54:14]

[00:54:22]

[00:54:30]

[00:54:38]

[00:54:46]

Henriquez 7

[00:54:54]

[00:55:02]

[00:55:10]

[00:55:18]

[00:55:26]

[00:55:34]

[00:55:42]

[00:55:51]

[00:55:58]

[00:56:06]

[00:56:12]

[00:56:20]

[00:56:28]

[00:56:37]

[00:56:46]

[00:56:55]

[00:57:03]

[00:57:11]

[00:57:20]

[00:57:30]

[00:57:40]

[00:57:49]

[00:57:56]

[00:58:04]

[00:58:09]

[00:58:15]

[00:58:29]

[00:58:35]

[00:58:44]

[00:58:52]

[00:58:59]

Henriquez 8

[00:59:04] Amy: But, the canal keeps going until the south, correct? From north to the south?

[00:59:10] Luz Angela: Yes, those canals are long.

[00:59:12] Amy: That means that the canal…

[00:59:14] David: That is the one that crosses the City of Cali; it goes through the entire city.

[00:59:17] Amy: Then why does it not ((   )) with another part?

[00:59:27] Amy: Because, we can see that they are canals in every neighborhood here, but there

are also other neighborhoods.

[00:59:33] Luz Angela: What happens is that this canal from—The city of Cali, right. And where

does the city of Cali’s canal come from?

[00:59:44]

[00:59:46] David: That canal, from where I have seen it, comes from Navarro.

[00:59:59] David: That one that is there from fifty-six, you can see it from Navarro’s shore.

[01:00:04] David: It meets with this one and it comes from all of the city of Cali.

[01:00:09] Luz Angela: And the city of Cali ends in Córdoba.

[01:00:14] David: No, the city of Cali ends, ah from here in the south, yes. But, the in the north it

ends there in that passage of the commerce.

[01:00:25] David: And there the canal ends in the Cauca river.

[01:00:28] David: But then there in Pertecuy they treat the residual waters and they throw them

to—

[01:00:40]

[01:00:48]

[01:00:50]

[01:00:58]

[01:01:09]

[01:01:17]

[01:01:24]

[01:01:32]

[01:01:40]

[01:01:48]

[01:01:55]

[01:02:04]

Henriquez 9

[01:02:12]

[01:02:20]

[01:02:26]

[01:02:34]

[01:02:42]

[01:02:52]

[01:03:01]

[01:03:14]

[01:03:21]

[01:03:31]

[01:03:40]

[01:03:51]

[01:04:00]

[01:04:07]

[01:04:14]

[01:04:20]

[01:04:29]

[01:04:40]

[01:04:50]

[01:05:03]

[01:05:25]

[01:05:33] David: I think that the zone that affects this is the canal.

[01:05:38] Amy: But we still don’t know why the canal doesn’t affect other places. Well that...

[01:05:44] David: There, do you see how the ((cluster)) until where the spots are, the hot spots,

right. And there is where you know that it is because of the canal

[01:05:53] Amy: Yes. Ah yes, ____ said something about the spots.

[01:05:57] David: Aha.

[01:05:58] Amy: Do they already have the spots, or no?

[01:06:00] David:Well um, of the – Dengue?

[01:06:07] Amy: Of the engineer, the directions, no?

[01:06:10] David: Ah, the ones the sir was going to do? Yes, he is doing the job.

Henriquez 10

[01:06:15] Amy: Ah, good.

[01:06:17] David: I think he will deliver them today.

[01:06:20] David: Yes, the town hall’s engineer.

[01:06:21] Amy: Can we stop for a moment?

[01:06:24]

[01:06:34]

[01:06:44]

[01:06:54]

[01:07:03]

[01:07:12]

[01:07:23]

[01:07:31]

[01:07:39] Luz Angela: There are various spots where they have been cleaning, that we have

found people.

[01:07:45] Amy: But, before we did not see that, right?

[01:07:48] David: No.

[01:07:50] Amy: That is something that they were not doing a couple of months and weeks ago.

It is new.

[01:07:53] Luz Angela: No. I think what the secretary is – She is deploying people to check it

and give education in regards to Zika.

[01:08:06] Amy: That is very good.

[01:08:07] Luz Angela: Yes. They had ninety people, ninety people that I understood that had to

come educate and--

[01:08:18] David: And fumigate too, I heard that they are training you in the stadium.

[01:08:22] Luz Angela: Look, there is another one in ((abril)) that they already opened, look.

[01:08:26] Luz Angela: Look at all the trash that they take out of there, look.

[01:08:29] Amy: But, do they leave it there?

[01:08:31] Luz Angela: I think they know, they come pick it up afterwards.

[01:08:34] David: Yes. They have to clean and the comes another car and picks it up

[01:08:39] Luz Angela: Look.

[01:08:40] Amy: Ah yes.

Henriquez 11

[01:08:41] Luz Angela: All of those--

[01:08:42] Amy: That was a risk before each time we saw one of the siphons- that they stayed

dirty.

[01:08:53] David: The work is hard, yes or no?

[01:08:55] Luz Angela: Yes. Getting all of that filth from everyone out.

[01:08:58] David: No and not only that, but how that smells, is horrible.

[01:09:03] Luz Angela: Yes, horrible.

[01:09:04] And that lady working there ((       )) on that.

[01:09:07]

[01:09:18]

[01:09:27]

[01:09:35]

[01:09:43]

[01:09:51]

[01:10:00]

[01:10:02] Amy: And that sand that they have there, what’s the use for it?

[01:10:07] David: The what?

[01:10:09] Luz Angela: The sand.

[01:10:09] Amy: The sand.

[01:10:14] Luz Angela: Constructions that they will soon—That they do.

[01:10:23]

[01:10:38]

[01:10:46] Luz Angela: Look, event here they clean the-- The siphons.

[01:10:52] David: Is that they are many, look. They are also cleaning there. And they are resting.

[01:10:58]

[01:11:05]

[01:11:13]

[01:11:21]

[01:11:30]

[01:11:40]

[01:11:50]

Henriquez 12

[01:12:00]

[01:12:10]

[01:12:20]

[01:12:30]

[01:12:40]

[01:12:50]

[01:13:00]

[01:13:10]

[01:13:20]

[01:13:30]

[01:13:40]

[01:13:46] Luz Angela: That is the city of Cali.

[01:13:49] David: This on--

[01:13:50] Luz Angela: Which one is it?

[01:13:52] David: Is another one. This one goes through Marroquin.

[01:14:00]

[01:14:10] David: This is the twenty-eight. Right, Amy?

[01:14:13] Amy: Yes, twenty-eight D.

[01:14:14] Luz Angela: Ninety-nine.

[01:14:30]

[01:14:43]

[01:14:53]

[01:15:00]

[01:15:18]

[01:15:30]

[01:15:40]

[01:15:50]

[01:16:00]

[01:16:10]

[01:16:20]

[01:16:33]

Henriquez 13

[01:16:40]

[01:16:50]

[01:17:00]

[01:17:09]

[01:17:20]

[01:17:30]

[01:17:40]

[01:17:50]

[01:18:00]

[01:18:12]

[01:18:20]

[01:18:30]

[01:18:40]

[01:18:50]

[01:19:00]

[01:19:10]

[01:19:20]

[01:19:30]

[01:19:40]

[01:19:51]

[01:20:05]

File: C:\Users\Amy\Google Drive\Kent\james\dissertation\chkv and dengue\data\video data\january 28\audio\ZOOM0004.WAV

Associated videos: C:\Users\Amy\Google Drive\Kent\james\dissertation\chkv and dengue\data\video data\january 28

Neighborhood: El Diamante

Driver/vector control specialist: David

Interviewer: Amy

Date: January 29, 2016

[00:00:00]

[00:00:17]

David: El Diamante neighborhood is a medium social strata.

[00:00:36]

David: The neighborhood is old and its not too small. Its medium. The size.

[00:00:43]

Amy:  It also has a lot of comercial area.

[00:01:00]

[00:01:21]

[00:01:45]

[00:01:58]

[00:02:15]

[00:06:06]

[00:07:46]

David: the neighborhood Diamante doesn't have a canal or residual water canals or anything.

Amy:  it's only for its neighbors, maybe?

David: yes.

[00:08:03]

Amy:  those that work in the other parts come here at night or what?

David: uhuh. well this part high maybe overflows when it rains and create puddles.

[00:08:13]

Amy:  mhm .

David: all of the 42 is unpaved, did you see?

[00:08:20]

Amy:  unpaved, the canal?

David: no, the street is not paved.

[00:08:25]

Amy:  ah ok, the streets are not in good condition.

David: yes, all of the 42 is this one.

[00:08:30]

David: this is the police station of the Diamante but look they have there sticks in the way for the private cars.

Amy:  ah, ok.

[00:08:55]

[00:09:16]

[00:09:36]

[00:10:18]

[00:10:49]

[00:11:34]

[00:13:37]

David: a corner of drug users here.

Amy:  how do you know?

David: they are all []

[00:13:48]

[00:14:59]

David: this here is a recreative center. THe center of family wellness of Diamante.

Amy:  mhm.

[00:15:12]

[00:15:46]

[00:16:20]

David: smoking marijuana there. boys.

Amy:  yes. they are very young.

[00:16:31]

David: selling. look at the one on the bike selling.

[00:16:34]

[00:16:54]

[00:17:04]

[00:17:16]

[00:17:36]

[00:18:45]

[00:19:08]

[00:19:56]

[00:20:11]

[00:21:12]

[00:21:54]

[00:22:45]

[00:23:08]

[00:23:27]

[00:23:56]

[00:24:40]

[00:24:54]

[00:25:42]

[00:26:27]

[00:26:55]

[00:27:15]

[00:27:40]

[00:28:09]

[00:28:34]

[00:28:34]

[00:28:56]

[00:29:18]

[00:29:40]

[00:29:55]

[00:30:44]

[00:31:22]

[00:31:45]

[00:31:59]

[00:33:36]

File: C:\Users\Amy\Google Drive\Kent\james\dissertation\chkv and dengue\data\video data\January 29\audio\ZOOM0002.WAV

Neighborhood: Marroquin 3 and Bonilla Aregon at [00:32:56]

Driver/vector control specialist: David

Interviewer: Amy

Community health worker: Luz Angela Peralta Valencia

Date: January 29, 2016

[00:00:00]

Amy:  ok, we are in Marroquin 3, today is January 29 with David and Luz Angela and we are going to see what it has to have dengue and chikungunya

Luz Angela: dengue and chikungunya.

Amy:  and zika now.

[00:00:23]

Luz Angela: here what do I write?

Amy:  can I use your voice in the investigation, in meetings, or in publications? yes or no? If it's yes, mark an x.

Luz Angela: x

[00:00:38]

Luz Angela: here too? and here

Amy:  yes.

Luz Angela: and here I said yes.

Amy:  ok.

[00:00:46]

Amy:  and here you have to put the date and id

[00:01:04]

Amy:  and here is the explanation of everything that we are doing so I will leave one of these with you. and here you can sign with your id. this is just what i already told you we are doing.

[00:01:25]

Luz Angela: id.

[00:01:29]

[00:02:01]

Amy:  so when you had chikungunya, did you think it was transmitted here or in another part? because you were working here, no?

[00:02:14]

Luz Angela: I assumed that it was in Jamundi.

Amy:  in Jamundi.

[00:02:18]

Luz Angela: yes. but equally from the sectors of community mothers that we had here, there were many incapacitations for chikungunya.

Amy:  yes?

Luz Angela: yes.

[00:02:32]

Luz Angela: what happened with those diagnostics- they got the incapacity and the doctor said "this is chikungunya," but the diagnosis that they had was to discard the dengue, initially.  yes?

[00:02:52]

Luz Angela: and then they were working with disability and when they came back they said it was a chikungunya for the symptoms. That is to say that they knew the symptoms of chikungunya so they told us it was a chikungunya. "i got a rash, my joints hurt." SO they assumed it was a chikungunya.

[00:03:14]

Luz Angela: but a diagnosis like a medical diagnosis we didn't find.

Amy:  ah.

[00:03:18]

Luz Angela: we always found to discard dengue.

[00:03:21]

Amy:  then the cases that you all had were not registered.

[00:03:27]

Luz Angela: eh, no they were not registered. Unless, unless, for example they did the analysis and took the samples and did the analysis. normally the cases that came to us.

[00:03:47]

David: look Amy, we are going to start here on this corner.

[00:03:50]

Amy:  ok.

[00:03:57]

[00:05:21]

[00:05:39]

Amy:  that is the same thing that happened to David's wife. She had dengue and went to the doctor to get a diagnosis without the test, right?

David: hm.

[00:05:52]

Amy:  then he told her, "come back on this day," but they don't have anything to give her so why would she want to go from work for the doctor then no. Then she didn't go and it wasn't registered either. Right, David?

[00:06:06]

Amy:  that Paola had dengue but it wasn't registered.

[00:06:11]

Luz Angela: they didn't diagnose her with chikungunya.

[00:06:13]

Luz Angela: the doctor supposed but they always had to discard dengue initially.

[00:06:17]

Luz Angela: with the hemorrhagic dengue, well, it was dangerous that both the association with dengue and chikungunya.

Amy:  yes.

[00:06:26]

Luz Angela: Yes?

Amy:  at the same time?

Luz Angela: at the same time. So, the discarded first the hemorrhagic dengue.

[00:06:31]

Luz Angela: they did the test of hemorrhagic dengue and after the ladies continued in the house with the symptoms of the chikungunya and simply didn't go to the trainings.

[00:06:50]

Amy:  mhm

Luz Angela: and when they came back, the came back with all the pain that lasted months so they told them "no, mine was dengue." They were the ones with the diagnosis. Mine wasn't a dengue. It was a chikungunya. Because look, it lasted so long and I had a rash and I a headache. And all my joints hurt. My feet []. Then all this process so long. They said "this is chikungunya." They were the ones that gave the diagnosis.

[00:07:26]

Amy:  oh, the mothers.

Luz Angela: the mothers, imagine.

[00:07:29]

Luz Angela: yes. as the transmission is oral, the transmission of knowledge is oral.

[00:07:43]

David: look at the canal of rain4water.

Amy:  yes, look here.

[00:07:46]

Luz Angela: this is the one you say is stagnant.

David: hm.

[00:07:49]

[00:08:13]

Amy:  we are filming all this. for example, the trash that is in the street, the water canal

Luz Angela: yes

Amy:  and the condition of this street that can create puddles of water.

[00:08:26]

Amy:  all this we can see.

Luz Angela: that there are streets without pavement.

David: mhm

[00:08:31]

Luz Angela: that there is a lot of, a lot of risk, danger.

Amy:  yes

Luz Angela: that they can produce places where it would be water

Amy:  yes, the mosquitoes

Luz Angela: where there is growth of the mosquito.

David: the stagnant water.

[00:08:48]

Amy:  and also we can see the people here who don't have clothes, who are in the street without clothes

David: uhuh

Amy:  this is because the mosquitoes can bite more then there are more opportunities because they aren't covering anything.

[00:09:01]

Amy:  who knows if they are using repellent or no.

Luz Angela: but as the mosquito goes out at certain hours, yes or no?

David: yes

[00:09:12]

Amy:  this one goes out during the day, no?

[00:09:13]

Luz Angela: but in some hours in the morning and some hours in the afternoon.

David: yes.

[00:09:18]

David: after 6 PM it’s terrible, the mosquitoes go out.

[00:09:23]

Luz Angela: yes.

David: the hours in the morning, at 7 or 8 when the people are opening their eyes, the mosquitoes appear.

Amy:  yes.

[00:09:30]

Luz Angela: and for example, all of these trees. these planters that they have outside.

Amy:  this, yes.

Luz Angela: all of this also contributes.

[00:09:49]

Amy:  yes. but as

Luz Angela: this, for example. The old constructions that they don't finish. Puddles form in the cement. Sometimes, they don't continue.

Amy:  this makes that it is more difficult to control the vector, no.

[00:10:09]

Luz Angela: of course. uhuh. because they don't climb up there to see if there are puddles of water. then there stays the vector.

[00:10:18]

Amy:  yes. although they are working, it is difficult to control everything.

[00:10:24]

David: and Marroquin is more tenacious because it has two. two canals. it has that one on that side and the one on this side.

[00:10:30]

Amy:  ah ok, it's on all the sides.

[00:10:33]

Luz Angela: and how often do they come to the . . .

David: this they did monthly or every third month. they didn't do it very constantly. that's the problem, it’s not constant.

[00:10:48]

Luz Angela: they didn't do a control of the control of dengue

David: right now the only thing they are doing is where there are the most cases and where they report most they are doing the control.

[00:11:01]

Luz Angela: and it's that its lacking the conscientiousness and education.

David: yes.

[00:11:10]

David: no, in terms better said, if they were conscious, the disease would fall.

[00:11:16]

David: if they didn't let accumulate for example these tires, things like that where they produce.

[00:11:29]

Luz Angela: this is the other canal.

[00:11:45]

David: this is the one of Ciudad de Cali. Imagine, two canals.

Luz Angela: yes.

[00:11:50]

Amy:  look at all the trash there in the canal.

[00:11:52]

Luz Angela: ah, yes, that's another thing. here, the management of the trash, no?

David: mhm

Luz Angela: they throw everything in the canal. There you can find furniture, you can find clothes, you can find even dead people.

[00:12:05]

Amy:  huh.

[00:12:14]

[00:13:00]

Luz Angela: look, we had on one occasion, we were, how many groups did we have in that time, we had about 7 groups of community mothers, and we counted almost 11 cases of chikungunya. yes? in any committee that they did, "ah no, in that group there are this many incapacitated for chikungunya." yes? but i tell you because it was the mothers who said "i have chikungunya." Because the medical diagnosis of chikungunya didn't reach us.

[00:13:40]

Luz Angela: no, it was a test to discard dengue and after the moms came back and said "no, i had a chikungunya because i had this, my feet swelled, i got a rash." even more, they invented so many treatments themselves. One treatment was the juice, in the morning, they drank blackberry juice with beet and celery. and that took away the chikungunya.

[00:14:15]

Amy:  or the pain.

Luz Angela: yes.

David: or also they take something mystic, like juice of the papaya leaves. of the papaya plant

Luz Angela: also, yes.

David: it raises the antibody levels, of the defenses.

[00:14:34]

David: they people took that. They gave Paola that because she was so bad that I didn't know what to do for her. Nothing gave her relief and I read that on internet and many people say it worked.

[00:14:51]

Luz Angela: yes.

David: then i went and i got the papaya leaves, i washed them, and crushed them and took out the pulp and i gave it her.

Amy:  and it worked?

David: well, she got better.

Amy:  ok.

[00:15:02]

Amy:  of all the cases that i received for chikungunya, only 5% are on the individual level. That is, if in this neighborhood there are 100 cases, i only received 5 id's and names.

Luz Angela: yes, because the doctor without the test of chikungunya can't write there that there is chikungunya

Amy:  yes

Luz Angela: and they have to discard the association of dengue with chikungunya

[00:15:43]

Amy:  yes

Luz Angela: that would be very dangerous, yes? so they. . . or you find, yes? you present the symptoms of chikungunya but they don't diagnose the chikungunya, not presented. Like I got it. When chikungunya started in me, the diagnosis was that way. Presented symptoms, nothing else. But they never told me I had chikungunya nor sent me for an exam or anything.

[00:16:18]

Amy:  No, without confirmation. Because the only place that I saw they have the exam is in Valle de Lilli.

Luz Angela: where?

Amy:  in the hospital of Valle de Lilli.

Luz Angela: ah, Valle de Lilli.

[00:16:30]

Luz Angela: but yes, this has to do with how the people live, no? how, how the people move socially. How, for example, I don't know if it would have to do with, for example, in the neighborhoods where there are more risks and danger to present dengue than for example in Ciudad Jardin. Yes?

Amy:  yes, because, well, the cases in another neighborhood where they can rest in the house without transmission between me and you by mosquito, this is important to because if there is one case it’s okay but if you go outside with the case and the transmission starts, this is. . .

[00:17:38]

Luz Angela: and there are no canals over there

Amy:  mhm, yes, less breeding sites.

[00:17:44]

Amy:  although in the streets too, there are streets that are bad and have water but . . . and also they bit me a lot in ciudad Jardin. they bit me a lot in the house but . . .

Luz Angela: but those didn't have chikungunya

Amy:  they didn't have chikungunya because there weren't many cases there.

[00:18:04]

Luz Angela: there weren't many cases. other the other hand here, when they clean, when they sleep three or four people in the same room and one is picked by chikungunya it is possible that the same mosquito goes and bites another person.

Amy:  of course, because one mosquito is biting me and I do this and he goes to pick you then we both have it.

[00:18:29]

David: the zika, yesterday they were saying that a man who was fumigating in brazil, he was from the US, he went to the US and the spouse got zika. That is to say that maybe for sexual contact it was transmitted.

[00:18:46]

Amy:  really?

David: uhuh.

Luz Angela: with this has come many things because in one. . .

David: he was fumigating in Brazil.

[00:18:53]

Luz Angela: in some news i heard about zika, they said its presenting in the Atlantic, in Barranquilla or something like that. one of those cities of the Caribbean and they were associating the zika with Guillain-Barre

[00:19:11]

Luz Angela: 14 cases are something like that, I understood, that there were 14 cases of Guillain-Barre and they were associated with zika bites.

[00:19:19]

Amy:  that is dangerous no?

Luz Angela: then, there are no studies and all the world, well, the people begin to suppose a lot of things and . . . well I don't know, to believe so much terror without knowing.

[00:19:43]

Amy:  yes

Luz Angela: or one doesn't know if the terror is better than than, than, right? as prevention?

[00:19:50]

Amy:  yes, it’s that they don't say. you are right, they don't know, so they are trying to take precautions.

Luz Angela: yes.

[00:20:01]

[00:20:35]

David: the house is empty so they take the spout from outside and they rob the water. then they don't use their water but rather the water of the neighborhood that's not there.

Amy:  wow

[00:20:51]

David: it’s an abuse.

Luz Angela: and relaxed, no? they don't care if they are seen.

[00:20:54]

David: they don't care if the people pass and see them or anything.

[00:20:56]

Luz Angela: the damage is normal. Here everyone lives off everyone and the one that leaves it, we crush him.

[00:21:04]

Luz Angela: correct?

David: disgraceful, no?

[00:21:15]

[00:21:31]

[00:24:27]

[00:24:32]

[00:25:46]

[00:26:10]

[00:26:58]

[00:28:57]

[00:29:54]

[00:30:13]

[00:30:47]

Luz Angela: look, all of this serves to accumulate water. all the plants outside.

[00:30:55]

Amy:  all the holes, plants, trash.

[00:30:57]

David: ok, then I think here we have finished with Marroquin.

[00:31:25]

[00:31:55]

Luz Angela: look at all the tires there are. They make games for the kids with tires but they don't share...

Amy:  these are okay because look, all that part is under ground where the water would accumulate.

[00:32:21]

[00:32:51]

Luz Angela: look, the parks with tires.

[00:32:57]

David: but look, they all have this is common.

Luz Angela: the canal.

David: yes, I think this is the worst breeding site of mosquitoes.

[00:33:14]

Amy:  yes, and the bush.

[00:33:21]

[00:34:59]

[00:34:41]

[00:34:58]

[00:35:04] Luz Angela: But, it is the lack of control, that is--

[00:35:09] David: They should decide.

[00:35:09] Luz Angela: They have to decide already.

[00:35:11] David: How are they going to claim another one?

[00:35:13] Luz Angela: Well, if people are like so that—Poverty is exactly that, poverty of conscious, poverty of everything..

[00:35:21] Luz Angela: That is poverty, is just not about the money, but if not believe that money--

[00:35:30] Luz Angela: They keep living in the same conditions just to have a house that they do no inhabit.

[00:35:36] David: We stayed in M. is that M too, yes?

[00:35:39] Luz Angela: M two.

[00:35:41] Amy: Mhm

[00:35:42] Luz Angela: Seventy-three B M two Carrera twenty-six. M two.

[00:35:49] David: And it starts from the M. That is, that is M one and then M alone follows.

[00:35:59]

[00:36:18]

[00:36:27]

[00:36:34]

[00:36:52]

[00:36:59]

[00:37:11]

[00:37:18] David: And they are cleaning the—those siphons because it is sure that it is going to rain. They are preventing--

[00:37:27] Luz Angela: Flooding.

[00:37:31] Amy: That is good.

[00:37:32] Amy: They are taking care of that.

[00:37:35] Luz Angela: Uh, they must remove all kinds of things.

[00:37:43]

[00:38:04]

[00:38:34]

[00:39:14]

[00:39:37]

[00:39:53]

[00:40:18]

[00:40:49]

[00:40:59] Amy: Are we in twenty-six M one?

[00:41:02] David: Mhm.

[00:41:03] Amy: Above this one.

[00:41:06] David: But we are in eighty-seven.

[00:41:09] Amy: We are in eighty-seven? Okay.

[00:41:13] Amy: We are going up.

[00:41:16] David: Yes.

[00:41:17] Amy: Okay

[00:41:32]

[00:42:03] David: The air conditioning doesn’t harm me, but it harms other people. It harms my wife.

[00:42:10] Luz Angela: Yes, I have a cough like—I have to go to ____ so I can take a ((bacilloscopy)) test.

[00:42:22]

[00:42:27] Luz Angela:  But, is more like an allergic reaction, yes.

[00:42:30] David: Allergic. She has gotten it bad. On these days, the same thing has happened to her. Fifteen more days and she has to go get a ((bacilloscopy)).

[00:42:37] Luz Angela: No, and even more with her that she is with the program.

[00:42:41] Amy: Yes.

[00:42:41] Luz Angela: Everyone must be very attentive because, the ones who—A doctor who works with tuberculosis was telling me, that the number of nurses and doctors who had be contaminated with tuberculosis was higher.

[00:43:04] Luz Angela: Because one thought that tuberculosis was only a problem with (( )) and poverty. But also, in hospitals one can.

[00:43:16] David: No, of course, if the immune system is weak.

[00:43:19] Luz Angela: Yes, because from anything, not necessary malnutrition.

[00:43:25] David: It is like the resistance of the organism.

[00:43:30] David: At least, it is difficult for me to get sick, ((better said)).  That’s how____ is, it is very difficult for her to get sick. That’s why when she got that Chikungunya, she ((      ))

[00:43:43] Luz Angela: It broke her.

[00:43:44] David: Because she was the type of person you never saw lying down, or like she could not even get up. No, that is weird.

[00:43:52] Luz Angela: Uh, no, that Chikungunya—When the pain started, I tried getting up and could not. That is to say, I turned around and I sat down, but I could not get out of bed.

[00:44:08] David: That’s how she was. And she could not do anything. ((She said that here in her hands, they couldn’t reach to move. ))

[00:44:19] Luz Angela: I was, well with the incapacity for the—for symptomology I was incapacitated for twelve days.

[00:44:29] Luz Angela:  And it continued for almost six months.

[00:44:33] David: And now that Zika one can supposedly last even a year in the body.

[00:44:41] Luz Angela: In the organism, yes.

[00:44:43] David: In the entire organism.

[00:44:46] David: And, it always has the tendency to neurologic harm.

[00:44:51] Luz Angela: That’s why is it being associated with Guillain-Barre.

[00:45:01]

[00:45:02] David: We are going to twenty what?

[00:45:04] Amy: Twenty-six M until-- Carrera twenty-six M until Carrera twenty eight D.

[00:45:11] David: Twenty-eight now. We are going to twenty-six B. They are increasing.

[00:45:21]

[00:45:28]

[00:45:34]

[00:45:40]

[00:45:47]

[00:45:53]

[00:45:57]

[00:46:03]

[00:46:11]

[00:46:17]

[00:46:24]

[00:46:30]

[00:46:36]

[00:46:42]

[00:46:48]

[00:46:52]

[00:46:57]

[00:47:10]

[00:47:17]

[00:47:22]

[00:47:28]

[00:47:37]

[00:47:44]

[00:47:50]

[00:47:56]

[00:48:02]

[00:48:08]

[00:48:18]

[00:48:24]

[00:48:31]

[00:48:38]

[00:48:45]

[00:48:53]

[00:48:58]

[00:49:04]

[00:49:11]

[00:49:20]

[00:49:27]

[00:49:34]

[00:49:41]

[00:49:49]

[00:49:56]

[00:50:02]

[00:50:11]

[00:50:18]

[00:50:26]

[00:50:32]

[00:50:38]

[00:50:45]

[00:50:49]

[00:50:56]

[00:50:59]

[00:51:07]

[00:51:13]

[00:51:20]

[00:51:26]

[00:51:33]

[00:51:41]

[00:51:49]

[00:51:57]

[00:52:05]

[00:52:13]

[00:52:16] David: Yes look, they are cleaning with the ((     )).

[00:52:22] David: Look at those drains too. They are the ones Public Health are controlling the—The ((toilets)) too.

[00:52:28] Amy: Okay

[00:52:31] David: And since they named ((It was commune)). In what communes where they going to attack more?

[00:52:37] Amy: All of the ones we are crossing. The same ones. They said that Calypsos was the most dangerous one.

[00:52:46] David: Ah Calypso, do you remember when we stopped there and we were looking at the canal that they were remodeling and it was got blocked.

[00:52:51] Amy: Mhm. The lagoon.

[00:52:59] David: I believe that—I feel like if the water is moving, they do not have a chance of reproducing.

[00:53:06] Amy: No, that’s the problem. If it’s moving, it is good.

[00:53:13] Amy: Even thought they can also reproduce in the border of the river.

[00:53:20] Luz Angela: In the shore, yes.

[00:53:22] David: Of course, but if there is something that like helps the water stop there.

[00:53:26] David: But that one, since they are remodeling it, is stopped completely.

[00:53:33] David: That is a little bit--

[00:53:36] Amy: Terrible.

[00:53:39] David: Yes, primarly there.

[00:53:50]

[00:54:00]

[00:54:08]

[00:54:14]

[00:54:22]

[00:54:30]

[00:54:38]

[00:54:46]

[00:54:54]

[00:55:02]

[00:55:10]

[00:55:18]

[00:55:26]

[00:55:34]

[00:55:42]

[00:55:51]

[00:55:58]

[00:56:06]

[00:56:12]

[00:56:20]

[00:56:28]

[00:56:37]

[00:56:46]

[00:56:55]

[00:57:03]

[00:57:11]

[00:57:20]

[00:57:30]

[00:57:40]

[00:57:49]

[00:57:56]

[00:58:04]

[00:58:09]

[00:58:15]

[00:58:29]

[00:58:35]

[00:58:44]

[00:58:52]

[00:58:59]

[00:59:04] Amy: But, the canal keeps going until the south, correct? From north to the south?

[00:59:10] Luz Angela: Yes, those canals are long.

[00:59:12] Amy: That means that the canal…

[00:59:14] David: That is the one that crosses the City of Cali; it goes through the entire city.

[00:59:17] Amy: Then why does it not ((   )) with another part?

[00:59:27] Amy: Because, we can see that they are canals in every neighborhood here, but there are also other neighborhoods.

[00:59:33] Luz Angela: What happens is that this canal from—The city of Cali, right. And where does the city of Cali’s canal come from?

[00:59:44]

[00:59:46] David: That canal, from where I have seen it, comes from Navarro.

[00:59:59] David: That one that is there from fifty-six, you can see it from Navarro’s shore.

[01:00:04] David: It meets with this one and it comes from all of the city of Cali.

[01:00:09] Luz Angela: And the city of Cali ends in Córdoba.

[01:00:14] David: No, the city of Cali ends, ah from here in the south, yes. But, the in the north it ends there in that passage of the commerce.

[01:00:25] David: And there the canal ends in the Cauca river.

[01:00:28] David: But then there in Pertecuy they treat the residual waters and they throw them to—

[01:00:40]

[01:00:48]

[01:00:50]

[01:00:58]

[01:01:09]

[01:01:17]

[01:01:24]

[01:01:32]

[01:01:40]

[01:01:48]

[01:01:55]

[01:02:04]

[01:02:12]

[01:02:20]

[01:02:26]

[01:02:34]

[01:02:42]

[01:02:52]

[01:03:01]

[01:03:14]

[01:03:21]

[01:03:31]

[01:03:40]

[01:03:51]

[01:04:00]

[01:04:07]

[01:04:14]

[01:04:20]

[01:04:29]

[01:04:40]

[01:04:50]

[01:05:03]

[01:05:25]

[01:05:33] David: I think that the zone that affects this is the canal.

[01:05:38] Amy: But we still don’t know why the canal doesn’t affect other places. Well that...

[01:05:44] David: There, do you see how the ((cluster)) until where the spots are, the hot spots, right. And there is where you know that it is because of the canal

[01:05:53] Amy: Yes. Ah yes, ____ said something about the spots.

[01:05:57] David: Aha.

[01:05:58] Amy: Do they already have the spots, or no?

[01:06:00] David:Well um, of the – Dengue?

[01:06:07] Amy: Of the engineer, the directions, no?

[01:06:10] David: Ah, the ones the sir was going to do? Yes, he is doing the job.

[01:06:15] Amy: Ah, good.

[01:06:17] David: I think he will deliver them today.

[01:06:20] David: Yes, the town hall’s engineer.

[01:06:21] Amy: Can we stop for a moment?

[01:06:24]

[01:06:34]

[01:06:44]

[01:06:54]

[01:07:03]

[01:07:12]

[01:07:23]

[01:07:31]

[01:07:39] Luz Angela: There are various spots where they have been cleaning, that we have found people.

[01:07:45] Amy: But, before we did not see that, right?

[01:07:48] David: No.

[01:07:50] Amy: That is something that they were not doing a couple of months and weeks ago. It is new.

[01:07:53] Luz Angela: No. I think what the secretary is – She is deploying people to check it and give education in regards to Zika.

[01:08:06] Amy: That is very good.

[01:08:07] Luz Angela: Yes. They had ninety people, ninety people that I understood that had to come educate and--

[01:08:18] David: And fumigate too, I heard that they are training you in the stadium.

[01:08:22] Luz Angela: Look, there is another one in ((abril)) that they already opened, look.

[01:08:26] Luz Angela: Look at all the trash that they take out of there, look.

[01:08:29] Amy: But, do they leave it there?

[01:08:31] Luz Angela: I think they know, they come pick it up afterwards.

[01:08:34] David: Yes. They have to clean and the comes another car and picks it up

[01:08:39] Luz Angela: Look.

[01:08:40] Amy: Ah yes.

[01:08:41] Luz Angela: All of those--

[01:08:42] Amy: That was a risk before each time we saw one of the siphons- that they stayed dirty.

[01:08:53] David: The work is hard, yes or no?

[01:08:55] Luz Angela: Yes. Getting all of that filth from everyone out.

[01:08:58] David: No and not only that, but how that smells, is horrible.

[01:09:03] Luz Angela: Yes, horrible.

[01:09:04] And that lady working there ((       )) on that.

[01:09:07]

[01:09:18]

[01:09:27]

[01:09:35]

[01:09:43]

[01:09:51]

[01:10:00]

[01:10:02] Amy: And that sand that they have there, what’s the use for it?

[01:10:07] David: The what?

[01:10:09] Luz Angela: The sand.

[01:10:09] Amy: The sand.

[01:10:14] Luz Angela: Constructions that they will soon—That they do.

[01:10:23]

[01:10:38]

[01:10:46] Luz Angela: Look, event here they clean the-- The siphons.

[01:10:52] David: Is that they are many, look. They are also cleaning there. And they are resting.

[01:10:58]

[01:11:05]

[01:11:13]

[01:11:21]

[01:11:30]

[01:11:40]

[01:11:50]

[01:12:00]

[01:12:10]

[01:12:20]

[01:12:30]

[01:12:40]

[01:12:50]

[01:13:00]

[01:13:10]

[01:13:20]

[01:13:30]

[01:13:40]

[01:13:46] Luz Angela: That is the city of Cali.

[01:13:49] David: This on--

[01:13:50] Luz Angela: Which one is it?

[01:13:52] David: Is another one. This one goes through Marroquin.

[01:14:00]

[01:14:10] David: This is the twenty-eight. Right, Amy?

[01:14:13] Amy: Yes, twenty-eight D.

[01:14:14] Luz Angela: Ninety-nine.

[01:14:30]

[01:14:43]

[01:14:53]

[01:15:00]

[01:15:18]

[01:15:30]

[01:15:40]

[01:15:50]

[01:16:00]

[01:16:10]

[01:16:20]

[01:16:33]

[01:16:40]

[01:16:50]

[01:17:00]

[01:17:09]

[01:17:20]

[01:17:30]

[01:17:40]

[01:17:50]

[01:18:00]

[01:18:12]

[01:18:20]

[01:18:30]

[01:18:40]

[01:18:50]

[01:19:00]

[01:19:10]

[01:19:20]

[01:19:30]

[01:19:40]

[01:19:51]

[01:20:05]

https://drive.google.com/folderview?id=0Bza3s3VWuatLa3pHb2hDaVZfSGc&amp;usp=shar

ing&amp;tid=0Bza3s3VWuatLNEZGTHUwZWFWN0E

ZOOM0003 Cali Mio 1/29/16.WAV

Neighborhood: Cali Mio

Driver/vector control specialist: David

Interviewer: Amy

Community health worker: Luz Angela Peralta Valencia

Date: January 29, 2016

[00:01:00]

[00:02:00]

[00:03:00]

[00:04:30]

[00:04:26] L: Almost all of the houses has roofs made out of tin or can.

[00:04:36] A: The roof.

[00:04:36] D: The most economic for construction.

[00:04:43] D: And Cali Mio has a lot of passageways. Look

[00:04:49] D: Is impossible for us to get in there.

[00:04:59] D: There is a shortage in vehicular roads.

[00:05:03] L: Yes, just look at that. They are

[00:05:08] D: Just passage ways

[00:05:12] L: Look.

[00:05:25] L: And the roads are blocked, why? Only motorcycles can go through.

[00:05:36] D: This is the health post they are fixing it. Remodeling it

[00:05:42] A: So it is closed these days?

[00:05:43] L: Is closed.

[00:05:44] D: They are not giving service.

[00:06:00]

[00:07:00]

[00:08:00]

[00:08:44] D: Look, that is what we told you that people the culture that have is that where

they see trash they keep putting more.

[00:09:00]

[00:09:49] L: There are signs that tell you not to throw trash

[00:10:00]

[00:11:00]

[00:11:32] A: There&#39;s a sofa over there, there&#39;s a man going up there.

[00:11:38] L: What is it that&#39;s there? A motorcycle?

[00:11:42] A: That&#39;s a sofa.

[00:11:44] L: A sofa?

[00:11:45] D: A chair, yes.

[00:11:46] L: And the man sits there.

[00:12:00]

[00:12:14] L: That is like a canal, like a wetland.

[00:13:00]

[00:13:51] L: Oh that is debris. I though that it was a (( ))

[00:15:00]

[00:15:10] A: Tires, more tires. A lot of tires.

[00:16:00]

[00:16:50] L: A lot of debris and tires.

[00:16:53] D: Yeah, that people from there. And that is what that lady does just throw out

debris.

[00:18:00]

[00:19:00]

[00:20:00]

https://drive.google.com/folderview?id=0Bza3s3VWuatLa3pHb2hDaVZfSGc&amp;usp=shar

ing&amp;tid=0Bza3s3VWuatLNEZGTHUwZWFWN0E

ZOOM0004 Puerto del Sol 1/29/16.WAV

Neighborhood: Puerta del Sol

Driver/vector control specialist: David

Interviewer: Amy

Community health worker: Luz Angela Peralta Valencia

Date: January 29, 2016

[00:00:10] D: This neighborhood is not that new. It looks old.

[00:00:13] A: Old?

[00:00:15] L: But the streets are wide.

[00:00:19] D: Yes, the vehicular roads are better.

[00:00:21] L: Yes.

[00:00:23] L: Even thought it is dangerous.

[00:00:26] D: Yes. Even thought it is...

[00:00:28] L: Fire.

[00:01:00]

[00:02:00]

[00:02:39] L: Just Debris.

[00:02:42] L: That is the problem of Dengue here. Is that. I think that

[00:02:49] L: Look, they are tubes, things that contain water.

[00:02:53] A: That. Bottles, bricks...

[00:02:55] L: Bricks.

[00:02:57] A: That is a breeding site the tires.

[00:02:59] L: The tires.

[00:03:01] A: The streets.

[00:03:05] A: Is a breeding site street.

[00:03:07] D: Look at that little girl who is pregnant.

[00:03:11] L: Oh so heavy. That is sad.

[00:03:12] D: Correct.

[00:03:14] L: And the problem is with

[00:03:21] L: The problem is with those programs with youth in action

[00:03:26] L: That they give them money.

[00:03:28] D: Look at the street. Is just thieves.

[00:03:35] L: They give them money for the kids they have.

[00:03:40] L: And the girls get pregnant.

[00:04:00]

[00:04:43] L: And when there&#39;s rationing, they are directed to these neighborhoods, the

poorest.

[00:04:49] L: Neighborhoods that are more vulnerable, like this one. More poor.

[00:05:43] L: Look at the blocks.

[00:05:47] L: And when they leave them standing up, that becomes stagnant. Those that are up

should be

[00:05:53] L: full of water.

[00:06:00]

[00:07:00]

[00:08:00]

[00:09:00]

[00:10:00]

[00:11:00]

[00:12:00]

[00:12:51] L: Look, stealing water.

[00:12:54] D:Is that they took their water and they are getting it from there.

[00:12:57] L: Yes.

[00:13:00]

[00:14:00]

[00:15:00]

[00:16:00]

[00:16:15] A: No wait let&#39;s look at the canal. Because

[00:16:21] A: in various parts of the canal is different. Sometimes is very ugly and sometimes

[00:16:27] A: Is in movement. Here is good.

[00:16:29] D: Yes, it is in movement.

[00:17:00]

[00:18:00]

https://drive.google.com/folderview?id=0Bza3s3VWuatLa3pHb2hDaVZfSGc&amp;usp=shar

ing&amp;tid=0Bza3s3VWuatLNEZGTHUwZWFWN0E

ZOOM0005 Los Lagos 1/29/16.WAV

Neighborhood: Los Lagos

Driver/vector control specialist: David

Interviewer: Amy

Community health worker: Luz Angela Peralta Valencia

Date: January 29, 2016

[00:01:00]

[00:02:00]

[00:03:00]

[00:04:00]

[00:05:00]

[00:06:00]

[00:07:00]

[00:08:00]

[00:09:00]

[00:10:00]

[00:11:00]

[00:12:00]

[00:12:31] D: Look that is it there, right next to the puddle.

[00:12:39] D: This is what they are fixing of the canal.

[00:12:41] A: Oh, okay.

[00:12:44] D: The machinery is fixing the canal.

[00:13:00]

[00:14:00]

[00:15:00]

[00:16:00]

[00:17:00]

[00:17:45] D: The good thing about this lake is that it is small.

[00:17:48] A: Is small.

[00:17:51] L: But looking for it is hard.

[00:18:00]

[00:19:00]

[00:20:00]

https://drive.google.com/folderview?id=0Bza3s3VWuatLa3pHb2hDaVZfSGc&amp;usp=sharing&amp;tid=0Bza3s3

VWuatLNEZGTHUwZWFWN0E

ZOOM0006 1/29/16

Driver/vector control specialist: David

Interviewer: Amy

Community health worker: Luz Angela Peralta Valencia

Date: January 29, 2016

[00:01:00]

[00:02:00]

[00:02:50] D: That alley looks too horrible.

[00:02:53] L: Yes, that&#39;s to put us in for to much.

[00:03:00]

[00:03:20] D: The water is stagnant there, look.

Video: C:\Users\Amy\Google Drive\Kent\james\dissertation\chkv and dengue\data\video

data\january 30

ZOOM0001.Wav

Neighborhood: Mojica at [00:20:55]

Driver/vector control specialist: David

Interviewer: Amy

Community health worker: Luz Angela Peralta Valencia

Date: -- --

[00:00:00]

[00:00:32]

[00:01:03]

[00:01:31]

[00:02:00]

[00:02:30]

[00:02:59]

[00:03:30]

[00:04:01] D: Yes, we are here. Seventy-two S.

[00:04:30]

[00:04:39] D: Everything here has been an invasion, no?

[00:04:43] A: How long ago?

[00:04:46] L: This invasion has been going for more than thirty years.

[00:04:53] A: When does an invasion become a neighborhood?

[00:04:58] D: After ten years?

[00:05:00] A: Ten years.

[00:05:01] D: When they take over the houses, after 10 years of being there and nobody

has told them anything they take over.

[00:05:11] A: And then the (( comunio )) recognizes them as a neighborhood and they

give them K S and and the city services.

[00:05:26] L: They can start asking for rights.

[00:05:41]

[00:05:50]

[00:06:00] A: I change the photo so it&#39;s always in the front.

[00:06:10]

[00:06:22] D: This is called housing estate Los Lagos.

[00:06:30]

[00:07:00]

[00:08:00]

[00:09:01]

[00:10:03]

[00:10:04] L: The use of tires is everywhere, and the parks to plant, ((for healing)).

[00:10:24] A: That is Los Lagos.

[00:10:38] D: There are good people but there are also gangs that ruin the neighborhood.

[00:10:44] L: The gangs, they ruin the kids, the youth.

[00:10:53]

[00:11:03]

[00:11:19]

[00:11:55] D: Here in Comuna they have Charco Azul, which is the lagoon of El

Pondaje, no? Charco Azul is that way.

[00:12:12]

[00:12:31]

[00:12:59]

[00:13:28] L: That is a C D I, is a school.

[00:13:39] D: It was until there.

[00:13:42] A: Oh.

[00:13:49] L: Do we turn it off?

[00:13:50] A: Did we finish?

[00:13:50] D: Wait, let&#39;s finish over there.

[00:14:00]

[00:14:14]

[00:14:41]

[00:15:24] L: these streets are so thin.

[00:15:32] D: The one from Berejel are even more, they are like the ones in Poblado.

[00:15:49] D: Do you see the (( lote))

[00:15:51] A: Mhm.

[00:15:52] D: ((Well, when we get there )) there&#39;s also an empty toilet.

[00:16:00]

[00:16:30]

[00:17:00]

[00:17:20] D: I think we finished here.

[00:17:22] A: Oh Okay. D you know from where to where?

[00:17:25] D: You can turn it off in the corner.

[00:18:00]

[00:18:05] D: ((They should have put a tire on the side)).

[00:18:30]

[00:19:00]

[00:19:30]

[00:19:43] A: Should we ask the police, or something?

[00:19:51] L: I do not think so, right?

[00:19:59] L: We bring more attention that way.

[00:20:01] D: With the police.

[00:20:02] L: Yeah, with the police.

[00:20:03] A: Yeah, I think we bring more attention, but are you sure we want to go in?

[00:20:09] D: Well, let&#39;s go in and see how it is.

[00:20:12] A: Okay

[00:20:12] D: We will just keep going if we see is too dangerous.

[00:20:17] L: We have passed through a lot.

[00:20:20] A: Yeah, what happens is that at the beginning, ____ told me to a lot of times

to be careful with that.

[00:20:26] A: That it is one of the most dangerous from the list that we have and that is

why we were waiting on the hot spots to see if it was too necessary for us to go there.

[00:20:43]

[00:20:53] A: Well we are in Mojica. We are going to see what we find.

[00:21:06]

[00:21:15] L: Tires and more tires.

[00:21:30]

[00:22:01]

[00:22:06] : I cannot believe what-- I used to come here around were, I walked alone.

How I exposed myself.

[00:22:15] D: And the little ones that I think they sent to the store. What a sin. Girls so

small I would not send them to the store.

[00:22:26] D: Something happens to them in the streets that are going give me a bad

conscious.

[00:23:00]

[00:23:30]

[00:24:00]

[00:24:30]

[00:25:00]

[00:25:30]

[00:26:00]

[00:26:30]

[00:26:42] D: We are on the thirty-nine with twenty-eight. We are around here. We are

going to- this part, the eighty-three is an avenue of Cali City.

[00:26:49]A: Okay.

[00:26:50]D: You know Mojica has this part here and has all of the part from that side.

[00:27:01]

[00:27:32]

[00:27:59]

[00:28:31]

[00:29:00]

[00:29:14] Here we are. Twenty-eight D with seventy-six. We are here. This corner.

[00:29:30]

[00:29:40] D: Look at the trash.

[00:29:40] A: Mhm.

[00:29:41] D: There&#39;s the canal. It is an entry that they have made to take out of the trash.

[00:29:46] A: Take out trash from the canal.

[00:29:48] D: That the same people throw there.

[00:29:51] A: Who takes it out?

[00:29:53] D: The town in in charge of taking it out. With the (( )).

[00:30:00] L: But they don&#39;t withdraw it if not they leave it there.

[00:30:17]

[00:30:31]

[00:30:34] A: But there is no river, right? So that water comes from the houses or what?

[00:30:39] L: No, there&#39;s the rain.

[00:30:39] D: Yeah, it rained.

[00:30:44] A: Ah yes.

[00:31:19] D: This is the safe part of Mojica. The dangerous part is on the other side.

[00:31:25] A: We did the other side.

[00:31:30] D: The other side is very dangerous, the Mojica side. Here it is divided in two.

[00:31:37] A: Yes but, have we done the other side, that small part?

[00:31:43] D: No, we haven&#39;t done it.  We are barely covering this part here since the

seventy-six to the eighty-three, which is the avenue of Cali City.

[00:31:53] D: We have to pass to the other side.

[00:31:55] L The hard one.

[00:31:57] D: This part right here is the very dangerous one.

[00:32:06]

[00:32:30]

[00:33:00]

[00:33:30]

[00:34:01] L: It is really hard to drive here.

[00:34:30]

[00:35:00]

[00:35:30]

[00:36:00]

[00:36:30]

[00:37:00]

[00:37:30]

[00:38:00]

[00:38:04] A: I don&#39;t feel like we are good in this neighborhood.

[00:38:11] D: Like what?

[00:38:12] A: Like we are good here in this neighborhood.

[00:38:14] L: She doesn&#39;t feel like we are good here.

[00:38:16] D: Safe.

[00:38:17] A: That, I don&#39;t feel good here. That was the first time I did not feel good here.

[00:38:24] L: Then, let&#39;s go because...

[00:38:26] A:  And yes, it is not worth it.

[00:39:00] D: Here is where all the gang members were with knives, remember?

[00:39:08] A: Yes I remember.

[00:39:17] D: Because we did the orchard that is right here.

[00:39:30]

[00:39:41] A: We already know that the same risk everywhere is here and there&#39;s life

risks here too. Then, let&#39;s go to another one of the one&#39;s we have and that&#39;s it.

[00:39:57] A: Because the intuition is strong.

[00:41:01] L One moment, let&#39;s see that. Hatcheries, old wood...

[00:41:35]

[00:42:00]

[00:42:30]

[00:43:00]

[00:43:11] D: See, in Mojica everything is uncovered.

[00:43:15] A: Mhm (( Like ... hurried)) correct? Everything uncovered.

[00:43:19] D: Yes.

[00:43:24] A: And everything...

[00:43:38] D: There was another dangerous spot. From E to F. There&#39;s the guys standing

there seeing whom they are going to rob or harm.

[00:44:00]

[00:44:11] D: This one is the twenty-nine that is that-- This way is Comunero, the part

that is not that ugly. The other one is over there and it is horrible.

[00:44:22] A: Yes.

[00:44:24]  D: Look at that. It is like ranch.

[00:44:26] A: We will not get in there.

[00:45:00] D: Twenty-nine with fifty-four. We are here. Let&#39;s do this part here, Amy.

[00:45:05] A: Okay. Ready.

[00:45:07] D: Yes, this is the eighty third street that is the Cali city and on the  other side

it is the invasion, which let&#39;s to this side which is better.

[00:45:12] A: That? Okay. Let&#39;s do it quickly and that&#39;s it. We will be done.

[00:45:19] A: We are in the Comunero and we will do the part that it is not the invasion.

[00:45:31] A: Because of the danger.

 [00:45:34] D: yeah, that other side is horrible.

[00:45:39] D: All of the entry ways are invasion then further down is even uglier, because

it is in the countryside. It is ranches that they have made that way.

[00:45:49] Behind the Isaias Duarte Hospital, they have invaded it all.

[00:45:55] D: But they are in search for the government to help them with subsidies and

for them to get relocated. Then, they buy those pieces of land for cheap.

[00:46:04] D: A piece of those lands do not cost them more than two

hundred thirty thousand dollars. And they obviously-- They are ones that come back, the

owners of bigger lands and they sell smaller pieces to others.

[00:46:19] D: So they can make they&#39;re houses and little ranches so they can be put in

subsidies.

[00:46:55]

[00:47:30]

[00:48:30]

[00:49:30]

[00:50:30]

[00:51:30]

[00:52:30]

[00:53:30]

[00:54:30]

[00:54:32] A: Well I think we will see a hot spot in Melendez. A hot spot in ((Valle de

Leny)). And around here I don&#39;t know, because everything is a spot. It is going to be...

[00:54:47] D: All of them are going to be here first. It will be a cluster..

[00:54:49] A: I don&#39;t know what is going come out of here because since everything is

close...But at least we will see three spots in the map.

[00:55:03] D: Yeah, and in all places. At least in Calipso where they are many.

[00:55:07] A: Yes, in Calipso Trece.

[00:55:08] D: There has to be a cluster there.

[00:55:10] A: Yes.

[00:55:14] A: In Calipso.

[00:55:26] A: When everything is together and well prettier, the map put together, it will

be great.

[00:55:37] D: So great that you will see everything that is finished in the map.

[00:55:41] A: Yes.&#39;

[00:56:00]

[00:56:30]

[00:56:59] D: That is the other side of Mojica.

[00:57:00]

[00:57:20] D: You see how are all invasions?

[00:57:22] A: Yes. Everything is very disorganized.

[00:57:28] D: Yes, look at the streets.

[00:57:32] A: Yes, it is very easy to see.

[00:58:00]

[00:58:30]

[00:59:00]

[00:59:30]

[01:00:00]

[01:00:30]

[01:01:00]

[01:01:30]

[01:02:00]

[01:02:30]

[01:03:00]

[01:03:30]

[01:04:00]

[01:04:30]

[01:05:00]

[01:05:30]

[01:06:00]

[01:06:30]

[01:07:00]

[01:07:30]

[01:08:00]

[01:08:30]

[01:09:00]

[01:09:30]

[01:10:00]

[01:10:30]

[01:11:00]

[01:11:30]

[01:12:00]

[01:12:30]

[01:13:00]

[01:13:18]

END

ZOOM0002.Wav

Neighborhood: Manuel Beltran

Driver/vector control specialist: David

Interviewer: Amy

Community health worker: Luz Angela Peralta Valencia

Date: -- --

[00:00:10] A: Well, we will cover Manuel Beltran, to see what we can find in the more

safer area.

[00:00:30]

[00:01:00]

[00:01:30]

[00:02:00]

[00:02:30]

[00:03:00]

[00:03:20] D: Manuel Beltran Neighborhood

[00:03:36] L: Look, a lot of tires.

[00:03:40] L: We will have to ((go away)) from the tires.

[00:04:00]

[00:04:11] L: This looks like the main street.

[00:04:14] D: Yes, this is one of the main streets. There is this one and the twenty-seven

which is where it ends.

[00:04:18] L: And it does not look that bad.

[00:04:20] D: But the ugly thing is-- look at the police station. The ugly thing about this

is the canal&#39;s shore.

[00:04:28] D: ((Look there)) so you can see where the canal begins.

[00:05:00]

[00:05:30]

[00:05:55] L: This one is not as ugly as Mojica&#39;s. This one is more wider and bigger.

[00:06:01] D: No, it has it&#39;s side.

[00:06:11] L: It is more like a city. Is less ugly.

[00:06:30] L: Very broad streets.

[00:06:37] L: But it also is, the main ones are bigger and the...

[00:06:47] D: This is a pretty tree, is a Ceiba.

[00:06:54] L: Very pretty, yes.

[00:07:05] D: We went through the canal street very quickly. So you could record.

[00:07:09] L: Yes yes, there was nothing there

[00:07:13] D: (( The older ones were playing there on the side.))

[00:07:30]

[00:08:00]

[00:08:03] L: Look at that.

[00:08:05] D: Look at all the trash there. I&#39;m trying to see if there is any rats there.

[00:08:09] L: Look at all the things filled with water. And it does mattresses there should

be water stagnant there, right?

[00:08:24] D: ((Where ever it wants to stay still...))

[00:09:00]

[00:09:30]

[00:10:00]xx

[00:10:30]

[00:10:49] D: The ugliest one was the Mojica one.

[00:10:54] L: And Berjel.

[00:10:59] D: Also, part of Comunero is horrible. Since it  has the yellow colony.

[00:11:05] L: Oh no, is horrible.

[00:11:10] L: Charco Azul.

[00:11:13]  D: Yes, it is horrible there.

[00:11:30]

[00:12:00]

[00:13:00]

[00:13:30]

[00:13:54] D: That is the (( )) for the canal. It is close, why?

[00:14:00]

[00:15:00]

[00:15:13] L: What are they doing?

[00:15:16]  A: Recycling?

[00:15:33] L: It is livelier here.

[00:15:35] L: More social interactions.

[00:15:38] D: Well yeah. It is because this neighborhood does not let kids do anything.

[00:15:43] D: Only streets.

[00:15:45] L: Doing what the teens do.

[00:15:48] D: Taking bad examples.

[00:16:30]

[00:17:00]

[00:17:30]

[00:18:30]

[00:19:30]

[00:20:01] D: I think what Manuel has is a canal. If you want we should just go up there.

[00:20:20] A: But where the canal is, let&#39;s mark it as risk crossed with poverty. Because a

canal without poverty is not much risk.

[00:20:33] But when it is crossed with trash where they do not pick it up there&#39;s risks.

[00:20:43] D: Look.

[00:20:46] D: Look what the small canal has.

[00:20:53] L: That one is crossed with poverty.

[00:20:56] A: Yes. That is what we will do in the map is put the levels of the

neighborhood with the canal and there I will see where there&#39;s more risk for Dengue.

[00:21:11] A: Because where there is people picking up the trash everyday, there&#39;s no

cases.

[00:21:30]

[00:22:00]

[00:22:38] L: But the canals here, almost all of them coincides with, well I don&#39;t know,

with poverty.

[00:22:49] D: Yes.

[00:22:50] A: Yes?

[00:22:52] L: It is to say that Oriente is the poorest city Cali has.

[00:22:57] L: It is the biggest zone and has the biggest population.

[00:23:03] A: In the places where there are no canals how do they gather the rainwater.

[00:23:14] L: Where there&#39;s no canal? Well isn&#39;t there rainwaters? Canals of rainwater.

[00:23:20] D: Canals of rainwater.

[00:23:23] A: But the canals have the same risk, or not?

[00:23:30] D: No.

[00:23:30] A: Because they are cleaner?

[00:23:32] D: Because where it is rain, there&#39;s movement.

[00:23:47] D: This is where it ends, Amy.

[00:23:50] A: Okay.

Audio File: C:\Users\Amy\Google Drive\Kent\james\dissertation\chkv and dengue\data\video

data\Lili_nov27\ZOOM0002.WAV

Associated video files:

1. C:\Users\Amy\Google Drive\Kent\james\dissertation\chkv and dengue\data\video

data\Lili_nov27\FILE0223.MOV

a. Transcript: [00:00:29] Santiago: Tell me something, I am asking as a taxi driver.

Normally, not all but many, taxi drivers, you can see them doing their business in

the weeds. If you they urinate, if they do their necessities of urinating or

whatever, that doesn&#39;t attract the mosquito?

b. Video: [00:01:04]

2. C:\Users\Amy\Google Drive\Kent\james\dissertation\chkv and dengue\data\video

data\Lili_nov27\FILE0264.MOV

a. Transcript: [00:00:29] Santiago: Tell me something, I am asking as a taxi driver.

Normally, not all but many, taxi drivers, you can see them doing their business in

the weeds. If you they urinate, if they do their necessities of urinating or

whatever, that doesn&#39;t attract the mosquito?

b. Video: [00:01:04]

Neighborhood: Lili

Date: November 27, 2015

Interviewer: Amy Krystosik

Driver, local resident: Santiago

[00:00:29]

Santiago: Tell me something, I am asking as a taxi driver. Normally, not all but many, taxi

drivers, you can see them doing their business in the weeds. If you they urinate, if they do their

necessities of urinating or whatever, that doesn&#39;t attract the mosquito?

[00:01:14]

Amy: No

Santiago: Look over there, there is only vegetation

[00:01:19]

[00:01:28]

[00:01:43]

Santiago: Everything over here is new

Amy:  yes beautiful. they are building everything

Santiago: But very expensive

[00:02:02]

[00:02:53]

Amy:  but here also I see that the people can be reporting more

Santiago: They don&#39;t report

Amy:  I think it can be that they are reporting more than in other neighborhoods. because maybe

they are visiting the doctor more or I don&#39;t know what. if they have the flu and they take the test

and for that reason they report more.

Santiago: this doesn&#39;t have anything to do with it because many times the people travel a lot to

other departments and can bring it from there

Amy: Yes, that too.

[00:03:33]

Amy: Also, that man over there is taking his dog out and the mosquitoes are biting him

[00:03:43]

Santiago: a lot of people go out like him to take out the dog but they don&#39;t take the bag

Santiago: This is the last one here. Over there they haven&#39;t built anything.

Amy:  I think this has something to do with it because there are a lot of mosquitos over there in

the limit

Santiago: and when they let the weeds grow, it worse.

[00:04:13]

Santiago:

These [weeds] creates the conditions to provide more humidity in the bush.

[00:04:25]

They closed the wall because one time this all flooded.

[00:04:26]

The water comes out of the stream. Eight years ago all of this flooded.

[00:04:38]

Amy:  eight years ago

Santiago: over there is a type of stream. A small river that when it grows. That why they made

the wall so tall so they could construct. No, I lived in this neighborhood 20 something years ago.

[00:04:50]

Amy:  You lived here in this part

[00:04:54]

Santiago: here no. In the end there in the complex apartments.   I have lived over there for 25

years. In that time there was none of this built.

[00:05:04]

Amy:  Yes, they have told me that this was a landfill

Santiago: the landfill was over there in the end but they took that out about eight years ago and

from there to here they started to construct because with the bad smell the people weren&#39;t buying

00:05:25]

Amy:  but here it looks expensive

Santiago: No, here you can buy for 80-100 million pesos. Maybe there is another range between

120-150.

[00:05:48]

The first ones there, the first apartments, 80 million.

[00:06:00]

Here I would say 120.

[00:06:05]

[00:06:31]

[00:07:18]

Santiago: here they have built a lot

Amy:  Yes, it’s growing

Santiago: Here there is a way to sell to the people cheaply and the people are tricked. They sell it

with gray walls and you have to finish the walls, paint, closet, doors, stove. And this cost a bit of

cash and the people don&#39;t assume this. They sell it to them at 80-90 and they don&#39;t take into

account the millions.

Amy:  No, it’s not easy

[00:08:39]

[00:09:00]

[00:09:19]

[00:09:43]

Santiago: these places are dangerous for a newborn?

Amy:  why?

Santiago: if a mosquito bites him?

Amy:  Oh, yes, for the dengue. Yes. It&#39;s important to use, for example, a bed net for the baby

Santiago: A what?

Amy:  A bed net

[00:10:19]

Santiago: I have a client here. They had a sick child but they didn&#39;t know what it was. I was

talking with the lady.

[00:10:25]

Amy:  And here they are constructing more?

Santiago: yes.

[00:10:29]

Amy:  This is the limit.

[00:10:38]

Santiago: all of this is to construct.

[00:11:15]

[00:11:43]

Santiago: there ends the construction. Look how the have constructed here.

[00:11:52]

Santiago: here it ends

[00:12:06]

[00:12:33]

Santiago: but you can see that there is no standing water nor anything. Not on the side of the

road. Nor trash. This is a high social strata neighborhood. They take good care of this.

[00:12:41]

Santiago: these are under construction. fixing the apartments.

[00:13:08]

Santiago: I am going to show you another part where there is more bush.

[00:13:46]

[00:14:43]

Santiago: These lots that say Comfandi are because they are going to construct.

Amy:  yes, they are working there.

[00:16:12]

Santiago: I don&#39;t know if it is important here. Here came people from popular (poor)

neighborhoods. a neighborhood that Comfandi built with compensation at the end that I want to

show you too. I don&#39;t know if that has something to do with it. There are people from popular

neighborhoods.

Santiago: illness doesn&#39;t respect social strata

Amy:  no, the mosquito neither.

[00:19:35]

Amy:  what is this? ESP clinic?

Santiago: No, this is from the police. The police clinic they call it. It is new. It’s already working.

Emergencies for the back.

Amy:  and what’s it called?

Santiago: police clinic. For the police, soldiers.

[00:20:01]

Amy:  This could also be. The soldiers have dengue, the mosquitos are biting them and

transmitting it in the neighborhood

[00:20:10]

Santiago: Here come a lot of soldiers from the department of Cauca. They are in military service

here. Or many give military service here and are from there, the towns of Cauca. From

Santander. The have a month or 15 days when they bring them.

[00:20:37]

Amy:  and here how is the construction inside? Like Valle de Lili Hospital with everything open

with plants inside?

[00:20:44]

Santiago: No, I don&#39;t know how it is inside. The plants they try not to keep in the hospital much,

who knows.

[00:22:04]

[00:23:59]

Santiago: I live five minutes from here

Amy:  this is a beautiful neighborhood

Santiago: this is yes; you don&#39;t get expensive houses here. I would say 150 million pesos. This

one I don&#39;t like because it’s the third social strata. I only like up to second level.

[00:24:24]

Here the apartments are expensive. 200 million.

[00:24:30]

Directory: C:\Users\Amy\Google Drive\Kent\james\dissertation\chkv and dengue\data\video

data\Melendez_nov27\

Audio File: ZOOM0001.WAV

Associated video files:

1. FILE0222.MOV

a. transcript: [00:01:15] Amy: Do you know what Melendez has to have so much

dengue. That&#39;s why I am interested in Melendez

b. video: [00:02:15]

2. FILE0263.MOV

a. transcript: [00:01:15] Amy: Do you know what Melendez has to have so much

dengue. That&#39;s why I am interested in Melendez

b. video: [00:02:12]

Neighborhood: Melendez

Date: November 27, 2015

Interviewer: Amy Krystosik

Taxi driver, local resident: Santiago

[00:00:43]

[00:01:15]

Amy:  Do you know what Melendez has to have so much dengue. That&#39;s why I am interested in

Melendez

Santiago: Well, the thing that happens, maybe, I see the same thing everywhere- accumulation of

standing water. In the trash they leave. In batteries or tires o en the trash. As they have it in any

part, if they don&#39;t take it on time, you see the accumulation of mosquitoes, mosquitoes.

Amy:  But more here than in other parts or what?

Santiago: No, I think it’s the contrary. Here at least I think the people have learned, I don&#39;t know

if I am wrong but they have learned to clean up more.

[00:01:59]

Amy: Yes, it seems to be very organized here. Then I don&#39;t understand why this neighborhood

precisely has more dengue compared to many others in other parts. And it is very strange

because in the immediate area the other neighborhoods don&#39;t have any. It is this neighborhood

and no more.

[00:02:23]

Santiago: And now for a long time it has been summer- it hasn&#39;t rained. They say that dengue

follows the accumulated waters. Or foods that they leave out and throw out. This attracts

mosquitoes- the food residuals. Instead of giving it away I suppose that they throw it away there.

Throw away- it’s to say for here nobody&#39;s coming, we throw it out. This food attracts these small

animals.

Amy:  Here there are many tires- but they are there protected under the roof

Santiago: Look

[00:02:58]

Santiago: Look, this is a good place

Amy:  Yes, it is very well organized.

[00:03:03]

Santiago: Up there it is not too bad for a hill. This is one of the hills that we are going down. We

have almost started the hill.

[00:03:12]

Santiago: from here up is hill now.

[00:03:16]

[00:04:23]

[00:06:14]

Santiago: But it seems to me that this part where they were talking about lots of mosquitoes is

the best part.

Amy:  Yes, it’s very strange, no.

Santiago: It seems strange to me.

[00:06:26]

Amy:  Or maybe it’s that the people here go more to the doctor to revise their situation and that&#39;s

why there are more cases reported than in other parts but maybe its equal.

[00:06:48]

[00:08:43]

Santiago: How strange it is that over there exist the mosquitoes. Or I don&#39;t know if it is the

soldiers who transmit it who come from other parts. As here there are many soldiers from Cauca-

they give them permission.

Amy:  this could be. That they are traveling and come with dengue

 Santiago: and they pass through the neighborhood- maybe they have contact with the people.

Amy:  Yes, and the mosquitoes are biting and transmitting between one and the other.

[00:09:16]

[00:09:28]

Amy:  And there in the front is the battalion

Santiago: That is the battalion. The dispensary where they have the doctors there. Now I will

show you over there where we can see the other. It’s that sometimes the transmission happens

sometimes when the people come from other regions.

Amy:  Yes

Santiago: and here there are many women who fall in love with keys or many they call service

employees who have a lot of contact with the soldiers. They sleep with them. The cultural- they

are many who like the soldiers. To sleep with them or to have them as lovers or boyfriends. What

do I know.

Amy:  Yes, there is also a lot of syphilis in this neighborhood.

Santiago: Look, people don&#39;t respect their bodies. I don&#39;t see it as a disease but as not respecting

your body. They maintain contact with, many persons visit prostitutes. Places of prostitution.

[00:10:36]

Thanks to god [] with mine I have a lot of responsibility.

[00:10:44]

[00:11:06]

Santiago: You told me it was the best part if not maybe it’s the people that there is not a

good behavior in the people.

[00:11:37]

Santiago: This is the part of the battalion that I told you. Over there is another four that is in the

hospital unit that is very good. That was a unit of my brother.

[00:11:49]

Amy:  Yes, maybe you are right that it’s the battalion that is transmitting there.

Santiago: Yes, I don&#39;t see anything else because there are people here who have years living here.

It’s not a social stratum so popular (poor). These are traditional people from Cali.

[00:12:39]

Santiago: Look, this is the dispensary that I told you about, look. Then that&#39;s where I see the

contact that make the transmission that there are people who come...

Santiago: What I see is the people who come to visit their family or those who have contact with

the service employees.

[00:13:18]

Santiago: and they give it to them

Amy:  But also the mosquitoes don&#39;t respect the fence there. The mosquitoes pass there, bite the

soldiers, and then go out and bite the people.

[00:13:35]

Santiago: Oh yes, there goes the transmission when it sees someone, it gets on the mosquitoes,

and it bites another.

[00:13:42]

Amy:  The mosquitoes are flying happily between the neighborhood and the ...

[00:13:48]

Santiago: The problem I see is here, the soldiers. Here come many soldiers from Cauca, Willa,

Putumayo. The give them rest every month or every month I don&#39;t know how often they give

them permission.

[00:14:01]

Amy:  And here they come to rest?

[00:14:05]

Santiago: No, they go out to home and come back here to take their post as a solider. The

permissions. That&#39;s the only thing I see looking at it from the culture.

[00:14:16]

And maybe they have lots of contact with employees as there are many girls that clean the

apartments and the houses. And there are many girls who have contact with them or they get

them as lovers or boyfriends.

[00:14:30]

Santiago: And as today people sleep together very quickly, there is the sexual contact. I don&#39;t see

where else it could come from.

[00:14:37]

Because this is the best part where you are putting me is the best part of Melendez, this section.

Up there is the area poorer where we were.

[00:14:45]

Santiago: I am going to show you up there another part.

[00:15:08]

[00:15:43]

[00:15:55]

Santiago: Look these are very good high schools. Not just any poor person can enter in there.

Amy:  American high school, yes. Yes, for that reason I thought it was strange too that this

neighborhood had dengue and the neighbors don&#39;t have it. Very strange.

[00:16:10]

Santiago: The transmission the mosquito makes when maybe the bite one of these people. And as

sometimes the soldiers do surveillance. Then be careful. Here lives my brother for about ten

years.

[00:16:31]

Amy:  Very beautiful.

[00:16:40]

[00:16:54]

Santiago: look these are parts where you wouldn&#39;t think would bite much the mosquitoes. This

trash I don&#39;t think will take long for them to pick up.

Amy:  No

Santiago: What happens is sometimes the recyclers take off the top it and leave it open

[00:17:06]

[00:18:01]

Santiago: then the thing can be that its next to the battalion. I don&#39;t see more.

[00:18:08]

Amy: I saw some tires here that could also be breeding sites

[00:18:13]

Santiago: and many times, forgive the expression, but sometimes many of these employees do

with their relations marijuana. Marijuana where there is bush where nobody sees them.

[00:18:44]

[00:20:47]

Santiago: These are very good parts

[00:20:47]

Amy:  Yes, you are right that it could be the battalion.

[00:20:50]

Santiago: What happens, you know what else could happen. That many people who live up there

in what I though was Melendez are reporting in a health post in Melendez. And maybe. I don&#39;t

know.

[00:21:03]

Amy:  But I am not using the health posts. I am using the home address.

[00:21:15]

Santiago: But how could it would be to do home visits to see who has contact with who or what

because for me the neighborhood.... or I don&#39;t know if the mosquito comes from the hill or from

other parts. I don&#39;t know.

[00:21:29]

Amy:  mosquitoes can reproduce in plants or in tires. They don&#39;t need a lot of water. Or in this

tree. They are very well adapted.

[00:21:43]

Santiago: The storm drains. Maybe there they don&#39;t clean well and there collects the dirt and

trash and they don&#39;t clean them on time.

[00:21:57]

Amy:  Yes, because this mosquito doesn&#39;t like anything clean. If there is water and its dirty,

perfect.

[00:22:07]

Santiago: Then there where there is rain water. It could be that they don&#39;t clean it well. I think

that the trash they collect quickly. They collect it the same week. Because if no the people jump.

[00:22:17]

Santiago: here they collect the trash twice a week, minimum. If not its these storm drains.

[00:22:30]

Santiago: They have to clean these constantly. Like in all the neighborhoods.

Amy:  yes, the storm drains are in all the neighborhoods. The strange thing is that here there is

transmission.

[00:22:50]

Directory:

C:\Users\Amy\Google Drive\Kent\james\dissertation\chkv and dengue\data\video data\nueva

floresta nov 26\Camera_8

Audio File: NuevaFloresta_nov26.wma

Associated video files:

1. FILE0013.MOV

a. Transcript: [00:01:12] Amy: we are in Nueva Floresta, 26 of November. We are

looking for, more or less, the risks of dengue and chikungunya because here there

are many cases of chikungunya and dengue too.

b. Video: [00:01:16]

2. FILE0262.MOV

a. Transcript: [00:01:12] Amy: we are in Nueva Floresta, 26 of November. We are

looking for, more or less, the risks of dengue and chikungunya because here there

are many cases of chikungunya and dengue too.

b. Video: [00:00:44]

3. FILE0221.MOV

a. Transcript: [00:01:12] Amy: we are in Nueva Floresta, 26 of November. We are

looking for, more or less, the risks of dengue and chikungunya because here there

are many cases of chikungunya and dengue too.

b. Video: [00:01:11]

Neighborhood: Nueva Floresta

Date: November 26, 2015

Interviewer: Amy Krystosik

Driver, vector control specialist: David

[00:01:12]

Amy:  we are in Nueva Floresta, 26 of November. We are looking for, more or less, the risks of

dengue and chikungunya because here there are many cases of chikungunya and dengue too.

David: In the neighborhood of Nueva Floresta

[00:01:33]

[00:02:03]

David: The neighborhood of Nueva Floresta has some parts with sewers or canals of residual

waters. Then this could also be [] . . . the mosquitoes

[00:02:39]

David: []

[00:02:51]

David: [] residual water here.

[00:02:54]

David: this is the division for the neighborhood.

[00:02:57]

Amy:  This water canal

David: It’s the canal of residual water. This I think is shared between residual water and rain

water. All of this goes to a principal canal that is over there on the Avenue Ciudad de Cali.

[00:03:20]

David: and these residual waters eventually go to a canal called the La Petal- The Treatment

Plant for residual waters.

[00:03:27]

David: and there they recuperate as much as they can the water and then the throw it into the

Cauca River.

Amy:  But the water is not moving much.

David: In this one no. Only when there are epochs of rain, you see lots of movement.

[00:03:41]

David: That&#39;s why for example I think there are stagnant parts.

[00:03:46]

David: as they say the limit of the neighborhood is divided by this canal.

[00:04:04]

Amy:  but the neighborhood is very organized.

David: yes, it’s not ugly

Amy:  No, you don&#39;t see any trash or people in the streets

David: No homeless people

[00:04:27]

David: this neighborhood is not too big

Amy:  this is interesting too because if it’s so small and they have so many cases, the density

should be very high.

[00:05:21]

Amy:  What do the people do here, do you know?

David: the people here are laborers normal. It’s a neighborhood of social stratus more or less 2.

The bills and life are not too expensive. They have a medium level of life.

[00:05:59]

[00:06:48]

Amy:  here is a garden of mosquitoes

David: This is the fault of the company of the city in charge of cleaning and field maintenance.

[00:07:06]

David: it is very unkempt here for the development of the image of the community. At least in

other communities there is more management and they maintain it constantly [].

Amy:  sometimes the holes in the trees are mosquito breeding sites

David: yes, and sometimes when it rains or the night [], it leaves water droplets in the field and

they there [] in the water that is left there. and who knows how much public health controls the

breeding sites.

Amy:  Fumigation. You do that right?

[00:08:06]

David: Yes, I do fumigation.

Amy:  Then yes, you are an expert in that. Tell me how it is.

[00:08:08]

David: The fumigation is- I do control of plagues and in this service they do also the control of

vectors. Apply larvicides in the breeding sites and do the control of the adult []. But the [] the

people do physical control to [] mosquito.

Amy:  Like bed nets.

David: Yes, like a screen. They put a screen so they don&#39;t enter in the house.

Amy:  Yes, also

[00:08:53]

David: This is the health post of nueva floresta.

[00:08:58]

Amy:  but these screens the people don&#39;t like because they think they are ugly, no

David: yes

Amy:  this I don&#39;t understand.

David: it’s like, they don&#39;t like it aesthetically because they see it as ugly but the reality is that

here in Cali they use them a lot in the rural areas. They put them outside and they put them to be

able keep it open and get a bit of air.

[00:09:30]

Amy:  then they contract you to do control in neighborhoods or what?

David: in houses, in residential units

Amy:  more private

David: a lot of service. and here I give also a bit of education to the people

Amy:  of course, that&#39;s great

[00:09:59]

[00:11:50]

Amy:  the lake too had a lot of cases

David: Los Lagos, the neighborhood. It’s that the lake is next to a neighborhood called Los

Lagos. Los Lagos has a problem- they have a type of lagoon that is very old and [].

Amy:  and its very big the neighborhood?

David: Yes, it’s on the other side but very close to Nueva Floresta.

Amy:  yes, let’s go there next to see it if we can

David: yes, it’s close. We go around Nueva Floresta and pass for over there?

Amy:  Yes.

[00:12:40]

David: and there is another part over there by the lakes called El Pondaje. The part over there [].

It has a Laguna that all its life has been []

[00:13:15]

Amy:  Yes, it has a lot of cases too. But there it is safe?

David: To pass it, yes. There we can pass.

Amy: Yes, because it is all related it’s not just an isolated neighborhood, it’s all related. And it

makes a lot of sense- the mosquitoes here

[00:13:39]

David: I think that there is [] the aqua duct []

[00:13:55]

Amy:  do you know the Laguna de Sonso, outside of Cali?

David: yes, I think so.

Amy:  that place was incredible as a breeding site. Terrible.

David: I think it’s like Charco Azul, have you gone there?

Amy:  Charco Azul is, have you gone there?

Amy:  No, never

David: No, Ok, we will go there too. and Los Lagos too, El Pondaje, the invasion of La Laguna

[00:14:46]

David: [] It has a canal of residual water []

[00:15:11]

David: Charco Azul is modifying the []. They have done some things

Amy:  Oh, they are trying to fix it but it hasn&#39;t worked yet. Do you know what works great?

Those fish that eat the larva.

David: Yes, that’s what I told Paola- they should turn it in to a fish farm. But in reality the people

around are a people []

Amy:  But this could do the government too

David: A lot of people have tried but [] they take them out of one part and they go to another part

Amy:  That&#39;s what Paola told me. There are various- Sucre they are going to knock down. And

then were are the people going to live?

[00:16:25]

David: [] in another place

[00:16:35]

David: [] Sucre with Paola

Amy:  Yes, that&#39;s what she told me. That was her homework, right?

David: we went over there [] everything there was []. Materials [].

[00:17:28]

David: []

[00:17:46]

Amy:  There was what?

[00:17:55]

David: Alfonzo, the people say []

[00:18:06]

[00:18:31]

David: I know the city because ten years ago I worked as a home delivery for a company.

Amy:  Yes, you know the neighborhoods and what they have. Valuable.

David: Yes, and I know all the addresses of the city because I always had to look for them to

bring the services.

[00:19:01]

[00:19:31]

David: for all of the Avenida Ciudad de Cali there is a canal of residual waters. It [] for all the

city

Amy:  And it’s not possible to control the mosquitoes in the canal?

David: they should do the control there but I think it’s like they would spend to many resources

in larvicides in all the canal in all the city.

[00:20:10]

David: the neighborhood Los Lagos []

[00:20:32]

David: [] remember that the [] the canal [] and it filled the houses

Amy:  a flood

David: [] residual water.

Amy: dirty water.

David: []

[00:21:21]

David: []

[00:21:28]

[00:21:50]

[00:23:15]

David: Look another canal of rain water. There goes to there to the canal of the Avenida of the

Ciudad de Cali.

Amy:  Ok, the water is not moving much and there is a lot of vegetation here too.

David: yes, it’s bad here.

[00:23:30]

David: this canal, in the rainy season, is full and all of these houses are full of rain water.

[00:23:36]

David: Paola and I have gone all over looking for patients but nothing has ever happened. One

day the police stopped us. []. We told him we are looking for a patient.

[00:24:45]

[00:24:58]

[00:25:31]

David: this is the canal of the Avenida Ciudad de Cali. And this canal traverses all of the city.

From North to South. It arrives to a place called La PETAL The plant of treatment of residual

waters. [] from this water and then they throw it in the River Cauca because the River Cauca is

more over there. All of the water that comes here they take the plant for treatment over there.

[00:25:59]

Amy: [] in all the city but it doesn&#39;t make more risk in all the parts then it doesn&#39;t make sense.

[00:26:27]

Amy:  it’s strange that in some parts it doesn&#39;t []

David: It’s that also here before the public health had a greater importance to the people.

They taught them, they went to the houses, [] the stagnant water [], [] the water. All this

education they left to public health but in reality now they don&#39;t do that. I think it’s very

important to create the consciousness in the people.

[00:27:07]

David: Now they simple come, the fumigator, applies the []. Done. They leave.

[00:27:17]

David: this over here is Charco Azul. This is the Laguna of Charco Azul.

Amy:  wow, [] this is a forest

David: This is the part they have tried to recuperate, this side here.

[00:27:29]

David: This piece here. Because [] contaminated []

Amy:  and here is an invasion

David: yes, that is an invasion of Charco Azul. And there of Los Lagos and over there el [].

[00:27:48]

David: EmCali is Cali&#39;s municipal aqueducts company.  They recuperated this part but look over

there they divided in two parts to recuperate it. This is the part recuperated and from here to there

is the part they haven&#39;t yet recuperated.

[00:28:06]

David: It also has invasion []

Amy:  Recuperated, what does that mean. That its clean or what?

David: The part back there has garbage, the people, everything.

Amy:  The trash, OK. And the other side is clean water to use.

[00:28:30]

David: Yes, No, this water is not. . . This piece is another piece that they have just recuperated.

All of this is one lake but it is a [] as it is bush and water at the same time. In this the company is

working.

[00:28:50]

Amy:  And behind here what is there? More water, more . . .

[00:28:53]

David: Yes, this part here like it is in the photo

[00:28:57]

David: The recuperation . . .

[00:29:06]

Amy:  OK, good.

[00:29:13]

David: that is what the company is doing, trying to recuperate it. []

[00:29:22]

[00:29:38]

David: This part they call Charco Azul

[00:29:47]

David: This part is bad.

[00:29:53]

David: Look at the canal. The canal comes like this traversing all of the city.

[00:30:06]

Amy:  With the lake here it explains everything.

David: Yes,

Amy:  you don&#39;t need more.

David: and over there, is a canal of residual waters [] it comes here to this canal to [] and it

comes to the canal. There is an exit to the lake over here.

[00:30:40]

Amy:  The water is leaving from there to here.

David: And from there going down with the residual waters

[00:30:53]

David: []

[00:31:04]

David: []

[00:31:28]

Amy:  it would be better to take out the canals and put tubes

David: Yes. []

[00:31:38]

Amy:  The tubes []

[00:31:49]

David: over there is Mariana Ramos, Juanchito, Alfonzo Lopez. All of them are over there.

[00:32:08]

[00:32:47]

David: the canals come from the Nueva Floresta

[00:32:52]

David: Over there is the neighborhood [], Alfonzo Lopez.

[00:33:37]

[00:35:12]

David: The is the outside of Charco Azul. The lake is []. The invasion is social strata 1. The

houses are all shacks.

[00:35:30]

David: []

[00:35:40]

David: You can&#39;t enter here at night

Amy:  No, that&#39;s why are working in the morning.

[00:35:55]

[00:36:26]

Amy:  And they are cleaning alone.

David: No, they are form the company [] from the city. They have to clean the street no more.

This is the part they recuperated. You see it looks better, cleaner. But already here the people are

not conscious with the trash.

[00:36:51]

David: and here is a neighborhood that is social strata three that is Villa del Lago.

[00:36:59]

David: Villa del Lago is a neighborhood that is not too [] in the city []

Amy:  It looks more organized.

[00:37:14]

David: Here is the sports center that is from Villa del Lago.

[00:37:21]

David: []

[00:37:42]

David: []

[00:37:59]

David: []

[00:38:36]

David: Do you see?

[00:38:48]

David: They have a key to enter there (the city blocks) because it is dangerous

[00:38:50]

Amy:  it is a community inside then, it is good

David: From here to the there is the Villa de Los Lagos.

[00:38:58]

[00:39:58]

David: her it looks very . . . the boys . . . doing drugs

[00:40:34]

David: This is the center of SENA that that built here for everyone here to learn technical trades

Amy:  of course, it looks great.

David: its new, the inauguration was a little while ago and they only need to fix this. the

installations are new. The people here study []

Amy:  great

[00:41:02]

Amy:  and here behind here what is it?

David: This is what they used for the construction. the lot to guard the things and the bathrooms.

Amy:  they left it there.

[00:41:24]

David: the neighborhood El Pondaje. Dangerous. [] dangerous.

David: Over there is Los Lagos and then El Pondaje and more over there is Charco Azul- where

the police where, to the right.

[00:41:55]

David: The have a culture very normal to smoke drugs in front of the world. They don&#39;t worry

about the people or anything. []

[00:42:09]

David: This is part of Charco Azul where we couldn&#39;t enter over there for the police. This is an

invasion (informal settlement).

[00:42:17]

David: Charco Azul and El Pondaje is what everyone calls it.

Amy:  and here in the invasions, the public services don&#39;t come, right?

[00:42:30]

David: yes, they are together.

Amy:  They have energy and water?

David: They rob it form the state.

Amy:  oh ok, but they have it

David: yes.

[00:42:44]

David: they take it from here. Look, the energy here that is illegal, all of them together over

there. Those are robbing the energy

[00:42:58]

[00:43:06]

[00:43:28]

[00:44:05]

[00:44:14]

Amy:  but the good thing is that they are already working on that, the lake.

David: Yes, [] recuperating it [] the respiratory disease and dengue there.

[00:44:28]

David: [] malaria

[00:44:38]

[00:45:05]

[00:45:41]

[00:45:56]

[00:46:04]

[00:46:17]

Amy:  also we were talking about that of course it is very obvious that if there is a lake that it is a

danger for dengue and malaria but if we aren&#39;t talking about it and looking closely at the relation

and saying something then it won&#39;t change. It is very obvious but we have to do it.

[00:46:49]

David: Yes, of course. [] work on something []. What happens in the city is []. A more beautiful

place []

Amy:

[00:47:24]

[00:47:54]

[00:48:23]

[00:49:10]

David: what you see here a lot are people with drugs and lots of TB that&#39;s why you see a lot of

homelessness

David: []

[00:50:02]

[00:58:18]

Directory:

C:\Users\Amy\Google Drive\Kent\james\dissertation\chkv and dengue\data\video

data\Republica_de_Isreal_Antonio Narino_Calipso_Dec3\Antonio Narino November 26 2015

Audio Files:

ZOOM0001.WAV

Associated video files:

1. FILE0268.MOV

a. Transcript: [00:00:33] David: The neighborhood of Antonio Nariño is a

neighborhood of like social stratus three. It is a neighborhood that is very busy,

many family businesses. And it is very old, it is not new. It is one the first

neighborhoods from Cali.

b. Video: [00:01:26]

2. FILE0016.MOV

a. Transcript: [00:00:33] David: The neighborhood of Antonio Nariño is a

neighborhood of like social stratus three. It is a neighborhood that is very busy,

many family businesses. And it is very old, it is not new. It is one the first

neighborhoods from Cali.

b. Video: [00:01:18]

Neighborhood: Antonio Nariño

Date: November 26, 2015

Interviewer: Amy Krystosik

Driver, vector control specialist: David

[00:00:33]

David: The neighborhood of Antonio Nariño is a neighborhood of like social stratus three. It is a

neighborhood that is very busy, many family businesses. And it is very old, it is not new. It is

one the first neighborhoods from Cali.

David: It has a lot of green areas. We will come to one part in the 48 street that has a water

canal.

[00:01:04]

Amy:  And you think that&#39;s why it has so much dengue.

David: mhm

Amy:  OK, let’s go see because I don&#39;t understand why it has so much dengue. let’s see.

David: Maybe the public health is not coming to do the fumigation service because at least they

used to if there was a case of dengue if they saw there was at least ten or fifteen cases or already

a cluster they will come and send the fumigation to the neighborhood at 6 AM and at 10 PM.

And that&#39;s how they controlled it to undermine the dengue.

Amy:  But not anymore

David: but they are not doing that anymore

[00:01:52]

David: Then this makes that the disease comes back and propagates more because they are not

doing any control.

[00:02:00]

David: the only thing that I see that the Secretary of Health does today is the control of larva in

the breeding sties.

[00:02:10]

David: This is all the 39, it is a canal of rain water.

[00:02:16]

Amy:  Yes, of course, with lots of grass.

David: When it rains in the winter, [] is full of RAS from the floor.

[00:02:24]

David: The water comes up to here to the edge of the street.

[00:02:30]

Amy:  I also saw that it depends on the part of the city if there is flooding when it rains because

in some parts there is flooding in the street and water everyone and in others no. This depends of

what, the level?

[00:03:06]

David: Look this can depend on the maintenance of the drain system because the company

EmCali that is in charge of the aqua duct and the drainage system they, when the winter starts,

the contract people and all the drains and they are taking out all the dirt that that stays there. But

there are places that they don&#39;t reach to do so obviously this fills and as it is clogged, the water

stays out there. This happens in a lot of neighborhoods that they flood for that- that they don&#39;t

maintain the drainage system.

[00:03:43]

[00:04:01]

David: and look here there is another canal of rain water. This one is canal canal.

[00:04:10]

David: I think this is what has more with the dengue incidence. This is black water.

Amy:  from the streets and the houses.

David: from the sewer.

[00:04:21]

Amy:  there are people that live here too, no

David: Yes, around this. There are a lot of homeless people.

[00:04:37]

[00:04:44]

[00:05:09]

[00:06:39]

[00:07:25]

[00:08:12]

Amy:  they are working a lot in the neighborhood in the streets. the streets are new.

David: they are new.

Amy:  what I see is that they are working here.

[00:08:54]

David: fixing it up. Maybe the infrastructure improves and diminishes because before this before

this was with holes and puddles and in the puddles is where they reproduce.

[00:09:03]

David: Look they have fixed the streets. All of this is new.

[00:09:08]

David: the north to south streets like this, it is the streets east to west that they haven&#39;t fixed.

[00:09:19]

[00:09:32]

[00:10:19]

[00:11:30]

[00:11:40]

[00:11:57]

[00:12:10]

[00:12:34]

[00:12:57]

[00:13:17]

[00:14:52]

[00:15:36]

[00:15:50]

[00:16:21]

[00:17:21]

[00:17:26]

[00:17:49]

David: and look here in the middle of Antonio Nariño is another canal that goes to the other

canal, look.

[00:17:53]

[00:17:57]

David: all of this is Antonio Nariño and there are parts, look at least here, all of this is covered

[00:18:05]

[00:18:12]

[00:19:59]

[00:20:38]

[00:21:54]

[00:23:30]

David: all of this is Antonio Nariño and look it has the canal there.

[00:23:39]

David: I think the thing that affects it most is the canal of residual water.

[00:24:17]

Amy:  yes, but the canal is in all of the city and it only affects some neighborhoods.

[00:24:24]

David: it should be that is more still in some parts because in other parts it can be that it more

fluid that is moves more whereas in other parts it is stagnant.

Amy:  Or for example the trash that it has there, the cans and all of that as a breeding site.

[00:24:44]

David: At least there look all the trash that is there.

[00:24:45]

[00:24:55]

[00:25:11]

[00:25:21]

[00:29:43]

David: over there are only businesses

[00:29:47]

[00:31:04]

David: and here look

Amy:  the church of San Mateo and this is a school but very old

David: they are remodeling it

[00:31:20]

Amy:  this is the sports center of the Poblado

[00:31:45]

[00:34:06]

[00:35:18]

David: These are Christmas decorations but they are hanging trash.

Amy:  but beautiful they are decorating and cleaning

David: It’s that here the tradition is to clean before the candles day which is supposedly the 7th

of December so everyone is cleaning the streets and this day the go out and put the candles

outside the houses in the gardens.

[00:35:59]

[00:40:36]

[00:42:13]

[00:43:46]

David: But look this piece of Antonio Nariño is very ugly, horrible.

[00:43:49]

[00:44:13]

[00:44:35]

David: but you can&#39;t go into those alleys because they are very narrow

[00:44:41]

[00:45:01]

[00:45:46]

[00:48:32]

[00:50:29]

David: look the police are scaring away all those boys that were there using drugs

[00:50:43]

[00:52:04]

[00:53:09]

[00:53:46]

Amy:  but it’s very strange that they have a mix of beautiful houses with tin houses and no they

are developing very bit by bit.

[00:54:14]

[00:54:44]

David: this here at night you can&#39;t enter. the people come out with arms.

[00:54:52]

David: they [] all the corners with arms and everything.

[00:55:01]

[00:55:44]

[00:55:52]

[00:56:05]

David: This egg car has a shooting guard guarding them so they don&#39;t rob them. They go in

motorcycles guarding the sales trucks. How would it be to live here.

[00:56:25]

[00:58:12]

David: Look at the canal. The 39. Look there is a man going into the canal. Look at him there.

Amy:  Lots of trash. Is it raining?

David: No, I think it’s the larva that make the water move like that.

[00:58:34]

David: Larva, tadpoles, who knows how much, look at the other side, it looks terrible.

[00:58:44]

Amy:  They use it as a trash pit.

David: the people don&#39;t have consciousness for . . .

[00:59:04]

[01:00:40]

[01:02:01]

David: this was a house, the abandoned it, the knocked it down, and now it’s just a lot.

[01:02:10]

[01:03:12]

Amy:  More turkeys, the have their own farm here.

David: a farm next to the canal. Those are all plantain trees and those are papaya trees. Look at

the papaya trees. The dirt here is more fertile. One drops any seed anywhere and it grows.

[01:03:47]

Amy:  that horse is sick on the floor.

[01:03:55]

David: they have some old ones look.

Amy:  I didn&#39;t know that, that this existed here in Cali.

David: look at the vultures there in a meeting.

Amy:  oh no, there is a dog there.

David: they buried him for sure. Yes, they are eating him.

Amy:  So ugly those birds.

David: those birds eat whatever

Amy:  but OK they clean up.

David: yes, they make an ecological control.

[01:04:52]

[01:05:34]

[01:05:56]

[01:06:22]

[01:06:39]

David: this is the one that we see before that I told you was a school.

[01:06:53]

[01:07:34]

[01:08:42]

[01:09:15]

[01:09:48]

[01:10:38]

[01:12:07]

[01:14:03]

[01:14:19]

David: Look all of this has the canal of rain water and the canal on the other side

Amy:  both

[01:14:27]

[01:16:11]

David: taking out water.

Amy:  Then the water services don&#39;t come to here

David: yes, this is that maybe, this is called the well, they are taking out

[01:16:37]

[01:18:18]

[01:19:17]

[01:19:56]

David: Republica de Israel. It is small.

Amy:  It is small, but it has a lot of cases

[01:20:01]

Amy:  The neighborhood has a lot of cases although it is small.

David: Yes.

[01:20:17]

[01:20:54]

David: Antonio Nariño is more dangerous than Republica de Israel. Republica is an old

neighborhood of the city and everything but it’s not so dangerous. Antonio Nariño is social

stratus one and two. Here it passes up to social strata three.

[01:21:38]

Amy:  then it should be that it is located next to the canal and Antonio Nariño that it had so many

cases.

[01:21:59]

[01:22:02]

[01:23:30]

[01:24:33]

[01:25:22]

[01:25:53]

[01:26:02]

Directory:

C:\Users\Amy\Google Drive\Kent\james\dissertation\chkv and dengue\data\video

data\Republica_de_Isreal_Antonio Narino_Calipso_Dec3\Calipso November 26 2015

Audio Files:

ZOOM0003.WAV

Associated video files:

1. FILE0269.MOV

a. Transcript: [00:00:45] David: Calipso was a bigger neighborhood however with

the construction of this they cut it. They bought houses and everything because

this a long time ago it was only this highway for the two lanes and imagine how is

the other highway now. They had to buy all of this that was houses so they could

build. On that side there was a control of buses where all the buses came and the

guarded them there.

b. Video: [00:00:41]

2. FILE0018.MOV

a. Transcript: [00:00:45] David: Calipso was a bigger neighborhood however with

the construction of this they cut it. They bought houses and everything because

this a long time ago it was only this highway for the two lanes and imagine how is

the other highway now. They had to buy all of this that was houses so they could

build. On that side there was a control of buses where all the buses came and the

guarded them there.

b. Video: [00:00:39]

Neighborhood: Calipso

Date: November 26, 2015

Interviewer: Amy Krystosik

Driver, vector control specialist: David

[00:00:22]

[00:00:45]

David: Calipso was a bigger neighborhood however with the construction of this they cut it.

They bought houses and everything because this a long time ago it was only this highway for the

two lanes and imagine how is the other highway now. They had to buy all of this that was houses

so they could build. On that side there was a control of buses where all the buses came and the

guarded them there.

[00:01:15]

Amy:  but I imagine with this bus station in front, it has improved a lot.

[00:01:29]

David: yes, it has improved a lot. The danger was [] crazy

[00:01:32]

Amy:  before

David: now it less dangerous. Before it was more dangerous.

[00:01:45]

[00:01:54]

David: they are pedestrian streets, like I told you, so we can only do the border of the

neighborhood because we can&#39;t go in.

[00:02:19]

[00:02:47]

Amy:  it is well organized.

[00:03:07]

[00:04:12]

[00:05:19]

[00:05:54]

David: look at the family recycling there.

Amy:  and very organized.

David: but what a shame the children they shouldn&#39;t put them there. The children should be

resting or doing something else.

Amy: Ah OK because they are on vacations. But if they are in school and they want to work on

vacations, ok.

[00:06:26]

[00:06:44]

Amy:  There was a sign that said &quot;Neighbor and recyclers, no throw the trash there. Caution.&quot;

David: Fines it says there. But this puts just the person. It’s not that the government is going to

fine them there so they don&#39;t throw.

[00:07:31]

[00:07:52]

[00:08:41]

Amy:  and was does Calipso have to have dengue?

David: Look, the only thing that I see is that there is a canal over there. It’s the only thing.

[00:08:49]

Amy:  Then we pass for that again

[00:08:55]

David: If no, the canal, supposedly, following the map, the canal is not in Calipso. It is there in

the trunk. That&#39;s what I think. As the mosquito is migratory and has a range of action.

[00:09:19]

David: How many [] can a mosquito fly?

Amy:  with wind, up to 5 kilometers.

David: imagine

Amy:  Yes, a lot

David: and the canal is just there, 100 meters away no more

[00:09:32]

David: you want to see where it is, the canal

Amy:  Yes

[00:09:38]

[00:10:15]

[00:10:27]

[00:11:13]

[00:11:54]

David: do you see why there is dengue in Calipso, look. The canal of

Amy: []

David: look at the trash.

[00:12:14]

David: look, do you see. This is maintained full. Not empty

Amy:  And it is not moving at all. It is stopped.

David: do you see. I think all the movement that you see is lava and who knows tadpoles what

else.

Amy:  can we get down and see?

David: of course.

Amy:  ok, let&#39;s get down.

[00:12:54]

David: do you see. The way is here and stagnant.

[00:13:09]

[00:14:19]

[00:15:13]

David: Now you see, Amy, why Calipso can have dengue.

[00:15:16]

Amy:  Yes.

David: I, I, that is, I assume the disease is more in these parts.

[00:15:28]

[00:15:38]

[00:16:01]

David: [] the sewage here.

[00:16:11]

David: When the sewage [] that way it also [] the mosquitoes. It is a foci of reproduction.

Amy:  for what?

David: for the mosquito. They are changing, that is, for all of this block they are changing the

sewage. That is the drain from the house.

[00:16:33]

Amy:  the condition of the street is good.

[00:16:40]

Amy:  also we have to look inside the house to see what they are doing. This

David: Also, right

Amy:  but that is another big project.

[00:17:03]

[00:18:42]

[00:20:01]

Amy:  these tanks, if they aren&#39;t moving constantly

David: that can also be. There they rent those to mix the cement.

Amy:  OK, those sitting there

[00:20:31]

[00:20:52]

David: and this that has this lot all [], look.

[00:20:56]

[00:21:22]

[00:21:54]

[00:22:05]

Amy:  and this lot that is here.

David: Yes, look, who knows what they have there or what- throw. It could be from the

municipality. All the lots from the municipality are like that.

[00:22:39]

[00:22:46]

David: this is what, a residential community.

[00:23:10]

[00:23:20]

David: We go around the lot; you want to see the lot so we go around?

Amy:  Yes

[00:23:41]

David: OK. But this part is like the ugly part of Calipso. Everything is covered. Did you see over

there everything was beautiful and organized? Look here.

[00:24:09]

We will go around the lot.

[00:24:45]

[00:25:28]

[00:25:49]

[00:25:39]

Amy:  a lot for kites, the lot eat kites

David: The people begin to fill it like that with trash, trash and it turns into a trash dump.

[00:25:57]

Amy:  Yes, but it looks like it was something before, that is had a building or something.

[00:26:14]

[00:27:00]

David: lots like this, look.

[00:27:05]

[00:27:39]

[00:27:54]

[00:28:04]

[00:28:41]

[00:29:05]

[00:30:04]

[00:30:16]

[00:30:50]

David: look these houses they have done here in between the lots- an invasion. They have

invaded the lot.

[00:31:03]

[00:31:30]

[00:31:59]

Amy:  then this is an invasion of Calipso

David: Yes, that lot, that shack is an invasion.

[00:32:27]

David: done, turn off now

Amy:  Yes.

Directory:

C:\Users\Amy\Google Drive\Kent\james\dissertation\chkv and dengue\data\video

data\Republica_de_Isreal_Antonio Narino_Calipso_Dec3\Nueva Floresta November 26 2015

Audio Files:

ZOOM0002.WAV

Associated video files:

1. FILE0268-1.MOV

a. Transcript: [00:01:08] David: look, this neighborhood is already a social stratum

three. The houses are more beautiful.

b. Video: [00:01:10]

2. FILE0017.MOV

a. Transcript: [00:01:08] David: look, this neighborhood is already a social stratum

three. The houses are more beautiful.

b. Video: [00:01:09]

Neighborhood: Nueva Floresta

Date: November 26, 2015

Interviewer: Amy Krystosik

Driver, vector control specialist: David

[00:00:08]

[00:00:43]

[00:00:50]

[00:01:08]

David: look, this neighborhood is already a social stratum three. The houses are more beautiful.

Amy:  Yes, they are more beautiful.

[00:01:14]

Amy:  and more organized

David: and more fixed up. The side over there was very ugly.

Amy:  although yes it was some yes and some no

[00:01:27]

[00:01:44]

David: this Carlos Carmona the hospital, I think here is where they report more cases of dengue.

[00:01:52]

Amy:  I imagine

[00:01:59]

[00:03:06]

[00:03:35]

[00:03:54]

[00:05:20]

[00:06:10]

[00:06:20]

David: But the good thing about this neighborhood is that at least the streets are good and there

are not too many alleys.

[00:06:33]

[00:07:01]

[00:07:07]

[00:09:43]

[00:09:52]

[00:10:38]

David: here you have the canal of rain water but it has become like a canal [of black water]. The

people have thrown the trash and everything there.

[00:10:48]

David: There are people who don&#39;t have the consciousness and who are very dirty. Instead of

paying or take out the trash when the car comes, yes or no. That they should take the garbage to

the car, not it comes and trumph they throw it there.

[00:11:00]

Amy:  but it’s not just a person, it’s the community

David: yes, but it’s for the lack of culture, the environmental culture.

Amy:  difficult.

[00:11:13]

Amy:  but Cali has everything, people who are very conscious and people who are not.

David: yes.

[00:11:34]

[00:11:55]

[00:12:39]

[00:13:09]

[00:14:09]

[00:14:37]

[00:15:02]

[00:17:03]

Amy: Juan Jacobo High School.

[00:17:14]

[00:17:43]

[00:19:35]

[00:20:15]

[00:20:26]

[00:20:46]

[00:21:10]

[00:21:48]

[00:22:05]

[00:21:58]

David: This one we could cover well because there are more vehicular streets but in Antonio

Nariño there were lots of dead ends and pedestrian streets. We arrived to Antonio Nariño early

and it is good to enter early to these dangerous neighborhoods because the later it gets the

more dangerous it gets. At this time in Antonio Nariño there are entering more gangsters,

homeless people, thieves.

Amy:  Yes, more difficult.

[00:22:21]

[00:22:39]

[00:22:55]

David: And this neighborhood to have cases of dengue like this- look the streets are not so ugly

or anything. The only thing it has is the canal over there and the zones with lots trees on each

side.

[00:23:11]

David: a lot like this in this neighborhood. Remember there was another one like this when we

were starting. Look, here is another one.

[00:23:36]

David: this maybe the owner died and didn&#39;t have anyone to leave it to so he left it there alone.

[00:23:44]

[00:24:46]

[00:24:51]

[00:25:46]

David: Ready, Amy, we have covered all of Republic of Israel.

Amy:  Ok, then let&#39;s go to Calipso but let&#39;s turn this off so it doesn&#39;t sound again.

[00:26:17]

Directory:

C:\Users\Amy\Google Drive\Kent\james\dissertation\chkv and dengue\data\video data\siloe

october 14

Audio Files:

siloeOCT20.wma

Associated video files:

siloe october 20 (209).MOV

siloe october 20 (212).MOV

siloe october 20 (213).MOV

siloe october 20 (214).MOV

Neighborhood: Siloe

Date: October 20, 2015

Interviewer: Amy Krystosik

Local Community member:

Driver: Santiago

[incomprehensible with camera audio]

Directory:

C:\Users\Amy\Google Drive\Kent\james\dissertation\chkv and dengue\data\video data\Terron Colorado November 2, 2015

Audio Files:

Terron Colorado November 2, 2015 Marco Lopez.MP3

Associated video files:

1.      Terron Colorado nov 20 2015 (216b).MOV

a.       transcript [00:34:50] Marco: we are going up to Patio Bonito.

b.      video [00:00:26]

2.      Terron Colorado nov 20 2105 (219).MOV

a.       transcript: [01:20:50] Marco: look at the dogs up there.

b.      video [00:01:26]

Neighborhood: Terron Colorado

Date: November 2, 2015

Interviewer: Amy Krystosik

Local community member: Marco

Local: Alvaro

Driver: Elmer

[00:00:18]

Alvaro: where are we

Marco: we are here in Terron Colorado on the street 4 east with 19

[00:00:42]

Marco: we are in the center of health of Terron Colorado

[00:00:53]

[00:01:04]

Marco: and as you can see, look

Alvaro:  there are lots of things outside, trash.

Marco: Yes.

Marco: at least look at the street, it is not too good.

[00:01:22]

Amy:  is it raining a lot here in these days?

Marco: in these days, yes. They are light rains but yes it is raining at least in the night and the other night too.

[00:01:38]

Driver:  these days [] the winter. [] the contamination of the mosquito.

[00:01:44]

Marco: there is a [] more than anything the mosquito. When it rains, it makes the puddles.

[00:01:51]

Driver:  and as the fumigation they do every six months or once a year

Marco: no, here- once a year they do a fumigation.

[00:02:01]

Driver:  This they always []. What they do is []. They do it in parts like this.

[00:02:13]

Marco: here we are entering the part with the apartments. The streets are in poor condition.

[00:02:20]

Marco: very bad. Wherever you look there are puddles. Where the mosquito produces a lot.

[00:02:35]

Marco: this is the "tablazo" part of Terron Colorado. "Tablazo" because it is very hilly. Very steep.

[00:02:38]

[00:02:43]

[00:02:58]

[00:03:23]

Marco: OK, look here the puddles in the road where the dengue mosquito propagates a lot.

[00:03:32]

[00:03:42]

Alvaro:  a lot of dogs.

Marco: yes, lots of dogs.

Driver:  yes, this is another problem because the people have their pets and they let it go. This produces a lot of infection. There are a lot of dogs leaving

Marco: his necessities in the street. This is a problem too.

[00:04:05]

Driver:  and it attracts a lot of flies.

Marco: a lot of flies a lot of mosquitoes.

[00:04:17]

Marco: look, as you can see, the streets are in bad condition

[00:04:21]

Marco: every time it rains it's forming puddles of water.

[00:04:29]

[00:04:42]

[00:04:58]

Driver:  down there is the part we should see.

Marco: That is the part most contaminated down there but too bad we can't go because in the part there are borders.

[00:05:12]

Marco: That is, it is a red zone and they make borders everywhere you look

Driver:  and the only police you see are sleeping [speed bumps]

[00:05:24]

Marco: they are sleeping there; they are the police who enter here. Here the form of that can kind of enter here are the soldiers. From here to there, no.

[00:05:39]

Amy:  The streets and buildings are the same?

[00:05:41]

Marco: yes, one part. In another part, they are in mud and straw.

[00:05:48]

Marco: here you can enter. From here to there you can't enter. It’s not recommended.

Alvaro:  but till now they are sleeping.

Marco: yes, they are sleeping.

Alvaro:  that was the idea of coming now- they had finished drinking and . . .

[00:06:04]

Marco: until now they are sleeping but around eight or nine at night. . .

[00:06:08]

Driver:  a companion taxi driver came to the station of the firefighters to leave two women who came from these houses. and [] they robbed him in motorcycles.

[00:06:41]

Alvaro: []

Marco:

[00:06:52]

[00:07:10]

Marco: this is the sixth street. This street they half-fixed because this was all dirt.

Alvaro:  Unpaved

Marco: yes, unpaved

[00:07:23]

Marco: half-way they fixed it

Amy:  when did they fix it?

[00:07:26]

Marco: It has been seven months, no? Something like that. But it, look, it already [] again. Look, there are too many dogs too in the streets and in the houses. Dogs, cats.

[00:07:45]

[00:08:19]

[00:08:22]

Marco: This is the street that one can go down more relaxed. My aunt lives here in this corner. She lives on the second floor closed in there. []. All of this is hers.

Alvaro:  how long have you lived here?

Marco: I was raised here since I was five years old.

[00:08:48]

Marco: forty years.

[00:08:59]

Driver:  my grandma brought me here in 1962, to Cali.

Marco: all the family of my mother lives here and my father's lives here too. Most of them.

[00:09:15]

Alvaro:  how often does the trash truck come here?

Marco: two times a week.

Alvaro:  and they come on time?

[00:09:22]

Marco: yes, when they don't stop.

[00:09:23]

Marco: when they don't stop it because when they stop it the trash stays there. This is another problem because when the cars don't collect the trash the people take out the trash and leave it at the corner. And the dogs begin to come back and leave the streets full of trash. The environment. Everyone knows that this smells very bad. And also, this is what attracts so many flies and mosquitoes.

[00:09:54]

Marco: this happens here Mondays- Mondays and Thursday or Friday

Driver:  Thursday.

Marco: Mondays and Thursdays no more.

[00:10:09]

Marco: Good, now, at least, they have taken all the trash from the corner.

[00:10:56]

[00:12:30]

Marco: This is another part down there that one cannot enter, La Planeta. Look how it is so ugly. And look at the sewer and everything. This is called La Planeta. But no, neither is recommended to enter there. Up to there in the first houses you can go but from come back. That is a zone of borders. [They would ask] "What are you doing here, where are you going?" OK, the start to investigate you and they put you in [].

[00:13:07]

Amy: []

Marco: this is the famous trapichito of Terron Colorado very well-known as they Trapichito.

[00:13:25]

Driver:  this street is so bad...

Marco: yes, some very tall speed bumps

[00:13:27]

Marco: Do you know why? The motorcycles. Because the thieves on motorcycles here- this is the only way the police have to slow them down. But anyways they all do wheelies over the speed bumps and fly.

[00:13:55]

[00:14:28]

Marco: This is a part where you can see a part of that below of La Planeta.

[00:14:45]

Marco: it's as you see. You can see- look how are the streets. Look down there is [] up to there, no.

[00:15:07]

Marco: Look, La Planeta, this is steep. But below are the stairs and pedestrian streets

Driver:  all pedestrian streets

Marco: pedestrian, yes.

[00:15:33]

Marco: and below is the river Aguacatal. I imagine that all of this water goes to the river too and . . .

Driver:  That is the river gets contaminated.

[00:15:50]

Marco: the river gets contaminated and there too the mosquito reproduces. Or the fly.

[00:16:07]

[00:16:34]

Marco: this looks like this, fixed up, because this they fixed a year ago. Because this was terrible.

[00:16:52]

Marco: there is a part- a part that is this part here- this side is the famous []. and here on the right too. All of this is water from the tubes. When they break, they come here.

Alvaro:  to the canals.

Marco: to the canals. And this contaminates all Terron Colorado- from here to the bottom.

[00:17:23]

Marco: From up there coming down- when those tubes break []. All of this is Terron Colorado but they all call it Vista Hermosa. What is the other neighborhood? Patio Bonito.

[00:17:47]

Marco: over there too it is hot.

[00:17:48]

Marco: Look, most of the houses, all of this, all of this consists of La Planeta. All of that over there. They are houses- All of this is invasion. Then what happens is there are people from one side and another side- people from one side and another have come here and this is what causes damages. [].

[00:18:14]

Marco: and they go and they do invasion and they don't make good piping and they make latrines and ok that. Exists a form of [].

[00:18:24]

Marco: here we can enter up to a point. This is also aware but we can enter up to a certain point too.

[00:18:46]

Marco: This is known as Villa de Martha. It's part of the head of Terron Colorado.

[00:18:52]

Marco: look at all of this here. Workshops and . . .

[00:19:05]

Marco: As you see, a house that isn't finished yet.

[00:19:23]

Marco: this is all invasion. All of this is invasion.

[00:19:25]

Marco: look here, look Via Lengua- how it extends

[00:19:36]

Marco: that is masamora they are selling there. The people buy it for 500 pesos each ladle. He sold him 2 thousand pesos.

[00:20:17]

Marco: those are pots of masamora- cooked corn.

[00:20:22]

Marco: it's hot. They put milk from a black cow in there and it's ready.

[00:20:35]

[00:21:15]

Marco: look, the further down you go, you can see more . . .

[00:21:23]

Marco: we will turn around later. or here because they are already waking up. really hot.

[00:21:39]

Marco: the good thing here is that you come down in a car and they think you are looking for an address or something, but...

[00:21:44]

Marco: this is the low part of the neighborhood Cabeze de Terron Colorado

[00:22:08]

Marco: This is the famous Villa del Mar down here

[00:22:16]

Marco: One can up to a certain point one go because the thieves are sleeping but later when they wake up . . .

Marco: this is vista del mar but it's part of Terron Colorado. They just put different names to the high part

[00:22:52]

Marco: look, here you see via Lengua. You say it as safe but if one enters . . . One cannot enter not even the police over there.

[00:23:10]

Alvaro:  what is the work of the people here.

Marco: most are recyclers. For the 16 they are recyclers. They are

Alvaro:  ladrones

Marco: yes. and the people hide there- in the bottom part in Via Lengue, from- people of drugs. There they sell a lot of drugs too. And the people who like the bad [manas].

[00:23:43]

Marco: well that is to say [tratadores] and all of those people.

[00:23:52]

Marco: look, maybe if you on a motorcycle enter there- you lose the year.

[00:24:04]

Marco: Yes, we will enter here in the high part of Villa del mar. It stays the same. All of the streets in Terron Colorado and the same in high parts, the streets are bad.

[00:24:16]

[00:24:27]

Marco: lots of bad streets, too many animals in the streets.

[00:24:39]

Marco: all of the streets go there and one goes back because from to there is hill. they are stairs.

[00:24:51]

Alvaro:  and here come the services or what do they do

Marco: yes, there are services because the water that comes from the river of Santa Rita or the Cali River comes to the [] and from there they pump it.

[00:25:15]

Marco: this is part of Villa del Mar.

[00:25:23]

Marco: Luckily came this driver because there are others who won't go inside here. They tell you "for here I don't go. I will leave you here."

[00:25:47]

Most of them are in pregnancy. Like this one, this one. Most of them are in pregnancy, look.

[00:25:53]

Marco: This was a paddock here. All paddock.

[00:26:03]

Marco: there’s a part there that to go in, in car you can't, it's all stairs.

Driver:  most of Terron Colorado is stairs.

[00:26:13]

Marco: It is stairs

Driver:  now with the new mayor they are going to put in escalators

[00:26:26]

[00:26:34]

Driver:  oh no, should we go up there?

Marco: no, I don't think so

Driver:  they are standing there

[00:26:44]

Marco: most of this so you see, look, look

Alvaro:  very ugly this piece here.

[00:26:50]

Marco: look, here no

Driver:  go back.

Marco: go to then end so we can go out the other end.

Driver:  it's very ugly

Marco: it's very ugly this piece

[00:27:04]

Driver:  all of this is Terron Colorado

Marco: all of this is the high part of Terron Colorado

[00:27:16]

Marco: Can it be that one can enter over there? Here they were going to invade but they didn't let them.

[00:27:22]

Driver:  here no more because

Marco: yes, over there they are already awake. Right there they ask you where are you going and the take one.

[00:27:35]

Marco: the hot parts better no. Leave them safe and alone.

[00:27:47]

Marco: as this is invasion. The people that invade are not good people.

[00:28:03]

Marco: the bad thing is that they know the cars and the people but when a different car enters- it's like "where are you going?" I think that to go in there it has to be someone who lives inside there.

[00:28:20]

Marco: They just see who enters with one and see who goes out and everything there

Alvaro:  equal, they are alert.

[00:28:35]

Marco: one can go inside up to a certain point. But nevertheless they look at one

Alvaro:  at once they all turned around.

Marco: they all turned around

[00:28:44]

Marco: that this is the first time. We are going and looking for a direction. ah ok yes.

[00:28:52]

Marco: yes, more than anything here. look at the deteriorated streets

[00:28:59]

Marco: a lot of []

[00:29:04]

Marco: look at the water as it falls it forms puddles

[00:29:12]

Marco: they form puddles and lots of humidity

[00:29:19]

Driver:  Really, what is missing here is the fumigation.

Marco: the fumigation

Driver:  The fumigation- if they at least did it every month or every two months

[00:29:34]

Marco: If they did it every two months, it wouldn't propagate much

Driver:  It wouldn't resist, the disease- not dengue, nor chikungunya wouldn't resist any of that.

Marco: we can up there.

Driver:  yes. I see the worst puddle for the mosquito is that they don't fumigate.

[00:29:54]

Driver:  fumigate every time

Marco: yes.

[00:30:11]

Marco: this is Cabazero of Terron Colorado, all of this is Terron Colorado

[00:30:40]

Marco: we are going to go down to- to what part? That is Patio Bonito.

[00:30:49]

Alvaro:  and that’s what they call Terron Coloradiado

Marco: up there. from here up see the houses that are colored. and this they did with someone who entered in there. Someone from TV. What’s he called? He came in and entered and united the people and between everyone they painted because no one can enter there.

[00:31:19]

Marco: He united all the people and all the people helped and everything that they gifted it the pain and they painted and for that reason they call it Terron Coloradiado.

[00:31:28]

Marco: it was an actor who entered here. The gave him a part and he entered and he didn't know and he had to meet with all the people and there the recorded the people but the cameras they brought. and the people from there helped. All of this is Patio Bonito- part of Terron Colorado. But this is also Terron Colorado

Driver:  we go in a bit there?

[00:31:58]

Marco: Yes, let's go in up there a little bit. Not up to very inside up there. Just up to a certain point and we come back.

[00:32:06]

Marco: ok, this is part of the cabeza of Terron Colorado which is very well known as Terron Coloradiado.

[00:32:30]

Marco: we are going to go up to a certain point up to where we can because the thieves are sleeping still and they haven't gotten up.

[00:32:54]

Marco: look, the streets up here look better than those down there. They look much better.

Amy: yes

[00:33:01]

[00:34:01]

Marco: the actor filmed everything going up here.

[00:34:02]

Marco: look, her you see via lengua. The bottom part of where we entered just now where the was dirt in the point where we arrived to the church.

[00:34:19]

Marco: This neighborhood is called vista Hermosa but it is part of Terron Colorado. The head of Terron Colorado.

Driver:  It has another, no?

Marco: Patio Bonito, but higher up it is Patio Bonito.

[00:34:44]

Marco: look, it's going to rain or what?

[00:34:50]

Marco: we are going up to Patio Bonito.

[00:35:11]

[00:35:28]

Marco: higher up can do a turn. He is there. We can up to there no more, where the motorcycle is up there.

[00:35:40]

Marco: Part of Terron Colorado very well known as Patio Bonito.

[00:35:55]

[00:36:57]

Marco: we didn’t' go into Patio Bonito in the point we reached. We went half way more or less. Here we come back again.

[00:37:07]

[00:38:05]

Marco: as you can see most are attentive. The go up and down the hill.

[00:38:11]

Marco: for here no one goes up except the jeeps.

[00:38:19]

Marco: for here you almost don't see taxis. Where do you see taxis? No you don't see them. Now you know why. The come to the principal road no more. Up to this hour, obviously as they are hungover and sleeping.

[00:38:50]

Marco: I have mine []. That way I have mine.

Driver:  hot, throw at you . . .

Marco: they are, the call them [libres]. Some [libres]

[00:38:59]

Marco: look, do you see. they go in circles. They are circling.

Alvaro:  what do we do?

Marco: no, they already left.

[00:39:39]

Marco: That here you can't come back but you already went up and down. The come up and down.

[00:39:51]

Marco: look.

[00:39:55]

Marco: they like to fight, the leaders here.

[00:39:59]

Driver:  they are the ones who sell the drugs here.

Marco: yes, they are those in charge drugs.

[00:40:10]

Driver:  That is one cannot go higher up there.

Alvaro:  there's no way to come back.

Marco: no, there is a way to come back. What happens is they tell you "OK, you are coming to sell here. It’s that they sell, or that is they think that it is territory of theirs. Someone else is going to come to their territory and they get riled up "What's going on?"

[00:40:31]

Driver:  look at him, when he is doing. Maybe he comes [] the law.

Marco: yes.

[00:40:37]

Marco: The law but theirs. They give fight and call "look".

Driver: []

[00:40:58]

Driver:  that is the problem is, all the neighborhoods outside of Cali, all of them, most of them, are dangerous.

[00:41:11]

Driver:  up to the part on the bottom, all of them. They are all invasions.

Marco: the problem of invasions. Look here. The problems that occur in the high part accumulate in the low part of Terron Colorado and this is what more brings, more brings the mosquito. The humidity. The streets broken. There they make the puddles and OK begins the whole thing with the mosquito.

[00:41:40]

Marco: mosquito, mosquito, flies also. For what I told you that the people leave the trash in the streets and the animals, the street dogs, do it and do it and leave all of the trash in the street. And the animal feces bring lots of animals like the flies and mosquitoes.

[00:42:05]

Driver:  here the idea would be to move you in all parts. To investigate well but no.

[00:42:13]

Marco: one can enter up to a certain point no more. Here we can enter for the part here. Here the is no problem. Although the police stay here but here no. They don't go over there. They come here and don't pass. Over there someone attacks you, while you call them, no, because there they take them. At once they take them and at once they harm there or what. They can't go up higher either. The only ones who can up there are the soldiers. []. But the police come to this point no more.

[00:42:57]

Marco: look at the problem of the trash. Where they leave it. Do you see?

[00:43:03]

[00:43:23]

Marco: look, most are recyclers. Today you don't see it but the amount of trash, because maybe the car already took a part.

[00:43:44]

Marco: this the bottom part of the Cabezero de Terron Colorado

[00:43:56]

Marco: here a bit it has improved although it was all like this.

[00:44:03]

[00:44:27]

Driver:  at now as the rain has started, I don't know if you see what I have, the flu.

Marco: the change in the weather.

Driver:  but I don't know if it's the weather or it's the amount of contamination that there is. The tremendous contamination that there is.

[00:44:48]

Marco: here it is more- here one can go even on foot.

[00:45:08]

Marco: This here is the bottom part of the church up there.

[00:45:17]

Marco: but the high part is the Cabezero de Terron Colorado

[00:45:20]

Marco: it's that all of this is Terron Colorado. all of this.

Marco: Yes, up there was Patio Bonita that we saw before although we didn't see all of it because you can only go in so far, no more. this is Vista Hermosa where we are going now. Over there is Villa del Mar, where we went with the mud. Villa del Mar.

[00:45:49]

Marco: up to a certain point no more.

[00:45:56]

Marco: but at least over there where they were following us above without fighting, they were looking and everything.

[00:46:08]

Marco: But we have already left there and they don't come down here. Because they can't come down here because those from here ask "OK, what are you doing here? Your territory is there."

[00:46:26]

Marco: they are the famous, how do you call them, the what, the invisible borders. Invisible borders because you don't know. You go for there and from here to there and you can't go from here to there nor there to there. But as you don't know, one doesn't know. . .

Marco: we take the other side of this because we will come up by the church. To go down the other were we entered. We are going good. We go up one and down the other. Look, lots of animals in the streets.

[00:47:20]

Marco: here you exit to the church. it exits to the principal. But what happens is [] where we exited.

[00:47:43]

[00:48:16]

Marco: look, lots of animals in each house. Each house has two or three animals.

[00:48:25]

Marco: abandoned houses. Lots of abandoned houses. Lots of trash in the streets. Look. [] lots of trash in the street. Because they take out the trash and they hope that the car will . . . The car cannot pass and manage the trash there. It's only [] the animals. Dogs and cats of the streets.

[00:49:03]

[00:50:50]

Marco: in all the parts where one goes, where one enters, trash. trash and trash for both sides. look. [] trash [].

[00:51:03]

Marco: look at the trash where they leave it. Look at the trash. Look.

[00:51:13]

[00:51:42]

Marco: the people here- they are recyclers and the same ones go around with two, three, four dogs.

[00:51:53]

Driver:  the problem with loose dogs is the feces

Marco: yes

Driver:  this attracts lots of flies.

Marco: look at the trash. Wherever you look there is trash. In every corner. trash, trash, trash. Because the trash here in the high part of Terron Colorado and the low part of Terron Colorado, Vista Hermosa of Terron Colorado, it passes Mondays and Thursdays. When the car goes, all the trash stays in the street.

[00:52:26]

[00:53:54]

Marco: yes, we are going down from the Cabezera of Terron Colorado. We are going again to the lower part of Terron Colorado.

Driver:  as you can see there is a lot of contamination of cut []

Marco: as you can see, the good thing here, today there is no, today there is no water. I don't know what happened. Look. I don't know what happened but here there is a lot of sewage. Contaminated water from the drains that are plugged. This maintains a river going down here.

[00:54:23]

Marco: this damages the street as well as well proliferating a lot of mosquitoes. Like this look, the stagnant water. Lots of stagnant water. Lots of sewer water.

[00:54:39]

Marco: lots of trash too on both sides. The same people take out their trash instead of the day when the car comes, no, they take it out one day before. and they leave it there all the night, all the day.

[00:55:02]

Marco: look at least at this case. Look at how the people take out the animals. They do there their necessities and they leave everything there. They don't pick it up. They don't pick it up rather they leave the necessities there.

[00:55:52]

[00:55:58]

Marco: but this part here in the high part of Terron Colorado is where you find the sewers that they maintain constantly broken. They are blocked, broken for the same people who throw the trash in the drains and it blocks the drains and the water goes out on all the street.

[00:56:20]

Marco: today no, today however look hasn't been, the []

[00:56:26]

Driver:  in the winter, this side over there in the winter huh, it gets full of ...

Marco: yes, it overflows and the contaminated water takes. . .

Driver:  there are some houses that had flooded here in this part.

[00:56:46]

Marco: this part down here, one doesn't go for the stairs.

[00:56:48]

Marco: here, cars don't go in or anything, it's all stairs.

[00:56:53]

Marco: look at this case, the stairs going down.

[00:56:59]

[00:57:05]

Driver:  but here what you see is the maintenance of the green zones.

[00:57:12]

Driver:  There isn't constant maintenance in the green zones so all this helps the mosquito to produce too.

Marco: They come back and they repeat, it's lacking the fumigation too. Not even every two or three months are they fumigating. For here no, ooph.

[00:57:35]

Alvaro:  the people are conscious that the puddles produce dengue and all that.

Marco: the people are conscious that the puddles produce the mosquito that produces the dengue and the tires that they leave in the trash too. they are conscious. It’s that nobody is going to take a tire and throw out the water. The people leave it there.

Alvaro:  yes, even though they know, they don't do anything

Marco: even though they know. They say, "this tire, I didn't throw it away. Let the one who threw it pick it up."

[00:58:09]

Marco: we go back and continue to Terron Colorado at the church below where the road they fixed a year ago more or less is good whereas the other streets are very bad and deteriorated.

[00:58:32]

Driver:  We go down here by the bakery too. There we can go down.

Marco: yes, we can go down there below.

Driver:  it is the part most affected

Marco: the part most affected, that is most contaminated.

[00:58:47]

Marco: we are going to enter up to the part of the schools. Let’s go see that part because there are the schools and the high schools.

[00:59:08]

Alvaro:  This part has invisible borders or no?

Marco: this part no

Driver:  No. This is the principal part of Terron Colorado. Here can go anybody.

[00:59:23]

Marco: here there is no problem. one can go relaxed and enter down here in the high schools and schools over here. For here neither. Good.

[00:59:35]

Driver:  we are going to arrive to the principal school of Terron Colorado.

Marco: yes, we are going to where the high school and primary school where I studied and I fell in love with the professor. That is another dengue.

[00:59:51]

Driver:  tell them about the- we go to there.

Marco: no we go to there. here we can go out until there in the street and we come back again. We go to there and exit over in the other street. This is the part of the schools of Terron Colorado. Where the schools of high school and primary are.

[01:00:40]

Marco: but this they have fixed. This wasn't like this, no?

[01:00:46]

Marco: this is the school of Ana Maria []. This is where I studied. I did the primary.

[01:01:04]

Marco: and we go to there until that house and come back. Here there is no problem. Here there is no problem with the borders. Nothing of that here.

[01:01:11]

Marco: as you can see is relaxed.

Alvaro:  here there is no problem.

Marco: on this side, no. There aren't too many problems. That is, let’s say, it hasn't been a place like places that are from- places where you come from and take. at least look at that over there, look. That is, this place is, look, the road is wide and you don't see many problems here with gangs.

[01:01:40]

Marco: I haven't seen gangs here.

Driver:  but all this is Terron Colorado, look.

[01:01:43]

Marco: all of this is Terron Colorado.

[01:01:43]

Driver:  That is you go up there for the Variante.

Marco: this is the Variante that goes up. It goes for the side of the Villa del Mar to the 16.

Alvaro:  this way, if we continue, we will arrive to the 16?

Marco: yes, this is the one that exits to the church.

Driver:  the church where we entered.

[01:01:59]

Driver:  this has always been like this, the principal.

Marco: this is the principal way of the Via del Mar.

[01:02:04]

Marco: but this is invasion, look.

[01:02:06]

Marco: all of this is invasion but they are a few houses so . . .

Driver:  that is, it's invasion, but they are people of good living

Marco: of good living, yes.

[01:02:22]

Marco: and this is an Eco park that they made from all plastic bottles. The people of Terron Colorado. All of those plastic bottles they put there and they filled them.

[01:02:33]

Marco: all of this part down here is more relaxed. There are good parts and bad parts.

[01:03:03]

Marco: this they don't do maintenance and all of this produces mosquitoes.

[01:03:05]

Marco: yes

Driver:  all of this produces mosquitoes, the mountains.

Marco: yes,

Driver:  and as it is the same neighborhood, for that reason, for that reason the mosquito looks for where to leave the egg, the [].

[01:03:22]

[stop to change video camera batteries and sign consent forms]

[01:09:44]

[01:10:46]

Marco: OK, then we will go for the part by the schools and continue to the part of the lower part of Terron Colorado

Driver:  la ladera it's called.

Marco: La Ladera de Terron Colorado it's called.

Driver:  they are going to make a garden center.

Marco: yes

[01:11:08]

Alvaro:  They make the hole there or what?

Marco: Bring down this dirt that

Alvaro:  the waterfalls above

Marco: some waterfalls above, this all comes from the house up there, look.

[01:11:19]

Driver:  this they are [] to make. . . look lots

Marco: lots of bush on each side. Lots of bush.

[01:11:31]

Marco: lots of bush on the side of the roads that- the bottom part of Terron Colorado

[01:11:44]

Marco: this they should constantly maintain.

[01:11:48]

Marco: every month or every two months, have this grass low.

[01:11:54]

Marco: ok, here we are entering the part of the schools again.

[01:12:06]

Marco: we came back and we are again where the schools are- primary and secondary.

Driver:  in the part of the ladera.

Marco: On the right side

[01:12:37]

Marco: the boys aren't even having breakfast, they don't see us, they aren't fighting us.

[01:12:41]

Marco: this is the side of the high school. this is the side of the high school. We take this one until way down. This one takes us out to in the health post.

Driver:  to the health post and lower []

Marco: yes.

[01:13:24]

Marco: ok, as you can see, it is the same trash. the same problem.

[01:13:31]

Marco: the same problem of the people, in the way, in the streets, in the way, lots of trash. Lower part of Terron Colorado, hilly part.

[01:13:53]

Marco: the same problem, the trash. On both sides- the right side and the left side.

[01:14:03]

Marco: the high school of Terron Colorado of []

[01:14:33]

Marco: look, look at this.

[01:14:41]

Marco: they have trash and bush clogged up, no.

[01:14:44]

Alvaro:  they are protecting the dirt.

Marco: yes, they are protecting that they don't go up on the bush.

[01:14:51]

Marco: this here is a bit more relaxed, but most of it . . .

[01:15:05]

Marco: and here comes out the street, La Via del Mar.

[01:15:14]

Marco: and this is the Avenida de Ladera of Terron Colorado

[01:15:18]

Driver:  and this is the 4th street of the lower part.

[01:15:42]

Marco: Look, I think, for me, the problem here is the trash. Look.

[01:15:45]

Marco: look at all of the flies.

[01:15:47]

Marco: contamination of flies, of mosquitos. But all of it, most of it is because the people leave the trash outside of their house in the street, look.

[01:15:58]

Marco: look at the puddles that form.

[01:16:08]

Marco: very bad streets.

[01:16:22]

Marco: streets with puddles, lots of puddles, lots of trash.

[01:16:28]

Driver:  that is, in the neighborhood, the only good street is the principal one. But the

Marco: the others are very bad. and lots of trash. Look, wherever you look is trash. Look. look at the dogs. How they break the trash and that is a problem. That is the reason for the amount flies. One is having lunch in the house and lots of flies. Look, look at the mud to go from on street to the next.

[01:17:01]

Marco: lots of trash, in amount, a lot of trash.

[01:17:10]

Marco: you keep seeing the trash.

[01:17:19]

[stop to check camera signal]

Amy:  is there a lot more to see?

Marco: no, why

Amy:  because the camera doesn't have a GPS signal anymore

Marco: and if there is no signal, it doesn't record, right or no?

Amy:  it records the video but the map no.

Alvaro:  it's that it's two things- the video and the map. The video is working but the map no. But we have already done a bit.

Marco: the hard part we already did

Marco: ok, then let’s continue.

[01:20:50]

Marco: look at the dogs up there.

Driver:  the most important is the video, no

Marco: and the GPS because it makes the map of where we went. OK we are going down again the lower part of Terron Colorado on the 4th avenue on the hilly side. The same problem- trash, on both sides. Look at how the people throw the trash.

[01:21:28]

Marco: the people come and leave their trash and that’s it, normal. Done, the took it out of the house and done.

[01:21:33]

[01:22:08]

Marco: lots of puddles, it's the same- lots of puddles in this street. Lots of trash.

[01:22:25]

Marco: To mucho trash I say.

[01:22:37]

Marco: the people are drinking their beer over there.

Alvaro:  waking up early to drink.

 [01:22:55]

Marco: it's almost elven am.

Marco: here we are again where we started at the health post. Here is where we started. There is a part down there that says for those. . . over there we already went up and came down. This is the health post that we arrived to again. This is the place were started.

[01:23:40]

Alvaro:  could we stop here because here it grabbed the signal before.

Marco: stop here a moment for the GPS.

[01:23:49]

Driver:  no, but it's not grabbing.

[01:23:59]

[01:24:11]

[01:24:33]

[01:25:03]

Marco: Here we are where we started, in this moment here at the health post.

[01:25:13]

[01:25:28]

[01:25:37]

[01:25:44]

[01:25:50]

[01:25:54]

Marco: there is the part where the River Cali comes, where it [] with the River Cali. For the side above the zoo. Look, that over there. Over there it goes in there.

[01:26:08]

Marco: This is a part of Terron Colorado very well-known too and we have to see the problems of this side too.

[01:26:15]

Marco: this is a high school of Terron Colorado, a new high school, very good.

[01:26:28]

[01:26:32]

Marco: look at the streets how they are on this side.

[01:26:40]

Marco: there live my aunts, on this side, but there are many mosquitoes and many mosquitoes there too,

[01:26:51]

Marco: they come out like clouds. you have to sleep with the mosquito net

[01:26:55]

Alvaro: like where we went to collect mosquitoes that one time, in Sonso.

Marco: Yes, that is a mosquito breeding ground. But in this time in the winter they produce more. And in the day in the sunny time, it produces a lot of flies

[01:27:14]

Marco: the fly produces a lot.

[01:27:19]

Alvaro: What is the neighborhood there on the right

[01:27:21]

Marco: that is the neighborhood Santa Rita. Down here, Santa Rita, where the River Cali goes down.

[01:27:27]

Marco: and the part up there is Bella Vista. That goes up there to Cristo Rey.

[01:27:39]

Marco: this is the lower part of Terron Colorado still.

[01:27:55]

[01:28:03]

Marco: what happens in these streets here is that you go there and they are all stairs.

[01:28:10]

Marco: here we are in the portal del mar

[01:28:33]

[01:28:39]

Marco: all of this vegetation, on the side goes water drains, water from tubes, black water. Today no, but you can see that in the black water the mosquito lays a lot of eggs.

[01:29:02]

Marco: water from tubes more than anything.

[01:29:04]

Amy:  yes, they just need a little bit of water, no more

[01:29:08]

Marco: yes, in a little, in a puddle, it forms a breeding site and in this vegetation too it helps.

[01:29:15]

Marco: helps a lot that . . .. all of this is stagnant water.

[01:29:23]

Marco: from tubes that break and there the water stays stagnant and there forms the problem of the mosquito and the flies.

[01:29:33]

Marco: from here up, all of this is invasion. this is invasion

[01:29:43]

Marco: yes, look, all stagnant water from tubes.

[01:29:47]

Marco: a lot of flies and mosquitoes

[01:29:53]

Marco: this is the lower part of Terron Colorado

[01:30:11]

Marco: do you smell the smell? it smells bad.

[01:30:16]

[01:30:29]

[01:30:47]

[01:30:59]

Marco: EmCali. Yes, as you can see it is a lot of vegetation.

[01:31:12]

Elmer: All of this is invasion

Marco: This doesn't have tubes or anything. all of this falls to the river because where else is it going to fall?

[01:31:23]

Alvaro: To here, the services don’t come.

Marco: No

Elmer: all of this, even it is turned on there, look. All of this is . . .

Marco: all of this is invasion. the lower part of Terron Colorado

[01:31:37]

[01:32:04]

Elmer: This is a big neighborhood of Terron Colorado but it's invasion.

[01:32:09]

Marco: look, it's all invasion, most of it is invasion, look.

[01:32:14]

[01:32:19]

[01:32:34]

Marco: look, all of this too is . . .

[01:32:42]

[01:32:51]

[01:33:11]

Marco: the beginning of the river

Elmer: all of this invasion falls into the river.

[01:33:25]

Elmer: I believe the river is a breeding site for mosquitos

Marco: contaminated too.

[01:33:31]

Marco: all of these black waters fall into the river.

[01:33:37]

[01:33:47]

Marco: here is normal

[01:33:57]

Marco: all of that is invasion too. All of that falls into the river.

[01:34:01]

Marco: All of this invasion here like that over there.

[01:34:07]

[01:34:23]

Marco: this is a touristic zone because here enters a lot of cars to go the river and all that, no. It is a touristic entrance because it goes to the river. Then no, there is no problem here.

[01:34:35]

Marco: motors, car, bicycles enter here. Most of the people who enter here, they go to the river.

[01:34:52]

[01:34:58]

Marco: the police are coming behind.

[01:35:07]

[01:35:13]

Marco: it is big. this is big

[01:35:40]

Elmer: Look this is the []. the small one.

Marco: that cat, you take it, full of fleas.

[01:35:37]

Elmer: Look, the [] illegal mosquito

[01:35:50]

Marco: look, so many animals.

Elmer: The river look, look.

[01:36:03]

Marco: all of this is part of Terron Colorado because look at the Cali River. This is Cali River.

[01:36:10]

Marco: The Cali River and here is where, when they close the road to Terron Colorado, one comes for Palermo, on motor. Here is an exit. When they close the Portal del Mar because there they do protests. look at the river.

[01:36:34]

Marco: this is the Aguacatal. For consumption of water for the city.

[01:36:45]

[01:37:15]

Marco: yes, the river is very close the to the invasion. Then tell me where do all of these tubes go, they go into the river.

[01:37:28]

Marco: this is a rehabilitation center for drug addicts.

[01:37:44]

Marco: Yes, look at the river. This has water because is has rained. The part up there. . ..

[01:37:53]

Marco: the famous Cali River and the [] up there where they take all the water.

[01:38:04]

[01:38:18]

[01:38:27]

[01:38:41]

[01:38:49]

[01:39:08]

[01:39:19]

Marco: the famous Cali river that is next to, more than anything, this zone of invasion.

[01:39:36]

Marco: this is the bridge and one can pass there to the zoo.

[01:39:48]

Marco: The Cali zoo.

[01:39:54]

Marco: look at the boys over there having breakfast, smoking their breakfast, marijuana. this is their zone.

[01:40:12]

Marco: look, all of this, how you find it. the good thing here is that I don't see trash, almost. No, look, up there, lots of trash.

[01:40:33]

Marco: lower part of Terron Colorado, known as Palermo. All invasion. the tubes here from Palermo are going to fall into the Cali River.

[01:40:50]

Marco: contaminating the river.  Look at the river.

[01:41:03]

Marco: private security. This is part of the zoo. This is part of the zoo here. No, this is not the zoo. This is the club of EmCali. The zoo is lower.

[01:41:36]

Marco: yes, club of EmCali, this one.

[01:41:51]

Marco: this is the only entrance it has. The other entrances are stairs. This is the only vehicular entrance that the lower part of Terron Colorado has, to the Cali River.

[01:42:20]

[01:42:30]

[01:42:40]

Marco: that is the last piece of Terron Colorado. Or the part . . . because the part of the Lengua is uglier but . . ..

[01:43:26]

FILE0008.MP4

[00:02:14] A: Yes, I can see it in the lines. (())

[00:02:19] S1: Sometimes they have to (()) they leave the trash (()).

[00:02:44] S2: (())

[00:04:00]

[00:06:35] S3: ((We always keep that clean))

[00:08:30] S2: This is a part were we dedicate the half (())

[00:11:00]

[00:17:04] S4: That trash that you see there is from the ((carresquilleros)). Look at the trash

(())

[00:17:35] S2: A breeding site.

[00:17:36] S5: A cloud of mosquitoes too?

[00:17:38] S6: A lot of mosquitoes.

[00:17:40] S5: Bellow the bridge too.

[00:17:42] S6: There’s (()) and bad odor.

[00:20:23] (()) everyone who comes from the (( )) over there, this is important.

[00:23:00]

[00:27:21] S7: Exactly, more (( )) from people. They need to (( )) to clean up  and (( )).

FILE0009.MP4

[00:10:28] S: Prevention, prevention. What type of prevention do you think is important?

[00:10:28] S2: ... larva and mosquitoes

[00:11:10] S: And from the community,

[00:11:45] S3: That is one of the subjects from the community board.

[00:12:01] S3: We don&#39;t time but we are available (()).

[00:12:25] S3: In that wall there, there is the (()).

[00:12:31] S4: (()) of water

[00:12:36] S5: The water has a... and there the water enters

[00:12:44] S4: Then that&#39;s it. What you have to do is prevention and education and

capacitation.

[00:12:56] S4: But how do you bring the word. You give them education and how do you know

that what you are teaching about

[00:13:03] S4: They are actually going to implement it. For example I can come and educate

but how do we do so they can do it.

 [00:14:00]

[00:14:20] S5: We once had a case the first time ...(())

[00:14:25] S5: The government did it the first time when ...(())

[00:14:44] S5: So we can with them...they can get a group of youths and give them ((water))

[00:15:49] S6: It is to say that what I propose is that form them

[00:15:55] S6: as promoters in environment health so they can start

[00:16:03] S6: So they can repeat the information, organized by sectors

[00:16:08] S6: or blocks or zones and they can be in charge so people can understand the

information

[00:16:23] S7: talks and talks...what other type of education can they explore other than talks,

what others can you use?

[00:16:32] S8: Videos.

[00:16:33] S7: Videos, what else?

[00:16:45] S7: Maybe a cultural center, it&#39;s called the house of (()).

[00:16:50] S7: Use this house for theater or something else that catches their attention.

[00:16:58] S7: And no a talk as a format. Could you think of that?

[00:17:07] S5: We have proposed to the initiation and we have gotten out the project. The

project

[00:17:13] S5: is in the matrix of the development plan of (()). And is a sport center.

Directory:

C:\Users\Amy\Google Drive\Kent\james\dissertation\chkv and dengue\data\video

data\ValleGrande_VillaDeGrande_Dec4

Audio Files:

ZOOM0001.WAV

Associated video files:

1. FILE0271.MOV

a. Transcript: [00:03:21] David: Valle Grande is not a very old neighborhood. It has

more or less 15 years.

b. Video: [00:00:41]

2. FILE0020.MOV

a. Transcript: [00:03:21] David: Valle Grande is not a very old neighborhood. It has

more or less 15 years.

b. Video: [00:00:33]

Neighborhood: Valle Grande

Date: December 4, 2015

Interviewer: Amy Krystosik

Driver, vector specialist: David

[00:03:21]

David: Valle Grande is not a very old neighborhood. It has more or less 15 years.

[00:03:36]

Amy:  very new.

David: And these house they made out of social interest.

[00:03:41]

David: Using a compensation account. That is, the workers contribute there and by taking out

from there they can take loan for the house.

Amy:  that’s great. yes, all of the houses are beautiful and new.

[00:03:58]

David: Yes. And this part over here on this side is part of canal behind it.

[00:04:06]

David: That&#39;s the commercial center of the Cauca River. Apart of the river passes behind there so

what they wanted to do with that was a shipping dock. To bring the market by the river. But in

the end it didn&#39;t work.

[00:04:20]

[00:04:31]

[00:04:35]

[00:04:56]

David: this is where they are going to make a lagoon. They are going to remodel a part of the

canal and make it there.

Amy:  for what?

David: to decontaminate the water more to put it in the river because that is there reuniting with a

canal.

Amy:  as part of the treatment.

David: Yes

Amy:  Good, they are going to control that then

[00:05:37]

[00:05:49]

Amy:  very tall grass.

[00:05:58]

[00:06:04]

[00:06:25]

[00:06:45]

[00:06:58]

[00:07:12]

Amy:  abandoned

David: a recreation center here.

Amy:  an Eco park. But it looks like it doesn&#39;t work anymore

[00:07:25]

David: it’s that the people aren&#39;t conscious with the trash. It’s everywhere.

[00:07:32]

David: and this is part of the dyke that is part of the Cauca River.

Amy: The dyke of Valle Grande.

[00:07:43]

[00:07:59]

[00:08:16]

David: this is a high school.

[00:08:21]

[00:08:50]

Amy:  Then the people here, from where did they come?

David: They were workers from all over the city.

Amy:  from all the house who wanted a new house so they came here.

David: Yes, through the compensation account they got the house.

[00:09:08]

[00:09:15]

[00:09:39]

[00:09:57]

[00:10:16]

David: this is the dyke.

[00:10:21]

David: The dyke that I saw that was most organized was that one in Floralia, no?

[00:10:27]

Amy:  Yes, it was very organized with business and everything. Do you think we could go into

the dyke too or no?

David: If there is an entrance, we can go.

[00:10:45]

[00:11:07]

Amy:  Here in this neighborhood, there are not many people. They are all working or in the

school or something.

David: Yes, they are very hard working people.

[00:11:18]

[00:11:46]

[00:12:42]

[00:12:51]

[00:13:45]

[left mic in car during tour of farm]

[00:23:00]

[00:23:35]

[00:24:18]

[00:24:39]

[00:25:24]

[00:27:21]

David: behind the dyke where the trees are, that is where the river starts.

Amy:  Yes, it is very far from here and I see that there are no mosquitoes here in this moment for

the rain. But I think that yes

David: there should be.

Amy:  With rain, yes.

[00:27:39]

David: and in with those holes that they make in the ground like there were back there, I think

they are foci too so that they produce.

[00:27:53]

David: and there are parts where they put animals and they don&#39;t change it with frequency but

they leave them there stagnant.

[00:28:03]

David: this side of the dyke is ok but the part up there is very dangerous, you can&#39;t go in there

Amy:  No, this part seems very organized. As the lady said, she has 30 years working there in her

farm. More farmland than anything.

[00:28:52]

[00:29:25]

[00:30:38]

[00:32:04]

Amy: but it is also interesting that each neighborhood has its own type of business- here it is

more farming and less recycling.

David: Yes. Floralia was like this too, all woods. Look at the beds. All beds for children in

wood.

[00:32:47]

[00:33:27]

[00:33:37]

[00:34:07]

[00:34:51]

David: Look, they said that it is CVC that is bringing all of those materials with those cars.

[00:34:56]

David: they are throwing it throw.

[00:35:10]

David: they are growing to throw everything there. This is from a part where they are building

something better and they come and they throw everything here.

[00:35:31]

[00:36:18]

[00:36:42]

David: look at this tower of electricity- very big.

[00:36:49]

David: The danger over there [] it would be very bad if something made that tower fall.

[00:36:55]

Amy:  It is dangerous?

David: Of course, it all current of high voltage. That shouldn&#39;t be there.

[00:37:06]

[00:37:56]

[00:38:28]

[00:38:45]

David: over there is another piece of dyke but the dyke over there is more dangerous. The is a

more populated dyke.

[00:38:55]

David: They are dropping off materials too, I think.

[00:39:09]

[00:41:27]

[00:41:41]

[00:42:18]

David: The only thing that I see in Valle Grande that has this is that part of the dyke. Because for

here it is all houses and parks.

[00:42:32]

Amy:  Yes, I think it’s the dyke too. You are right.

[00:42:45]

[00:44:39]

Amy:  This looks like a country neighborhood.

[00:44:39]

David: but look this neighborhood is . . .

Amy:  very open and organized.

David: This is the park where the people take out the trash.

Amy:  OK, good.

[00:44:50]

David: and over there is Calimio de Cepaz- a dangerous neighborhood

[00:45:03]

David: Over there they say it looks []

[00:45:05]

[00:45:54]

[00:47:14]

David: and this is the commercial part- all businesses.

[00:47:25]

[00:47:52]

Amy:  the condition of the street is good. Sometimes I don&#39;t understand why there is dengue. I

can&#39;t understand well what is happening here.

[00:48:15]

David: all of these parks this way so overgrown would help. It’s that in these there can be bottles,

things there.

[00:48:28]

[00:51:24]

[00:53:45]

[00:53:59]

David: Valle Grande center for studies

[00:54:10]

[00:55:41]

Amy:  this lot- there are a lot of lots that seem.

David: it is overgrown.

Amy:  yes

[00:55:48]

[00:56:29]

[00:58:05]

[00:59:30]

[01:00:34]

[01:00:39]

[01:00:51]

[01:00:59]

[01:01:03]

[01:01:13]

David: but this is beautiful, look, right?

Amy:  Yes, very beautiful with the park and the palms. What is this?

David: This is a junk yard to scrap cars.

[01:01:29]

Amy:  a very good breeding ground.

[01:01:32]

David: of course, this has where to breed

Amy:  everywhere.

[01:01:34]

David: if it rains, the puddle stays in the car.

[01:01:38]

David: do you want to see in there the junk yard? That is on the border of Valle Grande.

Amy:  yes.

[01:02:14]

[01:03:02]

[01:03:06]

Directory:

C:\Users\Amy\Google Drive\Kent\james\dissertation\chkv and dengue\data\video

data\ValleGrande_VillaDeGrande_Dec4

Audio Files:

ZOOM0002.WAV

Associated video files:

1. FILE0272.MOV

a. Transcript: [00:00:37] David: here is where they killed the archbishop Isaias

Duarte Cancino who was very famous in the world and everything and in all

Colombia.

b. Video: [00:00:36]

2. FILE0021.MOV

a. Transcript: [00:00:37] David: here is where they killed the archbishop Isaias

Duarte Cancino who was very famous in the world and everything and in all

Colombia.

b. Video: [00:00:34]

Neighborhood: Villa de Lago

Date: December 4, 2015

Interviewer: Amy Krystosik

Driver, vector specialist: David

[00:00:37]

David: here is where they killed the archbishop Isaias Duarte Cancino who was very famous in

the world and everything and in all Colombia.

[00:00:43]

[00:01:06]

[00:03:38]

Amy:  I think they could use the canal for something different.

David: but its black water.

Amy:  they should clean it and use it for transport or something.

[00:03:59]

[00:04:38]

[00:05:43]

David: that is a recycling company up there.

Amy:  a big business

[00:05:50]

David: Recycling is a business that is ...

[00:06:03]

[00:06:35]

David: These are the first houses of Villa del Lago and the people put those gates there like that

because Charco Azul is there on the side of Villa del Lago and in Charco Azul live like bad

people then the owners of the houses here have the keys to those gates. Look they have put up to

[] so that the thieves don&#39;t enter there.

[00:06:57]

Amy:  then they close their community and nobody else enters

David: yes, nobody except the owners of that pedestrian street.

[00:07:02]

[00:07:13]

[00:08:03]

David: look, this is Villa del Lago.

[00:08:14]

David: And this neighborhood is a bit older. . ..

[00:08:27]

David: the mayor changed all the addresses here.

Amy:  then I am going to have more problems with these addresses.

David: they have double addresses. Before it was 24d and they changed it to 24a1

[00:08:57]

[00:10:53]

[00:11:20]

Amy:  they I have the doubt if the people are reporting their old address or the new one.

David: no, this has to be the new one.

[00:11:33]

[00:11:54]

[00:12:55]

Amy:  and this is part of

David: Charco Azul

[00:13:12]

Amy:  and why Charco Azul doesn&#39;t have dengue? that they are not reporting? [note- a

conversation with a community health worker later explained that Charco Azul has a displaced

population from the afro descendants of the pacific coast area of Buenaventura. I am told that

they prefer to stay closed within their community rather than report to the health post. They also

visit the local shaman for health issues.]

[00:13:37]

[00:14:28]

[00:14:51]

Amy:  I think it’s that these parts that we saw before that has the dirty lake and the invasion also

have dengue but they are not reporting but here they have dengue for the location and they are

reporting it. It is the only thing I see. There is no other way.

[00:15:30]

David: The recreation center of Villa de Lago.

Amy:  beautiful.

David: It&#39;s big. It has a swimming pool and everything.

Amy:  this is from Comfandi

David: no, from the city.

[00:15:45]

David: all Villa del Lago has Charco Azul next to it. Look, that is part of the lake.

[00:15:58]

[00:17:58]

David: all of Villa del Lago is like this with pedestrian streets. There is no way to see anything

except the border of the neighborhood. Because from here to there is another neighborhood. And

from here to there is Villa del Lago. And over there is Los Lagos that has houses.

[00:18:22]

[00:19:16]

[00:19:32]

David: that is a hospital.

Amy:  yes, it is the hospital.

[00:19:45]

[00:21:18]

[00:22:02]

[00:22:30]

Amy:  the good thing is that we have already seen the lake and the canal and we already know

what is all around this neighborhood.

[00:22:52]

[00:23:21]

David: between the Laguna and El Pondaje it is the same Charco Azul that divides it

Amy:  in two parts

David: yes

[00:23:30]

Amy:  And although it looks like an invasion, it has an organization, a name. The mayor

recognizes it.

[00:23:52]

[00:24:02]

[00:24:07]

[00:25:05]

[00:25:45]

Amy:  junk yard

David: bronze, aluminum, copper, they buy everything

[00:25:55]

[00:26:28]

David: this street we were on the other day, remember?

Amy:  yes, from the SENA

David: the study center of the SENA

[00:26:37]

David: Amy, I think we have covered all of Villa del Lago.

Amy:  ok.

[00:27:05]

https://drive.google.com/folderview?id=0Bza3s3VWuatLa3EzQUpsMEVrdXM&amp;usp=shar

ing

Neighborhood: Vergel

Date: April 10, 2016

[00:00:05] A: We are in Vergel and today is April Tenth. We are in the rain season.

[00:00:12] A: (()). Let&#39;s see what there is here.

[00:00:20] A: In these days it is raining a lot, and the canals are getting bigger or what?

[00:00:27] D: Yes, they get bigger. The level rises.

[00:00:32] A: And there is flooding or not?

[00:00:34] D: The flooding happened for like four days in other neighborhoods.

[00:00:39] A: But not here.

[00:00:40] D: But there was not flooding here

[00:00:44] A: And you don&#39;t see that much water in the streets.

[00:00:47] D: No. But you can see the humidity in the sides.

[00:00:53] L: On the sides of the platforms.

[00:00:56] D: The sides of the platform, you can still see the humidity.

[00:01:04] D: Is the entire street that looks wet. And some water like that that get

accumulated.

[00:01:11] D: On the side of the road.

[00:01:14] A: And do they keep being in higher control of vectors for Zika or no?

[00:01:19] D: Yes, they&#39;re still doing it. But that is like putting a warm cloth on it.

[00:01:28] A: Not that strong.

[00:01:30] D: And sometimes they do it in general to all cities, the do not take into accounts

hot spots

[00:01:36] D: or anything for the to get to that part, if not they just do it in general to all of

the city.

[00:02:37] D: And what Vergel and Poblado have is all of this part of the canal. They have many

streets with out pavement.

[00:02:45] D: Then I think, how we passed through the street that is ugly and it has holes and

puddles

[00:02:51] D: And water accumulation.

[00:02:55] L: And tires.

[00:03:18] A: And here is the canal.

[00:04:22] A: Is there a lot of stagnant water?

[00:04:24] D: Yes.

[00:05:18] A: I think that there is more trash today because it is Sunday.

[00:05:25] D: Yes because they pick up trash here three times a week.

[00:05:32] L: Even thought there is more trash outside.

[00:05:35] A: And a lot of constructions.

[00:07:31] D: Look what I told you, that there are little puddles like that after the rain has

come.

[00:07:39] D: And I think if that last three days, there are easily mosquitoes reproducing there.

[00:07:45] A: Very fast.

[00:07:47] D: Look at the blocks. If they see puddles of rain.

[00:07:53] A: And they look for water everywhere.

[00:08:01] D: And look, here the pavement is bad and there are even more.

[00:09:24] D: You see them because of accumulation of rain from these days.

[00:10:00]

 [00:11:21] D: There is a lot of stagnant water in the streets, no?

[00:11:23] A: A lot.

[00:11:30] A: Yesterday it rained in the morning, and I imagine that lasts like two or three days

here.

[00:11:36] L: But is that from yesterday or last night.

[00:11:42] D: I don&#39;t think it rained last night because I live close by and it did not rain.

[00:11:46] L: Yes?

[00:15:14] A: Tires, tires, tires...

[00:15:16] L: That is the common factor, the tires.

[00:15:28] D: There are a lot of tires thrown around. Look, another tire there.

[00:15:58] A: Look how pretty that dog.

[00:16:03] A: They have like four there.

[00:16:05] L: Yes. The more poor the more dogs.

[00:16:11] D: Those are the habitants of the street that gather the filth and everything, paper,

and recycle

[00:16:16] D: And they have two or three dogs behind them.

[00:18:21] D: This is the last corner of Vergel. And this is the canal.

[00:19:00]

[00:20:13] D: Let&#39;s look at the canal here.

[00:20:25] D: All of that is the order of Vergel.

[00:20:26] A: And that water is stagnant.

[00:20:28] D: Yes, of course. And since it has rained is even worse. The trash that they throw

out is the one that stops the water.

[00:22:24] A: And the mosquitoes are biting more?

[00:23:00]

[00:24:00]

[00:25:00]

[00:26:00]

[00:27:00]

[00:28:00]

[00:29:00]

[00:30:00]

[00:30:23] D: That is the last corner of Poblado Uno and look how the canal is there.

[00:30:30] A: The park is well maintained. It had all of its grass cut.

[00:30:36] D: Look this is the canal.

[00:31:09] D: After they made the hospital, they organized everything here. They took out a lot

of the houses that looked like small ranches.

[00:31:17] L: Ranches.

[00:31:19] D: That is seen a lot to make this street. There were more houses until the shore of

the canal.

[00:31:30] D: Noticed that you see a lot of houses there, and in the border of the ground.

[00:31:42] A: And when did it change?

[00:31:45] D: They remodeled the hospital five years ago.

[00:31:49] A: Not a log time ago.

[00:32:05] A: But here the water is moving, right?

[00:32:12] L: Yes. It is not stagnant. The water is moving. But because it comes from where?

[00:32:18] D: That is from the houses. That is from the drain of the houses.

[00:32:33] A: This one comes from the other canal?

[00:32:36] D: Yes. Everything comes from the primary drain from the houses.

[00:32:46] D: There are tires. (())

[00:32:50] L: Furniture...mattresses...

[00:33:03] A: (())

[00:33:15]

[00:35:00]

[00:37:00]

[00:38:56]

[00:39:00]

[00:40:00]

[00:41:00]

[00:42:00]

[00:43:00]

[00:43:31] A: Here everything is closed, but I imagine that behind those doors there are open

gardens, correct?

[00:43:39] L: Here, gardens? No. The houses?

[00:43:43] D: This zone is like that, look.

[00:43:45] A: OK. But behind, in the blocks, there are not.

[00:43:56] L: This houses are really small. They are constructed by eye by people that

[00:44:02] L: did the invasions and all of them construct the house however they can.

[00:44:14] D: And there is the border of the river.

[00:44:15]

[00:44:00]

Leaders-vergel-athome.WAV

Neighborhood: Vergel

Date:

[00:00:00]

[00:01:00]

[00:02:00]

[00:03:00]

[00:04:00]

[00:05:00]

[00:06:00]

[00:07:00]

[00:08:00]

[00:09:00]

[00:10:00]

[00:11:00]

[00:12:00]

[00:13:00]

[00:14:00]

[00:15:00]

[00:16:00]

[00:17:00]

[00:18:20]

[00:18:27] S1: Have you recognized hot spots that call for the production of the mosquito?

[00:18:35] S3: In this area is a little

[00:18:41] S3: more cleaner for the mosquito in that area because

[00:18:48] S3: I think where it is most reproduced is for example in sinks.

[00:18:54] S3: The sinks are for evacuating rain water mixed dirty water.

[00:19:01] S3: And what is left becomes stagnant

[00:19:07] S3: and the mosquito more than anything is produced in still clean water.

[00:19:13] S3: And in the places where there is a lot of trash and in the

[00:19:20] S3: mountains, they reproduce.

[00:19:21] S2: Then the houses that are more exposed are next to the.

[00:19:25] S3: The border of the canal.

[00:19:26] S2: The border of the canal.

[00:19:27] S3: And the other thing is that the mosquito is

[00:19:33] S3: something that reproduces itself in the house because it is more nocturnal than diurnal.

[00:19:40] S3: Yes? The mosquito comes out more at night. And is where I more-- I mean,

[00:19:47] S3: to apply fumigation at the hours of the night. But

[00:19:54] S3: with the information towards the community,

[00:20:00] S3: that they open the windows and the doors, yes? So it can penetrate.

[00:20:07] S3: Because its effect I can tell  works when-- the quality of

[00:20:14] S3: applicant, the poison they are using, is decreased to a ninety percent.

[00:20:21] S3: So be it-- Let me explain myself. The secretary can

[00:20:27] S3:  give them the tin and the ones who prepare it, reduce it

[00:20:34] S3:  because it is very costly. With one liter

[00:20:40] S3: they can make ten, to tell you something. They are getting twenty.

[00:20:47] S1:Who does the fumigations of

[00:20:51] S2: The secretary.

[00:20:52] S3: And they are taking twenty and then there's nothing. There's no (( genre)). Because that happens and the mosquito bites.

[00:20:59] S3: Using that and the mosquitoes get mad because they do not kill them.

[00:21:04] S2: The fumigations normally are done in between five in the morning

[00:21:08] S3: At five i the morning when everything is closed.

[00:21:10] S2: Yes, like at five in the morning. But like he says they do not inform. They should inform so we can know.

[00:21:16] S2: And the idea is so that the fumigation can go in and we know to have our pots close and everything

[00:21:22] S1: When you say that the concentration is decreasing is because you notice there is no effect?

[00:21:30] S3: There's no effect. They are very reduced. That's why I am saying that from the hundred percent, the ninety percent.

[00:21:35] S1: And have you seen when they decrease it or.

[00:21:37] S3: No, because, at least I worked for ten year in the area

[00:21:43] S3: of chemicals, of contaminated water and you know.

[00:21:50] S3: Yes? Because it is something effective like drugs. The genetic and the

[00:21:57] S3: And the other, yes? Then that is what happens with that fumigation.

[00:22:04] S1: You have the same form

[00:22:07] S2: No, I in the part I do not know. On behalf of chemicals like he knows, I am not an expert.

[00:22:13] S2: But that the fumigation now works like it used to, no. It was more effective before.

[00:22:19] S2: I do not know how everything now a days it reduced. Everything is not the same. The same quantity

[00:22:25] S2: That they put on everything.

[00:22:27] S3: I would say that if they apply it well. it does more effect and there's more productivity.

[00:22:34] S3: to combat all of those phenomenon.

[00:22:40] S1: And the idea is for them to tell you when they are visiting..

[00:22:43] S2: That the community knows. The truth is that is very important, is a surprise factor and they are doing it in the dawn.

[00:22:55] S4: There's a responsibility from the state to fumigate.

[00:23:02] S4: But it is understood that the problematic is not just resolved with fumigation. What do you

[00:23:10] S4: think should be done from each person that has its house so sinks do not exist. In example.

[00:23:17] S4: Which one-- you are leaders of the sectors then

[00:23:23] S4: what is the responsibility of the population.

[00:23:26] S3: Well, so sinks cease to exist would be a mechanism of planning

[00:23:33] S3: Planning, because planning it not like it used to be. Before, if you

[00:23:38] S3: That go through the streets and they are old, because you know and others that are new.

[00:23:45] S3: The new ones get ruined first than the old ones. Yes, because they applied a

[00:23:51] S3: material of good quality. Now a day no, it is to say,

[00:23:59] S3: the contract mandates it. If I do things correctly, they are not going to call me in six months or a year.

[00:24:06] S3: But before, I would think of that, because the paving would be done with the

[00:24:13] S3:  community and they would be well made. That is what exists in that phenomenon.

[00:24:21] S4: Then that would be saying that the mosquito

[00:24:27] S4: is produced outside and not inside the houses.

[00:24:30] S2: It is also produced inside the house.

[00:24:33] S4: And how is it produced.

[00:24:34] S2: She says, how would I contribute from my house to avoid the mosquito.

[00:24:40] S2: I should not have stagnant water. The people who have vases, the plants.

[00:24:47] S2: At least I have a water plant. A bamboo. I change it everyday due to the Chikungunya.

[00:24:54] S2: Dengue. I live with my daughter and she is ten years old. I take a lot of care so neither of us get that.

[00:25:01] S2: Because is a disease that is really heavy, in the house everyone has gotten it. There are four apartments and everyone has gotten it.

[00:25:07] S2: Thanks to God, I have not gotten it. Not Chikungunya, Dengue nor Zika.

[00:25:14] S1: The education process to the community, you as community leaders

[00:25:20] S1: do you manage that type of knowledge.

[00:25:26] S2: Of behavior, that yes.

[00:25:29] S1: Yes, of behavior through education or do you wait

[00:25:35] S3: I will tell them. It results and it happens

[00:25:41] S3: that for the application

[00:25:48] S3: the administration never takes into account the leaders. Never. Those are the ones that know where

[00:25:54] S3: the production is of the effect that is affecting the population.

[00:26:00] S3: They never. They prefer to spend the money on publicity

[00:26:06] S3:  and not call the leaders to look for people to go with us and show us

[00:26:13] S3: where the hot spots are. With one hot spot that contaminates one quarter of the city of Cali.

[00:26:20] S3: They never do that, if you hear the secretary of health, I walked and went

[00:26:27] S3: and they go to Papayera, but what those that serve for? Celebrating parties to the mosquitoes.

[00:26:32] S3: That is ridiculous to put on TV.

[00:26:39] S3: If you look at the seventy percent of officials that they named,

[00:26:46] S3: they are afraid to go the neighborhoods, the sidewalks. They're afraid.

[00:26:52] S3:  Because they do not know what is their job to start with.

[00:26:58] S3: Second, with the image they have, which s what the lady here says, that she walked

[00:27:04] S3: around all of Vergel and nothing happened to her. But they do not go.

[00:27:12] A: That is what we want to do with you guys. Recognize the neighborhood more. And since we already went

[00:27:19] A: there how many times, with _____ and _____ and more people.

[00:27:27] A: that was really costly and I paid a taxi to go there.

[00:27:33] S2: Have you gone there alone.

[00:27:39] A: No.

[00:27:40] S4: With some partners of the secretary.

[00:27:42] S1: In a car doing the video.

[00:27:44] S2: You have not gone walking?

[00:27:46] S4: No because she needs to have those images

[00:27:52] S4: Then she has done that in polarize cars so she

[00:27:58] S2: For security and everything.

[00:28:00] S3: But is really good, principally I

[00:28:06] S3: would offer security

[00:28:13] S3: Because we can get a quadrant during what we can do and look at the places

[00:28:20] S3: Where the hot spot of the disease is. Where it is born.

[00:28:27] S3: Yes, where it is born. There we have to focus on it and look at it.

[00:28:31] S2: That's why I would ask if you have gone alone because in the neighborhood my dad is the president of the community board.

[00:28:36] S2: Always, when you are going to go, political figures, the secretary of different places that have to go

[00:28:43] S2: people are scared of the neighborhoods where we live, always my dad goes with them they have never gone like you just told me.

[00:28:50] S2: That they go on a polarize cars, that's why I ask you. In my father's case, a lot of people go that

[00:28:57] S2: have panic, but an organized fear, but my father guarantees security because people in the neighborhood respect him a lot.

[00:29:04] S2: They do not go in between my father because they know my dad does social work in the neighborhood. Then, if you are going to go

[00:29:10] S2: with him, is it in Poblado or Vergel, because my father has been there since I was a little girl.

[00:29:16] S2: And now since, well that was second time my dad goes, right?

[00:29:20] S3: The fourth time.

[00:29:22] S2: But now, since he started again. When I was little my dad would always be the communities board president. He would end

[00:29:29] S2: And then again. People follow him a lot. And now that we are old and I am about thirty years old.

[00:29:36] S2: He was about two years that we came back to the board. My dad puts a lot of effort into it. That's why

[00:29:44] S2: he lasted about fifteen to twenty years that he left that. But is the way he gives himself to the community. He even gets sick and we have to give him chicken.

[00:29:51] S2: Because he does not eat to go to meetings and I tell him _____. If it is not like that and he gives himself.

[00:29:57] S3: I am, I am the mayor, no?  And I have access to the

[00:30:03] S3: twenty-eight neighborhoods and the eight settlements.

[00:30:11] S3: That are the parts that are better to film.

[00:30:17] S2: And is it better if they to it while walking.

[00:30:21] S4: This study did not get settlements.

[00:30:24] S3: They did not give them?

[00:30:25] S4: They gave it Calipso its zone. The one after the health center, that

[00:30:32] S4: adjoins with Yira Castro. They gave them that.

[00:30:39] S4: And Vergel adjoining with another section.

[00:30:40] S2: With Poblado.

 [00:30:42] S4: But they did not give them the settlements.

[00:30:44] S2: But look at the video that she shows us, she practically passed-- that is the wide street of Vergel but for you

[00:30:50] S2: to see the sinks, what I tell you of the streets that are deteriorated that are, what he says

[00:30:56] S2: the cement is not like it used to be. They make a new streets and it get's ruined faster, like he said.

[00:31:02] S2: The new, because they do not put the same amount of components in the cement then you go in.

[00:31:08] S2: to the neighborhoods, so you can see the state of the canals, everything. That only when the rain is about to start

[00:31:14] S2: I do not know who pays them, and normally I do not see them with a identified uniform, normally I see that they are the

[00:31:20] S2: crazy. People like that, that you see and that

[00:31:26] S2: you see them pass with their hand like that, without any protection and they take the trash out of the sewers

[00:31:33] S2: And then so that streets do not get clogged and they passed through the houses and we give them the coin.

[00:31:38] S2: That's what they do. But not with a uniform. Someone that the secretary sent or something from health, no.

[00:31:44] S2: Then what do we do? We collaborate with them because they are collaborating with us, because when it rains a lot the streets get clogged.

[00:31:50] S2: and you have to get the water out a bucket with things, then if you walk and go in the houses

[00:31:55] S2: and look at the true stages, moreover, you are going to photograph or record the puddles

[00:32:01] S2: that are there, you will understand more clearly what we are doing. Then people with who to walk with complete security there is.

[00:32:08] S2: The truth is yes

[00:32:09] S3: Look for if you

[00:32:13] S4: That Vergel from the side in between Diamante and Vergel...

[00:32:17] S2: Yes, because there is the school of...

[00:32:21] S4: The school of the nuns.

[00:32:22] S2: So much fight to get that paved and nothing.

[00:32:24] S3: Look, search for two people, two photographers and

[00:32:30] S3: If you want don't go, and with them we will do social work

[00:32:36] A: Yes, sir.

[00:32:37] S3: We will help him. I promise to help you with that.

[00:32:40] A: That is what we want to do.

[00:32:42] S3: And we will give you security, so you are the number one in this project. and you can.

[00:32:49] S2: And to do it like it should be done. Because how you have to do it because you have not have the support, no.

[00:32:57] S2: You do it like you have to, walking you are going to find more of what you are looking for,

[00:33:00] S4: I assure you that when where we showed the school a block from there is the H that they put

[00:33:07] S4: in that side there, from that block, is where they are more present

[00:33:13] S4: but is the zone with most risk in Vergel. Right? But it is there where we need to go.

[00:33:22] S4: You understand? Because what you are filming there is big streets.

[00:33:30] S4:Is, I do not know later it did not let me open the archive to look directly at a direction.

[00:33:48] S4: You have an explanation that because of the bad constructions of the track it forms puddles that

[00:33:54] S4: That promote the creation of mosquitoes.

[00:33:57] S2: The streets in the block where we live there's a puddle that gets form. Furthermore, you clean the porch

[00:34:03] S2: And the puddle is there. You have to take the broom and take it to the aperture.

[00:34:10] S2: The sewer, so it does not stay.

[00:34:12] S4: It is not all about taking the water away but also using the chemical for the larva

[00:34:18]

[00:34:31]

[00:35:01]

[00:35:35]

[00:36:01]

[00:36:14] S2: That tire, is a breeding site because they get full of water, those tires.

[00:36:19] S4: They have made some parks

[00:36:21] S2: They are breeding sites.

[00:37:00] S4: If we go inside a house, I cannot be completely secure,

[00:37:07] S4: but I am almost sure that we will find the mosquito.

[00:37:11] S3: It is true.

[00:37:13] S1: What mechanism do you think are important when she says they are inside the house

[00:37:20] S1: collective mechanisms so the community can start to revise the subject of their own house.

[00:37:27] S2: At least in our case what the news say a lot, not to pay attention

[00:37:33] S2: to what the news recommends or the commercials, which they insist more

[00:37:40] S2: in the breeding sites, not let water become stagnant, avoid puddles, all of those things, it contributes to us doing it at our house.

[00:37:47] S2: I at least fumigate a lot. Because I do not like bed nets because of the heat

[00:37:53] S1: There's bed nets inside of the community?

[00:37:56] S2: Yes, at least my mother uses them. I do not. I live alone with my little girl, she is ten, and she gets to hot to go to sleep

[00:38:03] S2: So, practically we have never used it. In my house we almost never see a mosquito. You barely see it because I fumigate a lot.

[00:38:10] S2: I fumigate enough. But is a chemical. I used Baygon, which is very effective.

[00:38:18] S2: I fumigate a lot. Almost all the time. When the girl is studying then if I am in the house I take advantage. In this moment I am not working.

[00:38:24] S2: I always work but not in this moment. So in the times that they girl is not here, I fumigate the house, so my house is very

[00:38:31] S2: rarely when you see a mosquito. But I try to take the precautions with the recommendations they do. But we could

[00:38:37] S2: we could talk, look for which neighbors, well we would have to talk to them but we would sure find people

[00:38:43] S2: That will grant the access for certain people to go inside their house

[00:38:49] S2: So we can identify ourselves if in the houses they are collaborating with that because the mosquito doesn't just come from the outside

[00:38:55] S2: We also produce them from inside the houses and is not just two or three and they all go flying away because they are not going to stick in the house where they well made.

[00:39:00] S1: I remember when we were little that public health would go inside the houses.

[00:39:07] S1: Correct? They do not do that anymore?

[00:39:12] S2: It has been a lot of years. But now a day you do not see it. Now a day what happens is what he says, the people who participate are from their own community

[00:39:20] S2: board, which is the first filter to talk and say that they are going to go to the neighborhood.

[00:39:24] S4: No, but they are not the first filter.

[00:40:00]

[00:41:00]

[00:42:00]

[00:43:00]

[00:44:00]

[00:44:08] S1: And that applied to the Dengue situation, how would that

[00:44:13] S3: Dengue?

[00:44:23] S3: I would say that the secretary of health ((boycott))

[00:44:30] S3: Is going to back away. Because it is a thing, specially you who are in the process,

[00:44:36] S3: When is not your field, if not another area, that are doing things really bad.

[00:44:42] S3: Very badly applied. First I go back in what I said before

[00:44:48] S3: A lot of publicity there is nothing of application to really disappear

[00:44:54] S3: what is finishing with the community. Specially, the popular house

[00:44:59] S3: The marginalized class, which is the one that suffers the most. Because you here

[00:45:06] S3: you have an economic medium, you can buy detergent for twenty or thirty thousand dollars and you apply it

[00:45:12] S3: and then that makes them go away. In the marginalized sector, what they earn is twenty

[00:45:18] S3: or fifteen thousand pesos and if they buy the detergent then they do not have with what to eat

[00:45:24] S3: Then, I think that in that case they let the community participate that really is in the problematic

[00:45:32] S3: they would apply it correctly and it would end. It would not suddenly end

[00:45:39] S3:  but would decrease an eighty percent.

[00:45:45] S3: Because the diseases aren't detected quickly.

[00:45:47]

[00:46:00]

[00:47:00]

[00:48:00]

[00:49:00]

[00:49:06] S1:  Talking more about the influences and pathologies

[00:49:12] S1: of Dengue, Chikungunya or Zika, do you know cases close to you that

[00:49:18] S1: you know of? How is the management that has been given...

[00:49:27] S1: A specific case.

[00:49:30] S2: From the people who have gotten sick

[00:49:32] S1: Yes, that you of a case, what has been the attention route for example that they have had

[00:49:38] S1: Or those it happen a lot that they use not the doctor the route with the health center,

[00:49:46] S1: if not they say oh, I know this is Dengue and they auto-medicate that they say,

[00:49:54] S2: They do both.

[00:49:57] S1: That they do not know the difference between Chikungunya...

[00:49:58] S2: They do both, they auto medicate and they go to the doctor but people complain that they give them the same

[00:50:04] S2:  they give them acetaminophen, then people get it and why would they go

[00:50:10] S2: They auto medicate themselves, they take acetaminophen and homemade remedies.

[00:50:15] S4: Which?

[00:50:16] S2: They use matarratón a lot, which is a plant that nowadays in the places where it was abundant

[00:50:22] S2: there is non. With these disease people go, they grow in the canal's shore, they go there and take them

[00:50:28] S2: and they prepare in the way they have told them.

[00:50:30] S1: Where the mosquito is produced, there is the matarratón.

[00:50:34] S2: Because it is in the shore of the canal where most of the mosquitoes are. And it is very difficult to get it.

[00:50:40] S2: The plant, in the canal's shore.

[00:50:55] S1: And the community knows the differences between those diseases? How you should treat them?

[00:51:18] S3: Because I had Dengue. I had to support myself with the wall so I could walk and I would fall down.

[00:51:25] S1: How did you know it was Dengue?

[00:51:29] S3: Because I went to the doctor and they said it was Dengue so they gave me like ten acetaminophen pills.

[00:51:36] S3: And I told them that it was not working and I took ten or fifteen a day, so imagine.

[00:51:41] S2: But a lot of people do not say that they have the disease. He said he went to the doctor

[00:51:47] S2: But a lot of people do not go, if not someone told me that it hit them like this so that means I have Dengue.

[00:51:53] S2: Not I got Chikungunya. In my block I have a friend that is like that.

[00:51:58] S1: Why do you think people auto diagnose themselves?

[00:52:01] S2: She was sick and I asked what do you have. I thought she just had the flu she said no I have Zika.

[00:52:08] S4: And how did she know?

[00:52:09] S2: She did not go to the doctor.

[00:52:16] S1: Why does the community...

[00:52:17]

[00:53:10] S1: Why do people, let's say, when is one of those diseases that are transmitted

[00:53:17] S1: by the mosquito, people know it is because the mosquito, or there's a lot of people

[00:53:24] S2: Normally people know that is supposedly because of the mosquito. But there's other people that say

[00:53:31] S2: that the Zika is contagious. I have heard that it is contagious. That if you get it in your house, everyone gets it.

[00:53:38] A: Is with sexual contact or by the mosquito.

[00:53:42] S2: Yes? I heard that it was contagious. But people then if she gets it and she took it

[00:53:49] S2: and the sister got it than it was supposedly here that gave it to her. This is what I hear from the neighborhood.

[00:54:03]

[00:55:30]

[00:56:00]

[00:57:00]

[00:58:00]

[00:59:00]

[00:59:37] S1: Why did the community come in agreement that in parks

[00:59:43] S1: they had to use tires, for example.

[00:59:47] S1: Knowing that it is a breeding site of Dengue.

[01:00:00] S2: I head a case where supposedly when they send a letter or something to ask for

[01:00:09] S2: A petition or a project that when it is not approved or doesn't work

[01:00:14] S2: I have an understanding that they use those mediums. To get tires, then

[01:00:20] S2: That happens when the community by its own looks for ways to close their parks.

[01:00:28] S1: And for cheaper.

[01:00:28] S2: Cheaper, because they looked and knocked on doors and could not find a way

[01:00:34] S2: That is one of the reasons I have heard when that happens.

[01:00:40] S1: Because almost all of the parks we saw have tires. And buried tires or laid down that puddles there.

[01:00:49] S2: Another of the reasons that they use those tires is for the kids supposedly,

[01:00:55] S2: that they are happy jumping on top of those tires and they paint them and

[01:01:01] S2: they find it captivating. But now with how many diseases

[01:01:07] S2: But in the moment they think of that. In the kids and how the tires are going to let them have fun but when I took my daughter to the park

[01:01:13] S2: She was happy jumping thought those tires. The parks also do it thinking of the kids.

[01:01:19] S2: They are the two options that I have heard.

[01:01:31] S3: That is what was used before. And there are still container of all that.

[01:01:36] S3: Since it was so hard, since everyone needed a good political figure to

[01:01:42] S3: so they could better it. Then people would recur to that.

[01:01:50] S3: The tires, and with a machine and that is

[01:01:55] S1: And in relation to, that we saw a lot of trash

[01:02:01] S1: That is also considered that it elongates the time of the mosquito.

[01:02:07] S1: How are the neighborhoods in that sense?

[01:02:11] S2: That is like for each their own. Because at least in the neighborhood.

[01:02:17] S2: of the Aguas Blancas district, because I do not know about the more far away ones, the garbage truck passes three times a week. And I think

[01:02:23] S2: that with three days that is pass is more than enough so the trash does not pile up. But what happens, there's people that if the trash

[01:02:30] S2: was left, they pay someone, the ones who go on the streets with carts,

[01:02:36] S2:they pay someone and the person receives the money but they throw the trash anywhere.

[01:02:43] S1: And the canals, no? The canals have furniture

[01:02:44] S2: That happens with the people that since they weren't in the house or the trash passed

[01:02:52] S2: because they were sleeping a lot of cases. Then what do they do. They take advantage when there is no one and

[01:02:59] S2: They pay the kids to go take the trash out. But take case is from our behavior

[01:03:06] S2: And that is not something that came someone to throw it out there.

[01:03:15] NO-

[01:03:25] S1: Then, let's say what factor you guys have identified, well with the video

[01:03:31] S1: what we can see which are the parks with tires, sinks

[01:03:37] S1: which you said is getting in the community, the factor that are

[01:03:43] S1: from my daily living, my behaviors in the house

[01:03:49] S2:It would also be good, that in a future

[01:03:55] S2: Going directly to the schools. The schools also have those sinks of mosquitoes

[01:04:01] S2: Even thought they teach kids about it and tell them to it but if you go around

[01:04:07] S2: There are a lot buckets where they wash the mops, when I go to meetings

[01:04:12] S2: in my girls school and there are puddles there, they washed the mop and left it there with the puddle

[01:04:18] S2: Or it has a cover on it, they covered the siphon and there it is. I do not know if there is no money from the school to call someone

[01:04:23] S2: to fix that siphon or that canal. The sink, all of that influences, that's why it would be good to go in.

[01:04:31] S1: What mechanism do you guys search for, knowing that there is a vision for secretary, right?

[01:04:38] S1: What mechanisms do you think that could get you closer that the secretary could use more the leaders.

[01:04:43] S1: What resources would you think would be good for the community.

[01:04:51] S1: Well, in this cases, that were found here, circumstantial. But the community as a collective construction

[01:04:57] S1: To demand to the state, the secretary, yes. that they noticed those.

[01:05:08] S3: What happens is the following...

[01:05:17] S3: There are political figures that do not even know the district.

[01:05:37] S3: Then since they do not know the place or care about it, then they put obstacles

[01:05:45] S3:  to all projects.

[01:06:01]

[01:07:07]

[01:07:52] S1: Has there been collective constructions that come out front he community to manage those processes

[01:07:58] S2: Through the community board.

[01:09:10]

[01:11:06]

[01:12:03]

[01:13:03]

[01:14:02]

[01:15:02]

[01:16:02]

[01:17:00]

[01:19:00]

[01:21:00]

[01:23:00]

[01:25:00]
