# Supplementary material for: Community context and sub-neighborhood scale detail to explain dengue, chikungunya and Zika patterns in Cali, Colombia
Source: PLoS One. 2017 Aug 2;12(8):e0181208. doi: 10.1371/journal.pone.0181208 (PMC5540594; doi:10.1371/journal.pone.0181208)
Supplement: S2 Text — SVG interviews were semi-structured and led by the local expert or leader. These questions were used to prompt leaders to point risks related to arboviral transmission in the neighborhood. 10.6084/m9.figshare.5197315. (DOCX) [file pone.0181208.s003.docx]

***Chikungunya and Dengue in Cali, Colombia: epidemiological and geospatial analyses***

Semi-structured interviews with community health workers

1. Start each interview with

- Informed consent including the purpose of the project and the interview

- Name of the 1. community health worker and 2. technician

- Date and place of the interview

- The experience of the community health worker in the neighborhood (time working and / or living in the neighborhood or surrounding areas)

2. Let community health workers lead the interview and ask about the following:

- risks for dengue, chikungunya and zika in the neighborhood or around

- location of possible vector breeding sites in or around the neighborhood

- location of clinics in or around the neighborhood

- changes in vector control with Zika in the neighborhood or around

- changes in water with rains in the neighborhood or around (breeding grounds, water rationing, canals)

- current or historical situation of dengue, chikungunya or Zika diseases in the neighborhood or around

- What else needs to be done to control the vector / disease in the neighborhood or around

3. Ask about points of interest in the neighborhood or surrounding areas
