# Supplementary material for: Community context and sub-neighborhood scale detail to explain dengue, chikungunya and Zika patterns in Cali, Colombia
Source: PLoS One. 2017 Aug 2;12(8):e0181208. doi: 10.1371/journal.pone.0181208 (PMC5540594; doi:10.1371/journal.pone.0181208)
Supplement: S3 Text — All SVG interviews were transcribed and translated. We used examples taken from these expert narratives throughout the paper to illustrate the various points being raised. CHW = community health worker, VC = vector control specialist. 10.6084/m9.figshare.5172970. (DOCX) [file pone.0181208.s004.docx]

1 CHW3: “The administration never considers the leaders. Never… [They do] not call the leaders to look for people to go with us and show us where the hotspots are…If you look at the 70% of officials [in the vector campaign] that they named, they are afraid to go the neighborhoods, the sidewalks. They're afraid.”

2 VC1: “What happens is that as x neighborhood is so commercial, if there are cases there, everybody goes to their house and in their house, they look for remedy and they don't report it maybe, no?”

CHW1: “It was the mothers who said ‘I have chikungunya.’ Because the medical diagnosis of chikungunya didn't reach us.”

CHW1: “[The hospital] fills up and it maintains that way… [The health center] took someone and they did not want to serve them… Look, I haven’t been there a lot, but people go, I did not go …You know, I went there once and I remember I had dengue. I was burning with fever and with a bad headache with shivers but I did not have any rash or anything. My husband lost his job and did not have EPS [health insurance]. Then I went there and they told me I needed an identification card and I told them my husband’s situation—I had [insurance], but since my husband lost his job I didn’t [anymore]. ‘Sign up first’ they said, and I told them that ‘sure, my sickness will wait while my husband comes and gets settled, or while I do the SISBEN’... Hi, CHW2, come and tell us the experience you had with the health center when— I told them you had chikungunya 15 days ago, and you went to the health center and they said… CHW2: “They told me they could not tend to me because I had to go to the church there…They told me, I have the SISBEN [public health insurance] there and I had to go.”

3 VC1: “Look how the canal is. It is completely stagnant. You know why? Because of the construction more ahead. And look at the larvae how you can see them there.” Interviewer1: “All that movement is larva?” Vc1: “Yes. Yes, look at how they are moving in the water. [It] is because they are fixing something more ahead and the machine is inside in the middle.” Interviewer1: “But for many months?” VC1: “Yes.”

CHW1: “Yes, we here in x neighborhood, by the x point, we have a canal that we have suffered from because in the canal you get close at four or five in the afternoon and see thousands and thousands of mosquitoes.”

Interviewer1: “The large part of the cases is in these sectors.” CHW1: “Well, I blame the canals a lot. Because that canal, I would like for us to pass in the truck because it is full of dirty water. Go look at it and take a picture and you will see the number of mosquitoes there. They are thousands. They are small and they are big. And you see some mosquitoes and their legs are white. Those white legs.”

4 CHW1: “[In] the old constructions that they don't finish, puddles form in the cement. Sometimes, they don't continue.” Interviewer1: “This makes that it is more difficult to control the vector, no.” CHW1: “Of course. Because they don't climb up there to see if there are puddles of water. Then there stays the vector.”

5 CHW1: “I had chikungunya. It started with itching and then a rash and that—I had a small fever. And I had joint pain…When I went to stand up, I couldn’t because of the pain in my knee and I fell back into the bed. Then I told my son to help me stand up and he did but I walked like a robot, because I could not take care of myself. I couldn’t with the pain.”

6 CHW2: “They [, the patients] say ‘Oh, why am I going to go there [to the clinic]. No, with these pains and fever to go make lines... No, I’ll stay here in my house… [they will] send me to buy Acetaminophen and I do not have anything to buy it with.’ Then that is why we [informal community health workers] go to them. Because the [community] leaders told us, ‘we have this problem [of underdiagnoses]’.”

7 CHW3: “What I see is that a lot of cases were presented [with] symptomatology but no diagnosis…Simply because people would say they had articulation pain, [they would say] ‘I have fever, it’s dengue’….” CHW1: “…they got the incapacity and the doctor said ‘this is chikungunya,’ but the diagnosis that they had was to discard the dengue, …they came back [and] they said it was a chikungunya for the symptoms. That is to say that they knew the symptoms of chikungunya, so they told us it was a chikungunya. [They said] ‘I got a rash, my joints hurt,’ so they assumed it was a chikungunya. But a diagnosis, like a medical diagnosis, we didn't find...” Interviewer1: “Then the cases that you all had were not registered?” CHW: “eh, no they were not registered.”
